# Opioid prescription patterns and pain severity among patients with opioid use disorder and other substance use disorders: a mixed methods study

**DOI:** 10.1097/PR9.0000000000001261

**Published:** 2025-04-18

**Authors:** Erin F. Madden, Gintare Daulys, Benjamin Tingey, Felicia Frabis, Pooja Lagisetty, Philip J. Kroth, Fares Qeadan

**Affiliations:** a Department of Family Medicine and Public Health Sciences, Wayne State University, Detroit, MI, USA; b Parkinson School of Health Sciences and Public Health, Loyola University Chicago, Maywood, IL, USA; c Department of Internal Medicine, University of Michigan, Ann Arbor, MI, USA; d Center for Clinical Management and Research, Ann Arbor Veterans Health Administration, Ann Arbor, MI, USA; e Department of Biomedical Informatics, Western Michigan University, Homer Stryker M.D. School of Medicine, Kalamazoo, MI, USA

**Keywords:** Opioid prescription patterns, Pain severity, Substance use disorders, Opioid use disorder, Mixed methods, Numeric rating scale, Complementary medicine, Medications for opioid use disorder

## Abstract

**Objective:**

Pain management for patients with substance use disorders (SUDs) poses challenges, yet little is known about their pain severity perceptions. We conducted a mixed-methods study to understand numeric pain severity reporting among patients with SUD. We assessed how interactions between opioid prescribing and time, medications for opioid use disorder (MOUD), complementary medicine (CM), and outpatient SUD-related services (OS) predict differences in reported pain severity among patients with SUD.

**Methods:**

We analyzed electronic health records (EHR) data, from 2003 to 2023, among the *Oracle EHR Real-World Database*, which comprised 141 US health systems from 50 states. We used longitudinal patient-reported numeric rating scale pain severity scores in linear mixed effects regressions to predict mean pain severity scores. We also conducted 2 focus-groups, from 2022 to 2023, with patients with SUD and SUD-treating clinicians aiming to explore pain severity reporting.

**Results:**

Overall, predicted mean pain scores (95% confidence interval) were highest for patients with OUD (4.52 [4.51, 4.53]), lower for other patients with SUD (3.80 [3.79, 3.81]), and lowest for controls (3.28 [3.27, 3.29]) across all opioid prescription doses and durations. Complementary medicine use was associated with lower pain scores for all patients prescribed opioids. Pain scores were also lower for patients with OUD and SUD prescribed opioids and using OS, and for patients with OUD treated with MOUD and prescribed higher dose opioids. Focus-groups revealed pain reporting is influenced by anticipated clinician disbelief, treatment changes, and stigma.

**Conclusion:**

These findings highlight the need to address pain within addiction treatment and further examine effective pain management interventions for this complex patient population.

## Abstract 2

Supplemental Digital Content is Available in the Text.

Opioid prescribing patterns, combined with complementary medicine and outpatient services, significantly influence pain severity reporting among patients with opioid use and other substance use disorders.

## 1. Introduction

Substance use disorder (SUD) and chronic pain commonly co-occur.^54^ Chronic pain affects 21% of the US population^64^ but 75% of people with opioid use disorder (OUD) and 59% with alcohol use disorder (AUD).^41^ Untreated acute or chronic pain can contribute to patients returning to drug use,^24,25^ highlighting the importance of adequate pain management among people with SUDs. Clinicians often find effective pain management for patients with SUDs to be challenging, as it requires balancing adequate pain treatment and minimization of risks associated with opioid prescribing and substance use.^69^

Opioids have been prescribed for pain management more cautiously in recent years due to increases in opioid-related morbidity and mortality^11^ and research showing limited efficacy, including in pain intensity among individuals with chronic pain.^15^ Consequently, alternative interventions have been emphasized, as evidenced by the Centers for Disease Control and Prevention (CDC) clinical practice guidelines for opioid prescribing for chronic pain released in 2016^20^ and updated in 2022^21^ prioritizing nonpharmacologic therapies. The guidelines urged use of multimodal pain treatment and SUD treatment through behavioral health interventions and medications for opioid use disorder (MOUD) to manage chronic pain in patients with co-occurring SUDs. They also encouraged assessment of clinically meaningful improvement in pain using patient-reported pain intensity and interference scales. As the use of numeric pain assessment has become a common practice, assessing pain scores among patients with SUDs may provide useful information about their perceptions of clinical pain management.

Although many studies assess the efficacy of pain management using patient-reported severity,^10^ there is limited study of such outcomes among patients with SUDs. As with other patient-reported measures, numeric pain scores are complex and subjective. Numeric pain scores may be influenced by biological and social factors, including co-occurring depression,^34^ perceived social support,^26^ and power dynamics within clinical interactions, with some patients reporting more intense pain to ensure it is taken seriously.^4^ Patients with SUDs also frequently anticipate stigma due to their substance use from clinicians,^33,75^ complicating their potential responses to diverse forces influencing pain scores. Nevertheless, numeric pain scores are readily captured in electronic health records (EHR)^59^ and can thereby be useful in examining changes in pain over time among patients with SUDs.

This mixed-methods study uses qualitative focus groups and quantitative longitudinal analyses of EHR data to better understand numeric pain score reporting among patients with SUDs. Triangulating data from these sources facilitates a multifaceted understanding of how to interpret this category of patient-reported measures among a population of patients with high pain prevalence who are subject to complex psycho-socio-legal forces due to their SUD.

## 2. Methods

### 2.1. Mixed methods study design

This study used a concurrent triangulation design in which quantitative longitudinal and qualitative data were gathered and analyzed simultaneously.

### 2.2. Quantitative data source

This study used a retrospective, deidentified, EHR data set from the *Oracle EHR Real-World Data* (*OERWD*). As of September 2023 refresh (updated quarterly), 141 US health systems contribute to OERWD providing data for over 111 million patients across 50 states. Oracle EHR Real-World Data is extracted from the EMR of hospitals in which Oracle has a data use agreement. Encounters may include pharmacy, clinical and microbiology laboratory, admission, and billing information from affiliated patient care locations. All admissions, medication orders and dispensing, laboratory orders, and specimens are date and time stamped, providing a temporal relationship between treatment patters and clinical information. Oracle has established Health Insurance Portability and Accountability Act–compliant operating policies to establish deidentification for Oracle EHR Real-World Data.^23^

### 2.3. Quantitative sample

Patients were included in this study if they had ≥3 numeric rating scale (NRS) pain severity scores over ≥3 unique days within 2 years and were ≥12 years old at first pain severity score. At least 3 scores over 3 days were required to establish a trajectory.^58^ In addition, a 2-year time period was chosen to ensure that calculated trajectories were not incorporating pain scores substantially temporally separated. Patients were further classified as having OUD or other SUD (non-OUD) if they had qualifying codes (Supplemental Table 1, http://links.lww.com/PR9/A294) up to 2 years before first pain severity score or within 6 months after. The time window was chosen to ensure that those included were more likely to have ongoing SUD conditions that were more recent to pain reporting. Control patients with no qualifying codes for OUD or other SUD were matched, using a greedy exact matching algorithm with a 1 to 1 ratio, on year of first pain severity score and hospital ID.

### 2.4. Quantitative study design

**Figure 1.**
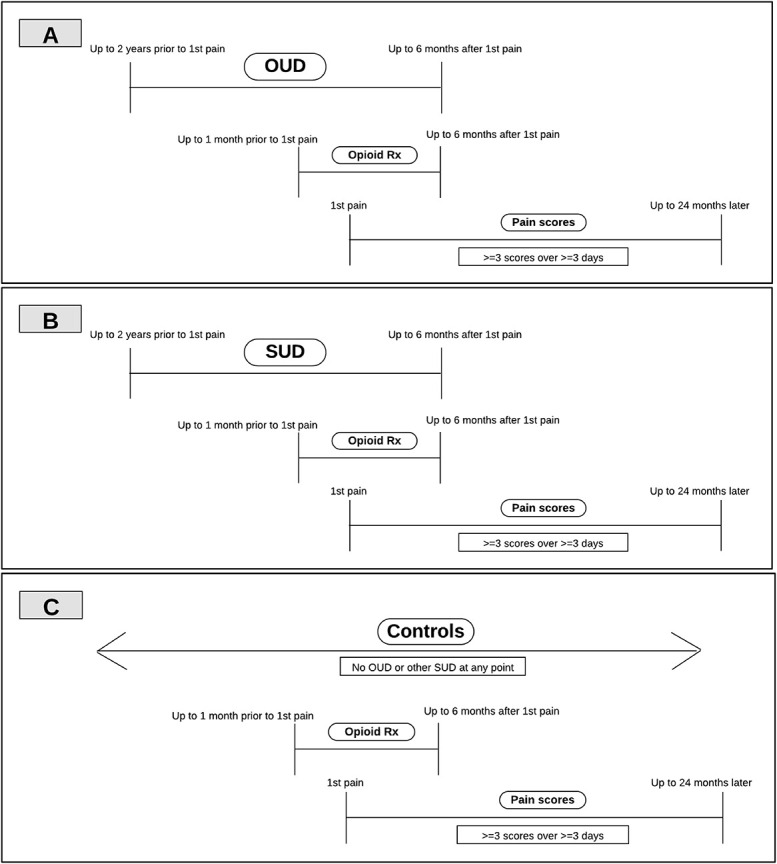
(A–C) Cohort, primary exposure, outcome time windows for (A) OUD, (B) other SUD, and (C) controls. OUD, opioid use disorder; SUD, substance use disorder.

This is a longitudinal, retrospective, cohort study with patients followed from first pain severity score up to 2 years later. Pain severity encounters ranged from January 2003 to March 2023, with the end date cutoff chosen to allow those lastly recruited to have at least 6 months of follow-up to allow the maximum time window to identify opioid prescribing exposure. This approach maximized available patients yet did not allow those lastly recruited to have a full 2 years of possible follow-up. Analyses were repeated while ending inclusion September 2021, to allow those lastly recruited to have a full 2 years of follow-up, and revealed similar findings to those of the main analysis. Figure 1A–C displays a visual representation of the cohort, primary exposure, and outcome time windows.

### 2.5. Quantitative measures

The primary outcome was the NRS pain severity score. The NRS is a validated pain screening tool used to assess pain severity at the moment of capture, ranging from 0 to 10, with 0 corresponding to “no pain” and 10 corresponding to “the worst pain imaginable.”^8,38,39^

The primary exposure was opioid prescribing, consisting of indications for both dosing and duration, with qualifying prescriptions (Supplemental Table 2, http://links.lww.com/PR9/A294) occurring up to 1 month before first pain severity score and up to 6 months after. The time window was chosen to focus on opioid prescribing that was timely with ongoing pain management. Dosing was defined by daily morphine milligram equivalents (MME)^13^ of qualifying prescriptions with the median MME captured for multiple daily prescriptions across the time window and duration was defined as the difference in maximum prescription stop date and minimum prescription start date. Thus, dosing was a categorical indication with levels of (1) no opioid prescription, (2) MME < 50, or (3) MME ≥ 50. Duration was a categorical indication with levels of (1) no opioid prescription, (2) duration <30 days, (3) duration ≥30 days and ≤90 days, or (4) duration >90 days. The dosing and duration categories were chosen based on CDC opioid prescribing guidelines indicating commonly used dosing cutoffs and general duration time windows of treating acute, subacute, and chronic pain.^21^

### 2.6. Additional quantitative measures

Additional demographic measures included the continuous age (in years), gender, race/ethnicity, census region, rurality, metropolitan status, and insurance. Clinical measures included year of first pain severity score, time (in months) to ensuing pain severity scores, baseline comorbidity (Charlson comorbidity index [CCI]; Supplemental Table 3, http://links.lww.com/PR9/A294),^14^ chronic pain (up to 1 year before first pain severity score and up to 6 months after; Supplemental Table 4, http://links.lww.com/PR9/A294),^3,12,40,51,62,65,79^ procedure history (up to 1 year, commonly associated with opioid prescribing, Supplemental Table 5, http://links.lww.com/PR9/A294),^6,9,16–19,27,28,30,31,36,43,44,47,48,50,56,57,66,70–72^ history (and count) of mental health conditions (Supplemental Table 6, http://links.lww.com/PR9/A294), indication (and count) of non-OUD SUDs, MOUD and substance use disorder (MSUD; inclusive of MOUD) treatment taking place on or after OUD/other SUD diagnosis and up to 2 years after first pain severity score (Supplemental Table 7, http://links.lww.com/PR9/A294), complementary medicine (CM) encounter taking place up to 1 year before first pain severity score and up to 2 years after (Supplemental Table 8, http://links.lww.com/PR9/A294), and outpatient SUD-related services (OS) encounter taking place up to 1 year before first pain severity score and up to 2 years after (Supplemental Table 9, http://links.lww.com/PR9/A294).^32^

### 2.7. Statistical analysis

All analyses were stratified by OUD, other SUD, and controls. Demographic and clinical characteristics were displayed. The association between opioid prescribing and reported pain severity was assessed with mixed-effects linear regressions. Models were adjusted with previously mentioned variables and incorporated patient ID and hospital ID as crossed random effects. In addition, interactions of interest were investigated between opioid prescribing and (1) time (in months) since first pain severity encounter (for each ensuing pain severity score), (2) MOUD treatment (only for those with OUD), (3) CM, and (4) OS (only for those with OUD or other SUD). Significant interactions were kept and indicated that the relationship between opioid prescribing and reported pain severity depended on presence/absence of treatment as well as across time. Average model predicted pain severity scores, with 95% Wald confidence intervals (CIs), were used to present results visually as well as tabularized to make comparisons between groups. Statistical significance was determined by nonoverlapping 95% CIs. A supplemental analysis was conducted in which all analyses were repeated only for those patients with chronic pain. All hypothesis tests were 2-sided with a significance level of 5%, and analyses were conducted in R version 4.0.2 (R Foundation for Statistical Computing).

### 2.8. Qualitative data collection and analysis

The qualitative portion of the study was guided by the Qualitative Description Approach.^5^ Purposive sampling was used to recruit 8 individuals with experience in chronic pain and SUDs. Six participants were patients with co-occurring SUD and chronic pain, and 2 participants were clinicians with experience treating this population. Two focus groups were conducted in 2022 to 2023, each lasting 2 hours and led by a topic guide soliciting experiences with opioid and nonopioid pain management and pain severity reporting. Two coders used thematic analysis^7^ to analyze the transcribed data.

## 3. Results

### 3.1. Descriptive statistics

The study identified 179,631 patients with OUD, 557,991 patients with other SUDs, and 737,213 controls. Most of the participants were female, from the Western US Region, had either private insurance or Medicare or Medicaid, and had a first pain severity score in a clinical encounter on or after 2017. Patients with OUD were more burdened by disease and clinical conditions, compared to those with other SUDs and controls, regarding higher comorbidity, chronic pain, and number of mental health conditions. Among those with OUD, 8.1% were treated with MOUD, and among those with other SUD, 8.5% were treated with MSUD. There were 3.2% and 6.8% of patients with OUD who used CM and OS services, respectively, whereas 2.4% and 9.7% of patients with other SUD used CM and OS and 1.8% of control patients used CM. Opioid prescribing was also variable across groups, with patients with OUD prescribed opioids more frequently, at higher doses, and for longer durations compared to other groups (Table 1).

**Table 1:** Patient demographic and clinical characteristics among those diagnosed with opioid use disorder/other substance use disorder up to 2 years prior or within 6 months of first pain severity score and controls (without opioid use disorder/substance use disorder diagnoses), among patients with ≥3 pain severity scores over ≥3 unique days within 2 years, among Oracle electronic health record real-world data-affiliated health systems, ≥12 years old, January 2003 to March 2023.

Characteristic	OUD	Other SUD	Control*
	n (%)†	n (%)†	n (%)†
Overall	179,631	557,991	737,213
Age (y), mean (SD‡)	53.56 (17.86)	48.72 (17.69)	51.29 (21.42)
Gender			
Female	100,080 (55.7)	266,008 (47.7)	468,568 (63.6)
Male	79,551 (44.3)	291,983 (52.3)	268,645 (36.4)
Race/Ethnicity			
NH-AI/AN§	2980 (1.7)	13,073 (2.3)	11,650 (1.6)
NH-API‖	794 (0.4)	3426 (0.6)	11,223 (1.5)
NH-Black	16,104 (9.0)	80,007 (14.3)	69,730 (9.5)
Hispanic	33,956 (18.9)	116,165 (20.8)	172,780 (23.4)
NH-White	119,639 (66.6)	320,128 (57.4)	427,951 (58.0)
NH-Other/Unknown	6158 (3.4)	25,192 (4.5)	43,879 (6.0)
Census region			
Northeast	17,445 (9.8)	82,423 (15.0)	100,201 (13.8)
Midwest	8502 (4.8)	40,788 (7.4)	50,199 (6.9)
South	33,953 (19.1)	198,173 (36.1)	231,490 (31.9)
West	118,314 (66.4)	226,911 (41.4)	344,017 (47.4)
Rurality			
Rural	21,147 (11.8)	79,984 (14.4)	101,158 (13.8)
Urban	157,642 (88.2)	474,378 (85.6)	632,239 (86.2)
Metropolitan			
Metropolitan	160,100 (89.5)	477,813 (86.2)	638,259 (87.0)
Nonmetropolitan	18,689 (10.5)	76,546 (13.8)	95,118 (13.0)
Insurance			
Private	29,644 (16.8)	139,738 (25.6)	246,987 (34.5)
Medicare	53,377 (30.2)	132,114 (24.2)	207,147 (29.0)
Medicaid	53,839 (30.5)	164,686 (30.1)	127,009 (17.8)
Other Govt/Misc	10,314 (5.8)	27,801 (5.1)	48,460 (6.8)
Self-pay	29,405 (16.7)	82,377 (15.1)	85,870 (12.0)
Year of first encounter			
≤2010	310 (0.2)	2825 (0.5)	3135 (0.4)
2011–2012	4868 (2.7)	19,232 (3.4)	24,100 (3.3)
2013–2014	8467 (4.7)	36,908 (6.6)	45,375 (6.2)
2015–2016	31,713 (17.7)	73,420 (13.2)	105,133 (14.3)
2017–2018	47,870 (26.6)	137,048 (24.6)	184,918 (25.1)
2019–2020	42,120 (23.4)	151,337 (27.1)	193,444 (26.2)
2021–2023	44,283 (24.7)	137,221 (24.6)	181,108 (24.6)
CCI#			
0	81,666 (45.5)	375,110 (67.2)	564,195 (76.5)
1–2	37,632 (20.9)	93,559 (16.8)	104,874 (14.2)
3–4	26,820 (14.9)	53,915 (9.7)	42,836 (5.8)
≥5	33,513 (18.7)	35,407 (6.3)	25,308 (3.4)
Chronic pain** (Yes)	144,493 (80.4)	309,257 (55.4)	330,929 (44.9)
Procedure history†† (Yes)	10,392 (5.8)	24,254 (4.3)	42,876 (5.8)
History of mental health conditions‡‡ (Yes)			
Anxiety	48,844 (27.2)	67,294 (12.1)	42,290 (5.7)
Depression	46,643 (26.0)	65,296 (11.7)	39,034 (5.3)
ADD/ADHD§§	3134 (1.7)	8181 (1.5)	4958 (0.7)
Bipolar	14,992 (8.3)	22,406 (4.0)	6787 (0.9)
Schizophrenia/Psychotic	5234 (2.9)	8462 (1.5)	2240 (0.3)
PTSD‖‖	6582 (3.7)	9396 (1.7)	2906 (0.4)
Other	5427 (3.0)	10,541 (1.9)	5579 (0.8)
No. of mental health conditions			
0	108,018 (60.1)	443,099 (79.4)	665,374 (90.3)
1	33,420 (18.6)	64,280 (11.5)	47,646 (6.5)
2	24,155 (13.4)	32,454 (5.8)	18,238 (2.5)
≥3	14,038 (7.8)	18,158 (3.3)	5955 (0.8)
Substance use disorders## (Yes)			
Alcohol	16,839 (9.4)	125,550 (22.5)	—
Tobacco	46,317 (25.8)	392,823 (70.4)	—
Cannabis	14,598 (8.1)	60,815 (10.9)	—
Sedatives	6602 (3.7)	8749 (1.6)	—
Stimulants	18,096 (10.1)	34,807 (6.2)	—
Hallucinogens	780 (0.4)	1858 (0.3)	—
Inhalants	104 (0.1)	318 (0.1)	—
Psychotropic medications	19,562 (10.9)	33,100 (5.9)	—
Other SUD	8616 (4.8)	15,597 (2.8)	—
No. of SUDs			
0	103,866 (57.8)	—	—
1	42,364 (23.6)	469,099 (84.1)	—
2	18,646 (10.4)	68,541 (12.3)	—
≥3	14,755 (8.2)	20,351 (3.6)	—
MOUD*** (Yes)	14,534 (8.1)	—	
MSUD††† (Yes)	—	47,215 (8.5)	—
CM‡‡‡ (Yes)	5713 (3.2)	13,640 (2.4)	13,638 (1.8)
OS§§§ (Yes)	12,284 (6.8)	54,234 (9.7)	—
Opioid Rx dosing‖‖‖			
No opioid Rx	40,887 (22.8)	274,217 (49.1)	399,659 (54.2)
MME < 50	115,721 (64.4)	252,309 (45.2)	287,797 (39.0)
MME ≥ 50	23,023 (12.8)	31,465 (5.6)	49,757 (6.7)
Opioid Rx duration###			
No opioid Rx	40,887 (22.8)	274,217 (49.1)	399,659 (54.2)
<30 d	52,205 (29.1)	162,620 (29.1)	221,769 (30.1)
≥30 d and ≤90 d	18,810 (10.5)	36,445 (6.5)	35,799 (4.9)
>90 d	67,729 (37.7)	84,709 (15.2)	79,986 (10.8)

* Matched on year of first pain severity score and hospital ID.

† Frequency (values may not add up to totals due to removal of missing rows) and column %'s (unless otherwise noted).

‡ Standard deviation.

§ American Indian or Alaskan Native.

‖ Asian or Pacific Islander.

# Charlson comorbidity index, calculated from conditions before first pain severity score.

** Up to 1 year before first pain severity score date or up to 6 months after.

†† Looking across ∼5 procedures within each of 13 surgical departments associated with higher opioid Rx, on or up to 1 year before first pain severity score date.

‡‡ Any condition before first pain severity score date.

§§ Attention-deficit disorder, attention-deficit/hyperactivity disorder.

‖‖ Post-traumatic stress disorder.

## Any condition up to 2 years before first pain severity score date and up to 6 months post.

*** Medications for opioid use disorder, taking place on or after OUD diagnosis and up to 2 years after first pain severity score.

††† Medications for substance use disorder (inclusive of MOUD), taking place on or after SUD diagnosis and up to 2 years after first pain severity score.

‡‡‡ Complementary medicine taking place up to 1 year before first pain severity score and up to 2 years after.

§§§ Outpatient SUD-related services taking place up to 1 year before first pain severity score and up to 2 years after.

‖‖‖ Opioid prescription dosing, consisting of prescriptions from up to 1 month before first pain severity score and up to 6 months later, median daily morphine milligram equivalents (if multiple).

### Opioid prescription duration, consisting of prescriptions from up to 1 month before first pain severity score and up to 6 months later, duration is difference in maximum stop date and minimum start date of prescriptions in that time frame.

CM, complementary medicine; MOUD, medications for opioid use disorder; OUD, opioid use disorder; SUD, substance use disorder.

### 3.2. Predicted mean pain severity

Overall, those with OUD had the highest model predicted mean (95% CI) pain severity score at 4.52 (4.51, 4.53) while those with other SUD reported a lower mean at 3.80 (3.79, 3.81), and controls had the lowest at 3.28 (3.27, 3.29) across 2 years of follow-up (Table 2).

**Table 2:** Predicted* mean pain severity scores (95% confidence intervals) by opioid Rx dosing/duration† and treatment, among patients with opioid use disorder, other substance use disorder, controls (without opioid use disorder or substance use disorder), on and up to 2 years after first pain severity score.

	Opioid Rx						
	No opioid Rx	MME < 50, duration <30 d	MME ≥ 50, duration <30 d	MME < 50, duration ≥30 d and duration ≤90 d	MME ≥ 50, duration ≥30 d and duration ≤90 d	MME < 50, duration >90 d	MME ≥ 50, duration >90 d
OUD							
n (%‡)	40,887 (22.8)	43,931 (24.5)	8274 (4.6)	16,318 (9.1)	2492 (1.4)	55,472 (30.9)	12,257 (6.8)
Mean (95% CI)	Overall = 4.52 (4.51, 4.53)						
MOUD§							
No	3.74 (3.73, 3.75)	4.28 (4.27, 4.29)	4.51 (4.50, 4.52)	4.47 (4.46, 4.48)	4.62 (4.61, 4.63)	4.75 (4.74, 4.76)	4.83 (4.82, 4.84)
Yes	3.82 (3.81, 3.83)	4.33 (4.32, 4.34)	4.30 (4.28, 4.32)	4.70 (4.69, 4.71)	4.59 (4.54, 4.63)	4.90 (4.89, 4.91)	4.79 (4.77, 4.81)
CM‖							
No	3.74 (3.73, 3.75)	4.30 (4.29, 4.31)	4.51 (4.50, 4.52)	4.50 (4.49, 4.51)	4.60 (4.59, 4.61)	4.77 (4.76, 4.78)	4.76 (4.75, 4.77)
Yes	3.97 (3.96, 3.98)	3.99 (3.98, 4.00)	4.19 (4.16, 4.23)	4.07 (4.05, 4.08)	4.10 (4.06, 4.14)	4.45 (4.44, 4.46)	4.51 (4.59, 4.53)
OS#							
No	3.73 (3.72, 3.74)	4.29 (4.28, 4.30)	4.51 (4.50, 4.52)	4.49 (4.48, 4.50)	4.60 (4.59, 4.61)	4.76 (4.75, 4.77)	4.77 (4.76, 4.78)
Yes	3.99 (3.98, 4.00)	4.21 (4.20, 4.22)	4.30 (4.28, 4.33)	4.40 (4.39, 4.41)	4.23 (4.19, 4.27)	4.68 (4.67, 4.69)	4.46 (4.45, 4.48)
R^2^	0.30						
Other SUD							
n (%‡)	274,217 (49.1)	144,314 (25.9)	18,306 (3.3)	33,142 (5.9)	3303 (0.6)	74,853 (13.4)	9856 (1.8)
Mean (95% CI)	Overall = 3.80 (3.79, 3.81)						
CM‖							
No	3.02 (3.01, 3.03)	3.94 (3.93, 3.95)	3.89 (3.88, 3.90)	4.27 (4.26, 4.28)	4.10 (4.09, 4.11)	4.51 (4.50, 4.52)	4.52 (4.51, 4.53)
Yes	3.16 (3.15, 3.17)	3.72 (3.71, 3.73)	3.47 (3.44, 3.50)	4.24 (4.21, 4.25)	3.72 (3.68, 3.76)	4.48 (4.47, 4.48)	4.48 (4.43, 4.51)
OS#							
No	3.01 (3.00, 3.02)	3.95 (3.94, 3.96)	3.91 (3.90, 3.92)	4.24 (4.23, 4.25)	4.13 (4.12, 4.14)	4.50 (4.49, 4.51)	4.48 (4.47, 4.49)
Yes	3.07 (3.06, 3.08)	3.70 (3.69, 3.71)	3.57 (3.56, 3.59)	4.04 (4.03, 4.05)	3.72 (3.69, 3.75)	4.32 (4.31, 4.33)	4.14 (4.13, 4.16)
R^2^	0.34						
Control							
n (%‡)	399,659 (54.2)	188,395 (25.6)	33,374 (4.5)	31,449 (4.3)	4350 (0.6)	67,953 (9.2)	12,033 (1.6)
Mean (95% CI)	Overall = 3.28 (3.27, 3.29)						
CM‖							
No	2.73 (2.72, 2.74)	3.53 (3.52, 3.54)	3.51 (3.50, 3.52)	3.73 (3.72, 3.74)	3.78 (3.77, 3.79)	3.95 (3.94, 3.96)	4.09 (3.06, 4.12)
Yes	2.74 (2.73, 2.75)	3.23 (3.22, 3.24)	3.11 (3.09, 3.14)	3.71 (3.69, 3.72)	3.75 (3.71, 3.79)	3.91 (3.90, 3.92)	3.95 (3.94, 3.96)
R^2^	0.31						

* Linear mixed-effects regression adjusting for age, gender, race, census region, insurance, year of first pain severity score, opioid Rx × time (in months) since first pain severity score, comorbidity, 1-year procedure history, mental health condition history, other SUD conditions (for OUD and SUD models), MSUD (for SUD model), with hospital ID and patient ID as crossed random effects (random effects accounted for clustering of repeated scores within patients as well as clustering resulting from patients visiting similar hospitals [yet allowing for patients to visit different hospital systems] and assumed unstructured covariance matrices), interactions tested with type III, χ^2^, tests.

† Prescriptions from up to 1 month before first pain severity score and up to 6 months later.

‡ Row % out of overall n.

§ Medications for opioid use disorder.

‖ Complementary medicine.

# Outpatient SUD-related services.

CM, complementary medicine; MOUD, medications for opioid use disorder; OUD, opioid use disorder; SUD, substance use disorder.

### 3.3. Predicted mean pain severity stratified by opioid prescribing and time since first pain severity score

**Figure 2.**
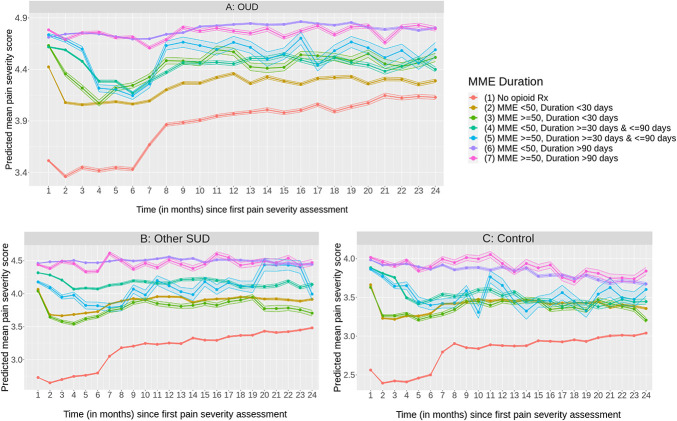
(A–C) Predicted^1^ mean pain severity scores (95% confidence intervals) vs time (in months) since first pain severity assessment (by opioid Rx dosing/duration^2^) among (A) patients with OUD; (B) patients with other SUD; (C) control patients (without OUD or SUD), on and up to 2 years after first pain severity score. ^1^Linear mixed-effects regression adjusting for age, gender, race, census region, insurance, comorbidity, 1-year procedure history, mental health condition history, other SUD conditions (for OUD and SUD models), opioid Rx × MOUD (for OUD model), MSUD (for SUD model), opioid Rx × CM, opioid Rx × OS (just for OUD and SUD model), with hospital ID and patient ID as crossed random effects. ^2^Prescriptions from first pain severity score and up to 6 months later. MOUD, medications for opioid use disorder; OUD, opioid use disorder; SUD, substance use disorder.

Across all groups and over time, those with no opioid prescription had the lowest predicted mean pain severity scores. Irrespective of dose (<50 or ≥50 MME), predicted pain severity was on average higher for low duration groups (<30 days) and was highest for high duration groups (>90 days). In addition, we observed patterns in mean pain severity across groups over time. The predicted mean pain severity was initially high for OUD and other patients with SUD at the first month from first pain severity encounter, then decreased and fluctuated at low levels up until 6 months from first encounter. However, after the 6-month period, mean pain severity increased and remained consistently high up until 2 years after first encounter. Similar trends were also observed among control patients except those with higher opioid prescription durations where mean pain severity levels decreased slightly over time (Fig. 2A–C, Supplemental Table 10a-10c, http://links.lww.com/PR9/A294).

### 3.4. Predicted mean pain severity stratified by opioid prescribing and treatment

Table 2 displays predicted mean pain severity scores stratified by opioid prescribing groups as well as by treatment groups. Among patients with OUD receiving higher opioid doses (MME ≥ 50), those receiving MOUD had lower predicted mean pain severity than those not treated with MOUD (MME ≥ 50, duration <30 days mean [95% CI] for MOUD: 4.30 [4.28, 4.32] vs no MOUD: 4.51 [4.50, 4.52]; MME ≥ 50, duration ≥30 days & ≤90 days mean [95% CI] for MOUD: 4.59 [4.54, 4.63] vs no MOUD: 4.62 [4.61, 4.63]; MME ≥ 50, duration >90 days mean [95% CI] for MOUD: 4.79 [4.77, 4.81] vs no MOUD: 4.83 [4.82, 4.84]). The higher dose/moderate duration group (≥30 days and ≤90 days) difference was not statistically significant. Patients receiving MOUD among all other prescribing groups had higher predicted mean pain severity than those not treated with MOUD. Among patients with OUD who were not prescribed any opioids, those receiving CM had higher predicted mean pain severity than those without CM. Yet, for those receiving opioids at any dose or duration, those using CM had lower predicted mean pain severity than those without. The same trend was seen with respect to OS, where patients prescribed any opioids who used OS reported lower mean pain severity than those who did not use OS. Similar trends were seen in patients with other SUDs and controls.

### 3.5. Supplemental analysis for patients with chronic pain

When restricting to patients with chronic pain, similar trends were observed across all groups as were observed in the primary analysis (Supplemental Figure 1A–C, http://links.lww.com/PR9/A294, Supplemental Table 11, http://links.lww.com/PR9/A294). However, mean pain severity was elevated in all groups. Comparing the overall group to only those with chronic pain, overall mean pain severity (95% CI) increased in the OUD group from 4.52 (4.51, 4.53) to 4.58 (4.57, 4.59), in the other SUD group from 3.80 (3.79, 3.81) to 3.98 (3.97, 3.99), and in the control group from 3.28 (3.27, 3.29) to 3.47 (3.46, 3.48).

### 3.6. Qualitative findings

Patients reported a variety of nonclinical influences on their pain scale reporting in interactions with clinicians. These influences included anticipation that clinicians would not believe high pain scores, fears of treatment changes, and expectations of stigma due to drug use. One White 67-year-old man on long-term opioid therapy and later diagnosed with OUD described trying to choose a number that conveyed the need for ongoing opioid treatment for chronic pain without appearing to seek drugs for illicit reasons: “*If a patient reports a high score, it might seem like they're drug seeking and may not get their medications. But if I report a low score, I might not get my medications*.” Another 34-year-old nonbinary person in recovery for OUD who later sought treatment for chronic pain described anticipating stigma when reporting higher pain severity,

“No drug user can use [pain scales] honestly… The moment you start hitting 10 more than once, then you're seen as a person that just wants opiates. It's no longer that I gave birth to triplets or have nerve damage, right? I'm just a [drug] user [to the clinician].”

Other patients on long-term opioid therapy resisted reporting lower pain scores when multidisciplinary nonopioid treatments were used and found to be effective because the patients feared clinicians would respond with opioid tapering. A 45-year-old Hispanic man on long-term opioid therapy and later diagnosed with multiple SUDs explained,

“After radiofrequency procedures the doctor asked where my pain's at right after the procedure and then at the follow up appointment. The radiofrequency treatments, they would help short term. But I also felt like if I told him it helped for a short period… that I wouldn't get [opioid] pain relief going forward…And I knew [radiofrequency] didn't help long term. It's not a permanent fix for my pain and I still needed some pain management. And so I would definitely lie on there.”

While these participants believed their pain scores played a significant role in clinical decision-making, another participant communicated pessimism about clinician responses to pain scales. A 64-year-old Black man with a history of OUD said,

“When I go to my doctor's appointments and they ask, “What's your pain level?” I tell them that my pain is at a five? They don't do nothing and want to lower my medicines. But if I tell them I'm at nine or ten, they don't do nothing. So, I don't think they pay attention to [pain scales] at all period.”

Pain scales may ideally capture a simple subjective patient assessment of pain severity, but these qualitative results illustrate additional complex upward and downward pressures that patients with SUDs feel when reporting numeric pain scores in interactions with clinicians.

## 4. Discussion

This mixed-methods study shows patients with SUDs, especially OUD, report higher pain severity than patients without SUDs, even as multiple social and interactional factors influence their pain reporting. Although qualitative results indicate some patients with SUDs underreport pain, anticipating clinician disbelief or treatment changes, quantitative analyses suggest that this patient population nevertheless reports pain severity at higher levels than other patients. Growing research suggests that pain management may be a key component of effective addiction treatment^77^ particularly OUD treatment because pain is associated with opioid craving.^74^ This study underscores pain severity among people with SUDs and the need for targeted pain management interventions for this population.

This study found patients with OUD had the highest predicted mean pain severity score, followed by those with other SUD, then controls. In addition to qualitative explanations provided by focus groups, which elucidated social and interactional dynamics with clinicians patients with SUD experienced, psychological reasons may contribute to this finding. Rates of comorbid major depression and anxiety disorders, eg, have been estimated at 28%–35% and 17%–30% among people with OUD, respectively.^68^ Pain and mental disorder frequently occur together,^2^ with pain intensity decreasing as anxiety symptoms are treated.^49^ Psychiatric disorders may contribute to our finding that more severe pain occurred among patients with OUD and SUD, who had higher prevalence of mental health conditions compared to controls.

Predicted mean pain severity scores were lowest for patients with no opioid prescriptions, higher with short-duration opioid prescriptions, and highest with long-duration prescriptions. This pattern may reflect provider adherence to opioid prescribing guidelines. Since opioids are intended for moderate to severe pain treatment,^11^ low pain severity scores with no opioid prescription could represent patients being treated with nonopioid interventions or whose pain level did not warrant a prescription. Similarly, the next highest predicted pain severities may reflect patients with more severe acute pain prescribed short-term opioid therapy, which aligns with guidelines suggesting that providers prescribe the lowest effective opioid dose for pain treatment, with acute pain generally treated for 3 or fewer days and rarely more than 7.^11^ Finally, the highest pain scores occurring among groups of patients with more than 30-day opioid prescriptions are likely driven by chronic pain treatment. Previous research has found higher pain intensity in patients with chronic pain treated with opioids compared to those not treated with opioids.^46^

The observed trends in predicted pain severity score demonstrated narrowest confidence intervals in the first few months after initial pain severity assessment, and predicted means decreasing over time for many opioid prescribing groups, across those with OUD, SUD, and controls. This suggests that most patients had better managed pain in the first months of pain reporting. The subsequent upward trend of predicted pain severity scores may be driven by patients with more persistent pain requiring lengthier treatment and those who have developed opioid tolerance. Wider predicted pain severity intervals in later months also suggest that these results stem from a smaller, more pain-burdened subset of patients. Control patients with higher opioid prescription durations exhibited slightly decreasing pain severity trends over time, consistent with literature showing better pain-related outcomes and higher likelihood for clinically significant improvements from usual pain treatments for those without SUD histories.^53^ Additional research may determine why trends differed for different opioid prescription durations.

While the prevalence of MOUD was low among patients with OUD in the sample, treatment with MOUD had variable associations with pain severity scores in this study. Scores were lower among patients with OUD prescribed higher opioid doses and treated with MOUD, but patients receiving MOUD among all other prescribing groups had higher predicted mean pain severity than those not treated with MOUD. Previous research suggests that transition from full opioid agonists to buprenorphine/naloxone is associated with reduced pain among patients with OUD.^22,76,78^ Such improvements in pain are thought to be due in part to buprenorphine/naloxone seemingly having antihyperalgesic effects.^76^ Our findings add to this area of research by examining the relationship between pain severity in patients with OUD receiving MOUD while considering varied opioid prescription doses and durations, which has seemingly not yet been studied. Additional research seeking to explain observed differences in mean pain severity with MOUD treatment for patients receiving high-dose opioids compared to low-dose opioids may be of interest.

Use of CM and OS was associated with lower predicted mean pain severity scores for all opioid prescription doses and durations in patients with OUD, other SUD, and controls. Other studies have found multidisciplinary approaches to pain treatment, such as physical therapy and mindfulness classes, may improve functional and psychosocial outcomes, even among patients using opioid treatment.^37,55,73^ Since pain depends on many biological, psychological, and social factors,^52,63^ treatment approaches that address multiple dimensions of pain may contribute to improvements in patient pain perceptions. For example, treatment of emotional aspects of pain using psychotherapeutic approaches such as cognitive behavioral therapy has also been found to be effective.^35^ While MOUD and CM/OS appeared to have beneficial effects on pain, it may be that healthier and more well-resourced patients are more likely to use these services, but assessing such access facilitators is outside of the scope of EHR data. Nevertheless, our results suggest that such multidisciplinary approaches to pain management may be useful for patients with OUD and other SUDs and may warrant future research rigorously testing such interventions.

### 4.1. Limitations and strengths

Patient-reported pain is subject to limitations that may affect our study, such as inconsistencies in (1) clinical administration (possibly causing misestimation of pain),^67^ (2) accuracy in identifying patients with clinically meaningful pain,^45^ and (3) reported scores within patients over brief time windows.^29,59,61^ Our study used qualitative focus group data to identify factors that may influence pain severity scores among patients with SUDs, but such findings are not generalizable and cannot account for the full scope of factors influencing pain measurement. Yet, with no objective method to measure pain severity, subjective patient reports of pain play a role in clinical decision-making^60^ and determining optimal treatment.^63^

Our quantitative analysis may be limited by analyzing medication prescriptions rather than medication fills, and being unable to consider the appropriateness and context of prescriptions. We examined a unidimensional numeric pain severity scale instead of a multidimensional measure that includes pain interference or functionality. Electronic health record codes used in determining SUD status may be unreliable and cannot indicate SUD severity. Medications for opioid use disorder inclusion of methadone was limited to use of procedure codes for OUD treatment with methadone, as methadone prescriptions in hospitals for pain vs OUD treatment could not be differentiated and EHR data sources did not include outpatient programs licensed to dispense methadone for OUD. Additional factors potentially influencing pain reporting, such as patient preferences and cultural factors,^1,42^ are not captured in EHR data, and it is unknown how these may intersect with SUDs to influence the results of this study. Electronic health record data were also limited to OERWD-affiliated hospitals. However, utilizing OERWD enabled analysis of a large, diverse patient population, including the ability to study rare SUD groups.

## 5. Conclusion

High predicted average pain severity in individuals with OUD and other SUDs compared to controls highlights the need to better understand pain among patients with addiction diagnoses and identify effective treatments for their co-occurring conditions. This study suggests the need for future research on pain management in this population and the potential for incorporation of MOUD, CM, and OS interventions. However, with low percentages of patients with OUD receiving MOUD and patients with SUD using OS and CM, our results also highlight underutilization of such interventions that may have dual benefits for addiction and pain. Past research underscores the need for effective pain management among people with OUD due to pain potentially exacerbating risk for return to use.^24^ Pain management is rarely addressed in addiction treatment contexts,^24^ but synergistic interventions combining evidence-based SUD practices with multimodal patient-centered pain management interventions in SUD treatment contexts may enable pain severity reduction in this marginalized population.

## Disclosures

The authors have no conflict of interest to declare.

## Appendix A. Supplemental digital content

Supplemental digital content associated with this article can be found online at http://links.lww.com/PR9/A294.
