# Supplementary material for: Opioid prescription patterns and pain severity among patients with opioid use disorder and other substance use disorders: a mixed methods study
Source: Pain Rep. 2025 Apr 18;10(3):e1261. doi: 10.1097/PR9.0000000000001261 (PMC12026388; doi:10.1097/PR9.0000000000001261)
Supplement: Supplementary file 1 [file painreports-10-e1261-s001.pdf]

Supplement

Supplemental Table 1: List of codes used to define substance use disorder by substance class

Substance Class	Code Type	Code(s)
Alcohol	ICD-9	291.X, 303.X, 305.00, 305.01, 305.02, 305.03, 357.5, 425.5, 535.30, 535.31, 571.0, 571.1, 571.2, 571.3, 760.71, E860.0, 980.X, E860.0
	ICD-9 Procedure	94.46, 94.53, 94.61, 94.62, 94.63, 94.67, 94.68, 94.69
	SNOMED	235875008, 1082611000119101, 307757001, 191802004, 191806001, 9953008, 713370005, 183486001, 15167005, 73097000, 66590003, 213687005, 212820004, 212819005, 212812001, 212812001, 212808007, 212807002, 212806006, 67426006, 82782008, 212809004, 10741871000119101, 7052005, 25702006, 18653004, 8635005, 191476005, 191480000, 34938008, 300992002, 235952002, 53936005, 29212009, 237738005, 42344001, 78524005, 41083005, 70701004, 192811002, 235875008, 191478006, 7200002, 191475009, 307757001, 713181003, 154221000119101, 154211000119108, 191804003, 191811004, 133301000119102, 281004, 191805002, 21000000, 445507008, 135311000119100, 55571001, 85561006, 713862009, 228281002, 284591009, 191477001, 191883007, 191882002, 268645007, 714829008, 713583005, 191471000
	ICD-10	F10.X, G62.1, I42.6, K29.20, K29.21, K70.0, K70.10, K70.11, K70.2, K70.3, K70.30, K7.31, K70.40, K70.41, K70.9, T51.X, Z71.41
Tobacco	ICD-9	305.1, 649.0X, 989.84
	SNOMED	230064005, 160606002, 191888003, 191887008, 89765005, 449868002, 56294008, 56578002, 230062009, 160604004, 428071000124103, 56771006, 230063004, 455431000124101, 455421000124104, 455441000124106, 65568007, 191888003, 191887008, 212899006, 111779009, 291284006, 291285007, 291286008, 66884007
	CPT	99406, 99407
	HCPCS	G9458
	ICD-10	F17.X, O99.33X, T65.2X, T65.211A, T65.212A, T65.213A, T65.214A, T65.221A, T65.222A, T65.223A, T65.224A, T65.291A, T65.292A, T65.293A, T65.294A, Z72.0
Cannabis	ICD-9	304.3X, 305.2X
	SNOMED	15233006, 216551003, 16292008, 291246000, 291247009, 291248004, 212666007, 1149328002, 85005007, 191838006, 191837001, 191839003, 11048011000119103
	ICD-10	F12.X, T40.7X
Opioids	ICD-9	304.0X, 304.7X, 305.5X, 965.0, 965.00, 965.01, 965.02, 965.09, E850.0, E850.1, E850.2, E935.0, E935.1, E935.2
	SNOMED	295213004, 297199006, 242253008, 295165009, 461001000124107, 288861000119108, 1081000119105, 724653003, 432353006, 426001001, 295195001, 295194002, 295193008, 295193008, 295186009, 295185008, 295184007, 295176008, 295175007, 295174006, 295172005, 295171003, 295170002, 295161000, 295148000, 292064003, 292063009, 292060007, 292059002, 292055008, 292052006, 290204003, 290203009, 290202004, 290201006, 290193005, 290183003, 290182008, 290181001, 290179003, 290172007, 290171000, 290170004, 290157008, 269264002, 242829007, 241749009, 231480002, 231479000, 231478008, 231477003, 222059008, 216470005,

		216469009, 216468001, 216466002, 216465003, 216464004, 216463005, 213660006, 213659001, 213658009, 212676005, 212591003, 212588003, 212587008, 191914006, 191913000, 191912005, 191909007, 191820008, 191819002, 191817000, 75544000, 74264003, 68099003, 60199004, 18052008, 13187008, 11196001, 5602001, 191868002, 191867007, 191869005, 191865004
	ICD-10	F11.X, R78.1, T40.0X, T40.1X, T40.2X, T40.3X, T40.4X, T40.6X, Z79.891
Sedatives	ICD-9	304.1X, 305.4X, E950.1, 967.0, E980.1
	SNOMED	216497003, 216504000, 81914009, 216530001, 216537003, 212662009, 427327003, 64386003, 125851000119106
	ICD-10	F13.X, T42.3X, T42.4X
Stimulants	ICD-9	304.2X, 304.4X, 305.6X, 305.7
	SNOMED	216583009, 241761001, 291261004, 291263001, 291264007, 9982009, 290543000, 290544006, 290545007, 241760000, 216558009, 216561005, 442406005, 441527004
	ICD-10	F14.X, F15.X, T40.5X, T43.6X
Hallucinogens	ICD-9	304.5X, 305.3X, 969.6, E854.1
	SNOMED	67893003, 216552005, 216550002, 213661005, 221809003, 221810008, 221811007, 221812000, 221813005, 221814004, 221815003, 221816002,221817006, 221818001, 221819009, 222070001, 222071002, 222072009, 222073004, 222074005, 222075006, 222076007, 222077003, 222078008, 222079000, 222080002, 222668009, 222669001, 222670000, 222671001, 222672008, 222673003,222674009, 222675005, 222676006, 222677002, 222678007, 38247002, 191850000, 191849000, 191851001, 74851005
	ICD-10	F16.X, T40.8X, T40.9X
Inhalants	SNOMED	213705007, 212900001, 216662006, 216663001, 216666009, 157833001, 5002000, 86401000119104, 86391000119101, 70340006, 427229002, 426095000
	ICD-10	F18.X, T65.6X
SUD	ICD-9	292.X, 304.6X, 304.8X, 304.9X, 305.9X, V654.2
	ICD-9 Procedure	94.45, 94.54, 94.64, 94.65, 94.66, 94.67, 94.68, 94.69
	SNOMED	11387009, 396344000, 28368009, 39003006, 50026000, 83168008, 11061003, 2403008, 91388009, 724730008
	HCPCS	H0005, H0006, H0007, H0008, H0009, H0010, H0011, H0012, H0013, H0014, H0015, H0016, H0050, H0047, H2034, T1006
	ICD-10	F19.X
	ICD-10 Procedure	HZ2ZZZZ,<br>HZ30ZZZ, HZ31ZZZ, HZ32ZZZ, HZ33ZZZ, HZ34ZZZ, HZ35ZZZ, HZ36ZZZ, HZ37ZZZ, HZ38ZZZ, HZ39ZZZ, HZ3BZZZ, HZ3CZZZ<br>HZ40ZZZ, HZ41ZZZ, HZ42ZZZ, HZ43ZZZ, HZ44ZZZ, HZ45ZZZ, HZ46ZZZ, HZ47ZZZ, HZ48ZZZ, HZ49ZZZ, HZ4BZZZ, HZ4CZZZ,<br>HZ50ZZZ, HZ51ZZZ, HZ52ZZZ, HZ53ZZZ, HZ54ZZZ, HZ55ZZZ, HZ56ZZZ, HZ57ZZZ, HZ58ZZZ, HZ59ZZZ, HZ5BZZZ, HZ5CZZZ, HZ5DZZZ,<br>HZ63ZZZ
Psychotropic	SNOMED	212653007, 219311001, 61438005, 699011008, 221786001, 212672007, 212673002, 213668004, 216544007, 216566000, 221787005, 221788000, 221789008, 221790004,

	221791000, 221792007, 221793002, 221794008, 221795009, 221796005, 222048004, 222049007, 222050007, 222051006, 222052004, 222053009, 222054003, 222055002, 222056001, 222057005, 222058000, 222646004, 222647008, 222648003, 222649006, 222650006, 222651005, 222652003, 222653008, 222654002, 222655001, 222656000, 82225006
ICD-10	T43.8X, T43.9X

Patients were classified as having OUD or other SUD (alcohol, tobacco, cannabis, sedatives, stimulants, hallucinogens, inhalants, psychotropic, other [general SUD]) if they had qualifying codes (first inpatient or emergency encounter with qualifying code or first of ≥2 encounters, of any encounter type, with qualifying codes)

Supplemental Table 2a: List of codes used to define opioid prescriptions

NDC	Proprietary Name	Generic Name
2101002	Codeine Sulfate	codeine
2106402	Dolophine	methadone
2107202	Dolophine	methadone
2163701	Morphine Sulfate	morphine
2168201	Dolophine	methadone
2254902	Morphine Sulfate	morphine
2255002	Morphine Sulfate	morphine
2255102	Morphine Sulfate	morphine
2255702	Codeine Phosphate	codeine
2255802	Codeine Phosphate	codeine
2260658	Opium	opium
4004401	Levo-Dromoran	levorphanol
4191006	Levo-Dromoran	levorphanol
8023501	Mepergan	meperidine-promethazine
8025801	Meperidine Hydrochloride	meperidine
8025901	Meperidine Hydrochloride	meperidine
8026102	Mepergan Fortis	meperidine-promethazine
8029501	Hydromorphone Hydrochloride	hydromorphone
8029601	Hydromorphone Hydrochloride	hydromorphone
8030803	Meperidine Hydrochloride	meperidine
8033002	Belladonna Alkaloids-Opium	belladonna-opium
8038703	Hydromorphone Hydrochloride	hydromorphone
8038801	Hydromorphone Hydrochloride	hydromorphone
8060102	Meperidine Hydrochloride	meperidine
8060202	Meperidine Hydrochloride	meperidine
8060502	Meperidine Hydrochloride	meperidine
8061302	Meperidine Hydrochloride	meperidine
8064901	Morphine Sulfate	morphine
8065301	Morphine Sulfate	morphine
8065503	Morphine Sulfate	morphine
8065601	Morphine Sulfate	morphine
8065701	Morphine Sulfate	morphine
8072801	Codeine Phosphate	codeine
8072850	Codeine Phosphate	codeine
8072901	Codeine Phosphate	codeine
15564420	Stadol	butorphanol
15564433	Stadol	butorphanol
15564515	Stadol	butorphanol
15564520	Stadol	butorphanol
15564533	Stadol	butorphanol
15564615	Stadol	butorphanol
15564620	Stadol	butorphanol
15564633	Stadol	butorphanol

15564820	Stadol	butorphanol
15564897	Stadol	butorphanol
24027202	Codeine Phosphate	codeine
24027402	Codeine Phosphate	codeine
24032402	Demerol HCl	meperidine
24032502	Demerol HCl	meperidine
24032602	Demerol HCl	meperidine
24032802	Demerol HCl	meperidine
24033206	Demerol HCl	meperidine
24033502	Demerol HCl	meperidine
24033504	Demerol HCl	meperidine
24033505	Demerol HCl	meperidine
24033704	Demerol HCl	meperidine
24036104	Demerol HCl	meperidine
24036204	Demerol HCl	meperidine
24037204	Demerol HCl	meperidine
24037304	Demerol HCl	meperidine
24037404	Demerol HCl	meperidine
24072602	Hydromorphone Hydrochloride	hydromorphone
24072702	Hydromorphone Hydrochloride	hydromorphone
24072802	Hydromorphone Hydrochloride	hydromorphone
24125702	Morphine Sulfate	morphine
24125802	Morphine Sulfate	morphine
24125803	Morphine Sulfate	morphine
24125902	Morphine Sulfate	morphine
24126102	Morphine Sulfate	morphine
24126202	Morphine Sulfate	morphine
24192614	Talwin Lactate	pentazocine
24193704	Talacen	acetaminophen-pentazoci
31625763	Phenaphen with Codeine	acetaminophen-codeine
34051310	MS Contin	morphine
34051325	MS Contin	morphine
34051410	MS Contin	morphine
34051412	MS Contin	morphine
34051425	MS Contin	morphine
34051510	MS Contin	morphine
34051525	MS Contin	morphine
34051545	MS Contin	morphine
34051550	MS Contin	morphine
34051610	MS Contin	morphine
34051625	MS Contin	morphine
34051690	MS Contin	morphine
34051710	MS Contin	morphine
34051725	MS Contin	morphine
34051810	MSIR	morphine

34051815	MSIR	morphine
34051910	MSIR	morphine
34051930	MSIR	morphine
34052102	MSIR	morphine
34052301	MSIR	morphine
34052302	MSIR	morphine
34102510	MSIR	morphine
34102515	MSIR	morphine
34102630	MSIR	morphine
44062302	Codeine Sulfate	codeine
44062602	Codeine Sulfate	codeine
44072302	Vicoprofen	hydrocodone-ibuprofen
44072303	Vicoprofen	hydrocodone-ibuprofen
44072341	Vicoprofen	hydrocodone-ibuprofen
44072702	Vicodin	acetaminophen-hydrocodo
44072741	Vicodin	acetaminophen-hydrocodo
44072802	Vicodin ES	acetaminophen-hydrocodo
44072841	Vicodin ES	acetaminophen-hydrocodo
44101101	Dilaudid	hydromorphone
44101201	Dilaudid	hydromorphone
44101209	Dilaudid	hydromorphone
44101401	Dilaudid	hydromorphone
44101706	Dilaudid-HP	hydromorphone
44101710	Dilaudid-HP	hydromorphone
44101725	Dilaudid-HP	hydromorphone
44102202	Dilaudid	hydromorphone
44102245	Dilaudid	hydromorphone
44102402	Dilaudid	hydromorphone
44102445	Dilaudid	hydromorphone
44102802	Dilaudid	hydromorphone
44104001	Dilaudid	hydromorphone
44105301	Dilaudid	hydromorphone
44106205	Dilaudid	hydromorphone
44108501	Dilaudid-5	hydromorphone
44191101	Dilaudid-HP	hydromorphone
45050816	Tylenol with Codeine	acetaminophen-codeine
45051160	Tylenol with Codeine #2	acetaminophen-codeine
45051172	Tylenol with Codeine #2	acetaminophen-codeine
45051360	Tylenol with Codeine #3	acetaminophen-codeine
45051370	Tylenol with Codeine #3	acetaminophen-codeine
45051372	Tylenol with Codeine #3	acetaminophen-codeine
45051380	Tylenol with Codeine #3	acetaminophen-codeine
45051560	Tylenol with Codeine #4	acetaminophen-codeine
45051570	Tylenol with Codeine #4	acetaminophen-codeine
45051572	Tylenol with Codeine #4	acetaminophen-codeine

45052660	Tylox	acetaminophen-oxycodone
45052679	Tylox	acetaminophen-oxycodone
45065010	Ultracet	acetaminophen-tramadol
45065060	Ultracet	acetaminophen-tramadol
45065910	Ultram	tramadol
45065960	Ultram	tramadol
47044830	Acetaminophen-Hydrocodone B	acetaminophen-hydrocodo
54023524	Morphine Sulfate	morphine
54023525	Morphine Sulfate	morphine
54023624	Morphine Sulfate	morphine
54023625	Morphine Sulfate	morphine
54023749	Morphine Sulfate	morphine
54023755	Morphine Sulfate	morphine
54023763	Morphine Sulfate	morphine
54023849	Morphine Sulfate	morphine
54023863	Morphine Sulfate	morphine
54024324	Codeine Sulfate	codeine
54024424	Codeine Sulfate	codeine
54024425	Codeine Sulfate	codeine
54024525	Codeine Sulfate	codeine
54026424	Hydromorphone Hydrochloride	hydromorphone
54026425	Hydromorphone Hydrochloride	hydromorphone
54026525	Hydromorphone Hydrochloride	hydromorphone
54028325	Oxymorphone Hydrochloride	oxymorphone
54028425	Oxymorphone Hydrochloride	oxymorphone
54035244	Morphine Sulfate	morphine
54035250	Morphine Sulfate	morphine
54038663	HYDROmorphone Hydrochloride	HYDROmorphone
54039268	Methadone Hydrochloride	methadone
54040441	Morphine Sulfate	morphine
54040444	Morphine Sulfate	morphine
54040450	Morphine Sulfate	morphine
54051741	Morphine Sulfate	morphine
54051744	Morphine Sulfate	morphine
54051750	Morphine Sulfate	morphine
54052244	OxyCODONE Hydrochloride	oxyCODONE
54052363	OxyCODONE Hydrochloride	oxyCODONE
54055125	Acetaminophen-OxyCODONE Hyd	acetaminophen-oxyCODONE
54121811	Methadone Hydrochloride	methadone
54121842	Methadone Hydrochloride	methadone
54279525	Roxilox	acetaminophen-oxycodone
54300563	Acetaminophen-Codeine Phosp	acetaminophen-codeine
54309036	Butorphanol Tartrate	butorphanol
54316163	Codeine Phosphate	codeine
54338750	Hydromorphone Hydrochloride	hydromorphone

54338763	Hydromorphone Hydrochloride	hydromorphone
54354563	Meperidine Hydrochloride	meperidine
54355344	Methadone Hydrochloride	methadone
54355367	Methadone Hydrochloride	methadone
54355563	Methadone Hydrochloride	methadone
54355663	Methadone Hydrochloride	methadone
54368263	Roxicodone	oxycodone
54368344	Roxicodone	oxycodone
54368663	Roxicet	acetaminophen-oxycodone
54375144	Roxanol	morphine
54375150	Roxanol	morphine
54375158	Roxanol 100	morphine
54377444	Roxanol-T	morphine
54378549	Morphine Sulfate	morphine
54378563	Morphine Sulfate	morphine
54378663	Morphine Sulfate	morphine
54415625	Codeine Sulfate	codeine
54415725	Codeine Sulfate	codeine
54421625	Methadone Hydrochloride	methadone
54421725	Methadone Hydrochloride	methadone
54421825	Dolophine	methadone
54421925	Dolophine	methadone
54437025	Hydromorphone	hydromorphone
54439225	Hydromorphone Hydrochloride	hydromorphone
54439425	Hydromorphone Hydrochloride	hydromorphone
54449425	Levorphanol Tartrate	levorphanol
54453825	Methadone Hydrochloride	methadone
54457025	Methadone Hydrochloride	methadone
54457125	Methadone Hydrochloride	methadone
54458225	Morphine Sulfate	morphine
54458325	Morphine Sulfate	morphine
54459525	Meperidine Hydrochloride	meperidine
54465025	Roxicet	acetaminophen-oxycodone
54465029	Roxicet	acetaminophen-oxycodone
54465325	Roxiprin	aspirin-oxycodone
54465725	Roxicodone	oxycodone
54466525	Roxicodone	oxycodone
54479025	Oramorph SR	morphine
54479225	Oramorph SR	morphine
54479325	Oramorph SR	morphine
54480519	Oramorph SR	morphine
54480525	Oramorph SR	morphine
54800204	Acetaminophen-Codeine Phosp	acetaminophen-codeine
54801304	Acetaminophen-Codeine Phosp	acetaminophen-codeine
54801704	Acetaminophen-Codeine Phosp	acetaminophen-codeine

54802224	Acetaminophen-Codeine Phosp	acetaminophen-codeine
54815524	Codeine Sulfate	codeine
54815624	Codeine Sulfate	codeine
54815724	Codeine Sulfate	codeine
54816016	Codeine Phosphate	codeine
54839224	Hydromorphone Hydrochloride	hydromorphone
54839424	Hydromorphone Hydrochloride	hydromorphone
54849424	Levorphanol Tartrate	levorphanol
54854516	Meperidine Hydrochloride	meperidine
54855324	Methadone Hydrochloride	methadone
54855424	Methadone Hydrochloride	methadone
54858224	Morphine Sulfate	morphine
54858324	Morphine Sulfate	morphine
54858516	Morphine Sulfate	morphine
54858616	Morphine Sulfate	morphine
54859511	Meperidine Hydrochloride	meperidine
54859611	Meperidine Hydrochloride	meperidine
54864816	Roxicet	acetaminophen-oxycodone
54865024	Roxicet	acetaminophen-oxycodone
54865324	Roxiprin	aspirin-oxycodone
54865724	Roxicodone	oxycodone
54865824	Roxicodone	oxycodone
54866524	Roxicodone	oxycodone
54878216	Roxicodone	oxycodone
54878424	Roxicet	acetaminophen-oxycodone
54878511	Roxanol UD	morphine
54878811	Roxanol UD	morphine
54879024	Oramorph SR	morphine
54879211	Oramorph SR	morphine
54879311	Oramorph SR	morphine
54880524	Oramorph SR	morphine
62065330	Ultram ER	tramadol
62065530	Ultram ER	tramadol
62065730	Ultram ER	tramadol
74109702	Codeine Phosphate	codeine
74109732	Codeine Phosphate	codeine
74110202	Codeine Phosphate	codeine
74110232	Codeine Phosphate	codeine
74113303	Morphine Sulfate	morphine
74113403	Morphine Sulfate	morphine
74113405	Morphine Sulfate	morphine
74113422	Morphine Sulfate	morphine
74113501	Morphine Sulfate	morphine
74113502	Morphine Sulfate	morphine
74113503	Morphine Sulfate	morphine

74117601	Demerol HCl	meperidine
74117611	Demerol HCl	meperidine
74117621	Demerol HCl	meperidine
74117630	Demerol HCl	meperidine
74117631	Demerol HCl	meperidine
74117801	Demerol HCl	meperidine
74117811	Demerol HCl	meperidine
74117821	Demerol HCl	meperidine
74117830	Demerol HCl	meperidine
74117831	Demerol HCl	meperidine
74117901	Demerol HCl	meperidine
74117902	Demerol HCl	meperidine
74117911	Demerol HCl	meperidine
74117921	Demerol HCl	meperidine
74117930	Demerol HCl	meperidine
74117931	Demerol HCl	meperidine
74118001	Demerol HCl	meperidine
74118011	Demerol HCl	meperidine
74118031	Demerol HCl	meperidine
74118069	Demerol HCl	meperidine
74118130	Demerol HCl	meperidine
74120120	Demerol HCl	meperidine
74120301	Demerol HCl	meperidine
74125301	Demerol HCl	meperidine
74125401	Demerol HCl	meperidine
74125502	Demerol HCl	meperidine
74125601	Demerol HCl	meperidine
74125801	Morphine Sulfate	morphine
74125802	Morphine Sulfate	morphine
74125830	Morphine Sulfate	morphine
74125831	Morphine Sulfate	morphine
74126001	Morphine Sulfate	morphine
74126031	Morphine Sulfate	morphine
74126069	Morphine Sulfate	morphine
74126101	Morphine Sulfate	morphine
74126102	Morphine Sulfate	morphine
74126130	Morphine Sulfate	morphine
74126131	Morphine Sulfate	morphine
74126201	Morphine Sulfate	morphine
74126301	Morphine Sulfate	morphine
74126321	Morphine Sulfate	morphine
74126401	Morphine Sulfate	morphine
74126431	Morphine Sulfate	morphine
74126601	Demerol HCl	meperidine
74126701	Demerol HCl	meperidine

74127605	Fentanyl Citrate	fentanyl
74127632	Fentanyl Citrate	fentanyl
74127635	Fentanyl Citrate	fentanyl
74128301	Hydromorphone	hydromorphone
74128331	Hydromorphone Hydrochloride	hydromorphone
74130401	Hydromorphone Hydrochloride	hydromorphone
74130431	Hydromorphone Hydrochloride	hydromorphone
74131201	Hydromorphone Hydrochloride	hydromorphone
74131202	Hydromorphone Hydrochloride	hydromorphone
74131212	Hydromorphone Hydrochloride	hydromorphone
74131230	Hydromorphone Hydrochloride	hydromorphone
74131231	Hydromorphone Hydrochloride	hydromorphone
74146301	Nalbuphine Hydrochloride	nalbuphine
74146401	Nalbuphine Hydrochloride	nalbuphine
74146501	Nalbuphine Hydrochloride	nalbuphine
74162301	Butorphanol Tartrate	butorphanol
74162349	Butorphanol Tartrate	butorphanol
74162601	Butorphanol Tartrate	butorphanol
74162602	Butorphanol Tartrate	butorphanol
74162649	Butorphanol Tartrate	butorphanol
74176201	Morphine Sulfate	morphine
74176202	Morphine Sulfate	morphine
74176211	Morphine Sulfate	morphine
74176221	Morphine Sulfate	morphine
74176230	Morphine Sulfate	morphine
74176231	Morphine Sulfate	morphine
74193701	Talwin Lactate	pentazocine
74193802	Talwin Lactate	pentazocine
74194101	Talwin Lactate	pentazocine
74194111	Talwin Lactate	pentazocine
74194912	Vicodin	acetaminophen-hydrocodo
74194914	Vicodin	acetaminophen-hydrocodo
74197312	Vicodin ES	acetaminophen-hydrocodo
74197314	Vicodin ES	acetaminophen-hydrocodo
74202802	Morphine Sulfate	morphine
74202902	Morphine Sulfate	morphine
74227414	Vicodin HP	acetaminophen-hydrocodo
74227712	Vicoprofen	hydrocodone-ibuprofen
74227714	Vicoprofen	hydrocodone-ibuprofen
74233211	Dilaudid	hydromorphone
74233311	Dilaudid	hydromorphone
74233326	Dilaudid	hydromorphone
74233411	Dilaudid	hydromorphone
74241421	Dilaudid	hydromorphone
74241512	Dilaudid	hydromorphone

74241514	Dilaudid	hydromorphone
74241612	Dilaudid	hydromorphone
74241614	Dilaudid	hydromorphone
74242614	Dilaudid	hydromorphone
74244305	Fentanyl Citrate	fentanyl
74244405	Fentanyl Citrate	fentanyl
74244505	Fentanyl Citrate	fentanyl
74244605	Fentanyl Citrate	fentanyl
74245107	Dilaudid	hydromorphone
74245202	Dilaudid-5	hydromorphone
74245311	Dilaudid-HP	hydromorphone
74245327	Dilaudid-HP	hydromorphone
74245351	Dilaudid-HP	hydromorphone
74245531	Dilaudid-HP	hydromorphone
74246024	Actiq	fentanyl
74246124	Actiq	fentanyl
74246224	Actiq	fentanyl
74246324	Actiq	fentanyl
74246424	Actiq	fentanyl
74246524	Actiq	fentanyl
74304113	Vicodin	acetaminophen-HYDROcodo
74304313	Vicodin ES	acetaminophen-HYDROcodo
74381412	Morphine Sulfate	morphine
74381512	Morphine Sulfate	morphine
74381912	Morphine Sulfate	morphine
74405712	Morphine Sulfate	morphine
74405812	Morphine Sulfate	morphine
74602304	Morphine Sulfate	morphine
74602804	Morphine Sulfate	morphine
74603004	Meperidine Hydrochloride	meperidine
74606202	Morphine Sulfate	morphine
74606203	Morphine Sulfate	morphine
74606211	Morphine Sulfate	morphine
74606302	Morphine Sulfate	morphine
74617714	Morphine Sulfate	morphine
74617914	Morphine Sulfate	morphine
74909332	Fentanyl Citrate	fentanyl
74909335	Fentanyl Citrate	fentanyl
74909336	Fentanyl Citrate	fentanyl
74909338	Fentanyl Citrate	fentanyl
74909422	Fentanyl Citrate	fentanyl
74909425	Fentanyl Citrate	fentanyl
74909428	Fentanyl Citrate	fentanyl
74909431	Fentanyl Citrate	fentanyl
74909461	Fentanyl Citrate	fentanyl

78010705	Fiorinal with Codeine	ASA/butalbital/caffeine
78010713	Fiorinal with Codeine	ASA/butalbital/caffeine
78024305	Fioricet with Codeine	APAP/butalbital/caffein
86004616	Capital with Codeine Suspen	acetaminophen-codeine
87565041	Stadol NS	butorphanol
93002401	Oxycodone Hydrochloride ER	oxycodone
93003101	Oxycodone Hydrochloride ER	oxycodone
93003201	Oxycodone Hydrochloride ER	oxycodone
93005001	Acetaminophen-Codeine Phosp	acetaminophen-codeine
93005801	Tramadol	tramadol
93005805	Tramadol	tramadol
93015001	Acetaminophen-Codeine Phosp	acetaminophen-codeine
93015010	Acetaminophen-Codeine Phosp	acetaminophen-codeine
93015093	Acetaminophen-Codeine Phosp	acetaminophen-codeine
93035001	Acetaminophen-Codeine Phosp	acetaminophen-codeine
93516101	Hydrocodone-Ibuprofen	hydrocodone-ibuprofen
93537165	Fentanyl Citrate	fentanyl
93573101	OxyCODONE Hydrochloride ER	oxyCODONE
93573201	OxyCODONE Hydrochloride ER	oxyCODONE
93586101	Oxymorphone Hydrochloride	oxymorphone
93690045	Fentanyl	fentanyl
93786865	FentaNYL Citrate	fentaNYL
95909016	Lortab Elixir	acetaminophen-HYDROcodo
115123113	Oxymorphone Hydrochloride E	oxymorphone
115123201	Oxymorphone Hydrochloride E	oxymorphone
115123213	Oxymorphone Hydrochloride E	oxymorphone
115123301	Oxymorphone Hydrochloride E	oxymorphone
115123401	Oxymorphone Hydrochloride E	oxymorphone
115131613	Oxymorphone Hydrochloride E	oxymorphone
121050404	Acetaminophen-Codeine Phosp	acetaminophen-codeine
121050405	Acetaminophen-Codeine Phosp	acetaminophen-codeine
121050410	Acetaminophen-Codeine Phosp	acetaminophen-codeine
121050412	Acetaminophen-Codeine Phosp	acetaminophen-codeine
121050415	Acetaminophen-Codeine Phosp	acetaminophen-codeine
121050416	Acetaminophen-Codeine Phosp	acetaminophen-codeine
121065504	Acetaminophen-Hydrocodone B	acetaminophen-hydrocodo
121065515	Acetaminophen-Hydrocodone B	acetaminophen-hydrocodo
121065516	Acetaminophen-Hydrocodone B	acetaminophen-hydrocodo
121077204	Acetaminophen-HYDROcodone B	acetaminophen-HYDROcodo
121077216	Acetaminophen-HYDROcodone B	acetaminophen-HYDROcodo
121465505	Acetaminophen-Hydrocodone B	acetaminophen-hydrocodo
121465510	Acetaminophen-Hydrocodone B	acetaminophen-hydrocodo
121465515	Acetaminophen-Hydrocodone B	acetaminophen-hydrocodo
121477107	Acetaminophen-Hydrocodone B	acetaminophen-hydrocodo
121477115	Acetaminophen-Hydrocodone B	acetaminophen-hydrocodo

121477205	Acetaminophen-HYDROcodone B	acetaminophen-HYDROcodo
121477210	Acetaminophen-HYDROcodone B	acetaminophen-HYDROcodo
121477215	Acetaminophen-HYDROcodone B	acetaminophen-HYDROcodo
121483905	OxyCODONE Hydrochloride	oxyCODONE
121483940	OxyCODONE Hydrochloride	oxyCODONE
143300001	APAP/Butalbital/Caffeine/Co	APAP/butalbital/caffein
143986410	Butorphanol Tartrate	butorphanol
143986710	Butorphanol Tartrate	butorphanol
172635460	Oxycodone Hydrochloride ER	oxycodone
172635560	Oxycodone Hydrochloride ER	oxycodone
172635660	Oxycodone Hydrochloride ER	oxycodone
172635910	Acetaminophen-Tramadol Hydr	acetaminophen-tramadol
172635960	Acetaminophen-Tramadol Hydr	acetaminophen-tramadol
172651510	Tramadol Hydrochloride	tramadol
182003601	Aspirin-Butalbital-Caffeine	ASA/butalbital/caffeine
182094889	Acetaminophen-Codeine Phosp	acetaminophen-codeine
182107837	Acetaminophen-Codeine Phosp	acetaminophen-codeine
182122501	Aspirin-Codeine Phosphate	aspirin-codeine
182914001	Meperidine Hydrochloride	meperidine
185031101	Tramadol	tramadol
185074901	ASA/Carisoprodol/Codeine Ph	ASA/carisoprodol/codein
186112081	Morphine Sulfate	morphine
186112495	Morphine Sulfate	morphine
186113913	Morphine Sulfate	morphine
186115002	Astramorph PF	morphine
186115102	Astramorph PF	morphine
186115212	Astramorph PF	morphine
186115312	Astramorph PF	morphine
186115802	Morphine Sulfate	morphine
186115903	Astramorph PF	morphine
186116003	Astramorph PF	morphine
186123213	Droperidol-Fentanyl	droperidol-fentanyl
186128301	Meperidine Hydrochloride	meperidine
186128401	Meperidine Hydrochloride	meperidine
186130901	Hydromorphone Hydrochloride	hydromorphone
187000301	Capital with Codeine Suspen	acetaminophen-codeine
187307210	Levo-Dromoran	levorphanol
209423712	Hydromorphone	hydromorphone
209685622	Morphine Sulfate	morphine
209686422	Morphine Sulfate	morphine
209686522	Morphine Sulfate	morphine
209686720	Morphine Sulfate	morphine
228271450	Tramadol	tramadol
228287811	OxyCODONE Hydrochloride	oxyCODONE
228298211	Acetaminophen-OxyCODONE Hyd	acetaminophen-oxyCODONE

228298311	Acetaminophen-OxyCODONE Hyd	acetaminophen-oxyCODONE
228309011	Morphine Sulfate ER	morphine
228322711	Oxymorphone Hydrochloride E	oxymorphone
228322811	Oxymorphone Hydrochloride E	oxymorphone
228322911	Oxymorphone Hydrochloride E	oxymorphone
228323011	Oxymorphone Hydrochloride E	oxymorphone
228326111	Oxymorphone Hydrochloride E	oxymorphone
228350111	Morphine Sulfate ER	morphine
228350211	Morphine Sulfate ER	morphine
228350306	Morphine Sulfate ER	morphine
228350311	Morphine Sulfate ER	morphine
228350411	Morphine Sulfate ER	morphine
228350711	Morphine Sulfate ER	morphine
228402911	Ibuprofen-Oxycodone Hydroch	ibuprofen-oxycodone
245016012	RMS	morphine
245016112	RMS	morphine
245016212	RMS	morphine
245016312	RMS	morphine
245042005	FentaNYL	fentaNYL
245042089	FentaNYL	fentaNYL
245042105	FentaNYL	fentaNYL
245042189	FentaNYL	fentaNYL
247007812	Acetaminophen-Codeine Phosp	acetaminophen-codeine
247007900	Acetaminophen-Hydrocodone B	acetaminophen-hydrocodo
247007906	Acetaminophen-Hydrocodone B	acetaminophen-hydrocodo
247009916	Acetaminophen-Codeine Phosp	acetaminophen-codeine
247023410	Acetaminophen-Codeine Phosp	acetaminophen-codeine
247023412	Acetaminophen-Codeine Phosp	acetaminophen-codeine
247034200	Acetaminophen-Hydrocodone B	acetaminophen-hydrocodo
254359735	Acetaminophen-Hydrocodone B	acetaminophen-hydrocodo
338268975	Morphine Sulfate	morphine
338269075	Morphine Sulfate	morphine
338269175	Meperidine Hydrochloride	meperidine
338931148	Fentanyl Citrate	fentanyl
338931402	Fentanyl Citrate	fentanyl
338932248	Bupivacaine-Fentanyl	bupivacaine-fentanyl
338932448	Bupivacaine-Fentanyl	bupivacaine-fentanyl
338932702	Bupivacaine-Fentanyl	bupivacaine-fentanyl
338933548	Bupivacaine-Fentanyl	bupivacaine-fentanyl
338933602	Bupivacaine-Fentanyl	bupivacaine-fentanyl
338934002	Bupivacaine-Fentanyl	bupivacaine-fentanyl
338936141	Hydromorphone Hydrochloride	hydromorphone
338936297	Hydromorphone Hydrochloride	hydromorphone
338936375	Hydromorphone Hydrochloride	hydromorphone
338936541	Hydromorphone Hydrochloride	hydromorphone

338936775	Hydromorphone Hydrochloride	hydromorphone
338937548	Morphine Sulfate	morphine
338937948	Morphine Sulfate	morphine
338938348	Morphine Sulfate	morphine
338938775	Morphine Sulfate	morphine
338940702	Bupivacaine-Fentanyl	bupivacaine-fentanyl
338942448	Hydromorphone Hydrochloride	hydromorphone
364052601	Acetaminophen-Codeine Phosp	acetaminophen-codeine
364245055	Morphine Sulfate	morphine
364301554	Morphine Sulfate	morphine
364301628	Morphine Sulfate	morphine
364301654	Morphine Sulfate	morphine
364306755	Morphine Sulfate	morphine
378265801	Morphine Sulfate ER	morphine
378265901	Morphine Sulfate ER	morphine
378266001	Morphine Sulfate ER	morphine
378266101	Morphine Sulfate ER	morphine
378415101	Tramadol Hydrochloride	tramadol
378415105	Tramadol Hydrochloride	tramadol
378415293	TraMADol Hydrochloride ER	traMADol
378611201	OxyCODONE Hydrochloride	oxyCODONE
378611301	OxyCODONE Hydrochloride	oxyCODONE
378611401	OxyCODONE Hydrochloride	oxyCODONE
378611701	Aspirin-OxyCODONE	aspirin-oxyCODONE
378710301	Acetaminophen-Oxycodone Hyd	acetaminophen-oxycodone
378710401	Acetaminophen-Oxycodone Hyd	acetaminophen-oxycodone
378710501	Acetaminophen-Oxycodone Hyd	acetaminophen-oxycodone
378710601	Acetaminophen-Oxycodone Hyd	acetaminophen-oxycodone
378808801	Acetaminophen-TraMADOL Hydr	acetaminophen-tramadol
378911916	Fentanyl	fentanyl
378911998	Fentanyl	fentanyl
378912116	Fentanyl	fentanyl
378912198	Fentanyl	fentanyl
378912216	Fentanyl	fentanyl
378912298	Fentanyl	fentanyl
378912316	Fentanyl	fentanyl
378912398	Fentanyl	fentanyl
378912498	Fentanyl	fentanyl
378963943	Butorphanol Tartrate	butorphanol
406012301	Acetaminophen-HYDROcodone B	acetaminophen-HYDROcodo
406012305	Acetaminophen-HYDROcodone B	acetaminophen-HYDROcodo
406012362	Acetaminophen-HYDROcodone B	acetaminophen-HYDROcodo
406012401	Acetaminophen-HYDROcodone B	acetaminophen-HYDROcodo
406012462	Acetaminophen-HYDROcodone B	acetaminophen-HYDROcodo
406012501	Acetaminophen-HYDROcodone B	acetaminophen-HYDROcodo

406012562	Acetaminophen-HYDROcodone B	acetaminophen-HYDROcodo
406035701	Acetaminophen-Hydrocodone B	acetaminophen-hydrocodo
406035705	Acetaminophen-Hydrocodone B	acetaminophen-hydrocodo
406035762	Acetaminophen-Hydrocodone B	acetaminophen-hydrocodo
406035763	Acetaminophen-Hydrocodone B	acetaminophen-hydrocodo
406035801	Acetaminophen-Hydrocodone B	acetaminophen-hydrocodo
406035805	Acetaminophen-Hydrocodone B	acetaminophen-hydrocodo
406035862	Acetaminophen-Hydrocodone B	acetaminophen-hydrocodo
406035901	Acetaminophen-Hydrocodone B	acetaminophen-hydrocodo
406036001	Acetaminophen-Hydrocodone B	acetaminophen-hydrocodo
406036005	Acetaminophen-Hydrocodone B	acetaminophen-hydrocodo
406036101	Acetaminophen-Hydrocodone B	acetaminophen-hydrocodo
406036105	Acetaminophen-Hydrocodone B	acetaminophen-hydrocodo
406036162	Acetaminophen-Hydrocodone B	acetaminophen-hydrocodo
406036201	Acetaminophen-Hydrocodone B	acetaminophen-hydrocodo
406036301	Acetaminophen-Hydrocodone B	acetaminophen-hydrocodo
406036305	Acetaminophen-Hydrocodone B	acetaminophen-hydrocodo
406036362	Acetaminophen-Hydrocodone B	acetaminophen-hydrocodo
406036501	Acetaminophen-Hydrocodone B	acetaminophen-hydrocodo
406036505	Acetaminophen-Hydrocodone B	acetaminophen-hydrocodo
406036562	Acetaminophen-Hydrocodone B	acetaminophen-hydrocodo
406036601	Acetaminophen-Hydrocodone B	acetaminophen-hydrocodo
406036605	Acetaminophen-Hydrocodone B	acetaminophen-hydrocodo
406036662	Acetaminophen-Hydrocodone B	acetaminophen-hydrocodo
406036701	Acetaminophen-Hydrocodone B	acetaminophen-hydrocodo
406036705	Acetaminophen-Hydrocodone B	acetaminophen-hydrocodo
406036762	Acetaminophen-Hydrocodone B	acetaminophen-hydrocodo
406037516	Acetaminophen-Hydrocodone B	acetaminophen-hydrocodo
406048301	Acetaminophen-Codeine Phosp	acetaminophen-codeine
406048401	Acetaminophen-Codeine Phosp	acetaminophen-codeine
406048410	Acetaminophen-Codeine Phosp	acetaminophen-codeine
406048462	Acetaminophen-Codeine Phosp	acetaminophen-codeine
406048501	Acetaminophen-Codeine Phosp	acetaminophen-codeine
406051201	Acetaminophen-Oxycodone Hyd	acetaminophen-oxycodone
406051205	Acetaminophen-Oxycodone Hyd	acetaminophen-oxycodone
406051262	Acetaminophen-Oxycodone Hyd	acetaminophen-oxycodone
406052201	Acetaminophen-Oxycodone Hyd	acetaminophen-oxycodone
406052262	Acetaminophen-Oxycodone Hyd	acetaminophen-oxycodone
406052301	Acetaminophen-Oxycodone Hyd	acetaminophen-oxycodone
406052362	Acetaminophen-Oxycodone Hyd	acetaminophen-oxycodone
406052710	Methadose	methadone
406053201	Acetaminophen-Oxycodone Hyd	acetaminophen-oxycodone
406054034	Methadose	methadone
406055201	M-Oxy	oxycodone
406055262	Oxycodone Hydrochloride	oxycodone

406055401	Oxycodone Hydrochloride	oxycodone
406058201	Acetaminophen-Oxycodone Hyd	acetaminophen-oxycodone
406059401	Oxycodone Hydrochloride ER	oxycodone
406059501	Oxycodone Hydrochloride ER	oxycodone
406059601	Oxycodone Hydrochloride ER	oxycodone
406083012	Morphine Sulfate	morphine
406083024	Morphine Sulfate	morphine
406083030	Morphine Sulfate	morphine
406113052	Fentanyl Citrate	fentanyl
406152153	Morphine Sulfate	morphine
406152155	Morphine Sulfate	morphine
406324301	Hydromorphone Hydrochloride	hydromorphone
406324401	Hydromorphone	hydromorphone
406324552	Hydromorphone Hydrochloride	hydromorphone
406324901	Hydromorphone Hydrochloride	hydromorphone
406330801	HYDROmorphone Hydrochloride	HYDROmorphone
406345434	Methadose	methadone
406536201	Anexsia	acetaminophen-hydrocodo
406575501	Methadone Hydrochloride	methadone
406575562	Methadone Hydrochloride	methadone
406577101	Methadone Hydrochloride	methadone
406577162	Methadone Hydrochloride	methadone
406697434	Methadose	methadone
406711301	Meperidine Hydrochloride	meperidine
406717101	Tramadol	tramadol
406717162	Tramadol Hydrochloride	tramadol
406800312	Morphine Sulfate	morphine
406800315	Morphine Sulfate	morphine
406800324	Morphine Sulfate	morphine
406800330	Morphine Sulfate	morphine
406831501	Morphine Sulfate SR	morphine
406831562	Morphine Sulfate SR	morphine
406832001	Morphine Sulfate SR	morphine
406833001	Morphine Sulfate SR	morphine
406833005	Morphine Sulfate SR	morphine
406833062	Morphine Sulfate SR	morphine
406838001	Morphine Sulfate SR	morphine
406838005	Morphine Sulfate SR	morphine
406838062	Morphine Sulfate SR	morphine
406839001	Morphine Sulfate SR	morphine
406839062	Morphine Sulfate SR	morphine
406851501	Oxycodone Hydrochloride	oxycodone
406851562	OxyCODONE Hydrochloride	oxyCODONE
406853001	Oxycodone Hydrochloride	oxycodone
406853062	OxyCODONE Hydrochloride	oxyCODONE

406855550	Oxycodone Hydrochloride	oxycodone
406855830	Oxycodone Hydrochloride	oxycodone
406866830	Oxycodone Hydrochloride	oxycodone
406872510	Methadose	methadone
406900076	Fentanyl	fentanyl
406901276	FentaNYL	fentaNYL
406902576	Fentanyl	fentanyl
406905076	Fentanyl	fentanyl
406907576	Fentanyl	fentanyl
406920230	Fentanyl Citrate	fentanyl
406920430	Fentanyl Citrate	fentanyl
406920630	Fentanyl Citrate	fentanyl
406920830	Fentanyl Citrate	fentanyl
406921230	Fentanyl Citrate	fentanyl
409109732	Codeine Phosphate	codeine
409110232	Codeine Phosphate	codeine
409113403	Morphine Sulfate	morphine
409113405	Morphine Sulfate	morphine
409113502	Morphine Sulfate	morphine
409117630	Demerol HCl	meperidine
409117830	Demerol HCl	meperidine
409117930	Demerol HCl	meperidine
409118069	Demerol HCl	meperidine
409118130	Demerol HCl	meperidine
409120120	Demerol HCl	meperidine
409120301	Demerol HCl	meperidine
409125301	Demerol HCl	meperidine
409125401	Demerol HCl	meperidine
409125502	Demerol HCl	meperidine
409125601	Demerol HCl	meperidine
409125830	Morphine Sulfate	morphine
409126069	Morphine Sulfate	morphine
409126130	Morphine Sulfate	morphine
409126431	Morphine Sulfate	morphine
409127632	Fentanyl Citrate	fentanyl
409128304	HYDROmorphone Hydrochloride	HYDROmorphone
409128305	HYDROmorphone Hydrochloride	HYDROmorphone
409128310	Hydromorphone Hydrochloride	hydromorphone
409128331	Hydromorphone Hydrochloride	hydromorphone
409130431	Hydromorphone Hydrochloride	hydromorphone
409131210	Hydromorphone Hydrochloride	hydromorphone
409131230	Hydromorphone Hydrochloride	hydromorphone
409146301	Nalbuphine Hydrochloride	nalbuphine
409146349	Nalbuphine Hydrochloride	nalbuphine
409146401	Nalbuphine Hydrochloride	nalbuphine

409146501	Nalbuphine Hydrochloride	nalbuphine
409146549	Nalbuphine Hydrochloride	nalbuphine
409146701	Nalbuphine Hydrochloride	nalbuphine
409162301	Butorphanol Tartrate	butorphanol
409162349	Butorphanol Tartrate Novapl	butorphanol
409162601	Butorphanol Tartrate	butorphanol
409162602	Butorphanol Tartrate	butorphanol
409162649	Butorphanol Tartrate Novapl	butorphanol
409162651	Butorphanol Tartrate Novapl	butorphanol
409176230	Morphine Sulfate	morphine
409189001	Morphine Sulfate	morphine
409189011	Morphine Sulfate	morphine
409189101	Morphine Sulfate	morphine
409189111	Morphine Sulfate	morphine
409189201	Morphine Sulfate	morphine
409189301	Morphine Sulfate	morphine
409189401	Morphine Sulfate	morphine
409192010	Talwin Lactate	pentazocine
409194101	Talwin Lactate	pentazocine
409202902	Morphine Sulfate	morphine
409217201	Hydromorphone Hydrochloride	hydromorphone
409217205	Hydromorphone Hydrochloride	hydromorphone
409254001	Hydromorphone Hydrochloride	hydromorphone
409255201	Hydromorphone Hydrochloride	hydromorphone
409263401	Hydromorphone Hydrochloride	hydromorphone
409263405	Hydromorphone Hydrochloride	hydromorphone
409263450	Hydromorphone Hydrochloride	hydromorphone
409335601	Hydromorphone Hydrochloride	hydromorphone
409336501	Hydromorphone Hydrochloride	hydromorphone
409381412	Morphine Sulfate	morphine
409381512	Morphine Sulfate	morphine
409405712	Morphine Sulfate	morphine
409405812	Morphine Sulfate	morphine
409602804	Morphine Sulfate	morphine
409603004	Meperidine Hydrochloride	meperidine
409606202	Morphine Sulfate	morphine
409606211	Morphine Sulfate	morphine
409617714	Morphine Sulfate	morphine
409617914	Morphine Sulfate	morphine
409909332	Fentanyl Citrate	fentanyl
409909335	Fentanyl Citrate	fentanyl
409909336	Fentanyl Citrate	fentanyl
409909338	Fentanyl Citrate	fentanyl
409909411	FentaNYL Citrate	fentaNYL
409909422	Fentanyl Citrate	fentanyl

409909425	Fentanyl Citrate	fentanyl
409909428	Fentanyl Citrate	fentanyl
409909431	Fentanyl Citrate	fentanyl
409909461	Fentanyl Citrate	fentanyl
472141904	Acetaminophen-Codeine Phosp	acetaminophen-codeine
472141916	Acetaminophen-Codeine Phosp	acetaminophen-codeine
525001601	Panlor DC	ASA/caffeine/dihydrocod
525003201	Panlor SS	APAP/caffeine/dihydroco
525003210	Panlor SS	APAP/caffeine/dihydroco
527131201	Aspirin-Butalbital-Caffeine	ASA/butalbital/caffeine
527135301	Hydromorphone Hydrochloride	hydromorphone
527135401	Hydromorphone Hydrochloride	hydromorphone
527135501	Hydromorphone Hydrochloride	hydromorphone
527142536	Morphine Sulfate	morphine
527142562	Morphine Sulfate	morphine
527142563	Morphine Sulfate	morphine
527142636	Oxycodone Hydrochloride	oxycodone
527169801	Codeine Sulfate	codeine
527169891	Codeine Sulfate	codeine
527169901	Codeine Sulfate	codeine
536391405	Acetaminophen-Hydrocodone B	acetaminophen-hydrocodo
548191100	Morphine Sulfate	morphine
548191125	Morphine Sulfate	morphine
548192700	Meperidine Hydrochloride	meperidine
548193100	Morphine Sulfate	morphine
548193110	Morphine Sulfate	morphine
548193310	Morphine Sulfate	morphine
548290100	Morphine Sulfate	morphine
548290125	Morphine Sulfate	morphine
548339110	Morphine Sulfate	morphine
548602000	Morphine Sulfate	morphine
548602100	Morphine Sulfate	morphine
548602400	Morphine Sulfate	morphine
548602500	Morphine Sulfate	morphine
548602600	Morphine Sulfate	morphine
548604025	Morphine Sulfate	morphine
548604125	Morphine Sulfate	morphine
548604225	Morphine Sulfate	morphine
548604325	Morphine Sulfate	morphine
548604525	Morphine Sulfate	morphine
548635500	Morphine Sulfate	morphine
555038102	Meperidine Hydrochloride	meperidine
555038202	Meperidine Hydrochloride	meperidine
555065802	Acetaminophen-Oxycodone Hyd	acetaminophen-oxycodone
555108001	Fentanyl Citrate	fentanyl

555108101	Fentanyl Citrate	fentanyl
555108201	Fentanyl Citrate	fentanyl
555108301	Fentanyl Citrate	fentanyl
555108401	Fentanyl Citrate	fentanyl
555111705	Hydromorphone Hydrochloride	hydromorphone
555111706	Hydromorphone Hydrochloride	hydromorphone
555111707	Hydromorphone Hydrochloride	hydromorphone
555111909	Nalbuphine Hydrochloride	nalbuphine
555112105	Nalbuphine Hydrochloride	nalbuphine
555112205	Nalbuphine Hydrochloride	nalbuphine
574015312	Morphine Sulfate	morphine
574015330	Morphine Sulfate	morphine
574200605	Morphine Sulfate	morphine
574200625	Morphine Sulfate	morphine
574201701	Hydromorphone Hydrochloride	hydromorphone
574704012	Belladonna Alkaloids-Opium	belladonna-opium
574704020	Belladonna Alkaloids-Opium	belladonna-opium
574704512	Belladonna Alkaloids-Opium	belladonna-opium
574711012	Morphine Sulfate	morphine
574711212	Morphine Sulfate	morphine
574711412	Morphine Sulfate	morphine
574722406	Hydromorphone Hydrochloride	hydromorphone
590012770	Percocet	acetaminophen-oxycodone
590012775	Percocet	acetaminophen-oxycodone
590013565	Percodan	aspirin-oxycodone
590013570	Percodan	aspirin-oxycodone
590037310	Numorphan HCl	oxymorphone
590043210	Nubain	nalbuphine
590043310	Nubain	nalbuphine
591034901	Acetaminophen-Hydrocodone B	acetaminophen-hydrocodo
591034905	Acetaminophen-Hydrocodone B	acetaminophen-hydrocodo
591038501	Acetaminophen-Hydrocodone B	acetaminophen-hydrocodo
591038505	Acetaminophen-Hydrocodone B	acetaminophen-hydrocodo
591038705	Acetaminophen-Hydrocodone B	acetaminophen-hydrocodo
591038801	Acetaminophen-Hydrocodone B	acetaminophen-hydrocodo
591039601	Acetaminophen-Pentazocine	acetaminophen-pentazoci
591042501	Aspirin-Butalbital-Caffeine	ASA/butalbital/caffeine
591054001	Acetaminophen-Hydrocodone B	acetaminophen-hydrocodo
591072701	Meperidine Hydrochloride	meperidine
591082001	Aspirin/Oxycodone HCl/Oxyco	aspirin-oxycodone
591082401	Acetaminophen-Oxycodone Hyd	acetaminophen-oxycodone
591082501	Acetaminophen-Oxycodone Hyd	acetaminophen-oxycodone
591085301	Acetaminophen-Hydrocodone B	acetaminophen-hydrocodo
591093201	Acetaminophen-Oxycodone Hyd	acetaminophen-oxycodone
591093301	Acetaminophen-Oxycodone Hyd	acetaminophen-oxycodone

591260501	Acetaminophen-HYDROcodone B	acetaminophen-HYDROcodo
591261201	Acetaminophen-HYDROcodone B	acetaminophen-HYDROcodo
591316801	Hydrocodone-Ibuprofen	hydrocodone-ibuprofen
591319872	Fentanyl	fentanyl
591320201	Acetaminophen-Hydrocodone B	acetaminophen-hydrocodo
591320205	Acetaminophen-HYDROcodone B	acetaminophen-HYDROcodo
591320301	Acetaminophen-Hydrocodone B	acetaminophen-hydrocodo
591321272	Fentanyl	fentanyl
591321372	Fentanyl	fentanyl
591321472	Fentanyl	fentanyl
591322001	APAP/Butalbital/Caffeine/Co	APAP/butalbital/caffein
591345001	Morphine Sulfate ER	morphine
591345301	Morphine Sulfate ER	morphine
591350101	Oxycodone Hydrochloride ER	oxycodone
591350201	Oxycodone Hydrochloride ER	oxycodone
591350301	Oxycodone Hydrochloride ER	oxycodone
591351101	Morphine Sulfate SR	morphine
591351201	Morphine Sulfate SR	morphine
591351401	Morphine Sulfate SR	morphine
591354601	Aspirin-Butalbital-Caffeine	ASA/butalbital/caffeine
591355101	Aspirin-OxyCODONE	aspirin-oxyCODONE
591360072	Fentanyl	fentanyl
591360172	Fentanyl	fentanyl
591360272	Fentanyl	fentanyl
591360372	Fentanyl	fentanyl
603102058	Acetaminophen-Codeine Phosp	acetaminophen-codeine
603129558	Acetaminophen-Hydrocodone B	acetaminophen-hydrocodo
603233721	Acetaminophen-Codeine Phosp	acetaminophen-codeine
603233802	Acetaminophen-Codeine Phosp	acetaminophen-codeine
603233804	Acetaminophen-Codeine Phosp	acetaminophen-codeine
603233821	Acetaminophen-Codeine Phosp	acetaminophen-codeine
603233832	Acetaminophen-Codeine Phosp	acetaminophen-codeine
603233921	Acetaminophen-Codeine Phosp	acetaminophen-codeine
603236121	Aspirin-Codeine Phosphate	aspirin-codeine
603255321	APAP/Butalbital/Caffeine/Co	APAP/butalbital/caffein
603388021	Acetaminophen-Hydrocodone B	acetaminophen-hydrocodo
603388121	Acetaminophen-Hydrocodone B	acetaminophen-hydrocodo
603388132	Acetaminophen-Hydrocodone B	acetaminophen-hydrocodo
603388321	Acetaminophen-Hydrocodone B	acetaminophen-hydrocodo
603388521	Acetaminophen-Hydrocodone B	acetaminophen-hydrocodo
603388721	Acetaminophen-Hydrocodone B	acetaminophen-hydrocodo
603388728	Acetaminophen-Hydrocodone B	acetaminophen-hydrocodo
603388821	Acetaminophen-Hydrocodone B	acetaminophen-hydrocodo
603388921	Acetaminophen-Hydrocodone B	acetaminophen-hydrocodo
603389021	Acetaminophen-Hydrocodone B	acetaminophen-hydrocodo

603389028	Acetaminophen-Hydrocodone B	acetaminophen-hydrocodo
603389121	Acetaminophen-Hydrocodone B	acetaminophen-hydrocodo
603389128	Acetaminophen-Hydrocodone B	acetaminophen-hydrocodo
603389721	Hydrocodone-Ibuprofen	hydrocodone-ibuprofen
603392621	Hydromorphone Hydrochloride	hydromorphone
603441521	Meperidine Hydrochloride	meperidine
603442421	Meperidine HCl-Promethazine	meperidine-promethazine
603497921	Acetaminophen-OxyCODONE Hyd	acetaminophen-oxyCODONE
603499021	Oxycodone Hydrochloride	oxycodone
603499121	Oxycodone Hydrochloride	oxycodone
603499221	Oxycodone Hydrochloride	oxycodone
603499821	Acetaminophen-Oxycodone Hyd	acetaminophen-oxycodone
603499828	Acetaminophen-Oxycodone Hyd	acetaminophen-oxycodone
603901354	Acetaminophen-Codeine Phosp	acetaminophen-codeine
603901358	Acetaminophen-Codeine Phosp	acetaminophen-codeine
615043013	Acetaminophen-Codeine Phosp	acetaminophen-codeine
641010025	Codeine Phosphate	codeine
641011025	Codeine Phosphate	codeine
641012125	Hydromorphone Hydrochloride	hydromorphone
641013025	Meperidine Hydrochloride	meperidine
641014025	Meperidine Hydrochloride	meperidine
641015025	Meperidine Hydrochloride	meperidine
641015026	Meperidine Hydrochloride	meperidine
641016025	Meperidine Hydrochloride	meperidine
641016825	Morphine Sulfate	morphine
641017025	Morphine Sulfate	morphine
641018025	Morphine Sulfate	morphine
641019025	Morphine Sulfate	morphine
641111233	Duramorph PF	morphine
641111433	Duramorph PF	morphine
641111633	Fentanyl Citrate	fentanyl
641111733	Fentanyl Citrate	fentanyl
641111834	Fentanyl Citrate	fentanyl
641111934	Fentanyl Citrate	fentanyl
641113035	Meperidine Hydrochloride	meperidine
641113131	Infumorph	morphine
641113231	Infumorph	morphine
641114035	Meperidine Hydrochloride	meperidine
641115035	Meperidine Hydrochloride	meperidine
641118035	Morphine Sulfate	morphine
641119035	Morphine Sulfate	morphine
641234141	Hydromorphone Hydrochloride	hydromorphone
641234341	Morphine Sulfate	morphine
641234541	Morphine Sulfate	morphine
641240241	Fentanyl Citrate	fentanyl

641240341	Fentanyl Citrate	fentanyl
641601910	Duramorph PF	morphine
641602010	Duramorph PF	morphine
641602410	FentaNYL Citrate	fentaNYL
641602510	FentaNYL Citrate	fentaNYL
641602605	FentaNYL Citrate	fentaNYL
641602725	FentaNYL Citrate	fentaNYL
641602825	FentaNYL Citrate	fentaNYL
641602925	FentaNYL Citrate	fentaNYL
641603001	FentaNYL Citrate	fentaNYL
641603901	Infumorph	morphine
641604001	Infumorph	morphine
641605225	Meperidine Hydrochloride	meperidine
641605325	Meperidine Hydrochloride	meperidine
641605425	Meperidine Hydrochloride	meperidine
641606801	Morphine Sulfate	morphine
641607001	Morphine Sulfate	morphine
641607025	Morphine Sulfate	morphine
641607125	Morphine Sulfate	morphine
641607201	Morphine Sulfate	morphine
641607325	Morphine Sulfate	morphine
641607525	Morphine Sulfate	morphine
641612525	Morphine Sulfate	morphine
641612625	Morphine Sulfate	morphine
641612725	Morphine Sulfate	morphine
677061101	Acetaminophen-Codeine Phosp	acetaminophen-codeine
677063201	Acetaminophen-Codeine Phosp	acetaminophen-codeine
703001801	Hydromorphone Hydrochloride	hydromorphone
703011003	Hydromorphone Hydrochloride	hydromorphone
703011303	Hydromorphone Hydrochloride	hydromorphone
713019312	Morphine Sulfate	morphine
713019412	Morphine Sulfate	morphine
781152401	Acetaminophen-Hydrocodone B	acetaminophen-hydrocodo
781222101	Fiortal with Codeine	ASA/butalbital/caffeine
781710955	Fentanyl	fentanyl
781711155	Fentanyl	fentanyl
781711255	Fentanyl	fentanyl
781711355	Fentanyl	fentanyl
781711455	Fentanyl	fentanyl
781724055	Fentanyl Matrix	fentanyl
781724155	Fentanyl Matrix	fentanyl
781724255	Fentanyl Matrix	fentanyl
781724355	Fentanyl Matrix	fentanyl
781724455	Fentanyl Matrix	fentanyl
785112201	Lorcet Plus	acetaminophen-hydrocodo

785635001	Lorcet 10/650	acetaminophen-hydrocodo
785635063	Lorcet 10/650	acetaminophen-hydrocodo
832022500	Morphine Sulfate ER	morphine
832022600	Morphine Sulfate ER	morphine
832022700	Morphine Sulfate ER	morphine
832022800	Morphine Sulfate ER	morphine
904344061	Acetaminophen-Hydrocodone B	acetaminophen-hydrocodo
904390060	Aspirin-Codeine Phosphate	aspirin-codeine
904514060	Aspirin-Butalbital-Caffeine	ASA/butalbital/caffeine
904555661	Tramadol Hydrochloride	tramadol
904611961	TraMADOL Hydrochloride	traMADOL
904636561	TraMADol Hydrochloride	traMADol
904641961	Acetaminophen-HYDROcodone B	acetaminophen-HYDROcodo
904642061	Acetaminophen-HYDROcodone B	acetaminophen-HYDROcodo
904642161	Acetaminophen-HYDROcodone B	acetaminophen-HYDROcodo
904643761	Acetaminophen-OxyCODONE Hyd	acetaminophen-oxyCODONE
904643861	Acetaminophen-OxyCODONE Hyd	acetaminophen-oxyCODONE
904643961	Acetaminophen-OxyCODONE Hyd	acetaminophen-oxyCODONE
904644461	OxyCODONE Hydrochloride	oxyCODONE
904644561	OxyCODONE Hydrochloride	oxyCODONE
904653061	Methadone Hydrochloride	methadone
904656761	Acetaminophen-Hydrocodone B	acetaminophen-hydrocodo
904763261	Acetaminophen-Hydrocodone B	acetaminophen-hydrocodo
998501675	B & O Supprettes 16-A	belladonna-opium
10019000673	Morphine Sulfate	morphine
10019000773	Morphine Sulfate	morphine
10019003372	Fentanyl Citrate	fentanyl
10019003473	Fentanyl Citrate	fentanyl
10019003574	Fentanyl Citrate	fentanyl
10019003682	Fentanyl Citrate	fentanyl
10019003725	Fentanyl Citrate	fentanyl
10019003727	Fentanyl Citrate	fentanyl
10019003730	Fentanyl Citrate	fentanyl
10019003783	Fentanyl Citrate	fentanyl
10019003867	Fentanyl Citrate	fentanyl
10019015147	Meperidine Hydrochloride	meperidine
10019015244	Meperidine Hydrochloride	meperidine
10019015247	Meperidine Hydrochloride	meperidine
10019015347	Meperidine Hydrochloride	meperidine
10019015444	Meperidine Hydrochloride	meperidine
10019015447	Meperidine Hydrochloride	meperidine
10019015568	Meperidine Hydrochloride	meperidine
10019015668	Meperidine Hydrochloride	meperidine
10019015768	Meperidine Hydrochloride	meperidine
10019015901	Meperidine Hydrochloride	meperidine

10019016001	Meperidine Hydrochloride	meperidine
10019016101	Meperidine Hydrochloride	meperidine
10019016201	Meperidine Hydrochloride	meperidine
10019017644	Morphine Sulfate	morphine
10019017744	Morphine Sulfate	morphine
10019017747	Morphine Sulfate	morphine
10019017768	Morphine Sulfate	morphine
10019017844	Morphine Sulfate	morphine
10019017847	Morphine Sulfate	morphine
10019017862	Morphine Sulfate	morphine
10019017868	Morphine Sulfate	morphine
10019017944	Morphine Sulfate	morphine
10019017963	Morphine Sulfate	morphine
10019018265	Morphine Sulfate	morphine
10019031122	Revex	nalmefene
10019031521	Revex	nalmefene
10019046101	Butorphanol Tartrate	butorphanol
10147090103	TraMADOL Hydrochloride ER	traMADOL
10147090203	TraMADOL Hydrochloride ER	traMADOL
10370022111	TraMADol Hydrochloride ER	traMADol
10702000801	Oxycodone Hydrochloride	oxycodone
10702000901	Oxycodone Hydrochloride	oxycodone
10702001801	Oxycodone Hydrochloride	oxycodone
10702005601	Oxycodone Hydrochloride	oxycodone
10702005701	Oxycodone Hydrochloride	oxycodone
10702007001	Oxymorphone Hydrochloride	oxymorphone
10702007101	Oxymorphone Hydrochloride	oxymorphone
11098003002	MRV	fentanyl
11098003005	MRV	fentanyl
11098003020	MRV	fentanyl
12496075701	Buprenex	buprenorphine
12496075705	Buprenex	buprenorphine
13107001901	Acetaminophen-HYDROcodone B	acetaminophen-HYDROcodo
13107002001	Acetaminophen-HYDROcodone B	acetaminophen-HYDROcodo
13107002101	Acetaminophen-HYDROcodone B	acetaminophen-HYDROcodo
13107004401	Acetaminophen-OxyCODONE Hyd	acetaminophen-oxyCODONE
13107004501	Acetaminophen-OxyCODONE Hyd	acetaminophen-oxyCODONE
13107005801	Acetaminophen-Codeine Phosp	acetaminophen-codeine
13551080105	Hycet	acetaminophen-hydrocodo
16714011104	TraMADol Hydrochloride	traMADol
16714011110	TraMADol Hydrochloride	traMADol
16714011111	TraMADol Hydrochloride	traMADol
16714011112	TraMADol Hydrochloride	traMADol
16714048103	TraMADol Hydrochloride	traMADol
16887021105	Apokyn	apomorphine

17478003002	Sublimaze	fentaNYL
17478003005	Sublimaze	fentaNYL
17478003020	Sublimaze	fentaNYL
17478003025	Sublimaze	fentaNYL
17478003055	Sublimaze	fentaNYL
17478045016	Lortab	acetaminophen-HYDROcodo
17478054001	Hydromorphone Hydrochloride	hydromorphone
17478054005	Hydromorphone Hydrochloride	hydromorphone
17478054050	Hydromorphone Hydrochloride	hydromorphone
20482000130	Subsys	fentaNYL
20482000210	Subsys	fentaNYL
20482000630	Subsys	fentaNYL
20482000830	Subsys	fentaNYL
23635011501	Xartemis XR	acetaminophen-oxyCODONE
23635040801	Exalgo	hydromorphone
23635041201	Exalgo	hydromorphone
23635041601	Exalgo	hydromorphone
24200025201	FentaNYL Citrate	fentaNYL
24200025244	FentaNYL Citrate	fentaNYL
24200025245	FentaNYL Citrate	fentaNYL
24200025249	FentaNYL Citrate	fentaNYL
24200025251	FentaNYL Citrate	fentaNYL
24200025261	FentaNYL Citrate	fentaNYL
24200025264	Fentanyl Citrate	fentanyl
24200025280	FentaNYL Citrate	fentaNYL
24200025310	Fentanyl Citrate	fentanyl
24200025444	FentaNYL Citrate	fentaNYL
24200025445	FentaNYL Citrate	fentaNYL
24200025464	FentaNYL Citrate	fentaNYL
24200025580	FentaNYL Citrate	fentaNYL
24200025744	FentaNYL Citrate	fentaNYL
24200029261	Hydromorphone Hydrochloride	hydromorphone
24200029264	Hydromorphone Hydrochloride	hydromorphone
24200029361	Hydromorphone Hydrochloride	hydromorphone
24200029364	HYDROmorphone Hydrochloride	HYDROmorphone
24200029680	Hydromorphone Hydrochloride	hydromorphone
24200029690	HYDROmorphone Hydrochloride	HYDROmorphone
24200029780	Hydromorphone Hydrochloride	hydromorphone
24200032004	Morphine Sulfate	morphine
24200032145	Morphine Sulfate	morphine
24200032282	Morphine Sulfate	morphine
24200032404	Morphine Sulfate	morphine
24200033545	Morphine Sulfate	morphine
24200043204	Bupivacaine-Fentanyl	bupivacaine-fentanyl
24200043211	Bupivacaine-FentaNYL	bupivacaine-fentaNYL

24200043704	Bupivacaine-Fentanyl	bupivacaine-fentanyl
24200043710	Bupivacaine-Fentanyl	bupivacaine-fentanyl
24200043810	Bupivacaine-Fentanyl	bupivacaine-fentanyl
24200048804	Fentanyl Citrate-Ropivacain	fentanyl-ropivacaine
24200048810	Fentanyl Citrate-Ropivacain	fentanyl-ropivacaine
24200049310	FentaNYL Citrate-Ropivacain	fentaNYL-ropivacaine
24200049404	FentaNYL Citrate-Ropivacain	fentaNYL-ropivacaine
24200229664	Hydromorphone Hydrochloride	hydromorphone
24477002004	DepoDur	morphine liposomal
27808003601	Acetaminophen-HYDROcodone B	acetaminophen-HYDROcodo
35356004800	Kadian	morphine
35356005000	Kadian	morphine
35356009030	Oxycodone Hydrochloride ER	oxycodone
38779067340	Morphine Sulfate	morphine
38779175609	Fentanyl Citrate	fentanyl
39769035310	Morphine Sulfate	morphine
39769035820	Morphine Sulfate	morphine
42806000801	OxyCODONE Hydrochloride	oxyCODONE
42806005001	Meperidine Hydrochloride	meperidine
42858000101	OxyCODONE Hydrochloride	oxyCODONE
42858000110	OxyCODONE Hydrochloride	oxyCODONE
42858000201	OxyCODONE Hydrochloride	oxyCODONE
42858000210	OxyCODONE Hydrochloride	oxyCODONE
42858000301	OxyCODONE Hydrochloride	oxyCODONE
42858000501	OxyCODONE Hydrochloride	oxyCODONE
42858010201	Acetaminophen-OxyCODONE Hyd	acetaminophen-oxyCODONE
42858010301	Acetaminophen-OxyCODONE Hyd	acetaminophen-oxyCODONE
42858010401	Acetaminophen-OxyCODONE Hyd	acetaminophen-oxyCODONE
42858030101	Hydromorphone Hydrochloride	hydromorphone
42858030125	Hydromorphone Hydrochloride	hydromorphone
42858030201	Hydromorphone Hydrochloride	hydromorphone
42858030225	Hydromorphone Hydrochloride	hydromorphone
42858030301	Hydromorphone Hydrochloride	hydromorphone
42858030416	HYDROmorphone Hydrochloride	HYDROmorphone
42858080101	Morphine Sulfate ER	morphine
42858080201	Morphine Sulfate ER	morphine
42858080301	Morphine Sulfate ER	morphine
42858080401	Morphine Sulfate ER	morphine
42998020301	Opium Deodorized	opium
43063000404	Acetaminophen-Hydrocodone B	acetaminophen-hydrocodo
43063001404	Acetaminophen-Codeine Phosp	acetaminophen-codeine
43063002504	Acetaminophen-Oxycodone Hyd	acetaminophen-oxycodone
43063002506	Acetaminophen-Oxycodone Hyd	acetaminophen-oxycodone
43063003106	Acetaminophen-Hydrocodone B	acetaminophen-hydrocodo
43063005506	TraMADOL Hydrochloride	tramadol

43063009106	Acetaminophen-Hydrocodone B	acetaminophen-hydrocodo
43063026506	Acetaminophen-Hydrocodone B	acetaminophen-hydrocodo
43063030406	OxyCODONE Hydrochloride	oxyCODONE
43063035402	OxyCONTIN	oxyCODONE
43376021010	Zohydro ER	HYDROcodone
43376022010	Zohydro ER	HYDROcodone
43376023010	Zohydro ER	HYDROcodone
43376024010	Zohydro ER	HYDROcodone
43376031060	Zohydro ER	HYDROcodone
43386092060	OxyCODONE Hydrochloride	oxyCODONE
46987032211	Kadian	morphine
46987032311	Kadian	morphine
46987032411	Kadian	morphine
46987032511	Kadian	morphine
46987032611	Kadian	morphine
46987037711	Kadian	morphine
46987041011	Kadian	morphine
46987041211	Kadian	morphine
47335085983	TraMADol Hydrochloride ER	traMADol
47335086183	TraMADol Hydrochloride ER	traMADol
47781022901	Acetaminophen-OxyCODONE Hyd	acetaminophen-oxyCODONE
47781023001	Acetaminophen-OxyCODONE Hyd	acetaminophen-oxyCODONE
49884045955	FentaNYL Citrate	fentaNYL
49884046055	FentaNYL Citrate	fentaNYL
49884046255	FentaNYL Citrate	fentaNYL
49884066601	Morphine Sulfate ER	morphine
49884066701	Morphine Sulfate ER	morphine
49884076178	FentaNYL	fentaNYL
49884076278	FentaNYL	fentaNYL
49884076378	FentaNYL	fentaNYL
49884076478	FentaNYL	fentaNYL
49884082111	TraMADOL Hydrochloride ER	traMADOL
49884082211	TraMADOL Hydrochloride ER	traMADOL
49884082311	TraMADol Hydrochloride ER	traMADol
49884083301	Morphine Sulfate ER	morphine
49884083401	Morphine Sulfate ER	morphine
49884083501	Morphine Sulfate ER	morphine
49884083601	Morphine Sulfate ER	morphine
49884083801	Morphine Sulfate ER	morphine
49884094601	Acetaminophen-Tramadol Hydr	acetaminophen-tramadol
50111061601	Tramadol	tramadol
50268040115	Acetaminophen-HYDROcodone B	acetaminophen-HYDROcodo
50268040215	Acetaminophen-HYDROcodone B	acetaminophen-HYDROcodo
50268040315	Acetaminophen-HYDROcodone B	acetaminophen-HYDROcodo
50268040815	Acetaminophen-HYDROcodone B	acetaminophen-HYDROcodo

50268077315	Acetaminophen-TraMADol Hydr	acetaminophen-traMADol
50268077415	Acetaminophen-TraMADol Hydr	acetaminophen-traMADol
50383007906	Acetaminophen-Codeine Phosp	acetaminophen-codeine
50383007913	Acetaminophen-Codeine Phosp	acetaminophen-codeine
50383007916	Acetaminophen-Codeine Phosp	acetaminophen-codeine
50458002002	Innovar	droperidol-fentanyl
50458003002	MRV	fentanyl
50458003005	MRV	fentanyl
50458003020	MRV	fentanyl
50458003305	Duragesic	fentanyl
50458003405	Duragesic	fentanyl
50458003505	Duragesic	fentanyl
50458003605	Duragesic	fentanyl
50458003705	Duragesic	fentanyl
50458009005	Duragesic-12	fentanyl
50458009105	Duragesic-25	fentanyl
50458009205	Duragesic-50	fentanyl
50458009305	Duragesic-75	fentanyl
50458009405	Duragesic-100	fentanyl
50458051360	Tylenol with Codeine #3	acetaminophen-codeine
50458051570	Tylenol with Codeine #4	acetaminophen-codeine
50458065060	Ultracet	acetaminophen-traMADOL
50458065330	Ultram ER	traMADOL
50458065530	Ultram ER	traMADOL
50458065730	Ultram ER	traMADOL
50458065960	Ultram	traMADOL
50458082002	Nucynta	tapentadol
50458082004	Nucynta	tapentadol
50458083002	Nucynta	tapentadol
50458083004	Nucynta	tapentadol
50458084002	Nucynta	tapentadol
50458084004	Nucynta	tapentadol
50458086001	Nucynta ER	tapentadol
50458086101	Nucynta ER	tapentadol
50458086201	Nucynta ER	tapentadol
50458086301	Nucynta ER	tapentadol
50458086401	Nucynta ER	tapentadol
50474050001	Lortab ASA	aspirin-hydrocodone
50474090201	Lortab 5/500	acetaminophen-hydrocodo
50474090260	Lortab 5/500	acetaminophen-hydrocodo
50474090701	Lortab 7.5/500	acetaminophen-hydrocodo
50474090750	Lortab 7.5/500	acetaminophen-hydrocodo
50474090760	Lortab 7.5/500	acetaminophen-hydrocodo
50474090916	Lortab Elixir	acetaminophen-hydrocodo
50474091001	Lortab 10	acetaminophen-hydrocodo

50474091050	Lortab 10	acetaminophen-hydrocodo
50474091060	Lortab 10	acetaminophen-hydrocodo
50474092501	Lortab	acetaminophen-hydrocodo
51079010620	Acetaminophen-Codeine Phosp	acetaminophen-codeine
51079010621	Acetaminophen-Codeine Phosp	acetaminophen-codeine
51079016120	Acetaminophen-Codeine Phosp	acetaminophen-codeine
51079016121	Acetaminophen-Codeine Phosp	acetaminophen-codeine
51079016199	Acetaminophen-Codeine Phosp	acetaminophen-codeine
51079025421	Acetaminophen-Hydrocodone B	acetaminophen-hydrocodo
51079042020	Acetaminophen-Hydrocodone B	acetaminophen-hydrocodo
51079042021	Acetaminophen-Hydrocodone B	acetaminophen-hydrocodo
51079042099	Acetaminophen-Hydrocodone B	acetaminophen-hydrocodo
51079059371	Morphine Sulfate	morphine
51079059471	Morphine Sulfate	morphine
51079074821	Acetaminophen-Hydrocodone B	acetaminophen-hydrocodo
51079077720	Acetaminophen-HYDROcodone B	acetaminophen-HYDROcodo
51079077721	Acetaminophen-Hydrocodone B	acetaminophen-hydrocodo
51079077820	Acetaminophen-HYDROcodone B	acetaminophen-HYDROcodo
51079077821	Acetaminophen-Hydrocodone B	acetaminophen-hydrocodo
51079077920	Acetaminophen-HYDROcodone B	acetaminophen-HYDROcodo
51079077921	Acetaminophen-Hydrocodone B	acetaminophen-hydrocodo
51079078099	Acetaminophen-Hydrocodone B	acetaminophen-hydrocodo
51079086721	Acetaminophen-Hydrocodone B	acetaminophen-hydrocodo
51079093320	Acetaminophen-Hydrocodone B	acetaminophen-hydrocodo
51079093321	Acetaminophen-Hydrocodone B	acetaminophen-hydrocodo
51079099120	Tramadol	tramadol
51079099156	Tramadol Hydrochloride	tramadol
51224030010	Codeine Sulfate	codeine
51285030402	Acetaminophen-Codeine Phosp	acetaminophen-codeine
51991007301	APAP/Butalbital/Caffeine/Co	APAP/butalbital/caffein
51991007401	Aspirin-Butalbital-Caffeine	ASA/butalbital/caffeine
52152004102	Acetaminophen-Oxycodone Hyd	acetaminophen-oxycodone
52152016502	Oxycodone Hydrochloride	oxycodone
52152019002	Meperidine HCl-Promethazine	meperidine-promethazine
52152021302	Acetaminophen-Pentazocine	acetaminophen-pentazoci
52152021402	Oxycodone Hydrochloride	oxycodone
52152021411	Oxycodone Hydrochloride	oxycodone
52152021502	Oxycodone Hydrochloride	oxycodone
52533000203	HYDROmorphone Hydrochloride	HYDROmorphone
52533000204	HYDROmorphone Hydrochloride	HYDROmorphone
52533000205	HYDROmorphone Hydrochloride	HYDROmorphone
52533000243	HYDROmorphone Hydrochloride	HYDROmorphone
52533000604	HYDROmorphone Hydrochloride	HYDROmorphone
52533002404	FentaNYL Citrate	fentaNYL
52533002461	FentaNYL Citrate	fentaNYL

52533007416	FentaNYL Citrate	fentaNYL
52533016004	Morphine Sulfate	morphine
52533016043	Morphine Sulfate	morphine
52533016075	Morphine Sulfate	morphine
52544016101	Norco	acetaminophen-HYDROcodo
52544016201	Norco	acetaminophen-HYDROcodo
52544016460	Kadian	morphine
52544034901	Acetaminophen-Hydrocodone B	acetaminophen-hydrocodo
52544038501	Acetaminophen-Hydrocodone B	acetaminophen-hydrocodo
52544038801	Acetaminophen-Hydrocodone B	acetaminophen-hydrocodo
52544042501	Aspirin-Butalbital-Caffeine	ASA/butalbital/caffeine
52544050201	Acetaminophen-Hydrocodone B	acetaminophen-hydrocodo
52544053901	Norco	acetaminophen-hydrocodo
52544054001	Acetaminophen-Hydrocodone B	acetaminophen-hydrocodo
52544072601	Meperidine Hydrochloride	meperidine
52544072901	Norco	acetaminophen-hydrocodo
52544091301	Norco	acetaminophen-hydrocodo
52544091348	Norco	acetaminophen-hydrocodo
52544095601	Fiorinal with Codeine	ASA/butalbital/caffeine
52544095801	Fiorinal with Codeine	ASA/butalbital/caffeine
52959018610	Lortab 7.5/500	acetaminophen-hydrocodo
52959066660	Ultracet	acetaminophen-tramadol
53489049901	Tramadol	tramadol
53489049905	Tramadol	tramadol
53746010901	Acetaminophen-Hydrocodone B	acetaminophen-hydrocodo
53746010905	Acetaminophen-Hydrocodone B	acetaminophen-hydrocodo
53746011001	Acetaminophen-Hydrocodone B	acetaminophen-hydrocodo
53746011005	Acetaminophen-Hydrocodone B	acetaminophen-hydrocodo
53746011801	Acetaminophen-Hydrocodone B	acetaminophen-hydrocodo
53746014501	Hydrocodone-Ibuprofen	hydrocodone-ibuprofen
53746020301	Acetaminophen-Oxycodone Hyd	acetaminophen-oxycodone
53746020401	Acetaminophen-Oxycodone Hyd	acetaminophen-oxycodone
53746061701	Acetaminophen-TraMADol Hydr	acetaminophen-traMADol
54569141600	Buprenex	buprenorphine
54569481900	Nubain	nalbuphine
54868107101	Demerol HCl	meperidine
54868381300	OxyContin	oxycodone
54868381501	OxyContin	oxycodone
55253007030	Fentanyl Citrate	fentanyl
55253007130	Fentanyl Citrate	fentanyl
55253007230	Fentanyl Citrate	fentanyl
55253007330	Fentanyl Citrate	fentanyl
55289000560	Acetaminophen-Codeine Phosp	acetaminophen-codeine
55289099704	Acetaminophen-HYDROcodone B	acetaminophen-HYDROcodo
55390018301	Butorphanol Tartrate	butorphanol

55390018401	Butorphanol Tartrate	butorphanol
55390018402	Butorphanol Tartrate	butorphanol
55390018510	Butorphanol Tartrate	butorphanol
55390034110	Butorphanol Tartrate	butorphanol
55390034210	Butorphanol Tartrate	butorphanol
57480050001	Acetaminophen-Codeine Phosp	acetaminophen-codeine
57664012613	Acetaminophen-Hydrocodone B	acetaminophen-hydrocodo
57664012688	Acetaminophen-Hydrocodone B	acetaminophen-hydrocodo
57664017088	Acetaminophen-Hydrocodone B	acetaminophen-hydrocodo
57664017688	Acetaminophen-Hydrocodone B	acetaminophen-hydrocodo
57664018788	Oxycodone Hydrochloride	oxycodone
57664022388	Oxycodone Hydrochloride	oxycodone
57664022488	Oxycodone Hydrochloride	oxycodone
57664037088	OxyCODONE Hydrochloride	oxyCODONE
57664037708	Tramadol	tramadol
57664037713	Tramadol Hydrochloride	tramadol
57664037718	Tramadol Hydrochloride	tramadol
57664046708	Meperidine Hydrochloride	meperidine
57664053788	Acetaminophen-Tramadol Hydr	acetaminophen-tramadol
58177002704	Meperidine HCl-Promethazine	meperidine-promethazine
58177004104	Oxycodone Hydrochloride	oxycodone
58177004111	Oxycodone Hydrochloride	oxycodone
58177029804	Hydromorphone	hydromorphone
58177029811	Hydromorphone Hydrochloride	hydromorphone
58177029904	Hydromorphone	hydromorphone
58177029911	Hydromorphone Hydrochloride	hydromorphone
58177031004	Morphine Sulfate SR	morphine
58177031304	Morphine IR	morphine
58177031404	Morphine IR	morphine
58177031504	Oxycodone Hydrochloride	oxycodone
58177031511	Oxycodone Hydrochloride	oxycodone
58177044504	Oxycodone Hydrochloride	oxycodone
58177044604	Oxycodone Hydrochloride	oxycodone
58177062004	Hydromorphone Hydrochloride	hydromorphone
58177062011	Hydromorphone Hydrochloride	hydromorphone
58177062111	Hydromorphone Hydrochloride	hydromorphone
58177062504	Oxycodone Hydrochloride	oxycodone
58177062511	Oxycodone Hydrochloride	oxycodone
58177088601	Morphine Sulfate	morphine
58177088603	Morphine Sulfate	morphine
58177088605	Morphine Sulfate	morphine
58177088656	Morphine Sulfate	morphine
58177088657	Morphine Sulfate	morphine
58177088880	Morphine Sulfate	morphine
58177088981	Morphine Sulfate	morphine

58177090907	Acetaminophen-Hydrocodone B	acetaminophen-hydrocodo
58177091401	Oxydose	oxycodone
58177091456	ETH-Oxydose	oxycodone
59011010010	OxyContin	oxycodone
59011010020	OxyContin	oxycodone
59011010025	OxyContin	oxycodone
59011010310	OxyContin	oxycodone
59011010320	OxyContin	oxycodone
59011010325	OxyContin	oxycodone
59011010510	OxyContin	oxycodone
59011010520	OxyContin	oxycodone
59011010525	OxyContin	oxycodone
59011010710	OxyContin	oxycodone
59011010720	OxyContin	oxycodone
59011010725	OxyContin	oxycodone
59011010910	OxyContin	oxycodone
59011010925	OxyContin	oxycodone
59011020110	Oxyir	oxycodone
59011022520	Oxyfast	oxycodone
59011026010	MS Contin	morphine
59011026125	MS Contin	morphine
59011026210	MS Contin	morphine
59011026310	MS Contin	morphine
59011026410	MS Contin	morphine
59011031260	Palladone	hydromorphone
59011031360	Palladone	hydromorphone
59011033430	Ryzolt	tramadol
59011033530	Ryzolt	tramadol
59011041010	OxyContin	oxycodone
59011041020	OxyContin	oxycodone
59011041510	OxyContin	oxycodone
59011041520	OxyContin	oxycodone
59011042010	OxyContin	oxycodone
59011042020	OxyContin	oxycodone
59011043010	OxyContin	oxycodone
59011043020	OxyContin	oxycodone
59011044010	OxyContin	oxycodone
59011044020	OxyContin	oxycodone
59011044110	Dilaudid	hydromorphone
59011044210	Dilaudid	hydromorphone
59011044225	Dilaudid	hydromorphone
59011044410	Dilaudid	hydromorphone
59011044501	Dilaudid-HP	hydromorphone
59011044505	Dilaudid-HP	hydromorphone
59011044550	Dilaudid-HP	hydromorphone

59011044625	Dilaudid-HP	hydromorphone
59011045101	Dilaudid-5	hydromorphone
59011045201	Dilaudid	hydromorphone
59011045210	Dilaudid	hydromorphone
59011045401	Dilaudid	hydromorphone
59011045410	Dilaudid	hydromorphone
59011045810	Dilaudid	hydromorphone
59011046010	OxyContin	oxycodone
59011046020	OxyContin	oxycodone
59011048010	OxyContin	oxycodone
59011048020	OxyContin	oxycodone
59011075004	Butrans	buprenorphine
59011075104	Butrans	buprenorphine
59011075204	Butrans	buprenorphine
59011081510	OxyContin	oxycodone
59011083010	OxyContin	oxycodone
59702067416	Zolvit	acetaminophen-hydrocodo
60258072016	Acetaminophen-Hydrocodone B	acetaminophen-hydrocodo
60432012200	Morphine Sulfate	morphine
60432024504	Acetaminophen-Codeine Phosp	acetaminophen-codeine
60432024516	Acetaminophen-Codeine Phosp	acetaminophen-codeine
60505017108	TraMADOL Hydrochloride	tramadol
60505065800	Butorphanol Tartrate	butorphanol
60505065900	Butorphanol Tartrate	butorphanol
60505066000	Butorphanol Tartrate	butorphanol
60505081301	Butorphanol Tartrate	butorphanol
60505264401	Acetaminophen-TraMADol Hydr	acetaminophen-traMADol
60505700102	Fentanyl	fentanyl
60505700202	Fentanyl	fentanyl
60505700302	Fentanyl	fentanyl
60505700402	Fentanyl	fentanyl
60505700600	FentaNYL	fentaNYL
60505700602	FentaNYL	fentaNYL
60505700700	FentaNYL	fentaNYL
60505700702	FentaNYL	fentaNYL
60505700800	FentaNYL	fentaNYL
60505700802	FentaNYL	fentaNYL
60505700900	FentaNYL	fentaNYL
60505700902	FentaNYL	fentaNYL
60505701002	FentaNYL	fentaNYL
60505701100	FentaNYL	fentaNYL
60505701102	FentaNYL	fentaNYL
60505701200	FentaNYL	fentaNYL
60505701202	FentaNYL	fentaNYL
60505701300	FentaNYL	fentaNYL

60505701302	FentaNYL	fentaNYL
60505701400	FentaNYL	fentaNYL
60505701402	FentaNYL	fentaNYL
60793043101	Embeda	morphine-naltrexone
60793043401	Embeda	morphine-naltrexone
60793043701	Embeda	morphine-naltrexone
60793060301	Avinza	morphine
60793060501	Avinza	morphine
60793060601	Avinza	morphine
60793060701	Avinza	morphine
60793060801	Avinza	morphine
60951031070	Endodan	aspirin-oxycodone
60951060270	Endocet	acetaminophen-oxycodone
60951060285	Endocet	acetaminophen-oxycodone
60951061070	Endodan	aspirin-oxycodone
60951065270	Morphine Sulfate	morphine
60951065370	Morphine Sulfate	morphine
60951065570	Morphine Sulfate	morphine
60951065870	Morphine Sulfate SR	morphine
60951065970	Morphine Sulfate SR	morphine
60951070070	Endocet	acetaminophen-oxycodone
60951070270	Oxycodone Hydrochloride ER	oxycodone
60951070370	Oxycodone Hydrochloride ER	oxycodone
60951070570	Oxycodone Hydrochloride ER	oxycodone
60951071070	Oxycodone Hydrochloride ER	oxycodone
60951071270	Endocet	acetaminophen-oxycodone
60951079470	Oxymorphone Hydrochloride	oxymorphone
60951079670	Endocet	acetaminophen-oxycodone
60951079770	Acetaminophen-Oxycodone Hyd	acetaminophen-oxycodone
60977001602	Duramorph PF	morphine
60977001673	Duramorph PF	morphine
60977001701	Duramorph PF	morphine
60977001773	Duramorph PF	morphine
60977011401	Infumorph	morphine
60977011501	Infumorph	morphine
61451501507	B & O Supprettes 15-A	belladonna-opium
61451501607	B & O Supprettes 16-A	belladonna-opium
61553001740	Morphine Sulfate	morphine
61553010702	Fentanyl Citrate	fentanyl
61553011148	Fentanyl Citrate	fentanyl
61553011302	Fentanyl Citrate	fentanyl
61553011648	Fentanyl Citrate	fentanyl
61553011841	Fentanyl Citrate	fentanyl
61553012078	Methadone Hydrochloride	methadone
61553012148	Bupivacaine-Fentanyl	bupivacaine-fentanyl

61553012348	Bupivacaine-Fentanyl	bupivacaine-fentanyl
61553012448	Bupivacaine-Fentanyl	bupivacaine-fentanyl
61553012802	Bupivacaine-Fentanyl	bupivacaine-fentanyl
61553012948	Bupivacaine-Fentanyl	bupivacaine-fentanyl
61553013102	Bupivacaine-Fentanyl	bupivacaine-fentanyl
61553013202	Bupivacaine-Fentanyl	bupivacaine-fentanyl
61553013602	Bupivacaine-Fentanyl	bupivacaine-fentanyl
61553013802	Bupivacaine-Fentanyl	bupivacaine-fentanyl
61553013902	Bupivacaine-Fentanyl	bupivacaine-fentanyl
61553013950	Bupivacaine-FentaNYL	bupivacaine-fentaNYL
61553014502	Bupivacaine-Fentanyl	bupivacaine-fentanyl
61553014848	Fentanyl Citrate-Ropivacain	fentanyl-ropivacaine
61553014978	Methadone Hydrochloride	methadone
61553015002	Fentanyl Citrate-Ropivacain	fentanyl-ropivacaine
61553015202	Fentanyl Citrate-Ropivacain	fentanyl-ropivacaine
61553015340	FentaNYL Citrate	fentaNYL
61553015737	Bupicavaine-Hydromorphone	bupivacaine-hydromorpho
61553016141	Hydromorphone Hydrochloride	hydromorphone
61553016244	Hydromorphone Hydrochloride	hydromorphone
61553016267	Hydromorphone Hydrochloride	hydromorphone
61553016375	Hydromorphone Hydrochloride	hydromorphone
61553016541	Hydromorphone Hydrochloride	hydromorphone
61553016644	HYDROmorphone Hydrochloride	HYDROmorphone
61553016667	Hydromorphone Hydrochloride	hydromorphone
61553017041	Meperidine Hydrochloride	meperidine
61553017348	Meperidine Hydrochloride	meperidine
61553017478	FentaNYL Citrate	fentaNYL
61553017741	Morphine Sulfate	morphine
61553017848	Morphine Sulfate	morphine
61553017852	Morphine Sulfate	morphine
61553017948	Morphine Sulfate	morphine
61553018102	Morphine Sulfate	morphine
61553018202	FentaNYL Citrate-Ropivacain	fentaNYL-ropivacaine
61553018348	Morphine Sulfate	morphine
61553018502	Morphine Sulfate	morphine
61553019052	FentaNYL Citrate-Ropivacain	fentaNYL-ropivacaine
61553020137	Bupivacaine-Fentanyl	bupivacaine-fentanyl
61553020437	Bupivacaine-Fentanyl	bupivacaine-fentanyl
61553020502	Bupivacaine-Fentanyl	bupivacaine-fentanyl
61553020550	Bupivacaine-Fentanyl	bupivacaine-fentanyl
61553020648	Hydromorphone Hydrochloride	hydromorphone
61553020701	Hydromorphone Hydrochloride	hydromorphone
61553021302	Fentanyl Citrate-Ropivacain	fentanyl-ropivacaine
61553021837	Bupivacaine-Fentanyl	bupivacaine-fentanyl
61553023902	Bupivacaine-FentaNYL	bupivacaine-fentaNYL

61553024037	FentaNYL Citrate-Ropivacain	fentaNYL-ropivacaine
61553024252	HYDROmorphone Hydrochloride	HYDROmorphone
61553024648	Fentanyl Citrate-Ropivacain	fentanyl-ropivacaine
61553025979	Morphine Sulfate	morphine
61553026037	Fentanyl Citrate-Ropivacain	fentanyl-ropivacaine
61553026102	Fentanyl Citrate-Ropivacain	fentanyl-ropivacaine
61553026401	Fentanyl Citrate-Ropivacain	fentanyl-ropivacaine
61553030379	Fentanyl Citrate	fentanyl
61553030670	Fentanyl Citrate	fentanyl
61553031972	Hydromorphone Hydrochloride	hydromorphone
61553035379	Morphine Sulfate	morphine
61553043961	Fentanyl Citrate	fentanyl
61553050169	Hydromorphone Hydrochloride	hydromorphone
61553050269	Hydromorphone Hydrochloride	hydromorphone
61553050469	Hydromorphone Hydrochloride	hydromorphone
61553050569	Hydromorphone	hydromorphone
61553051069	Hydromorphone Hydrochloride	hydromorphone
61553052169	Fentanyl Citrate	fentanyl
61553052569	Fentanyl Citrate	fentanyl
61553060120	HYDROmorphone Hydrochloride	HYDROmorphone
61553060168	HYDROmorphone Hydrochloride	HYDROmorphone
61553060248	Fentanyl Citrate	fentanyl
61553061175	Meperidine Hydrochloride	meperidine
61553061275	Hydromorphone Hydrochloride	hydromorphone
61553062448	Hydromorphone Hydrochloride	hydromorphone
61553063002	Fentanyl Citrate-Ropivacain	fentanyl-ropivacaine
61553063375	Hydromorphone Hydrochloride	hydromorphone
61553064775	Morphine Sulfate	morphine
61553064844	Morphine Sulfate	morphine
61553064975	Morphine Sulfate	morphine
61553065244	Morphine Sulfate	morphine
61553065376	Morphine Sulfate	morphine
61553065576	Morphine Sulfate	morphine
61553066376	Fentanyl Citrate	fentanyl
61553066444	Fentanyl Citrate	fentanyl
61553066576	Fentanyl Citrate	fentanyl
61553066844	Fentanyl Citrate	fentanyl
61553067020	FentaNYL Citrate	fentaNYL
61553067176	FentaNYL Citrate	fentaNYL
61553067976	Hydromorphone Hydrochloride	hydromorphone
61553068176	Hydromorphone Hydrochloride	hydromorphone
61553068376	Hydromorphone Hydrochloride	hydromorphone
61553068775	Bupivacaine-Fentanyl	bupivacaine-fentanyl
61553069168	HYDROmorphone Hydrochloride	HYDROmorphone
61553070168	Hydromorphone Hydrochloride	hydromorphone

61553070268	Hydromorphone Hydrochloride	hydromorphone
61553070317	Bupivacaine-FentaNYL	bupivacaine-fentaNYL
61553071068	Hydromorphone Hydrochloride	hydromorphone
61553071668	Meperidine Hydrochloride	meperidine
61553073068	Fentanyl Citrate	fentanyl
61553080248	Bupivacaine-FentaNYL	bupivacaine-fentaNYL
61553082041	Morphine Sulfate	morphine
61553082148	Morphine Sulfate	morphine
61553082348	Hydromorphone Hydrochloride	hydromorphone
61553082748	Morphine Sulfate	morphine
61553083041	Hydromorphone Hydrochloride	hydromorphone
61553083820	HYDROmorphone Hydrochloride	HYDROmorphone
61553084148	Meperidine Hydrochloride	meperidine
61553084448	FentaNYL Citrate-Ropivacain	fentaNYL-ropivacaine
61553086348	Bupivacaine-Fentanyl	bupivacaine-fentanyl
61553086948	Bupivacaine-Fentanyl	bupivacaine-fentanyl
61553086950	Bupivacaine-FentaNYL	bupivacaine-fentaNYL
61553087050	Bupivacaine-FentaNYL	bupivacaine-fentaNYL
61553087950	FentaNYL Citrate-Ropivacain	fentaNYL-ropivacaine
61553088050	FentaNYL Citrate-Ropivacain	fentaNYL-ropivacaine
61703022321	Morphine Sulfate	morphine
61703022471	Morphine Sulfate	morphine
61703022472	Morphine Sulfate	morphine
61703022480	Morphine Sulfate	morphine
61703022521	Morphine Sulfate	morphine
61703022543	Morphine Sulfate	morphine
61703022672	Morphine Sulfate	morphine
61703022676	Morphine Sulfate	morphine
61703022680	Morphine Sulfate	morphine
61703024345	Hydromorphone Hydrochloride	hydromorphone
61703024353	Hydromorphone Hydrochloride	hydromorphone
61703024615	Hydromorphone Hydrochloride	hydromorphone
62037052401	Hydrocodone-Ibuprofen	hydrocodone-ibuprofen
62559015116	OxyCODONE Hydrochloride	oxyCODONE
62584055901	Tramadol Hydrochloride	tramadol
62584073801	Acetaminophen-Hydrocodone B	acetaminophen-hydrocodo
63304020301	Opium Deodorized	opium
63304020302	Opium Deodorized	opium
63304040001	Oxycodone Hydrochloride ER	oxycodone
63304045001	Morphine Sulfate ER	morphine
63304045101	Morphine Sulfate ER	morphine
63304045201	Morphine Sulfate ER	morphine
63304056101	Acetaminophen-Codeine Phosp	acetaminophen-codeine
63304056105	Acetaminophen-Codeine Phosp	acetaminophen-codeine
63304070601	Morphine Sulfate	morphine

63304070701	Morphine Sulfate	morphine
63304070801	Morphine Sulfate	morphine
63304074801	Codeine Phosphate	codeine
63304074901	Codeine Phosphate	codeine
63323029110	Astramorph PF	morphine
63323029180	Astramorph PF	morphine
63323029197	Astramorph PF	morphine
63323029210	Astramorph PF	morphine
63323029280	Astramorph PF	morphine
63323029297	Astramorph PF	morphine
634590300224	Actiq	fentanyl
63459030424	Actiq	fentanyl
63459030624	Actiq	fentanyl
63459030824	Actiq	fentanyl
63459031224	Actiq	fentanyl
63459031624	Actiq	fentanyl
63459050230	Actiq	fentanyl
63459050430	Actiq	fentanyl
63459050630	Actiq	fentanyl
63459050830	Actiq	fentanyl
63459051230	Actiq	fentanyl
63459051630	Actiq	fentanyl
63459054128	Fentora	fentanyl
63459054228	Fentora	fentanyl
63459054428	Fentora	fentanyl
63459054628	Fentora	fentanyl
63459054828	Fentora	fentanyl
63481011021	DepoDur	morphine liposomal
63481011521	DepoDur	morphine liposomal
63481012021	DepoDur	morphine liposomal
63481012170	Percodan	aspirin-oxycodone
63481012770	Percocet	acetaminophen-oxycodone
63481012775	Percocet	acetaminophen-oxycodone
63481013270	Percolone	oxycodone
63481013275	Percolone	oxycodone
63481013570	Percodan	aspirin-oxycodone
63481013575	Percodan	aspirin-oxycodone
63481043210	Nubain	nalbuphine
63481043310	Nubain	nalbuphine
63481043670	Opana ER	oxymorphone
63481043870	Opana ER	oxymorphone
63481044410	Numorphan HCl	oxymorphone
63481052270	Opana ER	oxymorphone
63481055370	Opana ER	oxymorphone
63481057170	Opana ER	oxymorphone

63481061270	Opana	oxymorphone
63481061370	Opana	oxymorphone
63481061770	Opana ER	oxymorphone
63481062170	Percocet-7.5/500	acetaminophen-oxycodone
63481062175	Percocet-7.5/500	acetaminophen-oxycodone
63481062275	Percocet-10/650	acetaminophen-oxycodone
63481062370	Percocet-5/325	acetaminophen-oxycodone
63481062375	Percocet-5/325	acetaminophen-oxycodone
63481062410	Opana	oxymorphone
63481062770	Percocet-2.5/325	acetaminophen-oxycodone
63481062870	Percocet 7.5/325	acetaminophen-oxycodone
63481062875	Percocet 7.5/325	acetaminophen-oxycodone
63481062970	Percocet-10/325	acetaminophen-oxycodone
63481062975	Percocet-10/325	acetaminophen-oxycodone
63481067470	Opana ER	oxymorphone
63481069370	Opana ER	oxymorphone
63481069870	Zydone	acetaminophen-hydrocodo
63481081220	Opana ER	oxymorphone
63481081260	Opana ER	oxymorphone
63481081360	Opana ER	oxymorphone
63481081420	Opana ER	oxymorphone
63481081460	Opana ER	oxymorphone
63481081560	Opana ER	oxymorphone
63481081620	Opana ER	oxymorphone
63481081660	Opana ER	oxymorphone
63481081760	Opana ER	oxymorphone
63481081860	Opana ER	oxymorphone
63481090770	Opana ER	oxymorphone
63481090775	Opana ER	oxymorphone
63739000410	Acetaminophen-Codeine Phosp	acetaminophen-codeine
63739000415	Acetaminophen-Codeine Phosp	acetaminophen-codeine
63739000610	Methadone Hydrochloride	methadone
63739013001	Acetaminophen-Hydrocodone B	acetaminophen-hydrocodo
63739013003	Acetaminophen-Hydrocodone B	acetaminophen-hydrocodo
63739013010	Acetaminophen-Hydrocodone B	acetaminophen-hydrocodo
63739013015	Acetaminophen-Hydrocodone B	acetaminophen-hydrocodo
63739013110	Acetaminophen-Hydrocodone B	acetaminophen-hydrocodo
63739014101	Acetaminophen-Hydrocodone B	acetaminophen-hydrocodo
63739014110	Acetaminophen-Hydrocodone B	acetaminophen-hydrocodo
63739014115	Acetaminophen-Hydrocodone B	acetaminophen-hydrocodo
63739021710	Acetaminophen-OxyCODONE Hyd	acetaminophen-oxyCODONE
63739027510	HYDROmorphone Hydrochloride	HYDROmorphone
63739032610	Acetaminophen-Hydrocodone B	acetaminophen-hydrocodo
63739036510	OxyCODONE Hydrochloride	oxyCODONE
63739038410	Acetaminophen-Hydrocodone B	acetaminophen-hydrocodo

63739045501	Acetaminophen-Hydrocodone B	acetaminophen-hydrocodo
63739045510	Acetaminophen-Hydrocodone B	acetaminophen-hydrocodo
63739053210	Acetaminophen-HYDROcodone B	acetaminophen-HYDROcodo
63739055710	Hydrocodone-Ibuprofen	HYDROcodone-ibuprofen
63739067110	TraMADol Hydrochloride	traMADol
63739070410	Acetaminophen-HYDROcodone B	acetaminophen-HYDROcodo
63857032206	Kadian	morphine
63857032211	Kadian	morphine
63857032306	Kadian	morphine
63857032311	Kadian	morphine
63857032406	Kadian	morphine
63857032411	Kadian	morphine
63857032506	Kadian	morphine
63857032511	Kadian	morphine
63857032606	Kadian	morphine
63857032611	Kadian	morphine
63857041011	Kadian	morphine
64019053825	Methadone Hydrochloride	methadone
64019055367	Methadone Hydrochloride	methadone
64365050503	Avinza	morphine
64365050603	Avinza	morphine
64365050702	Avinza	morphine
64365050802	Avinza	morphine
64376061101	APAP/Caffeine/Dihydrocodein	APAP/caffeine/dihydroco
64376064016	Acetaminophen-HYDROcodone B	acetaminophen-HYDROcodo
64376064040	Acetaminophen-HYDROcodone B	acetaminophen-HYDROcodo
64376064801	Acetaminophen-Hydrocodone B	acetaminophen-hydrocodo
64950035303	Oxycodone Hydrochloride	oxycodone
64950035450	OxyCODONE Hydrochloride	oxyCODONE
64950035455	OxyCODONE Hydrochloride	oxyCODONE
65162003310	Acetaminophen-Codeine Phosp	acetaminophen-codeine
65162020710	Acetaminophen-OxyCODONE Hyd	acetaminophen-oxyCODONE
65162062710	Tramadol Hydrochloride	tramadol
65162062711	Tramadol Hydrochloride	tramadol
65162062750	Tramadol Hydrochloride	tramadol
65234004616	Capital with Codeine Suspen	acetaminophen-codeine
65250002004	DepoDur	morphine liposomal
65293001106	Ionsys	fentaNYL
66267075906	Acetaminophen-Codeine Phosp	acetaminophen-codeine
66336014510	Acetaminophen-Oxycodone Hyd	acetaminophen-oxycodone
66479053002	Methadone Hydrochloride	methadone
66479054010	Oramorph SR	morphine
66479054025	Oramorph SR	morphine
66479054110	Oramorph SR	morphine
66479054125	Oramorph SR	morphine

66479054210	Oramorph SR	morphine
66479054225	Oramorph SR	morphine
66479054310	Oramorph SR	morphine
66479054325	Oramorph SR	morphine
66479056003	Roxanol	morphine
66479056012	Roxanol	morphine
66479056024	Roxanol	morphine
66479057416	Hycet	acetaminophen-hydrocodo
66479058010	Roxicodone	oxycodone
66479058025	Roxicodone	oxycodone
66479058210	Roxicodone	oxycodone
66479058305	Roxicodone	oxycodone
66479058350	Roxicodone	oxycodone
66591081551	Methadone Hydrochloride	methadone
66591084141	Oramorph SR	morphine
66591084142	Oramorph SR	morphine
66591085142	Morphine Sulfate SR	morphine
66591087141	Oramorph SR	morphine
66591091332	Roxicodone	oxycodone
66591091391	Roxicodone	oxycodone
66591093141	Roxicodone	oxycodone
66647200142	FentaNYL	fentaNYL
66647200173	FentaNYL	fentaNYL
66647200394	FentaNYL Citrate	fentaNYL
66647201094	HYDROmorphone Hydrochloride	HYDROmorphone
66647201325	Hydromorphone	HYDROmorphone
66647204073	Morphine Sulfate	morphine
66647207142	FentaNYL Citrate	fentaNYL
66647214000	Morphine Sulfate	morphine
66647214033	Morphine Sulfate	morphine
66647217111	FentaNYL Citrate	fentaNYL
66647218133	Bupivacaine-FentaNYL	bupivacaine-fentaNYL
66647218211	Bupivacaine-FentaNYL	bupivacaine-fentaNYL
66647218533	Bupivacaine-FentaNYL	bupivacaine-fentaNYL
66647227100	FentaNYL Citrate	fentaNYL
66647237320	FentaNYL Citrate	fentaNYL
66689002150	Acetaminophen-Hydrocodone B	acetaminophen-hydrocodo
66689002304	Acetaminophen-HYDROcodone B	acetaminophen-HYDROcodo
66689002316	Acetaminophen-HYDROcodone B	acetaminophen-HYDROcodo
66689002350	Acetaminophen-HYDROcodone B	acetaminophen-HYDROcodo
66689002416	Oxycodone Hydrochloride	oxycodone
66689002450	Oxycodone Hydrochloride	oxycodone
66689002530	OxyCODONE Hydrochloride	oxyCODONE
66689003216	Morphine Sulfate	morphine
66689003250	Morphine Sulfate	morphine

66689003304	Morphine Sulfate	morphine
66689003350	Morphine Sulfate	morphine
66689040150	OxyCODONE Hydrochloride	oxyCODONE
66689040316	OxyCODONE Hydrochloride	oxyCODONE
66689069430	Methadone Hydrochloride	methadone
66689069479	Methadone Hydrochloride	methadone
66689071116	Methadone Hydrochloride	methadone
66689071216	Methadone Hydrochloride	methadone
66689089840	Methadone Hydrochloride	methadone
66992034010	Trezix	APAP/caffeine/dihydroco
67457021720	Methadone Hydrochloride	methadone
67767012018	Fentanyl	fentanyl
67767012118	Fentanyl	fentanyl
67767012218	Fentanyl	fentanyl
67767012318	Fentanyl	fentanyl
67877011601	Methadone Hydrochloride	methadone
68084004801	OxyCODONE Hydrochloride	oxyCODONE
68084010001	Acetaminophen-HYDROcodone B	acetaminophen-HYDROcodo
68084013901	Acetaminophen-Tramadol Hydr	acetaminophen-tramadol
68084014501	Acetaminophen-Hydrocodone B	acetaminophen-hydrocodo
68084015701	Morphine Sulfate ER	morphine
68084015801	Morphine Sulfate ER	morphine
68084018401	Oxycodone Hydrochloride	oxycodone
68084018501	Oxycodone Hydrochloride	oxycodone
68084022701	Hydrocodone-Ibuprofen	hydrocodone-ibuprofen
68084035301	Acetaminophen-Hydrocodone B	acetaminophen-hydrocodo
68084035401	Oxycodone Hydrochloride	oxycodone
68084035501	Acetaminophen-Oxycodone Hyd	acetaminophen-oxycodone
68084036201	Acetaminophen-Hydrocodone B	acetaminophen-hydrocodo
68084036801	Acetaminophen-Hydrocodone B	acetaminophen-hydrocodo
68084037201	Acetaminophen-Codeine Phosp	acetaminophen-codeine
68084037301	Acetaminophen-Codeine Phosp	acetaminophen-codeine
68084037801	Acetaminophen-Oxycodone Hyd	acetaminophen-oxycodone
68084037901	Acetaminophen-Oxycodone Hyd	acetaminophen-oxycodone
68084040301	Morphine Sulfate ER	morphine
68084040401	Morphine Sulfate ER	morphine
68084040501	Morphine Sulfate ER	morphine
68084040601	Morphine Sulfate ER	morphine
68084042301	Hydromorphone Hydrochloride	hydromorphone
68084047201	Hydromorphone Hydrochloride	hydromorphone
68084049601	Acetaminophen-TraMADol Hydr	acetaminophen-traMADol
68084060101	Acetaminophen-HYDROcodone B	acetaminophen-HYDROcodo
68084069901	Acetaminophen-OxyCODONE Hyd	acetaminophen-oxyCODONE
68084071001	Acetaminophen-OxyCODONE Hyd	acetaminophen-oxyCODONE
68084073801	Methadone Hydrochloride	methadone

68084079821	HYDROmorphone Hydrochloride	HYDROmorphone
68084080801	TraMADol Hydrochloride	traMADol
68084082501	Acetaminophen-TraMADol Hydr	acetaminophen-traMADol
68084084101	Hydrocodone-Ibuprofen	HYDROcodone-ibuprofen
68084086301	Acetaminophen-HYDROcodone B	acetaminophen-HYDROcodo
68084086309	Acetaminophen-HYDROcodone B	acetaminophen-HYDROcodo
68084088401	Acetaminophen-HYDROcodone B	acetaminophen-HYDROcodo
68084088409	Acetaminophen-HYDROcodone B	acetaminophen-HYDROcodo
68084089501	Acetaminophen-HYDROcodone B	acetaminophen-HYDROcodo
68084089509	Acetaminophen-HYDROcodone B	acetaminophen-HYDROcodo
68084096321	TraMADol Hydrochloride ER	traMADol
68094000162	Morphine Sulfate	morphine
68094071462	Acetaminophen-HYDROcodone B	acetaminophen-HYDROcodo
68094075458	Morphine Sulfate	morphine
68094075558	Morphine Sulfate	morphine
68094085261	HYDROmorphone Hydrochloride	HYDROmorphone
68180038306	TraMADol Hydrochloride ER	traMADol
68180069706	TraMADol Hydrochloride ER	traMADol
68308002003	OxyCODONE Hydrochloride	oxyCODONE
68308040547	Acetaminophen-OxyCODONE Hyd	acetaminophen-oxyCODONE
68308047547	Acetaminophen-HYDROcodone B	acetaminophen-HYDROcodo
68308048047	Acetaminophen-OxyCODONE Hyd	acetaminophen-oxyCODONE
68308050547	OxyCODONE Hydrochloride	oxyCODONE
68308084201	Acetaminophen-OxyCODONE Hyd	acetaminophen-oxyCODONE
68308084501	Aspirin-OxyCODONE	aspirin-oxyCODONE
68382031901	TraMADol Hydrochloride	traMADol
68382079401	OxyCODONE Hydrochloride	oxyCODONE
68382079601	OxyCODONE Hydrochloride	oxyCODONE
68382079701	OxyCODONE Hydrochloride	oxyCODONE
68462019301	Codeine Sulfate	codeine
68462020201	Morphine Sulfate	morphine
68462020301	Morphine Sulfate	morphine
68462020401	Oxycodone Hydrochloride	oxycodone
68462034737	Oxycodone Hydrochloride	oxycodone
68462034921	Morphine Sulfate	morphine
68552070101	Tramadol Hydrochloride	tramadol
68774016101	Oxycodone Hydrochloride ER	oxycodone
68774016201	Oxycodone Hydrochloride ER	oxycodone
68774016301	Oxycodone Hydrochloride ER	oxycodone
68774016401	Oxycodone Hydrochloride ER	oxycodone
76014000125	Hycet	acetaminophen-HYDROcodo
76045000410	Morphine Sulfate	morphine
76045000510	Morphine Sulfate	morphine
76045000610	Morphine Sulfate	morphine
76045000810	Morphine Sulfate	morphine

76329191101	Morphine Sulfate	morphine

Supplemental Table 2b: List of codes used to define opioid prescriptions[1]

NDC	Proprietary Name	Generic Name	Multum Drug ID
00591264101	APAP/Butalbital/Caffeine/Codeine	codeine	d03425
52544008201	Fioricet with Codeine	codeine	d03425
00143300001	APAP/Butalbital/Caffeine/Codeine	codeine	d03425
00247084530	Fioricet with Codeine	codeine	d03425
00591322001	APAP/Butalbital/Caffeine/Codeine	codeine	d03425
00603255321	APAP/Butalbital/Caffeine/Codeine	codeine	d03425
21695025828	APAP/Butalbital/Caffeine/Codeine	codeine	d03425
51991007301	APAP/Butalbital/Caffeine/Codeine	codeine	d03425
52544095801	Fioricet with Codeine	codeine	d03425
52959091260	APAP/Butalbital/Caffeine/Codeine	codeine	d03425
55887076940	APAP/Butalbital/Caffeine/Codeine	codeine	d03425
55887076960	APAP/Butalbital/Caffeine/Codeine	codeine	d03425
42195084010	APAP/Caffeine/Dihydrocodeine Bitartrate	dihydrocodeine	d04269
66992084010	Trezix	dihydrocodeine	d04269
68047072001	APAP/Caffeine/Dihydrocodeine Bitartrate	dihydrocodeine	d04269
00121050404	Acetaminophen-Codeine Phosphate	codeine	d03423
00121050405	Acetaminophen-Codeine Phosphate	codeine	d03423
00121050410	Acetaminophen-Codeine Phosphate	codeine	d03423
00121050412	Acetaminophen-Codeine Phosphate	codeine	d03423
00121050415	Acetaminophen-Codeine Phosphate	codeine	d03423
00121050416	Acetaminophen-Codeine Phosphate	codeine	d03423
00121100800	Acetaminophen-Codeine Phosphate	codeine	d03423
00121100812	Acetaminophen-Codeine Phosphate	codeine	d03423
00603102058	Acetaminophen-Codeine Phosphate	codeine	d03423
00603901354	Acetaminophen-Codeine Phosphate	codeine	d03423
00603901358	Acetaminophen-Codeine Phosphate	codeine	d03423
21695054704	Acetaminophen-Codeine Phosphate	codeine	d03423
23490796901	Acetaminophen-Codeine Phosphate	codeine	d03423
46672056116	Acetaminophen-Codeine Phosphate	codeine	d03423
50383007905	Acetaminophen-Codeine Phosphate	codeine	d03423
50383007906	Acetaminophen-Codeine Phosphate	codeine	d03423
50383007912	Acetaminophen-Codeine Phosphate	codeine	d03423
50383007913	Acetaminophen-Codeine Phosphate	codeine	d03423
50383007916	Acetaminophen-Codeine Phosphate	codeine	d03423
52959014103	Acetaminophen-Codeine Phosphate	codeine	d03423
52959014104	Acetaminophen-Codeine Phosphate	codeine	d03423
54569100100	Acetaminophen-Codeine Phosphate	codeine	d03423
54868037801	Acetaminophen-Codeine Phosphate	codeine	d03423
54868037802	Acetaminophen-Codeine Phosphate	codeine	d03423
60432024504	Acetaminophen-Codeine Phosphate	codeine	d03423
60432024516	Acetaminophen-Codeine Phosphate	codeine	d03423
66267099704	Acetaminophen-Codeine Phosphate	codeine	d03423

00187000301	Capital and Codeine Suspension	codeine	d03423
00093005001	Acetaminophen-Codeine Phosphate	codeine	d03423
00406048301	Acetaminophen-Codeine Phosphate	codeine	d03423
00603233721	Acetaminophen-Codeine Phosphate	codeine	d03423
13107005801	Acetaminophen-Codeine Phosphate	codeine	d03423
43063076315	Acetaminophen-Codeine Phosphate	codeine	d03423
54569031100	Acetaminophen-Codeine Phosphate	codeine	d03423
54569031102	Acetaminophen-Codeine Phosphate	codeine	d03423
55289044915	Acetaminophen-Codeine Phosphate	codeine	d03423
55289044930	Acetaminophen-Codeine Phosphate	codeine	d03423
63874025901	Acetaminophen-Codeine Phosphate	codeine	d03423
63874025903	Acetaminophen-Codeine Phosphate	codeine	d03423
63874025904	Acetaminophen-Codeine Phosphate	codeine	d03423
63874025912	Acetaminophen-Codeine Phosphate	codeine	d03423
63874025915	Acetaminophen-Codeine Phosphate	codeine	d03423
63874025920	Acetaminophen-Codeine Phosphate	codeine	d03423
63874025924	Acetaminophen-Codeine Phosphate	codeine	d03423
63874025930	Acetaminophen-Codeine Phosphate	codeine	d03423
63874025960	Acetaminophen-Codeine Phosphate	codeine	d03423
00093015001	Acetaminophen-Codeine Phosphate	codeine	d03423
00093015010	Acetaminophen-Codeine Phosphate	codeine	d03423
00247007845	Acetaminophen-Codeine Phosphate	codeine	d03423
00406048401	Acetaminophen-Codeine Phosphate	codeine	d03423
00406048403	Acetaminophen-Codeine Phosphate	codeine	d03423
00406048410	Acetaminophen-Codeine Phosphate	codeine	d03423
00406048420	Acetaminophen-Codeine Phosphate	codeine	d03423
00406048423	Acetaminophen-Codeine Phosphate	codeine	d03423
00406048450	Acetaminophen-Codeine Phosphate	codeine	d03423
00406048462	Acetaminophen-Codeine Phosphate	codeine	d03423
00603233802	Acetaminophen-Codeine Phosphate	codeine	d03423
00603233804	Acetaminophen-Codeine Phosphate	codeine	d03423
00603233816	Acetaminophen-Codeine Phosphate	codeine	d03423
00603233820	Acetaminophen-Codeine Phosphate	codeine	d03423
00603233821	Acetaminophen-Codeine Phosphate	codeine	d03423
00603233822	Acetaminophen-Codeine Phosphate	codeine	d03423
00603233828	Acetaminophen-Codeine Phosphate	codeine	d03423
00603233832	Acetaminophen-Codeine Phosphate	codeine	d03423
13107005901	Acetaminophen-Codeine Phosphate	codeine	d03423
13107005999	Acetaminophen-Codeine Phosphate	codeine	d03423
16590002315	Acetaminophen-Codeine Phosphate	codeine	d03423
21695024212	Acetaminophen-Codeine Phosphate	codeine	d03423
23490500400	Acetaminophen-Codeine Phosphate	codeine	d03423
23490500401	Acetaminophen-Codeine Phosphate	codeine	d03423
23490500402	Acetaminophen-Codeine Phosphate	codeine	d03423
23490500403	Acetaminophen-Codeine Phosphate	codeine	d03423

23490500404	Acetaminophen-Codeine Phosphate	codeine	d03423
23490500405	Acetaminophen-Codeine Phosphate	codeine	d03423
23490500406	Acetaminophen-Codeine Phosphate	codeine	d03423
23490500407	Acetaminophen-Codeine Phosphate	codeine	d03423
23490500408	Acetaminophen-Codeine Phosphate	codeine	d03423
23490500409	Acetaminophen-Codeine Phosphate	codeine	d03423
33358000101	Acetaminophen-Codeine Phosphate	codeine	d03423
43063001403	Acetaminophen-Codeine Phosphate	codeine	d03423
43063001404	Acetaminophen-Codeine Phosphate	codeine	d03423
43063001406	Acetaminophen-Codeine Phosphate	codeine	d03423
43063001412	Acetaminophen-Codeine Phosphate	codeine	d03423
43063055406	Acetaminophen-Codeine Phosphate	codeine	d03423
43063055410	Acetaminophen-Codeine Phosphate	codeine	d03423
43063055412	Acetaminophen-Codeine Phosphate	codeine	d03423
43063055415	Acetaminophen-Codeine Phosphate	codeine	d03423
43063055418	Acetaminophen-Codeine Phosphate	codeine	d03423
43063055420	Acetaminophen-Codeine Phosphate	codeine	d03423
43063055424	Acetaminophen-Codeine Phosphate	codeine	d03423
43063055425	Acetaminophen-Codeine Phosphate	codeine	d03423
43063055428	Acetaminophen-Codeine Phosphate	codeine	d03423
43063055430	Acetaminophen-Codeine Phosphate	codeine	d03423
43063055460	Acetaminophen-Codeine Phosphate	codeine	d03423
43063055490	Acetaminophen-Codeine Phosphate	codeine	d03423
43063055498	Acetaminophen-Codeine Phosphate	codeine	d03423
50458051360	Tylenol with Codeine #3	codeine	d03423
50458051380	Tylenol with Codeine #3	codeine	d03423
51079016101	Acetaminophen-Codeine Phosphate	codeine	d03423
51079016120	Acetaminophen-Codeine Phosphate	codeine	d03423
51079016121	Acetaminophen-Codeine Phosphate	codeine	d03423
51079016196	Acetaminophen-Codeine Phosphate	codeine	d03423
51079016199	Acetaminophen-Codeine Phosphate	codeine	d03423
52959000300	Acetaminophen-Codeine Phosphate	codeine	d03423
52959000302	Acetaminophen-Codeine Phosphate	codeine	d03423
52959000304	Acetaminophen-Codeine Phosphate	codeine	d03423
52959000306	Acetaminophen-Codeine Phosphate	codeine	d03423
52959000310	Acetaminophen-Codeine Phosphate	codeine	d03423
52959000312	Acetaminophen-Codeine Phosphate	codeine	d03423
52959000315	Acetaminophen-Codeine Phosphate	codeine	d03423
52959000316	Acetaminophen-Codeine Phosphate	codeine	d03423
52959000320	Acetaminophen-Codeine Phosphate	codeine	d03423
52959000324	Acetaminophen-Codeine Phosphate	codeine	d03423
52959000325	Acetaminophen-Codeine Phosphate	codeine	d03423
52959000328	Acetaminophen-Codeine Phosphate	codeine	d03423
52959000330	Acetaminophen-Codeine Phosphate	codeine	d03423
52959000340	Acetaminophen-Codeine Phosphate	codeine	d03423

52959000342	Acetaminophen-Codeine Phosphate	codeine	d03423
52959000345	Acetaminophen-Codeine Phosphate	codeine	d03423
52959000350	Acetaminophen-Codeine Phosphate	codeine	d03423
52959000360	Acetaminophen-Codeine Phosphate	codeine	d03423
52959000375	Acetaminophen-Codeine Phosphate	codeine	d03423
52959000384	Acetaminophen-Codeine Phosphate	codeine	d03423
52959000390	Acetaminophen-Codeine Phosphate	codeine	d03423
54569002402	Tylenol with Codeine #3	codeine	d03423
54569002500	Acetaminophen-Codeine Phosphate	codeine	d03423
54569002501	Acetaminophen-Codeine Phosphate	codeine	d03423
54569002502	Acetaminophen-Codeine Phosphate	codeine	d03423
54569002503	Acetaminophen-Codeine Phosphate	codeine	d03423
54569002504	Acetaminophen-Codeine Phosphate	codeine	d03423
54569002506	Acetaminophen-Codeine Phosphate	codeine	d03423
54569002507	Acetaminophen-Codeine Phosphate	codeine	d03423
54569002509	Acetaminophen-Codeine Phosphate	codeine	d03423
54569252301	Acetaminophen-Codeine Phosphate	codeine	d03423
54569252304	Acetaminophen-Codeine Phosphate	codeine	d03423
54569252306	Acetaminophen-Codeine Phosphate	codeine	d03423
54569252307	Acetaminophen-Codeine Phosphate	codeine	d03423
55289000506	Acetaminophen-Codeine Phosphate	codeine	d03423
55289000510	Acetaminophen-Codeine Phosphate	codeine	d03423
55289000512	Acetaminophen-Codeine Phosphate	codeine	d03423
55289000515	Acetaminophen-Codeine Phosphate	codeine	d03423
55289000518	Acetaminophen-Codeine Phosphate	codeine	d03423
55289000520	Acetaminophen-Codeine Phosphate	codeine	d03423
55289000524	Acetaminophen-Codeine Phosphate	codeine	d03423
55289000525	Acetaminophen-Codeine Phosphate	codeine	d03423
55289000530	Acetaminophen-Codeine Phosphate	codeine	d03423
55289000560	Acetaminophen-Codeine Phosphate	codeine	d03423
55289000590	Acetaminophen-Codeine Phosphate	codeine	d03423
55289000598	Acetaminophen-Codeine Phosphate	codeine	d03423
55289004815	Tylenol with Codeine #3	codeine	d03423
55887095150	Acetaminophen-Codeine Phosphate	codeine	d03423
58864000415	Acetaminophen-Codeine Phosphate	codeine	d03423
60429050060	Acetaminophen-Codeine Phosphate	codeine	d03423
60429050090	Acetaminophen-Codeine Phosphate	codeine	d03423
60760002215	Acetaminophen-Codeine Phosphate	codeine	d03423
60760002230	Acetaminophen-Codeine Phosphate	codeine	d03423
63304056201	Acetaminophen-Codeine Phosphate	codeine	d03423
63304056210	Acetaminophen-Codeine Phosphate	codeine	d03423
63739000410	Acetaminophen-Codeine Phosphate	codeine	d03423
63874020202	Acetaminophen-Codeine Phosphate	codeine	d03423
63874020205	Acetaminophen-Codeine Phosphate	codeine	d03423
64720030410	Acetaminophen-Codeine Phosphate	codeine	d03423

64720030411	Acetaminophen-Codeine Phosphate	codeine	d03423
64720030450	Acetaminophen-Codeine Phosphate	codeine	d03423
65162003311	Acetaminophen-Codeine Phosphate	codeine	d03423
66267075906	Acetaminophen-Codeine Phosphate	codeine	d03423
66336005930	Acetaminophen-Codeine Phosphate	codeine	d03423
68084037201	Acetaminophen-Codeine Phosphate	codeine	d03423
68084037211	Acetaminophen-Codeine Phosphate	codeine	d03423
00093035001	Acetaminophen-Codeine Phosphate	codeine	d03423
00093035005	Acetaminophen-Codeine Phosphate	codeine	d03423
00093035010	Acetaminophen-Codeine Phosphate	codeine	d03423
00406048501	Acetaminophen-Codeine Phosphate	codeine	d03423
00406048505	Acetaminophen-Codeine Phosphate	codeine	d03423
00603233921	Acetaminophen-Codeine Phosphate	codeine	d03423
00603233928	Acetaminophen-Codeine Phosphate	codeine	d03423
13107006001	Acetaminophen-Codeine Phosphate	codeine	d03423
13107006005	Acetaminophen-Codeine Phosphate	codeine	d03423
13107006099	Acetaminophen-Codeine Phosphate	codeine	d03423
21695024328	Acetaminophen-Codeine Phosphate	codeine	d03423
23490500500	Acetaminophen-Codeine Phosphate	codeine	d03423
23490500501	Acetaminophen-Codeine Phosphate	codeine	d03423
23490500502	Acetaminophen-Codeine Phosphate	codeine	d03423
23490500503	Acetaminophen-Codeine Phosphate	codeine	d03423
33358000260	Acetaminophen-Codeine Phosphate	codeine	d03423
49999002601	Acetaminophen-Codeine Phosphate	codeine	d03423
49999002630	Acetaminophen-Codeine Phosphate	codeine	d03423
49999002640	Acetaminophen-Codeine Phosphate	codeine	d03423
49999002660	Acetaminophen-Codeine Phosphate	codeine	d03423
49999002690	Acetaminophen-Codeine Phosphate	codeine	d03423
50458051560	Tylenol with Codeine #4	codeine	d03423
50458051570	Tylenol with Codeine #4	codeine	d03423
52959044602	Acetaminophen-Codeine Phosphate	codeine	d03423
52959044620	Acetaminophen-Codeine Phosphate	codeine	d03423
52959044650	Acetaminophen-Codeine Phosphate	codeine	d03423
52959044690	Acetaminophen-Codeine Phosphate	codeine	d03423
54569030201	Acetaminophen-Codeine Phosphate	codeine	d03423
54569030202	Acetaminophen-Codeine Phosphate	codeine	d03423
54569030208	Acetaminophen-Codeine Phosphate	codeine	d03423
55289091620	Acetaminophen-Codeine Phosphate	codeine	d03423
55289091630	Acetaminophen-Codeine Phosphate	codeine	d03423
55289091690	Acetaminophen-Codeine Phosphate	codeine	d03423
63304056101	Acetaminophen-Codeine Phosphate	codeine	d03423
63304056105	Acetaminophen-Codeine Phosphate	codeine	d03423
63874026701	Acetaminophen-Codeine Phosphate	codeine	d03423
63874026702	Acetaminophen-Codeine Phosphate	codeine	d03423
63874026703	Acetaminophen-Codeine Phosphate	codeine	d03423

63874026704	Acetaminophen-Codeine Phosphate	codeine	d03423
63874026705	Acetaminophen-Codeine Phosphate	codeine	d03423
63874026708	Acetaminophen-Codeine Phosphate	codeine	d03423
63874026710	Acetaminophen-Codeine Phosphate	codeine	d03423
63874026712	Acetaminophen-Codeine Phosphate	codeine	d03423
63874026715	Acetaminophen-Codeine Phosphate	codeine	d03423
63874026720	Acetaminophen-Codeine Phosphate	codeine	d03423
63874026728	Acetaminophen-Codeine Phosphate	codeine	d03423
63874026730	Acetaminophen-Codeine Phosphate	codeine	d03423
63874026740	Acetaminophen-Codeine Phosphate	codeine	d03423
63874026750	Acetaminophen-Codeine Phosphate	codeine	d03423
63874026760	Acetaminophen-Codeine Phosphate	codeine	d03423
63874026790	Acetaminophen-Codeine Phosphate	codeine	d03423
64720030510	Acetaminophen-Codeine Phosphate	codeine	d03423
68084037301	Acetaminophen-Codeine Phosphate	codeine	d03423
68084037311	Acetaminophen-Codeine Phosphate	codeine	d03423
00121477205	Acetaminophen-Hydrocodone Bitartrate	hydrocodone	d03428
00121477240	Acetaminophen-Hydrocodone Bitartrate	hydrocodone	d03428
00121465505	Acetaminophen-Hydrocodone Bitartrate	hydrocodone	d03428
00121154410	Acetaminophen-Hydrocodone Bitartrate	hydrocodone	d03428
00121154440	Acetaminophen-Hydrocodone Bitartrate	hydrocodone	d03428
00121477210	Acetaminophen-Hydrocodone Bitartrate	hydrocodone	d03428
00074305413	Vicodin HP	hydrocodone	d03428
00074305453	Vicodin HP	hydrocodone	d03428
00406037801	Acetaminophen-Hydrocodone Bitartrate	hydrocodone	d03428
00406037805	Acetaminophen-Hydrocodone Bitartrate	hydrocodone	d03428
00591217601	Acetaminophen-Hydrocodone Bitartrate	hydrocodone	d03428
00591217605	Acetaminophen-Hydrocodone Bitartrate	hydrocodone	d03428
27808011601	Acetaminophen-Hydrocodone Bitartrate	hydrocodone	d03428
27808011602	Acetaminophen-Hydrocodone Bitartrate	hydrocodone	d03428
43386035301	Acetaminophen-Hydrocodone Bitartrate	hydrocodone	d03428
43386035305	Acetaminophen-Hydrocodone Bitartrate	hydrocodone	d03428
59630091110	Xodol	hydrocodone	d03428
64376064301	Acetaminophen-Hydrocodone Bitartrate	hydrocodone	d03428
64376064305	Acetaminophen-Hydrocodone Bitartrate	hydrocodone	d03428
65162069610	Acetaminophen-Hydrocodone Bitartrate	hydrocodone	d03428
65162069650	Acetaminophen-Hydrocodone Bitartrate	hydrocodone	d03428
68453091110	Xodol	hydrocodone	d03428
00095067416	Zolvit	hydrocodone	d03428
00095909016	Lortab Elixir	hydrocodone	d03428
17478045016	Lortab	hydrocodone	d03428
59702067416	Zolvit	hydrocodone	d03428
00074304113	Vicodin	hydrocodone	d03428
00074304153	Vicodin	hydrocodone	d03428
00406037601	Acetaminophen-Hydrocodone Bitartrate	hydrocodone	d03428

00406037605	Acetaminophen-Hydrocodone Bitartrate	hydrocodone	d03428
00591217401	Acetaminophen-Hydrocodone Bitartrate	hydrocodone	d03428
00591217405	Acetaminophen-Hydrocodone Bitartrate	hydrocodone	d03428
27808011401	Acetaminophen-Hydrocodone Bitartrate	hydrocodone	d03428
27808011402	Acetaminophen-Hydrocodone Bitartrate	hydrocodone	d03428
43386035101	Acetaminophen-Hydrocodone Bitartrate	hydrocodone	d03428
43386035105	Acetaminophen-Hydrocodone Bitartrate	hydrocodone	d03428
59630091210	Xodol	hydrocodone	d03428
64376064801	Acetaminophen-Hydrocodone Bitartrate	hydrocodone	d03428
64376064805	Acetaminophen-Hydrocodone Bitartrate	hydrocodone	d03428
68084085225	Acetaminophen-Hydrocodone Bitartrate	hydrocodone	d03428
68084085295	Acetaminophen-Hydrocodone Bitartrate	hydrocodone	d03428
00074304313	Vicodin ES	hydrocodone	d03428
00074304353	Vicodin ES	hydrocodone	d03428
00406037701	Acetaminophen-Hydrocodone Bitartrate	hydrocodone	d03428
00406037705	Acetaminophen-Hydrocodone Bitartrate	hydrocodone	d03428
00591217501	Acetaminophen-Hydrocodone Bitartrate	hydrocodone	d03428
00591217505	Acetaminophen-Hydrocodone Bitartrate	hydrocodone	d03428
27808011501	Acetaminophen-Hydrocodone Bitartrate	hydrocodone	d03428
27808011502	Acetaminophen-Hydrocodone Bitartrate	hydrocodone	d03428
43386035201	Acetaminophen-Hydrocodone Bitartrate	hydrocodone	d03428
43386035205	Acetaminophen-Hydrocodone Bitartrate	hydrocodone	d03428
59630091310	Xodol	hydrocodone	d03428
64376064901	Acetaminophen-Hydrocodone Bitartrate	hydrocodone	d03428
64376064905	Acetaminophen-Hydrocodone Bitartrate	hydrocodone	d03428
68084099011	Acetaminophen-Hydrocodone Bitartrate	hydrocodone	d03428
68084099021	Acetaminophen-Hydrocodone Bitartrate	hydrocodone	d03428
68453091310	Xodol	hydrocodone	d03428
00406012501	Acetaminophen-Hydrocodone Bitartrate	hydrocodone	d03428
00406012505	Acetaminophen-Hydrocodone Bitartrate	hydrocodone	d03428
00406012510	Acetaminophen-Hydrocodone Bitartrate	hydrocodone	d03428
00406012512	Acetaminophen-Hydrocodone Bitartrate	hydrocodone	d03428
00406012523	Acetaminophen-Hydrocodone Bitartrate	hydrocodone	d03428
00406012562	Acetaminophen-Hydrocodone Bitartrate	hydrocodone	d03428
00406036701	Acetaminophen-Hydrocodone Bitartrate	hydrocodone	d03428
00406036705	Acetaminophen-Hydrocodone Bitartrate	hydrocodone	d03428
00406036723	Acetaminophen-Hydrocodone Bitartrate	hydrocodone	d03428
00406036762	Acetaminophen-Hydrocodone Bitartrate	hydrocodone	d03428
00591085301	Acetaminophen-Hydrocodone Bitartrate	hydrocodone	d03428
00591085305	Acetaminophen-Hydrocodone Bitartrate	hydrocodone	d03428
00591261201	Acetaminophen-Hydrocodone Bitartrate	hydrocodone	d03428
00591261205	Acetaminophen-Hydrocodone Bitartrate	hydrocodone	d03428
00603360921	Acetaminophen-Hydrocodone Bitartrate	hydrocodone	d03428
00603388702	Acetaminophen-Hydrocodone Bitartrate	hydrocodone	d03428
00603388704	Acetaminophen-Hydrocodone Bitartrate	hydrocodone	d03428

00603388712	Acetaminophen-Hydrocodone Bitartrate	hydrocodone	d03428
00603388720	Acetaminophen-Hydrocodone Bitartrate	hydrocodone	d03428
00603388721	Acetaminophen-Hydrocodone Bitartrate	hydrocodone	d03428
00603388722	Acetaminophen-Hydrocodone Bitartrate	hydrocodone	d03428
00603388726	Acetaminophen-Hydrocodone Bitartrate	hydrocodone	d03428
00603388728	Acetaminophen-Hydrocodone Bitartrate	hydrocodone	d03428
00603388732	Acetaminophen-Hydrocodone Bitartrate	hydrocodone	d03428
00904642161	Acetaminophen-Hydrocodone Bitartrate	hydrocodone	d03428
13107002101	Acetaminophen-Hydrocodone Bitartrate	hydrocodone	d03428
13107002105	Acetaminophen-Hydrocodone Bitartrate	hydrocodone	d03428
13107002199	Acetaminophen-Hydrocodone Bitartrate	hydrocodone	d03428
16590011830	Acetaminophen-Hydrocodone Bitartrate	hydrocodone	d03428
21695027200	Acetaminophen-Hydrocodone Bitartrate	hydrocodone	d03428
23490705300	Acetaminophen-Hydrocodone Bitartrate	hydrocodone	d03428
23490705301	Acetaminophen-Hydrocodone Bitartrate	hydrocodone	d03428
23490705302	Acetaminophen-Hydrocodone Bitartrate	hydrocodone	d03428
23490705303	Acetaminophen-Hydrocodone Bitartrate	hydrocodone	d03428
23490705304	Acetaminophen-Hydrocodone Bitartrate	hydrocodone	d03428
23490705305	Acetaminophen-Hydrocodone Bitartrate	hydrocodone	d03428
27808003701	Acetaminophen-Hydrocodone Bitartrate	hydrocodone	d03428
27808003702	Acetaminophen-Hydrocodone Bitartrate	hydrocodone	d03428
27808003703	Acetaminophen-Hydrocodone Bitartrate	hydrocodone	d03428
33358017001	Acetaminophen-Hydrocodone Bitartrate	hydrocodone	d03428
42291033401	Acetaminophen-Hydrocodone Bitartrate	hydrocodone	d03428
42858020301	Acetaminophen-Hydrocodone Bitartrate	hydrocodone	d03428
42858020350	Acetaminophen-Hydrocodone Bitartrate	hydrocodone	d03428
43063033901	Acetaminophen-Hydrocodone Bitartrate	hydrocodone	d03428
43063033920	Acetaminophen-Hydrocodone Bitartrate	hydrocodone	d03428
43063033930	Acetaminophen-Hydrocodone Bitartrate	hydrocodone	d03428
43063033960	Acetaminophen-Hydrocodone Bitartrate	hydrocodone	d03428
43063033990	Acetaminophen-Hydrocodone Bitartrate	hydrocodone	d03428
43063033998	Acetaminophen-Hydrocodone Bitartrate	hydrocodone	d03428
43063074906	Acetaminophen-Hydrocodone Bitartrate	hydrocodone	d03428
43063074915	Acetaminophen-Hydrocodone Bitartrate	hydrocodone	d03428
43063074920	Acetaminophen-Hydrocodone Bitartrate	hydrocodone	d03428
43063074921	Acetaminophen-Hydrocodone Bitartrate	hydrocodone	d03428
43063074930	Acetaminophen-Hydrocodone Bitartrate	hydrocodone	d03428
43063074940	Acetaminophen-Hydrocodone Bitartrate	hydrocodone	d03428
43063074956	Acetaminophen-Hydrocodone Bitartrate	hydrocodone	d03428
43063074960	Acetaminophen-Hydrocodone Bitartrate	hydrocodone	d03428
43063074984	Acetaminophen-Hydrocodone Bitartrate	hydrocodone	d03428
43063074988	Acetaminophen-Hydrocodone Bitartrate	hydrocodone	d03428
43063074990	Acetaminophen-Hydrocodone Bitartrate	hydrocodone	d03428
43063074993	Acetaminophen-Hydrocodone Bitartrate	hydrocodone	d03428
43063074998	Acetaminophen-Hydrocodone Bitartrate	hydrocodone	d03428

43353015930	Acetaminophen-Hydrocodone Bitartrate	hydrocodone	d03428
43353015960	Acetaminophen-Hydrocodone Bitartrate	hydrocodone	d03428
49999001920	Norco	hydrocodone	d03428
49999001930	Norco	hydrocodone	d03428
49999016900	Acetaminophen-Hydrocodone Bitartrate	hydrocodone	d03428
49999016901	Acetaminophen-Hydrocodone Bitartrate	hydrocodone	d03428
49999016918	Acetaminophen-Hydrocodone Bitartrate	hydrocodone	d03428
49999016920	Acetaminophen-Hydrocodone Bitartrate	hydrocodone	d03428
49999016930	Acetaminophen-Hydrocodone Bitartrate	hydrocodone	d03428
49999016950	Acetaminophen-Hydrocodone Bitartrate	hydrocodone	d03428
49999016960	Acetaminophen-Hydrocodone Bitartrate	hydrocodone	d03428
49999016990	Acetaminophen-Hydrocodone Bitartrate	hydrocodone	d03428
50268040215	Acetaminophen-Hydrocodone Bitartrate	hydrocodone	d03428
50268040815	Acetaminophen-Hydrocodone Bitartrate	hydrocodone	d03428
50474093201	Lortab 10/325	hydrocodone	d03428
51079077901	Acetaminophen-Hydrocodone Bitartrate	hydrocodone	d03428
51079077920	Acetaminophen-Hydrocodone Bitartrate	hydrocodone	d03428
51079077921	Acetaminophen-Hydrocodone Bitartrate	hydrocodone	d03428
51862021701	Acetaminophen-Hydrocodone Bitartrate	hydrocodone	d03428
51862021705	Acetaminophen-Hydrocodone Bitartrate	hydrocodone	d03428
51862022901	Acetaminophen-Hydrocodone Bitartrate	hydrocodone	d03428
51862022905	Acetaminophen-Hydrocodone Bitartrate	hydrocodone	d03428
51862058701	Lorcet HD	hydrocodone	d03428
51862058705	Lorcet HD	hydrocodone	d03428
52544016101	Norco	hydrocodone	d03428
52544016105	Norco	hydrocodone	d03428
52544053901	Norco	hydrocodone	d03428
52544053905	Norco	hydrocodone	d03428
52959032400	Acetaminophen-Hydrocodone Bitartrate	hydrocodone	d03428
52959032401	Acetaminophen-Hydrocodone Bitartrate	hydrocodone	d03428
52959032402	Acetaminophen-Hydrocodone Bitartrate	hydrocodone	d03428
52959032403	Acetaminophen-Hydrocodone Bitartrate	hydrocodone	d03428
52959032405	Acetaminophen-Hydrocodone Bitartrate	hydrocodone	d03428
52959032406	Acetaminophen-Hydrocodone Bitartrate	hydrocodone	d03428
52959032408	Acetaminophen-Hydrocodone Bitartrate	hydrocodone	d03428
52959032410	Acetaminophen-Hydrocodone Bitartrate	hydrocodone	d03428
52959032415	Acetaminophen-Hydrocodone Bitartrate	hydrocodone	d03428
52959032420	Acetaminophen-Hydrocodone Bitartrate	hydrocodone	d03428
52959032424	Acetaminophen-Hydrocodone Bitartrate	hydrocodone	d03428
52959032425	Acetaminophen-Hydrocodone Bitartrate	hydrocodone	d03428
52959032428	Acetaminophen-Hydrocodone Bitartrate	hydrocodone	d03428
52959032430	Acetaminophen-Hydrocodone Bitartrate	hydrocodone	d03428
52959032440	Acetaminophen-Hydrocodone Bitartrate	hydrocodone	d03428
52959032442	Acetaminophen-Hydrocodone Bitartrate	hydrocodone	d03428
52959032445	Acetaminophen-Hydrocodone Bitartrate	hydrocodone	d03428

52959032450	Acetaminophen-Hydrocodone Bitartrate	hydrocodone	d03428
52959032460	Acetaminophen-Hydrocodone Bitartrate	hydrocodone	d03428
52959032475	Acetaminophen-Hydrocodone Bitartrate	hydrocodone	d03428
52959032484	Acetaminophen-Hydrocodone Bitartrate	hydrocodone	d03428
52959032490	Acetaminophen-Hydrocodone Bitartrate	hydrocodone	d03428
52959053324	Norco	hydrocodone	d03428
52959053330	Norco	hydrocodone	d03428
52959053340	Norco	hydrocodone	d03428
53746011001	Acetaminophen-Hydrocodone Bitartrate	hydrocodone	d03428
53746011005	Acetaminophen-Hydrocodone Bitartrate	hydrocodone	d03428
53746011010	Acetaminophen-Hydrocodone Bitartrate	hydrocodone	d03428
54569524000	Acetaminophen-Hydrocodone Bitartrate	hydrocodone	d03428
54569524001	Acetaminophen-Hydrocodone Bitartrate	hydrocodone	d03428
54569524002	Acetaminophen-Hydrocodone Bitartrate	hydrocodone	d03428
54569524004	Acetaminophen-Hydrocodone Bitartrate	hydrocodone	d03428
54569524005	Acetaminophen-Hydrocodone Bitartrate	hydrocodone	d03428
54569524006	Acetaminophen-Hydrocodone Bitartrate	hydrocodone	d03428
54569524008	Acetaminophen-Hydrocodone Bitartrate	hydrocodone	d03428
55289065130	Norco	hydrocodone	d03428
55289073706	Acetaminophen-Hydrocodone Bitartrate	hydrocodone	d03428
55289073715	Acetaminophen-Hydrocodone Bitartrate	hydrocodone	d03428
55289073720	Acetaminophen-Hydrocodone Bitartrate	hydrocodone	d03428
55289073721	Acetaminophen-Hydrocodone Bitartrate	hydrocodone	d03428
55289073730	Acetaminophen-Hydrocodone Bitartrate	hydrocodone	d03428
55289073740	Acetaminophen-Hydrocodone Bitartrate	hydrocodone	d03428
55289073756	Acetaminophen-Hydrocodone Bitartrate	hydrocodone	d03428
55289073760	Acetaminophen-Hydrocodone Bitartrate	hydrocodone	d03428
55289073784	Acetaminophen-Hydrocodone Bitartrate	hydrocodone	d03428
55289073788	Acetaminophen-Hydrocodone Bitartrate	hydrocodone	d03428
55289073790	Acetaminophen-Hydrocodone Bitartrate	hydrocodone	d03428
55289073793	Acetaminophen-Hydrocodone Bitartrate	hydrocodone	d03428
55289073798	Acetaminophen-Hydrocodone Bitartrate	hydrocodone	d03428
57664017613	Acetaminophen-Hydrocodone Bitartrate	hydrocodone	d03428
57664017688	Acetaminophen-Hydrocodone Bitartrate	hydrocodone	d03428
60429057412	Acetaminophen-Hydrocodone Bitartrate	hydrocodone	d03428
60429057418	Acetaminophen-Hydrocodone Bitartrate	hydrocodone	d03428
60429057460	Acetaminophen-Hydrocodone Bitartrate	hydrocodone	d03428
60429057490	Acetaminophen-Hydrocodone Bitartrate	hydrocodone	d03428
60760038815	Acetaminophen-Hydrocodone Bitartrate	hydrocodone	d03428
60760038830	Acetaminophen-Hydrocodone Bitartrate	hydrocodone	d03428
60760038860	Acetaminophen-Hydrocodone Bitartrate	hydrocodone	d03428
60760038890	Acetaminophen-Hydrocodone Bitartrate	hydrocodone	d03428
68084010001	Acetaminophen-Hydrocodone Bitartrate	hydrocodone	d03428
68084035301	Acetaminophen-Hydrocodone Bitartrate	hydrocodone	d03428
68084035311	Acetaminophen-Hydrocodone Bitartrate	hydrocodone	d03428

68084088401	Acetaminophen-Hydrocodone Bitartrate	hydrocodone	d03428
68084088409	Acetaminophen-Hydrocodone Bitartrate	hydrocodone	d03428
68084088411	Acetaminophen-Hydrocodone Bitartrate	hydrocodone	d03428
68308022301	Lorcet HD	hydrocodone	d03428
68308022601	Lorcet HD	hydrocodone	d03428
68387023512	Acetaminophen-Hydrocodone Bitartrate	hydrocodone	d03428
00121477107	Acetaminophen-Hydrocodone Bitartrate	hydrocodone	d03428
00121477115	Acetaminophen-Hydrocodone Bitartrate	hydrocodone	d03428
00486077116	Zamicet	hydrocodone	d03428
63717089516	Zamicet	hydrocodone	d03428
00591217101	Acetaminophen-Hydrocodone Bitartrate	hydrocodone	d03428
13811065710	Acetaminophen-Hydrocodone Bitartrate	hydrocodone	d03428
68025006910	Verdrocet	hydrocodone	d03428
00406012301	Acetaminophen-Hydrocodone Bitartrate	hydrocodone	d03428
00406012305	Acetaminophen-Hydrocodone Bitartrate	hydrocodone	d03428
00406012310	Acetaminophen-Hydrocodone Bitartrate	hydrocodone	d03428
00406012312	Acetaminophen-Hydrocodone Bitartrate	hydrocodone	d03428
00406012323	Acetaminophen-Hydrocodone Bitartrate	hydrocodone	d03428
00406012362	Acetaminophen-Hydrocodone Bitartrate	hydrocodone	d03428
00406036501	Acetaminophen-Hydrocodone Bitartrate	hydrocodone	d03428
00406036505	Acetaminophen-Hydrocodone Bitartrate	hydrocodone	d03428
00406036523	Acetaminophen-Hydrocodone Bitartrate	Hydrocodone	d03428
00406036562	Acetaminophen-Hydrocodone Bitartrate	Hydrocodone	d03428
00591217201	Acetaminophen-Hydrocodone Bitartrate	hydrocodone	d03428
00591217205	Acetaminophen-Hydrocodone Bitartrate	hydrocodone	d03428
00591320201	Acetaminophen-Hydrocodone Bitartrate	hydrocodone	d03428
00591320205	Acetaminophen-Hydrocodone Bitartrate	hydrocodone	d03428
00603389002	Acetaminophen-Hydrocodone Bitartrate	hydrocodone	d03428
00603389004	Acetaminophen-Hydrocodone Bitartrate	hydrocodone	d03428
00603389016	Acetaminophen-Hydrocodone Bitartrate	hydrocodone	d03428
00603389020	Acetaminophen-Hydrocodone Bitartrate	hydrocodone	d03428
00603389021	Acetaminophen-Hydrocodone Bitartrate	hydrocodone	d03428
00603389022	Acetaminophen-Hydrocodone Bitartrate	hydrocodone	d03428
00603389028	Acetaminophen-Hydrocodone Bitartrate	hydrocodone	d03428
00603389032	Acetaminophen-Hydrocodone Bitartrate	hydrocodone	d03428
00904641961	Acetaminophen-Hydrocodone Bitartrate	hydrocodone	d03428
00904656761	Acetaminophen-Hydrocodone Bitartrate	hydrocodone	d03428
13107001901	Acetaminophen-Hydrocodone Bitartrate	hydrocodone	d03428
13107001905	Acetaminophen-Hydrocodone Bitartrate	hydrocodone	d03428
21695026828	Acetaminophen-Hydrocodone Bitartrate	hydrocodone	d03428
23490748700	Acetaminophen-Hydrocodone Bitartrate	hydrocodone	d03428
23490748701	Acetaminophen-Hydrocodone Bitartrate	hydrocodone	d03428
23490748702	Acetaminophen-Hydrocodone Bitartrate	hydrocodone	d03428
23490748703	Acetaminophen-Hydrocodone Bitartrate	hydrocodone	d03428
23490748709	Acetaminophen-Hydrocodone Bitartrate	hydrocodone	d03428

27808003501	Acetaminophen-Hydrocodone Bitartrate	hydrocodone	d03428
27808003502	Acetaminophen-Hydrocodone Bitartrate	hydrocodone	d03428
27808003503	Acetaminophen-Hydrocodone Bitartrate	hydrocodone	d03428
42291033201	Acetaminophen-Hydrocodone Bitartrate	hydrocodone	d03428
42858020101	Acetaminophen-Hydrocodone Bitartrate	hydrocodone	d03428
42858020150	Acetaminophen-Hydrocodone Bitartrate	hydrocodone	d03428
43063009102	Acetaminophen-Hydrocodone Bitartrate	hydrocodone	d03428
43063009104	Acetaminophen-Hydrocodone Bitartrate	hydrocodone	d03428
43063009106	Acetaminophen-Hydrocodone Bitartrate	hydrocodone	d03428
43063009120	Acetaminophen-Hydrocodone Bitartrate	hydrocodone	d03428
43063068610	Acetaminophen-Hydrocodone Bitartrate	hydrocodone	d03428
43063068612	Acetaminophen-Hydrocodone Bitartrate	hydrocodone	d03428
43063068615	Acetaminophen-Hydrocodone Bitartrate	hydrocodone	d03428
43063068620	Acetaminophen-Hydrocodone Bitartrate	hydrocodone	d03428
43063068630	Acetaminophen-Hydrocodone Bitartrate	hydrocodone	d03428
43063068640	Acetaminophen-Hydrocodone Bitartrate	hydrocodone	d03428
43063068656	Acetaminophen-Hydrocodone Bitartrate	hydrocodone	d03428
43063068660	Acetaminophen-Hydrocodone Bitartrate	hydrocodone	d03428
43063068684	Acetaminophen-Hydrocodone Bitartrate	hydrocodone	d03428
43063068688	Acetaminophen-Hydrocodone Bitartrate	hydrocodone	d03428
43063068690	Acetaminophen-Hydrocodone Bitartrate	hydrocodone	d03428
43063068693	Acetaminophen-Hydrocodone Bitartrate	hydrocodone	d03428
43063068698	Acetaminophen-Hydrocodone Bitartrate	hydrocodone	d03428
43386035601	Acetaminophen-Hydrocodone Bitartrate	hydrocodone	d03428
49999060801	Acetaminophen-Hydrocodone Bitartrate	hydrocodone	d03428
49999060830	Acetaminophen-Hydrocodone Bitartrate	hydrocodone	d03428
49999060860	Acetaminophen-Hydrocodone Bitartrate	hydrocodone	d03428
49999060890	Acetaminophen-Hydrocodone Bitartrate	hydrocodone	d03428
50268040115	Acetaminophen-Hydrocodone Bitartrate	hydrocodone	d03428
50268040315	Acetaminophen-Hydrocodone Bitartrate	hydrocodone	d03428
50474093001	Lortab 5/325	hydrocodone	d03428
51079077701	Acetaminophen-Hydrocodone Bitartrate	hydrocodone	d03428
51079077720	Acetaminophen-Hydrocodone Bitartrate	hydrocodone	d03428
51079077721	Acetaminophen-Hydrocodone Bitartrate	hydrocodone	d03428
51862021501	Acetaminophen-Hydrocodone Bitartrate	hydrocodone	d03428
51862021505	Acetaminophen-Hydrocodone Bitartrate	hydrocodone	d03428
51862022701	Acetaminophen-Hydrocodone Bitartrate	hydrocodone	d03428
51862022705	Acetaminophen-Hydrocodone Bitartrate	hydrocodone	d03428
51862058501	Lorcet	hydrocodone	d03428
51862058505	Lorcet	hydrocodone	d03428
52544007101	Norco	hydrocodone	d03428
52544091301	Norco	hydrocodone	d03428
52959073702	Acetaminophen-Hydrocodone Bitartrate	hydrocodone	d03428
52959073715	Acetaminophen-Hydrocodone Bitartrate	hydrocodone	d03428
52959073720	Acetaminophen-Hydrocodone Bitartrate	hydrocodone	d03428

52959073725	Acetaminophen-Hydrocodone Bitartrate	hydrocodone	d03428
52959073728	Acetaminophen-Hydrocodone Bitartrate	hydrocodone	d03428
52959073730	Acetaminophen-Hydrocodone Bitartrate	hydrocodone	d03428
52959073740	Acetaminophen-Hydrocodone Bitartrate	hydrocodone	d03428
52959073742	Acetaminophen-Hydrocodone Bitartrate	hydrocodone	d03428
52959073745	Acetaminophen-Hydrocodone Bitartrate	hydrocodone	d03428
52959073750	Acetaminophen-Hydrocodone Bitartrate	hydrocodone	d03428
52959073760	Acetaminophen-Hydrocodone Bitartrate	hydrocodone	d03428
52959073790	Acetaminophen-Hydrocodone Bitartrate	hydrocodone	d03428
53746010901	Acetaminophen-Hydrocodone Bitartrate	hydrocodone	d03428
53746010905	Acetaminophen-Hydrocodone Bitartrate	hydrocodone	d03428
53746010910	Acetaminophen-Hydrocodone Bitartrate	hydrocodone	d03428
54569552300	Acetaminophen-Hydrocodone Bitartrate	hydrocodone	d03428
54569552302	Acetaminophen-Hydrocodone Bitartrate	hydrocodone	d03428
54569552303	Acetaminophen-Hydrocodone Bitartrate	hydrocodone	d03428
54569552304	Acetaminophen-Hydrocodone Bitartrate	hydrocodone	d03428
55289089415	Norco	hydrocodone	d03428
55289099702	Acetaminophen-Hydrocodone Bitartrate	hydrocodone	d03428
55289099704	Acetaminophen-Hydrocodone Bitartrate	hydrocodone	d03428
55289099706	Acetaminophen-Hydrocodone Bitartrate	hydrocodone	d03428
55289099712	Acetaminophen-Hydrocodone Bitartrate	hydrocodone	d03428
55289099715	Acetaminophen-Hydrocodone Bitartrate	hydrocodone	d03428
55289099730	Acetaminophen-Hydrocodone Bitartrate	hydrocodone	d03428
55289099756	Acetaminophen-Hydrocodone Bitartrate	hydrocodone	d03428
55289099784	Acetaminophen-Hydrocodone Bitartrate	hydrocodone	d03428
55289099788	Acetaminophen-Hydrocodone Bitartrate	hydrocodone	d03428
55289099790	Acetaminophen-Hydrocodone Bitartrate	hydrocodone	d03428
55887029501	Acetaminophen-Hydrocodone Bitartrate	hydrocodone	d03428
57664012613	Acetaminophen-Hydrocodone Bitartrate	hydrocodone	d03428
57664012688	Acetaminophen-Hydrocodone Bitartrate	hydrocodone	d03428
60429057201	Acetaminophen-Hydrocodone Bitartrate	hydrocodone	d03428
60429057205	Acetaminophen-Hydrocodone Bitartrate	hydrocodone	d03428
60429057212	Acetaminophen-Hydrocodone Bitartrate	hydrocodone	d03428
60429057218	Acetaminophen-Hydrocodone Bitartrate	hydrocodone	d03428
60429057230	Acetaminophen-Hydrocodone Bitartrate	hydrocodone	d03428
60429057260	Acetaminophen-Hydrocodone Bitartrate	hydrocodone	d03428
60429057290	Acetaminophen-Hydrocodone Bitartrate	hydrocodone	d03428
63739032610	Acetaminophen-Hydrocodone Bitartrate	hydrocodone	d03428
63739053210	Acetaminophen-Hydrocodone Bitartrate	hydrocodone	d03428
63739070410	Acetaminophen-Hydrocodone Bitartrate	hydrocodone	d03428
68084036801	Acetaminophen-Hydrocodone Bitartrate	hydrocodone	d03428
68084036811	Acetaminophen-Hydrocodone Bitartrate	hydrocodone	d03428
68084089501	Acetaminophen-Hydrocodone Bitartrate	hydrocodone	d03428
68084089509	Acetaminophen-Hydrocodone Bitartrate	hydrocodone	d03428
68084089511	Acetaminophen-Hydrocodone Bitartrate	hydrocodone	d03428

68308022101	Lorcet	hydrocodone	d03428
68308022401	Lorcet	hydrocodone	d03428
00406012401	Acetaminophen-Hydrocodone Bitartrate	hydrocodone	d03428
00406012405	Acetaminophen-Hydrocodone Bitartrate	hydrocodone	d03428
00406012410	Acetaminophen-Hydrocodone Bitartrate	hydrocodone	d03428
00406012412	Acetaminophen-Hydrocodone Bitartrate	hydrocodone	d03428
00406012423	Acetaminophen-Hydrocodone Bitartrate	hydrocodone	d03428
00406012462	Acetaminophen-Hydrocodone Bitartrate	hydrocodone	d03428
00406036601	Acetaminophen-Hydrocodone Bitartrate	hydrocodone	d03428
00406036605	Acetaminophen-Hydrocodone Bitartrate	hydrocodone	d03428
00406036623	Acetaminophen-Hydrocodone Bitartrate	hydrocodone	d03428
00406036662	Acetaminophen-Hydrocodone Bitartrate	hydrocodone	d03428
00591260501	Acetaminophen-Hydrocodone Bitartrate	hydrocodone	d03428
00591260505	Acetaminophen-Hydrocodone Bitartrate	hydrocodone	d03428
00591320301	Acetaminophen-Hydrocodone Bitartrate	hydrocodone	d03428
00603389102	Acetaminophen-Hydrocodone Bitartrate	hydrocodone	d03428
00603389121	Acetaminophen-Hydrocodone Bitartrate	hydrocodone	d03428
00603389122	Acetaminophen-Hydrocodone Bitartrate	hydrocodone	d03428
00603389128	Acetaminophen-Hydrocodone Bitartrate	hydrocodone	d03428
00603389132	Acetaminophen-Hydrocodone Bitartrate	hydrocodone	d03428
00904642061	Acetaminophen-Hydrocodone Bitartrate	hydrocodone	d03428
13107002001	Acetaminophen-Hydrocodone Bitartrate	hydrocodone	d03428
13107002005	Acetaminophen-Hydrocodone Bitartrate	hydrocodone	d03428
21695038610	Acetaminophen-Hydrocodone Bitartrate	hydrocodone	d03428
23490748603	Acetaminophen-Hydrocodone Bitartrate	hydrocodone	d03428
23490748604	Acetaminophen-Hydrocodone Bitartrate	hydrocodone	d03428
23490748607	Acetaminophen-Hydrocodone Bitartrate	hydrocodone	d03428
23490748609	Acetaminophen-Hydrocodone Bitartrate	hydrocodone	d03428
27808003601	Acetaminophen-Hydrocodone Bitartrate	hydrocodone	d03428
27808003602	Acetaminophen-Hydrocodone Bitartrate	hydrocodone	d03428
27808003603	Acetaminophen-Hydrocodone Bitartrate	hydrocodone	d03428
42291033301	Acetaminophen-Hydrocodone Bitartrate	hydrocodone	d03428
42858020201	Acetaminophen-Hydrocodone Bitartrate	hydrocodone	d03428
42858020250	Acetaminophen-Hydrocodone Bitartrate	hydrocodone	d03428
43063026506	Acetaminophen-Hydrocodone Bitartrate	hydrocodone	d03428
43063068506	Acetaminophen-Hydrocodone Bitartrate	hydrocodone	d03428
43063068512	Acetaminophen-Hydrocodone Bitartrate	hydrocodone	d03428
43063068515	Acetaminophen-Hydrocodone Bitartrate	hydrocodone	d03428
43063068520	Acetaminophen-Hydrocodone Bitartrate	hydrocodone	d03428
43063068530	Acetaminophen-Hydrocodone Bitartrate	hydrocodone	d03428
43063068560	Acetaminophen-Hydrocodone Bitartrate	hydrocodone	d03428
43386035701	Acetaminophen-Hydrocodone Bitartrate	hydrocodone	d03428
43547037310	Acetaminophen-Hydrocodone Bitartrate	hydrocodone	d03428
49999060901	Acetaminophen-Hydrocodone Bitartrate	hydrocodone	d03428
49999060930	Acetaminophen-Hydrocodone Bitartrate	hydrocodone	d03428

49999060960	Acetaminophen-Hydrocodone Bitartrate	hydrocodone	d03428
49999060990	Acetaminophen-Hydrocodone Bitartrate	hydrocodone	d03428
50268040015	Acetaminophen-Hydrocodone Bitartrate	hydrocodone	d03428
50474093101	Lortab 7.5/325	hydrocodone	d03428
51079077801	Acetaminophen-Hydrocodone Bitartrate	hydrocodone	d03428
51079077820	Acetaminophen-Hydrocodone Bitartrate	hydrocodone	d03428
51079077821	Acetaminophen-Hydrocodone Bitartrate	hydrocodone	d03428
51862021601	Acetaminophen-Hydrocodone Bitartrate	hydrocodone	d03428
51862021605	Acetaminophen-Hydrocodone Bitartrate	hydrocodone	d03428
51862022801	Acetaminophen-Hydrocodone Bitartrate	hydrocodone	d03428
51862022805	Acetaminophen-Hydrocodone Bitartrate	hydrocodone	d03428
51862058601	Lorcet Plus	hydrocodone	d03428
51862058605	Lorcet Plus	hydrocodone	d03428
52544016201	Norco	hydrocodone	d03428
52544072901	Norco	hydrocodone	d03428
52959073500	Acetaminophen-Hydrocodone Bitartrate	hydrocodone	d03428
52959073502	Acetaminophen-Hydrocodone Bitartrate	hydrocodone	d03428
52959073503	Acetaminophen-Hydrocodone Bitartrate	hydrocodone	d03428
52959073530	Acetaminophen-Hydrocodone Bitartrate	hydrocodone	d03428
52959073540	Acetaminophen-Hydrocodone Bitartrate	hydrocodone	d03428
52959073550	Acetaminophen-Hydrocodone Bitartrate	hydrocodone	d03428
52959073560	Acetaminophen-Hydrocodone Bitartrate	hydrocodone	d03428
52959073580	Acetaminophen-Hydrocodone Bitartrate	hydrocodone	d03428
52959073584	Acetaminophen-Hydrocodone Bitartrate	hydrocodone	d03428
52959073590	Acetaminophen-Hydrocodone Bitartrate	hydrocodone	d03428
54569530500	Norco	hydrocodone	d03428
54569602701	Acetaminophen-Hydrocodone Bitartrate	hydrocodone	d03428
54569602702	Acetaminophen-Hydrocodone Bitartrate	hydrocodone	d03428
54569602703	Acetaminophen-Hydrocodone Bitartrate	hydrocodone	d03428
55289080212	Acetaminophen-Hydrocodone Bitartrate	hydrocodone	d03428
55289080220	Acetaminophen-Hydrocodone Bitartrate	hydrocodone	d03428
55289080230	Acetaminophen-Hydrocodone Bitartrate	hydrocodone	d03428
55289080260	Acetaminophen-Hydrocodone Bitartrate	hydrocodone	d03428
55289080290	Acetaminophen-Hydrocodone Bitartrate	hydrocodone	d03428
57664017013	Acetaminophen-Hydrocodone Bitartrate	hydrocodone	d03428
57664017088	Acetaminophen-Hydrocodone Bitartrate	hydrocodone	d03428
60429057301	Acetaminophen-Hydrocodone Bitartrate	hydrocodone	d03428
60429057305	Acetaminophen-Hydrocodone Bitartrate	hydrocodone	d03428
60429057312	Acetaminophen-Hydrocodone Bitartrate	hydrocodone	d03428
60429057318	Acetaminophen-Hydrocodone Bitartrate	hydrocodone	d03428
60429057330	Acetaminophen-Hydrocodone Bitartrate	hydrocodone	d03428
60429057360	Acetaminophen-Hydrocodone Bitartrate	hydrocodone	d03428
60429057390	Acetaminophen-Hydrocodone Bitartrate	hydrocodone	d03428
65162011510	Acetaminophen-Hydrocodone Bitartrate	hydrocodone	d03428
65162011511	Acetaminophen-Hydrocodone Bitartrate	hydrocodone	d03428

65162011550	Acetaminophen-Hydrocodone Bitartrate	hydrocodone	d03428
68084060101	Acetaminophen-Hydrocodone Bitartrate	hydrocodone	d03428
68084060111	Acetaminophen-Hydrocodone Bitartrate	hydrocodone	d03428
68084086301	Acetaminophen-Hydrocodone Bitartrate	hydrocodone	d03428
68084086309	Acetaminophen-Hydrocodone Bitartrate	hydrocodone	d03428
68084086311	Acetaminophen-Hydrocodone Bitartrate	hydrocodone	d03428
68308022201	Lorcet Plus	hydrocodone	d03428
68308022501	Lorcet Plus	hydrocodone	d03428
68387023712	Acetaminophen-Hydrocodone Bitartrate	hydrocodone	d03428
00121077204	Acetaminophen-Hydrocodone Bitartrate	hydrocodone	d03428
00121077216	Acetaminophen-Hydrocodone Bitartrate	hydrocodone	d03428
00121231615	Acetaminophen-Hydrocodone Bitartrate	hydrocodone	d03428
00121231640	Acetaminophen-Hydrocodone Bitartrate	hydrocodone	d03428
00121477215	Acetaminophen-Hydrocodone Bitartrate	hydrocodone	d03428
00713070389	Hycet	hydrocodone	d03428
00713070489	Acetaminophen-Hydrocodone Bitartrate	hydrocodone	d03428
13551080105	Hycet	hydrocodone	d03428
64376064016	Acetaminophen-Hydrocodone Bitartrate	hydrocodone	d03428
64376064040	Acetaminophen-Hydrocodone Bitartrate	hydrocodone	d03428
64950034047	Acetaminophen-Hydrocodone Bitartrate	hydrocodone	d03428
66689002301	Acetaminophen-Hydrocodone Bitartrate	hydrocodone	d03428
66689002304	Acetaminophen-Hydrocodone Bitartrate	hydrocodone	d03428
66689002316	Acetaminophen-Hydrocodone Bitartrate	hydrocodone	d03428
66689002350	Acetaminophen-Hydrocodone Bitartrate	hydrocodone	d03428
68094071459	Acetaminophen-Hydrocodone Bitartrate	hydrocodone	d03428
68094071462	Acetaminophen-Hydrocodone Bitartrate	hydrocodone	d03428
76014000125	Hycet	hydrocodone	d03428
76181000125	Acetaminophen-Hydrocodone Bitartrate	hydrocodone	d03428
24090068371	Primlev	oxycodone	d03431
24090068379	Primlev	oxycodone	d03431
24090068385	Primlev	oxycodone	d03431
24090068388	Primlev	oxycodone	d03431
59702068301	Primlev	oxycodone	d03431
24090068171	Primlev	oxycodone	d03431
24090068179	Primlev	oxycodone	d03431
24090068185	Primlev	oxycodone	d03431
24090068188	Primlev	oxycodone	d03431
59702068101	Primlev	oxycodone	d03431
24090068285	Primlev	oxycodone	d03431
24090068288	Primlev	oxycodone	d03431
59702068201	Primlev	oxycodone	d03431
00228298311	Acetaminophen-Oxycodone Hydrochloride	oxycodone	d03431
00378710601	Acetaminophen-Oxycodone Hydrochloride	oxycodone	d03431
00406052301	Acetaminophen-Oxycodone Hydrochloride	oxycodone	d03431
00406052305	Acetaminophen-Oxycodone Hydrochloride	oxycodone	d03431

00406052362	Acetaminophen-Oxycodone Hydrochloride	oxycodone	d03431
00591093201	Acetaminophen-Oxycodone Hydrochloride	oxycodone	d03431
00603498221	Acetaminophen-Oxycodone Hydrochloride	oxycodone	d03431
00603498228	Acetaminophen-Oxycodone Hydrochloride	oxycodone	d03431
00904643961	Acetaminophen-Oxycodone Hydrochloride	oxycodone	d03431
13107004601	Acetaminophen-Oxycodone Hydrochloride	oxycodone	d03431
13107004605	Acetaminophen-Oxycodone Hydrochloride	oxycodone	d03431
16590061830	Percocet 10/325	oxycodone	d03431
21695061940	Acetaminophen-Oxycodone Hydrochloride	oxycodone	d03431
23490759201	Acetaminophen-Oxycodone Hydrochloride	oxycodone	d03431
23490759202	Acetaminophen-Oxycodone Hydrochloride	oxycodone	d03431
23490759203	Acetaminophen-Oxycodone Hydrochloride	oxycodone	d03431
23490759204	Acetaminophen-Oxycodone Hydrochloride	oxycodone	d03431
23490759207	Acetaminophen-Oxycodone Hydrochloride	oxycodone	d03431
23490759209	Acetaminophen-Oxycodone Hydrochloride	oxycodone	d03431
31722019401	Acetaminophen-Oxycodone Hydrochloride	oxycodone	d03431
31722019405	Acetaminophen-Oxycodone Hydrochloride	oxycodone	d03431
42291064601	Acetaminophen-Oxycodone Hydrochloride	oxycodone	d03431
42858010401	Acetaminophen-Oxycodone Hydrochloride	oxycodone	d03431
42858010450	Acetaminophen-Oxycodone Hydrochloride	oxycodone	d03431
43063023120	Acetaminophen-Oxycodone Hydrochloride	oxycodone	d03431
43063023130	Acetaminophen-Oxycodone Hydrochloride	oxycodone	d03431
43063023160	Acetaminophen-Oxycodone Hydrochloride	oxycodone	d03431
43063023190	Acetaminophen-Oxycodone Hydrochloride	oxycodone	d03431
43063052806	Acetaminophen-Oxycodone Hydrochloride	oxycodone	d03431
43063052830	Acetaminophen-Oxycodone Hydrochloride	oxycodone	d03431
43063052860	Acetaminophen-Oxycodone Hydrochloride	oxycodone	d03431
43063052890	Acetaminophen-Oxycodone Hydrochloride	oxycodone	d03431
43063052898	Acetaminophen-Oxycodone Hydrochloride	oxycodone	d03431
47781023001	Acetaminophen-Oxycodone Hydrochloride	oxycodone	d03431
47781023005	Acetaminophen-Oxycodone Hydrochloride	oxycodone	d03431
47781023063	Acetaminophen-Oxycodone Hydrochloride	oxycodone	d03431
49999085405	Acetaminophen-Oxycodone Hydrochloride	oxycodone	d03431
53746020401	Acetaminophen-Oxycodone Hydrochloride	oxycodone	d03431
53746020405	Acetaminophen-Oxycodone Hydrochloride	oxycodone	d03431
54868502400	Acetaminophen-Oxycodone Hydrochloride	oxycodone	d03431
54868502405	Acetaminophen-Oxycodone Hydrochloride	oxycodone	d03431
55887012901	Acetaminophen-Oxycodone Hydrochloride	oxycodone	d03431
57664016088	Acetaminophen-Oxycodone Hydrochloride	oxycodone	d03431
60951071270	Endocet 10/325	oxycodone	d03431
63481062970	Percocet 10/325	oxycodone	d03431
63739021710	Acetaminophen-Oxycodone Hydrochloride	oxycodone	d03431
63874123300	Acetaminophen-Oxycodone Hydrochloride	oxycodone	d03431
63874123303	Acetaminophen-Oxycodone Hydrochloride	oxycodone	d03431
63874123306	Acetaminophen-Oxycodone Hydrochloride	oxycodone	d03431

63874123309	Acetaminophen-Oxycodone Hydrochloride	oxycodone	d03431
66336014794	Acetaminophen-Oxycodone Hydrochloride	oxycodone	d03431
68071034430	Acetaminophen-Oxycodone Hydrochloride	oxycodone	d03431
68084037801	Acetaminophen-Oxycodone Hydrochloride	oxycodone	d03431
68084037811	Acetaminophen-Oxycodone Hydrochloride	oxycodone	d03431
68084071001	Acetaminophen-Oxycodone Hydrochloride	oxycodone	d03431
68308048047	Acetaminophen-Oxycodone Hydrochloride	oxycodone	d03431
68308084301	Acetaminophen-Oxycodone Hydrochloride	oxycodone	d03431
00378710301	Acetaminophen-Oxycodone Hydrochloride	oxycodone	d03431
00603497821	Acetaminophen-Oxycodone Hydrochloride	oxycodone	d03431
31722019101	Acetaminophen-Oxycodone Hydrochloride	oxycodone	d03431
60951070170	Endocet 2.5/325	oxycodone	d03431
63481062770	Percocet 2.5/325	oxycodone	d03431
68308084001	Acetaminophen-Oxycodone Hydrochloride	oxycodone	d03431
00054055125	Acetaminophen-Oxycodone Hydrochloride	oxycodone	d03431
00054055129	Acetaminophen-Oxycodone Hydrochloride	oxycodone	d03431
00054465025	Roxicet	oxycodone	d03431
00054865024	Roxicet	oxycodone	d03431
00228298111	Acetaminophen-Oxycodone Hydrochloride	oxycodone	d03431
00228298150	Acetaminophen-Oxycodone Hydrochloride	oxycodone	d03431
00378710401	Acetaminophen-Oxycodone Hydrochloride	oxycodone	d03431
00406051201	Acetaminophen-Oxycodone Hydrochloride	oxycodone	d03431
00406051205	Acetaminophen-Oxycodone Hydrochloride	oxycodone	d03431
00406051223	Acetaminophen-Oxycodone Hydrochloride	oxycodone	d03431
00406051262	Acetaminophen-Oxycodone Hydrochloride	oxycodone	d03431
00406051291	Acetaminophen-Oxycodone Hydrochloride	oxycodone	d03431
00591074901	Acetaminophen-Oxycodone Hydrochloride	oxycodone	d03431
00591074905	Acetaminophen-Oxycodone Hydrochloride	oxycodone	d03431
00603499821	Acetaminophen-Oxycodone Hydrochloride	oxycodone	d03431
00603499828	Acetaminophen-Oxycodone Hydrochloride	oxycodone	d03431
00904643761	Acetaminophen-Oxycodone Hydrochloride	oxycodone	d03431
13107004401	Acetaminophen-Oxycodone Hydrochloride	oxycodone	d03431
13107004405	Acetaminophen-Oxycodone Hydrochloride	oxycodone	d03431
16590061962	Percocet 5/325	oxycodone	d03431
23490605300	Acetaminophen-Oxycodone Hydrochloride	oxycodone	d03431
23490605301	Acetaminophen-Oxycodone Hydrochloride	oxycodone	d03431
23490605302	Acetaminophen-Oxycodone Hydrochloride	oxycodone	d03431
23490605303	Acetaminophen-Oxycodone Hydrochloride	oxycodone	d03431
23490605304	Acetaminophen-Oxycodone Hydrochloride	oxycodone	d03431
23490605305	Acetaminophen-Oxycodone Hydrochloride	oxycodone	d03431
23490605306	Acetaminophen-Oxycodone Hydrochloride	oxycodone	d03431
23490605307	Acetaminophen-Oxycodone Hydrochloride	oxycodone	d03431
23490605308	Acetaminophen-Oxycodone Hydrochloride	oxycodone	d03431
23490605309	Acetaminophen-Oxycodone Hydrochloride	oxycodone	d03431
31722019201	Acetaminophen-Oxycodone Hydrochloride	oxycodone	d03431

31722019205	Acetaminophen-Oxycodone Hydrochloride	oxycodone	d03431
42291064501	Acetaminophen-Oxycodone Hydrochloride	oxycodone	d03431
42858010201	Acetaminophen-Oxycodone Hydrochloride	oxycodone	d03431
42858010250	Acetaminophen-Oxycodone Hydrochloride	oxycodone	d03431
43063002502	Acetaminophen-Oxycodone Hydrochloride	oxycodone	d03431
43063002504	Acetaminophen-Oxycodone Hydrochloride	oxycodone	d03431
43063002506	Acetaminophen-Oxycodone Hydrochloride	oxycodone	d03431
43063002512	Acetaminophen-Oxycodone Hydrochloride	oxycodone	d03431
43063027904	Acetaminophen-Oxycodone Hydrochloride	oxycodone	d03431
43063027906	Acetaminophen-Oxycodone Hydrochloride	oxycodone	d03431
43063027910	Acetaminophen-Oxycodone Hydrochloride	oxycodone	d03431
43063027912	Acetaminophen-Oxycodone Hydrochloride	oxycodone	d03431
43063027918	Acetaminophen-Oxycodone Hydrochloride	oxycodone	d03431
43063027920	Acetaminophen-Oxycodone Hydrochloride	oxycodone	d03431
43063027924	Acetaminophen-Oxycodone Hydrochloride	oxycodone	d03431
43063027930	Acetaminophen-Oxycodone Hydrochloride	oxycodone	d03431
43063027940	Acetaminophen-Oxycodone Hydrochloride	oxycodone	d03431
43063027960	Acetaminophen-Oxycodone Hydrochloride	oxycodone	d03431
43063027986	Acetaminophen-Oxycodone Hydrochloride	oxycodone	d03431
43063027990	Acetaminophen-Oxycodone Hydrochloride	oxycodone	d03431
43063027993	Acetaminophen-Oxycodone Hydrochloride	oxycodone	d03431
43063027998	Acetaminophen-Oxycodone Hydrochloride	oxycodone	d03431
43063027999	Acetaminophen-Oxycodone Hydrochloride	oxycodone	d03431
43063065302	Acetaminophen-Oxycodone Hydrochloride	oxycodone	d03431
43063065304	Acetaminophen-Oxycodone Hydrochloride	oxycodone	d03431
43063065306	Acetaminophen-Oxycodone Hydrochloride	oxycodone	d03431
43063065310	Acetaminophen-Oxycodone Hydrochloride	oxycodone	d03431
43063065312	Acetaminophen-Oxycodone Hydrochloride	oxycodone	d03431
43063065318	Acetaminophen-Oxycodone Hydrochloride	oxycodone	d03431
43063065320	Acetaminophen-Oxycodone Hydrochloride	oxycodone	d03431
43063065324	Acetaminophen-Oxycodone Hydrochloride	oxycodone	d03431
43063065330	Acetaminophen-Oxycodone Hydrochloride	oxycodone	d03431
43063065340	Acetaminophen-Oxycodone Hydrochloride	oxycodone	d03431
43063065356	Acetaminophen-Oxycodone Hydrochloride	oxycodone	d03431
43063065360	Acetaminophen-Oxycodone Hydrochloride	oxycodone	d03431
43063065388	Acetaminophen-Oxycodone Hydrochloride	oxycodone	d03431
43063065390	Acetaminophen-Oxycodone Hydrochloride	oxycodone	d03431
43063065398	Acetaminophen-Oxycodone Hydrochloride	oxycodone	d03431
47781019601	Acetaminophen-Oxycodone Hydrochloride	oxycodone	d03431
47781019605	Acetaminophen-Oxycodone Hydrochloride	oxycodone	d03431
49999085201	Acetaminophen-Oxycodone Hydrochloride	oxycodone	d03431
49999085230	Acetaminophen-Oxycodone Hydrochloride	oxycodone	d03431
49999085260	Acetaminophen-Oxycodone Hydrochloride	oxycodone	d03431
49999085290	Acetaminophen-Oxycodone Hydrochloride	oxycodone	d03431
52959055602	Acetaminophen-Oxycodone Hydrochloride	oxycodone	d03431

52959055630	Acetaminophen-Oxycodone Hydrochloride	oxycodone	d03431
52959055660	Acetaminophen-Oxycodone Hydrochloride	oxycodone	d03431
52959055690	Acetaminophen-Oxycodone Hydrochloride	oxycodone	d03431
53746020301	Acetaminophen-Oxycodone Hydrochloride	oxycodone	d03431
53746020305	Acetaminophen-Oxycodone Hydrochloride	oxycodone	d03431
54868170001	Acetaminophen-Oxycodone Hydrochloride	oxycodone	d03431
54868170002	Acetaminophen-Oxycodone Hydrochloride	oxycodone	d03431
54868170004	Acetaminophen-Oxycodone Hydrochloride	oxycodone	d03431
54868170007	Acetaminophen-Oxycodone Hydrochloride	oxycodone	d03431
55289095110	Acetaminophen-Oxycodone Hydrochloride	oxycodone	d03431
55289095112	Acetaminophen-Oxycodone Hydrochloride	oxycodone	d03431
55289095115	Acetaminophen-Oxycodone Hydrochloride	oxycodone	d03431
55289095120	Acetaminophen-Oxycodone Hydrochloride	oxycodone	d03431
55289095124	Acetaminophen-Oxycodone Hydrochloride	oxycodone	d03431
55289095130	Acetaminophen-Oxycodone Hydrochloride	oxycodone	d03431
55289095140	Acetaminophen-Oxycodone Hydrochloride	oxycodone	d03431
55289095156	Acetaminophen-Oxycodone Hydrochloride	oxycodone	d03431
55289095160	Acetaminophen-Oxycodone Hydrochloride	oxycodone	d03431
55289095173	Acetaminophen-Oxycodone Hydrochloride	oxycodone	d03431
55289095186	Acetaminophen-Oxycodone Hydrochloride	oxycodone	d03431
55289095190	Acetaminophen-Oxycodone Hydrochloride	oxycodone	d03431
55289095193	Acetaminophen-Oxycodone Hydrochloride	oxycodone	d03431
55289095198	Acetaminophen-Oxycodone Hydrochloride	oxycodone	d03431
55289095199	Acetaminophen-Oxycodone Hydrochloride	oxycodone	d03431
57664015513	Acetaminophen-Oxycodone Hydrochloride	oxycodone	d03431
57664015588	Acetaminophen-Oxycodone Hydrochloride	oxycodone	d03431
60951060270	Endocet 5/325	oxycodone	d03431
60951060285	Endocet 5/325	oxycodone	d03431
63481062370	Percocet 5/325	oxycodone	d03431
63481062385	Percocet 5/325	oxycodone	d03431
63739070910	Acetaminophen-Oxycodone Hydrochloride	oxycodone	d03431
63874122700	Acetaminophen-Oxycodone Hydrochloride	oxycodone	d03431
63874122701	Acetaminophen-Oxycodone Hydrochloride	oxycodone	d03431
63874122703	Acetaminophen-Oxycodone Hydrochloride	oxycodone	d03431
63874122706	Acetaminophen-Oxycodone Hydrochloride	oxycodone	d03431
63874122709	Acetaminophen-Oxycodone Hydrochloride	oxycodone	d03431
68071015830	Acetaminophen-Oxycodone Hydrochloride	oxycodone	d03431
68071015840	Acetaminophen-Oxycodone Hydrochloride	oxycodone	d03431
68071015860	Acetaminophen-Oxycodone Hydrochloride	oxycodone	d03431
68071015890	Acetaminophen-Oxycodone Hydrochloride	oxycodone	d03431
68071015891	Acetaminophen-Oxycodone Hydrochloride	oxycodone	d03431
68071022420	Acetaminophen-Oxycodone Hydrochloride	oxycodone	d03431
68071022430	Acetaminophen-Oxycodone Hydrochloride	oxycodone	d03431
68084035501	Acetaminophen-Oxycodone Hydrochloride	oxycodone	d03431
68084035511	Acetaminophen-Oxycodone Hydrochloride	oxycodone	d03431

68094001361	Acetaminophen-Oxycodone Hydrochloride	oxycodone	d03431
68308040547	Acetaminophen-Oxycodone Hydrochloride	oxycodone	d03431
68308084101	Acetaminophen-Oxycodone Hydrochloride	oxycodone	d03431
00054368663	Roxicet	oxycodone	d03431
00054864816	Roxicet	oxycodone	d03431
00228298211	Acetaminophen-Oxycodone Hydrochloride	oxycodone	d03431
00378710501	Acetaminophen-Oxycodone Hydrochloride	oxycodone	d03431
00406052201	Acetaminophen-Oxycodone Hydrochloride	oxycodone	d03431
00406052205	Acetaminophen-Oxycodone Hydrochloride	oxycodone	d03431
00406052262	Acetaminophen-Oxycodone Hydrochloride	oxycodone	d03431
00591093301	Acetaminophen-Oxycodone Hydrochloride	oxycodone	d03431
00603497921	Acetaminophen-Oxycodone Hydrochloride	oxycodone	d03431
00603497928	Acetaminophen-Oxycodone Hydrochloride	oxycodone	d03431
00904643861	Acetaminophen-Oxycodone Hydrochloride	oxycodone	d03431
13107004501	Acetaminophen-Oxycodone Hydrochloride	oxycodone	d03431
13107004505	Acetaminophen-Oxycodone Hydrochloride	oxycodone	d03431
23490782603	Acetaminophen-Oxycodone Hydrochloride	oxycodone	d03431
23490782606	Acetaminophen-Oxycodone Hydrochloride	oxycodone	d03431
23490782607	Acetaminophen-Oxycodone Hydrochloride	oxycodone	d03431
23490782609	Acetaminophen-Oxycodone Hydrochloride	oxycodone	d03431
23490782703	Endocet 7.5/325	oxycodone	d03431
23490782706	Endocet 7.5/325	oxycodone	d03431
23490782709	Endocet 7.5/325	oxycodone	d03431
31722019301	Acetaminophen-Oxycodone Hydrochloride	oxycodone	d03431
31722019305	Acetaminophen-Oxycodone Hydrochloride	oxycodone	d03431
42858010301	Acetaminophen-Oxycodone Hydrochloride	oxycodone	d03431
42858010350	Acetaminophen-Oxycodone Hydrochloride	oxycodone	d03431
43063037912	Acetaminophen-Oxycodone Hydrochloride	oxycodone	d03431
43063037930	Acetaminophen-Oxycodone Hydrochloride	oxycodone	d03431
43063052312	Acetaminophen-Oxycodone Hydrochloride	oxycodone	d03431
43063052330	Acetaminophen-Oxycodone Hydrochloride	oxycodone	d03431
47781022901	Acetaminophen-Oxycodone Hydrochloride	oxycodone	d03431
47781022905	Acetaminophen-Oxycodone Hydrochloride	oxycodone	d03431
47781022963	Acetaminophen-Oxycodone Hydrochloride	oxycodone	d03431
49999085301	Acetaminophen-Oxycodone Hydrochloride	oxycodone	d03431
49999085330	Acetaminophen-Oxycodone Hydrochloride	oxycodone	d03431
49999085360	Acetaminophen-Oxycodone Hydrochloride	oxycodone	d03431
49999085390	Acetaminophen-Oxycodone Hydrochloride	oxycodone	d03431
52959055402	Acetaminophen-Oxycodone Hydrochloride	oxycodone	d03431
52959055430	Acetaminophen-Oxycodone Hydrochloride	oxycodone	d03431
52959055460	Acetaminophen-Oxycodone Hydrochloride	oxycodone	d03431
52959055490	Acetaminophen-Oxycodone Hydrochloride	oxycodone	d03431
54868533804	Acetaminophen-Oxycodone Hydrochloride	oxycodone	d03431
57664015688	Acetaminophen-Oxycodone Hydrochloride	oxycodone	d03431
60951070070	Endocet 7.5/325	oxycodone	d03431

63481062870	Percocet 7.5/325	oxycodone	d03431
63739063610	Acetaminophen-Oxycodone Hydrochloride	oxycodone	d03431
65162020710	Acetaminophen-Oxycodone Hydrochloride	oxycodone	d03431
65162020750	Acetaminophen-Oxycodone Hydrochloride	oxycodone	d03431
68084037901	Acetaminophen-Oxycodone Hydrochloride	oxycodone	d03431
68084037911	Acetaminophen-Oxycodone Hydrochloride	oxycodone	d03431
68084069901	Acetaminophen-Oxycodone Hydrochloride	oxycodone	d03431
68084069911	Acetaminophen-Oxycodone Hydrochloride	oxycodone	d03431
68308047547	Acetaminophen-Oxycodone Hydrochloride	oxycodone	d03431
68308084201	Acetaminophen-Oxycodone Hydrochloride	oxycodone	d03431
23635011501	Xartemis XR	oxycodone	d03431
00591039601	Acetaminophen-Pentazocine	pentazocine	d03682
21695063460	Acetaminophen-Pentazocine	pentazocine	d03682
43386067001	Acetaminophen-Pentazocine	pentazocine	d03682
00172635900	Acetaminophen-Tramadol Hydrochloride	tramadol	d04766
00172635910	Acetaminophen-Tramadol Hydrochloride	tramadol	d04766
00172635960	Acetaminophen-Tramadol Hydrochloride	tramadol	d04766
00172635970	Acetaminophen-Tramadol Hydrochloride	tramadol	d04766
00378808801	Acetaminophen-Tramadol Hydrochloride	tramadol	d04766
00378808805	Acetaminophen-Tramadol Hydrochloride	tramadol	d04766
16590063471	Acetaminophen-Tramadol Hydrochloride	tramadol	d04766
21695014372	Ultracet	tramadol	d04766
21695023620	Acetaminophen-Tramadol Hydrochloride	tramadol	d04766
23490758601	Acetaminophen-Tramadol Hydrochloride	tramadol	d04766
23490758602	Acetaminophen-Tramadol Hydrochloride	tramadol	d04766
23490758603	Acetaminophen-Tramadol Hydrochloride	tramadol	d04766
23490758604	Acetaminophen-Tramadol Hydrochloride	tramadol	d04766
23490758605	Acetaminophen-Tramadol Hydrochloride	tramadol	d04766
23490758606	Acetaminophen-Tramadol Hydrochloride	tramadol	d04766
33358034330	Acetaminophen-Tramadol Hydrochloride	tramadol	d04766
42291083290	Acetaminophen-Tramadol Hydrochloride	tramadol	d04766
42571011901	Acetaminophen-Tramadol Hydrochloride	tramadol	d04766
42571011905	Acetaminophen-Tramadol Hydrochloride	tramadol	d04766
43063059901	Acetaminophen-Tramadol Hydrochloride	tramadol	d04766
43063059915	Acetaminophen-Tramadol Hydrochloride	tramadol	d04766
43063059920	Acetaminophen-Tramadol Hydrochloride	tramadol	d04766
43063059930	Acetaminophen-Tramadol Hydrochloride	tramadol	d04766
43063059960	Acetaminophen-Tramadol Hydrochloride	tramadol	d04766
43063078220	Acetaminophen-Tramadol Hydrochloride	traMADol	d04766
43063078230	Acetaminophen-Tramadol Hydrochloride	traMADol	d04766
43063078260	Acetaminophen-Tramadol Hydrochloride	traMADol	d04766
49884094601	Acetaminophen-Tramadol Hydrochloride	tramadol	d04766
49884094605	Acetaminophen-Tramadol Hydrochloride	tramadol	d04766
49999011830	Ultracet	tramadol	d04766
49999069300	Acetaminophen-Tramadol Hydrochloride	tramadol	d04766

49999069330	Acetaminophen-Tramadol Hydrochloride	tramadol	d04766
49999069360	Acetaminophen-Tramadol Hydrochloride	tramadol	d04766
50268077315	Acetaminophen-Tramadol Hydrochloride	tramadol	d04766
50268077415	Acetaminophen-Tramadol Hydrochloride	tramadol	d04766
50458065010	Ultracet	tramadol	d04766
50458065060	Ultracet	tramadol	d04766
52959066600	Ultracet	tramadol	d04766
52959066602	Ultracet	tramadol	d04766
52959066620	Ultracet	tramadol	d04766
52959066628	Ultracet	tramadol	d04766
52959066630	Ultracet	tramadol	d04766
52959066640	Ultracet	tramadol	d04766
52959066656	Ultracet	tramadol	d04766
52959066660	Ultracet	tramadol	d04766
52959066690	Ultracet	tramadol	d04766
52959081400	Acetaminophen-Tramadol Hydrochloride	tramadol	d04766
52959081410	Acetaminophen-Tramadol Hydrochloride	tramadol	d04766
52959081412	Acetaminophen-Tramadol Hydrochloride	tramadol	d04766
52959081415	Acetaminophen-Tramadol Hydrochloride	tramadol	d04766
52959081418	Acetaminophen-Tramadol Hydrochloride	tramadol	d04766
52959081420	Acetaminophen-Tramadol Hydrochloride	tramadol	d04766
52959081430	Acetaminophen-Tramadol Hydrochloride	tramadol	d04766
52959081440	Acetaminophen-Tramadol Hydrochloride	tramadol	d04766
52959081460	Acetaminophen-Tramadol Hydrochloride	tramadol	d04766
52959081490	Acetaminophen-Tramadol Hydrochloride	tramadol	d04766
53746061701	Acetaminophen-Tramadol Hydrochloride	tramadol	d04766
53746061705	Acetaminophen-Tramadol Hydrochloride	tramadol	d04766
53746061710	Acetaminophen-Tramadol Hydrochloride	tramadol	d04766
54569530800	Ultracet	tramadol	d04766
54569530801	Ultracet	tramadol	d04766
54569568000	Acetaminophen-Tramadol Hydrochloride	tramadol	d04766
54569568001	Acetaminophen-Tramadol Hydrochloride	tramadol	d04766
54569568002	Acetaminophen-Tramadol Hydrochloride	tramadol	d04766
54569568003	Acetaminophen-Tramadol Hydrochloride	tramadol	d04766
54868529100	Acetaminophen-Tramadol Hydrochloride	tramadol	d04766
55289061720	Ultracet	tramadol	d04766
55289089515	Acetaminophen-Tramadol Hydrochloride	tramadol	d04766
55289089520	Acetaminophen-Tramadol Hydrochloride	tramadol	d04766
55289089530	Acetaminophen-Tramadol Hydrochloride	tramadol	d04766
55289089560	Acetaminophen-Tramadol Hydrochloride	tramadol	d04766
57664053713	Acetaminophen-Tramadol Hydrochloride	tramadol	d04766
57664053718	Acetaminophen-Tramadol Hydrochloride	tramadol	d04766
57664053788	Acetaminophen-Tramadol Hydrochloride	tramadol	d04766
60429050001	Acetaminophen-Tramadol Hydrochloride	tramadol	d04766
60429050005	Acetaminophen-Tramadol Hydrochloride	tramadol	d04766

60505264401	Acetaminophen-Tramadol Hydrochloride	tramadol	d04766
60505264405	Acetaminophen-Tramadol Hydrochloride	tramadol	d04766
60760005320	Acetaminophen-Tramadol Hydrochloride	tramadol	d04766
65162061710	Acetaminophen-Tramadol Hydrochloride	tramadol	d04766
65162061711	Acetaminophen-Tramadol Hydrochloride	tramadol	d04766
65162061750	Acetaminophen-Tramadol Hydrochloride	tramadol	d04766
65862092201	Acetaminophen-Tramadol Hydrochloride	tramadol	d04766
65862092205	Acetaminophen-Tramadol Hydrochloride	tramadol	d04766
67877032201	Acetaminophen-Tramadol Hydrochloride	tramadol	d04766
67877032205	Acetaminophen-Tramadol Hydrochloride	tramadol	d04766
68084013901	Acetaminophen-Tramadol Hydrochloride	tramadol	d04766
68084013911	Acetaminophen-Tramadol Hydrochloride	tramadol	d04766
68084049601	Acetaminophen-Tramadol Hydrochloride	tramadol	d04766
68084049611	Acetaminophen-Tramadol Hydrochloride	tramadol	d04766
68084082501	Acetaminophen-Tramadol Hydrochloride	tramadol	d04766
68084082511	Acetaminophen-Tramadol Hydrochloride	tramadol	d04766
68382033401	Acetaminophen-Tramadol Hydrochloride	tramadol	d04766
68382033405	Acetaminophen-Tramadol Hydrochloride	tramadol	d04766
00527131201	Aspirin/Butalbital/Caffeine/Codeine	codeine	d03426
00591354601	Aspirin/Butalbital/Caffeine/Codeine	codeine	d03426
00591354605	Aspirin/Butalbital/Caffeine/Codeine	codeine	d03426
23490682503	Aspirin/Butalbital/Caffeine/Codeine	codeine	d03426
51991007401	Ascomp with Codeine	codeine	d03426
51991007405	Ascomp with Codeine	codeine	d03426
52544095601	Fiorinal with Codeine	codeine	d03426
52959086500	Aspirin/Butalbital/Caffeine/Codeine	codeine	d03426
52959086530	Aspirin/Butalbital/Caffeine/Codeine	codeine	d03426
54868103701	Aspirin/Butalbital/Caffeine/Codeine	codeine	d03426
54868103703	Aspirin/Butalbital/Caffeine/Codeine	codeine	d03426
55289002620	Fiorinal with Codeine	codeine	d03426
63629295201	Aspirin/Butalbital/Caffeine/Codeine	codeine	d03426
63874021901	Aspirin/Butalbital/Caffeine/Codeine	codeine	d03426
63874021920	Aspirin/Butalbital/Caffeine/Codeine	codeine	d03426
63874021925	Aspirin/Butalbital/Caffeine/Codeine	codeine	d03426
63874021930	Aspirin/Butalbital/Caffeine/Codeine	codeine	d03426
68308031210	Aspirin/Butalbital/Caffeine/Codeine	codeine	d03426
00247121620	Synalgos-DC	dihydrocodeine	d03430
00247201512	Synalgos-DC	dihydrocodeine	d03430
49708041988	Synalgos-DC	dihydrocodeine	d03430
55289033915	Synalgos-DC	dihydrocodeine	d03430
57664041988	Aspirin/caffeine/dihydrocodeine	dihydrocodeine	d03430
00185074901	ASA/Carisoprodol/Codeine Phosphate	codeine	d03470
00247176120	ASA/Carisoprodol/Codeine Phosphate	codeine	d03470
52959086800	ASA/Carisoprodol/Codeine Phosphate	codeine	d03470
52959086860	ASA/Carisoprodol/Codeine Phosphate	codeine	d03470

52959086890	ASA/Carisoprodol/Codeine Phosphate	codeine	d03470
64980017601	ASA/Carisoprodol/Codeine Phosphate	codeine	d03470
00378611701	Aspirin-Oxycodone	oxycodone	d03432
00591355101	Aspirin-Oxycodone	oxycodone	d03432
60951031070	Endodan	oxycodone	d03432
68308084501	Aspirin-Oxycodone	oxycodone	d03432
00574704512	Belladonna Alkaloids-Opium	opium	d03436
00574704012	Belladonna Alkaloids-Opium	opium	d03436
00093360140	Buprenorphine	buprenorphine	d00840
42858049340	Buprenorphine	buprenorphine	d00840
54569632500	Butrans	buprenorphine	d00840
59011075104	Butrans	buprenorphine	d00840
00093360240	Buprenorphine	buprenorphine	d00840
42858058640	Buprenorphine	buprenorphine	d00840
59011075804	Butrans	buprenorphine	d00840
63481020760	Belbuca	buprenorphine	d00840
00093360340	Buprenorphine	buprenorphine	d00840
42858083940	Buprenorphine	buprenorphine	d00840
54569632600	Butrans	buprenorphine	d00840
59011075204	Butrans	buprenorphine	d00840
59385002360	Belbuca	buprenorphine	d00840
63481034860	Belbuca	buprenorphine	d00840
59385002460	Belbuca	buprenorphine	d00840
63481051960	Belbuca	buprenorphine	d00840
00093360040	Buprenorphine	buprenorphine	d00840
42858075040	Buprenorphine	buprenorphine	d00840
59011075004	Butrans	buprenorphine	d00840
59385002560	Belbuca	buprenorphine	d00840
63481068560	Belbuca	buprenorphine	d00840
42858035340	Buprenorphine	buprenorphine	d00840
59011075704	Butrans	buprenorphine	d00840
59385002160	Belbuca	buprenorphine	d00840
63481016101	Belbuca	buprenorphine	d00840
63481016160	Belbuca	buprenorphine	d00840
63481082060	Belbuca	buprenorphine	d00840
59385002760	Belbuca	buprenorphine	d00840
63481095260	Belbuca	buprenorphine	d00840
59385001201	Bunavail	buprenorphine	d04819
59385001230	Bunavail	buprenorphine	d04819
59385001401	Bunavail	buprenorphine	d04819
59385001430	Bunavail	buprenorphine	d04819
59385001601	Bunavail	buprenorphine	d04819
59385001630	Bunavail	buprenorphine	d04819
00054309036	Butorphanol Tartrate	butorphanol	d00838
00378963943	Butorphanol Tartrate	butorphanol	d00838

54569598800	Butorphanol Tartrate	butorphanol	d00838
60505081301	Butorphanol Tartrate	butorphanol	d00838
00054024324	Codeine Sulfate	codeine	d00012
00054024424	Codeine Sulfate	codeine	d00012
00054024425	Codeine Sulfate	codeine	d00012
00527169801	Codeine Sulfate	codeine	d00012
00527169891	Codeine Sulfate	codeine	d00012
51224030010	Codeine Sulfate	codeine	d00012
68094076059	Codeine Sulfate	codeine	d00012
68094076062	Codeine Sulfate	codeine	d00012
68094076159	Codeine Sulfate	codeine	d00012
68094076162	Codeine Sulfate	codeine	d00012
00054024525	Codeine Sulfate	codeine	d00012
00527169901	Codeine Sulfate	codeine	d00012
54868254100	Codeine Sulfate	codeine	d00012
54868254101	Codeine Sulfate	codeine	d00012
54868254102	Codeine Sulfate	codeine	d00012
63459054104	Fentora	fentanyl	d00233
63459054128	Fentora	fentanyl	d00233
20482000110	Subsys	fentanyl	d00233
20482000130	Subsys	fentanyl	d00233
42358010032	Abstral	fentanyl	d00233
42747022132	Abstral	fentanyl	d00233
57881033132	Abstral	fentanyl	d00233
00093690319	FentaNYL	fentanyl	d00233
00093690345	FentaNYL	fentanyl	d00233
00245042305	FentaNYL	fentanyl	d00233
00245042389	FentaNYL	fentanyl	d00233
00378912416	FentaNYL	fentanyl	d00233
00378912498	FentaNYL	fentanyl	d00233
00406900076	FentaNYL	fentanyl	d00233
00591321454	FentaNYL	fentanyl	d00233
00591321472	FentaNYL	fentanyl	d00233
00591360372	FentaNYL	fentanyl	d00233
00781724455	FentaNYL Matrix	fentanyl	d00233
47781042847	FentaNYL	fentanyl	d00233
49884076478	FentaNYL	fentanyl	d00233
49999083401	FentaNYL	fentanyl	d00233
49999083405	FentaNYL	fentanyl	d00233
50458009405	Duragesic-100	fentanyl	d00233
54868152300	FentaNYL	fentanyl	d00233
60505700400	FentaNYL	fentanyl	d00233
60505700402	FentaNYL	fentanyl	d00233
60505700900	FentaNYL	fentanyl	d00233
60505700902	FentaNYL	fentanyl	d00233

60505701400	FentaNYL	fentanyl	d00233
60505701402	FentaNYL	fentanyl	d00233
67767012318	FentaNYL	fentanyl	d00233
13913000901	Lazanda	fentanyl	d00233
51772031101	Lazanda	fentanyl	d00233
51772031104	Lazanda	fentanyl	d00233
00378911916	FentaNYL	fentanyl	d00233
00378911998	FentaNYL	fentanyl	d00233
00406901276	FentaNYL	fentanyl	d00233
00781724055	FentaNYL Matrix	fentanyl	d00233
35356006205	FentaNYL	fentanyl	d00233
47781042347	FentaNYL	fentanyl	d00233
50458009005	Duragesic-12	fentanyl	d00233
60505701000	FentaNYL	fentanyl	d00233
60505701002	FentaNYL	fentanyl	d00233
00093786965	FentaNYL Citrate	fentanyl	d00233
00406921230	FentaNYL Citrate	fentanyl	d00233
49884046355	FentaNYL Citrate	fentanyl	d00233
55253007401	FentaNYL Citrate	fentanyl	d00233
55253007430	FentaNYL Citrate	fentanyl	d00233
63459051201	Actiq	fentanyl	d00233
63459051230	Actiq	fentanyl	d00233
00093787065	FentaNYL Citrate	fentanyl	d00233
00406921630	FentaNYL Citrate	fentanyl	d00233
49884046455	FentaNYL Citrate	fentanyl	d00233
55253007501	FentaNYL Citrate	fentanyl	d00233
55253007530	FentaNYL Citrate	fentanyl	d00233
63459051601	Actiq	fentanyl	d00233
63459051630	Actiq	fentanyl	d00233
63459054204	Fentora	fentanyl	d00233
63459054228	Fentora	fentanyl	d00233
00093786565	FentaNYL Citrate	fentanyl	d00233
00406920230	FentaNYL Citrate	fentanyl	d00233
49884045952	FentaNYL Citrate	fentanyl	d00233
49884045955	FentaNYL Citrate	fentanyl	d00233
55253007001	FentaNYL Citrate	fentanyl	d00233
55253007030	FentaNYL Citrate	fentanyl	d00233
63459050201	Actiq	fentanyl	d00233
63459050230	Actiq	fentanyl	d00233
20482000210	Subsys	fentanyl	d00233
20482000230	Subsys	fentanyl	d00233
42358020032	Abstral	fentanyl	d00233
42747022232	Abstral	fentanyl	d00233
57881033232	Abstral	fentanyl	d00233
00093690019	FentaNYL	fentanyl	d00233

00093690045	FentaNYL	fentanyl	d00233
00245042005	FentaNYL	fentanyl	d00233
00245042089	FentaNYL	fentanyl	d00233
00378912116	FentaNYL	fentanyl	d00233
00378912198	FentaNYL	fentanyl	d00233
00406902576	FentaNYL	fentanyl	d00233
00591319854	FentaNYL	fentanyl	d00233
00591319872	FentaNYL	fentanyl	d00233
00591360072	FentaNYL	fentanyl	d00233
00781724155	FentaNYL Matrix	fentanyl	d00233
47781042447	FentaNYL	fentanyl	d00233
49884076152	FentaNYL	fentanyl	d00233
49884076178	FentaNYL	fentanyl	d00233
49999083101	FentaNYL	fentanyl	d00233
49999083105	FentaNYL	fentanyl	d00233
50458009105	Duragesic-25	fentanyl	d00233
51862031452	FentaNYL	fentanyl	d00233
51862031478	FentaNYL	fentanyl	d00233
54868016200	FentaNYL	fentanyl	d00233
54868307400	Duragesic-25	fentanyl	d00233
60505700100	FentaNYL	fentanyl	d00233
60505700102	FentaNYL	fentanyl	d00233
60505700600	FentaNYL	fentanyl	d00233
60505700602	FentaNYL	fentanyl	d00233
60505701100	FentaNYL	fentanyl	d00233
60505701102	FentaNYL	fentanyl	d00233
67767012018	FentaNYL	fentanyl	d00233
42358030032	Abstral	fentanyl	d00233
42747022332	Abstral	fentanyl	d00233
57881033332	Abstral	fentanyl	d00233
13913001301	Lazanda	fentanyl	d00233
00378912598	FentaNYL	fentanyl	d00233
65293001106	Ionsys	fentanyl	d00233
63459054404	Fentora	fentanyl	d00233
63459054428	Fentora	fentanyl	d00233
00093786665	FentaNYL Citrate	fentanyl	d00233
00406920430	FentaNYL Citrate	fentanyl	d00233
49884046055	FentaNYL Citrate	fentanyl	d00233
52959054930	FentaNYL Citrate	fentanyl	d00233
55253007101	FentaNYL Citrate	fentanyl	d00233
55253007130	FentaNYL Citrate	fentanyl	d00233
63459050401	Actiq	fentanyl	d00233
63459050430	Actiq	fentanyl	d00233
20482000410	Subsys	fentanyl	d00233
20482000430	Subsys	fentanyl	d00233

42358040032	Abstral	fentanyl	d00233
42747022432	Abstral	fentanyl	d00233
57881033432	Abstral	fentanyl	d00233
13913001001	Lazanda	fentanyl	d00233
51772031401	Lazanda	fentanyl	d00233
51772031404	Lazanda	fentanyl	d00233
00093690119	FentaNYL	fentanyl	d00233
00093690145	FentaNYL	fentanyl	d00233
00245042105	FentaNYL	fentanyl	d00233
00245042189	FentaNYL	fentanyl	d00233
00378912216	FentaNYL	fentanyl	d00233
00378912298	FentaNYL	fentanyl	d00233
00406905076	FentaNYL	fentanyl	d00233
00591321254	FentaNYL	fentanyl	d00233
00591321272	FentaNYL	fentanyl	d00233
00591360172	FentaNYL	fentanyl	d00233
00781711255	FentaNYL	fentanyl	d00233
00781724255	FentaNYL Matrix	fentanyl	d00233
47781042647	FentaNYL	fentanyl	d00233
49884076252	FentaNYL	fentanyl	d00233
49884076278	FentaNYL	fentanyl	d00233
49999083201	FentaNYL	fentanyl	d00233
49999083205	FentaNYL	fentanyl	d00233
50458009205	Duragesic-50	fentanyl	d00233
54868024400	FentaNYL	fentanyl	d00233
60505700200	FentaNYL	fentanyl	d00233
60505700202	FentaNYL	fentanyl	d00233
60505700700	FentaNYL	fentanyl	d00233
60505700702	FentaNYL	fentanyl	d00233
60505701200	FentaNYL	fentanyl	d00233
60505701202	FentaNYL	fentanyl	d00233
67767012118	FentaNYL	fentanyl	d00233
63459054604	Fentora	fentanyl	d00233
63459054628	Fentora	fentanyl	d00233
00093786765	FentaNYL Citrate	fentanyl	d00233
00406920630	FentaNYL Citrate	fentanyl	d00233
49884046155	FentaNYL Citrate	fentanyl	d00233
52959054830	FentaNYL Citrate	fentanyl	d00233
55253007201	FentaNYL Citrate	fentanyl	d00233
55253007230	FentaNYL Citrate	fentanyl	d00233
63459050601	Actiq	fentanyl	d00233
63459050630	Actiq	fentanyl	d00233
20482000630	Subsys	fentanyl	d00233
20482001215	Subsys	fentanyl	d00233
42358060032	Abstral	fentanyl	d00233

42747022632	Abstral	fentanyl	d00233
57881033632	Abstral	fentanyl	d00233
00378912698	FentaNYL	fentanyl	d00233
00093690219	FentaNYL	fentanyl	d00233
00093690245	FentaNYL	fentanyl	d00233
00245042205	FentaNYL	fentanyl	d00233
00245042289	FentaNYL	fentanyl	d00233
00378912316	FentaNYL	fentanyl	d00233
00378912398	FentaNYL	fentanyl	d00233
00406907576	FentaNYL	fentanyl	d00233
00591321354	FentaNYL	fentanyl	d00233
00591321372	FentaNYL	fentanyl	d00233
00591360272	FentaNYL	fentanyl	d00233
00781724355	FentaNYL Matrix	fentanyl	d00233
47781042747	FentaNYL	fentanyl	d00233
49884076352	FentaNYL	fentanyl	d00233
49884076378	FentaNYL	fentanyl	d00233
49999083301	FentaNYL	fentanyl	d00233
49999083305	FentaNYL	fentanyl	d00233
50458009305	Duragesic-75	fentanyl	d00233
54868028700	FentaNYL	fentanyl	d00233
60505700300	FentaNYL	fentanyl	d00233
60505700302	FentaNYL	fentanyl	d00233
60505700800	FentaNYL	fentanyl	d00233
60505700802	FentaNYL	fentanyl	d00233
60505701300	FentaNYL	fentanyl	d00233
60505701302	FentaNYL	fentanyl	d00233
67767012218	FentaNYL	fentanyl	d00233
63459054804	Fentora	fentanyl	d00233
63459054828	Fentora	fentanyl	d00233
00093786865	FentaNYL Citrate	fentanyl	d00233
00406920830	FentaNYL Citrate	fentanyl	d00233
49884046255	FentaNYL Citrate	fentanyl	d00233
52959055330	FentaNYL Citrate	fentanyl	d00233
55253007301	FentaNYL Citrate	fentanyl	d00233
55253007330	FentaNYL Citrate	fentanyl	d00233
63459050801	Actiq	fentanyl	d00233
63459050830	Actiq	fentanyl	d00233
20482000830	Subsys	fentanyl	d00233
20482001615	Subsys	fentanyl	d00233
42358080032	Abstral	fentanyl	d00233
42747022832	Abstral	fentanyl	d00233
57881033832	Abstral	fentanyl	d00233
00378912798	FentaNYL	fentanyl	d00233
43376021010	Zohydro ER	HYDROcodone	d03075

43376031060	Zohydro ER	HYDROcodone	d03075
65224031060	Zohydro ER	HYDROcodone	d03075
59011027660	Hysingla ER	HYDROcodone	d03075
59011027760	Hysingla ER	HYDROcodone	d03075
43376021510	Zohydro ER	HYDROcodone	d03075
43376031560	Zohydro ER	HYDROcodone	d03075
65224031560	Zohydro ER	HYDROcodone	d03075
43376022010	Zohydro ER	HYDROcodone	d03075
43376032060	Zohydro ER	HYDROcodone	d03075
65224032060	Zohydro ER	HYDROcodone	d03075
59011027160	Hysingla ER	HYDROcodone	d03075
43376023010	Zohydro ER	HYDROcodone	d03075
43376033060	Zohydro ER	HYDROcodone	d03075
65224033060	Zohydro ER	HYDROcodone	d03075
59011027260	Hysingla ER	HYDROcodone	d03075
43376024010	Zohydro ER	HYDROcodone	d03075
43376034060	Zohydro ER	HYDROcodone	d03075
65224034060	Zohydro ER	HYDROcodone	d03075
59011027360	Hysingla ER	HYDROcodone	d03075
43376025010	Zohydro ER	HYDROcodone	d03075
43376035060	Zohydro ER	HYDROcodone	d03075
65224035060	Zohydro ER	HYDROcodone	d03075
59011027460	Hysingla ER	HYDROcodone	d03075
59011027560	Hysingla ER	HYDROcodone	d03075
00603358621	Hydrocodone-Ibuprofen	HYDROcodone	d04225
23710090201	Reprexain	HYDROcodone	d04225
50991057901	Ibudone	HYDROcodone	d04225
51021091030	Xylon 10	HYDROcodone	d04225
53746011701	Hydrocodone-Ibuprofen	HYDROcodone	d04225
60846090201	Reprexain	HYDROcodone	d04225
63717090201	Reprexain	HYDROcodone	d04225
23710090001	Reprexain	HYDROcodone	d04225
53746011601	Hydrocodone-Ibuprofen	HYDROcodone	d04225
60846090001	Reprexain	HYDROcodone	d04225
63717090001	Reprexain	HYDROcodone	d04225
00603358421	Hydrocodone-Ibuprofen	HYDROcodone	d04225
23710090101	Reprexain	HYDROcodone	d04225
50991057801	Ibudone	HYDROcodone	d04225
53746014601	Hydrocodone-Ibuprofen	HYDROcodone	d04225
60846090101	Reprexain	HYDROcodone	d04225
63717090101	Reprexain	HYDROcodone	d04225
00074227712	Vicoprofen	HYDROcodone	d04225
00074227714	Vicoprofen	HYDROcodone	d04225
00074227754	Vicoprofen	HYDROcodone	d04225
00093516101	Hydrocodone-Ibuprofen	HYDROcodone	d04225

00247172700	Vicoprofen	HYDROcodone	d04225
00247172704	Vicoprofen	HYDROcodone	d04225
00247172706	Vicoprofen	HYDROcodone	d04225
00247172720	Vicoprofen	HYDROcodone	d04225
00247172724	Vicoprofen	HYDROcodone	d04225
00247172750	Vicoprofen	HYDROcodone	d04225
00247204106	Hydrocodone-Ibuprofen	HYDROcodone	d04225
00247204112	Hydrocodone-Ibuprofen	HYDROcodone	d04225
00247204120	Hydrocodone-Ibuprofen	HYDROcodone	d04225
00603389721	Hydrocodone-Ibuprofen	HYDROcodone	d04225
00603389728	Hydrocodone-Ibuprofen	HYDROcodone	d04225
13107000401	Hydrocodone-Ibuprofen	HYDROcodone	d04225
21695063120	Hydrocodone-Ibuprofen	HYDROcodone	d04225
23490708600	Hydrocodone-Ibuprofen	HYDROcodone	d04225
23490708602	Hydrocodone-Ibuprofen	HYDROcodone	d04225
23490708603	Hydrocodone-Ibuprofen	HYDROcodone	d04225
43063034920	Hydrocodone-Ibuprofen	HYDROcodone	d04225
43063034930	Hydrocodone-Ibuprofen	HYDROcodone	d04225
43063034960	Hydrocodone-Ibuprofen	HYDROcodone	d04225
49999058801	Hydrocodone-Ibuprofen	HYDROcodone	d04225
49999058815	Hydrocodone-Ibuprofen	HYDROcodone	d04225
49999058830	Hydrocodone-Ibuprofen	HYDROcodone	d04225
49999058860	Hydrocodone-Ibuprofen	HYDROcodone	d04225
49999058890	Hydrocodone-Ibuprofen	HYDROcodone	d04225
52959052212	Vicoprofen	HYDROcodone	d04225
52959052215	Vicoprofen	HYDROcodone	d04225
52959052220	Vicoprofen	HYDROcodone	d04225
52959052230	Vicoprofen	HYDROcodone	d04225
52959052240	Vicoprofen	HYDROcodone	d04225
52959073800	Hydrocodone-Ibuprofen	HYDROcodone	d04225
52959073802	Hydrocodone-Ibuprofen	HYDROcodone	d04225
52959073812	Hydrocodone-Ibuprofen	HYDROcodone	d04225
52959073815	Hydrocodone-Ibuprofen	HYDROcodone	d04225
52959073820	Hydrocodone-Ibuprofen	HYDROcodone	d04225
52959073830	Hydrocodone-Ibuprofen	HYDROcodone	d04225
52959073840	Hydrocodone-Ibuprofen	HYDROcodone	d04225
52959073850	Hydrocodone-Ibuprofen	HYDROcodone	d04225
52959073860	Hydrocodone-Ibuprofen	HYDROcodone	d04225
52959073890	Hydrocodone-Ibuprofen	HYDROcodone	d04225
53746014501	Hydrocodone-Ibuprofen	HYDROcodone	d04225
54569612100	Hydrocodone-Ibuprofen	HYDROcodone	d04225
54868403500	Vicoprofen	HYDROcodone	d04225
54868497600	Hydrocodone-Ibuprofen	HYDROcodone	d04225
55289034815	Vicoprofen	HYDROcodone	d04225
55289094415	Hydrocodone-Ibuprofen	HYDROcodone	d04225

55289094430	Hydrocodone-Ibuprofen	HYDROcodone	d04225
55289094460	Hydrocodone-Ibuprofen	HYDROcodone	d04225
62037052401	Hydrocodone-Ibuprofen	HYDROcodone	d04225
62037052405	Hydrocodone-Ibuprofen	HYDROcodone	d04225
63739055710	Hydrocodone-Ibuprofen	HYDROcodone	d04225
63874110601	Hydrocodone-Ibuprofen	HYDROcodone	d04225
63874110602	Hydrocodone-Ibuprofen	HYDROcodone	d04225
63874110603	Hydrocodone-Ibuprofen	HYDROcodone	d04225
63874110604	Hydrocodone-Ibuprofen	HYDROcodone	d04225
63874110605	Hydrocodone-Ibuprofen	HYDROcodone	d04225
63874110606	Hydrocodone-Ibuprofen	HYDROcodone	d04225
63874110608	Hydrocodone-Ibuprofen	HYDROcodone	d04225
63874110609	Hydrocodone-Ibuprofen	HYDROcodone	d04225
65162014510	Hydrocodone-Ibuprofen	HYDROcodone	d04225
68084022701	Hydrocodone-Ibuprofen	HYDROcodone	d04225
68084022711	Hydrocodone-Ibuprofen	HYDROcodone	d04225
68084084101	Hydrocodone-Ibuprofen	HYDROcodone	d04225
68084084109	Hydrocodone-Ibuprofen	HYDROcodone	d04225
00054038663	HYDROmorphone Hydrochloride	HYDROmorphone	d00255
42858030416	HYDROmorphone Hydrochloride	HYDROmorphone	d00255
42858041616	Dilaudid	HYDROmorphone	d00255
59011045101	Dilaudid-5	HYDROmorphone	d00255
00406331201	HYDROmorphone Hydrochloride	HYDROmorphone	d00255
00574029401	HYDROmorphone Hydrochloride	HYDROmorphone	d00255
00591373901	HYDROmorphone Hydrochloride	HYDROmorphone	d00255
23635041201	Exalgo	HYDROmorphone	d00255
00406331601	HYDROmorphone Hydrochloride	HYDROmorphone	d00255
00574029501	HYDROmorphone Hydrochloride	HYDROmorphone	d00255
00591363001	HYDROmorphone Hydrochloride	HYDROmorphone	d00255
23635041601	Exalgo	HYDROmorphone	d00255
00406324301	HYDROmorphone Hydrochloride	HYDROmorphone	d00255
00527135301	HYDROmorphone Hydrochloride	HYDROmorphone	d00255
13107010701	HYDROmorphone Hydrochloride	HYDROmorphone	d00255
23490777801	HYDROmorphone Hydrochloride	HYDROmorphone	d00255
23490777803	HYDROmorphone Hydrochloride	HYDROmorphone	d00255
23490777806	HYDROmorphone Hydrochloride	HYDROmorphone	d00255
23490777809	HYDROmorphone Hydrochloride	HYDROmorphone	d00255
42858012201	Dilaudid	HYDROmorphone	d00255
42858030101	HYDROmorphone Hydrochloride	HYDROmorphone	d00255
42858030125	HYDROmorphone Hydrochloride	HYDROmorphone	d00255
43063005106	HYDROmorphone Hydrochloride	HYDROmorphone	d00255
52959042002	HYDROmorphone Hydrochloride	HYDROmorphone	d00255
59011045201	Dilaudid	HYDROmorphone	d00255
59011045210	Dilaudid	HYDROmorphone	d00255
63739027510	HYDROmorphone Hydrochloride	HYDROmorphone	d00255

68084042301	HYDROmorphone Hydrochloride	HYDROmorphone	d00255
68084042311	HYDROmorphone Hydrochloride	HYDROmorphone	d00255
00574722406	HYDROmorphone Hydrochloride	HYDROmorphone	d00255
00406333201	HYDROmorphone Hydrochloride ER	HYDROmorphone	d00255
00574029701	HYDROmorphone Hydrochloride ER	HYDROmorphone	d00255
00591363101	HYDROmorphone Hydrochloride ER	HYDROmorphone	d00255
13811070410	HYDROmorphone Hydrochloride ER	HYDROmorphone	d00255
23635043201	Exalgo	HYDROmorphone	d00255
00054026424	HYDROmorphone Hydrochloride	HYDROmorphone	d00255
00054026425	HYDROmorphone Hydrochloride	HYDROmorphone	d00255
00406324401	HYDROmorphone Hydrochloride	HYDROmorphone	d00255
00527135401	HYDROmorphone Hydrochloride	HYDROmorphone	d00255
13107010801	HYDROmorphone Hydrochloride	HYDROmorphone	d00255
23490777903	HYDROmorphone Hydrochloride	HYDROmorphone	d00255
23490777906	HYDROmorphone Hydrochloride	HYDROmorphone	d00255
23490777908	HYDROmorphone Hydrochloride	HYDROmorphone	d00255
23490777909	HYDROmorphone Hydrochloride	HYDROmorphone	d00255
42858023401	Dilaudid	HYDROmorphone	d00255
42858023450	Dilaudid	HYDROmorphone	d00255
42858030201	HYDROmorphone Hydrochloride	HYDROmorphone	d00255
42858030225	HYDROmorphone Hydrochloride	HYDROmorphone	d00255
42858030250	HYDROmorphone Hydrochloride	HYDROmorphone	d00255
49999083501	HYDROmorphone Hydrochloride	HYDROmorphone	d00255
49999083530	HYDROmorphone Hydrochloride	HYDROmorphone	d00255
49999083560	HYDROmorphone Hydrochloride	HYDROmorphone	d00255
49999083590	HYDROmorphone Hydrochloride	HYDROmorphone	d00255
55154161001	Dilaudid	HYDROmorphone	d00255
59011045401	Dilaudid	HYDROmorphone	d00255
59011045405	Dilaudid	HYDROmorphone	d00255
59011045410	Dilaudid	HYDROmorphone	d00255
63739079110	HYDROmorphone Hydrochloride	HYDROmorphone	d00255
68084047201	HYDROmorphone Hydrochloride	HYDROmorphone	d00255
68084047211	HYDROmorphone Hydrochloride	HYDROmorphone	d00255
00054026525	HYDROmorphone Hydrochloride	HYDROmorphone	d00255
00406324901	HYDROmorphone Hydrochloride	HYDROmorphone	d00255
00527135501	HYDROmorphone Hydrochloride	HYDROmorphone	d00255
13107010901	HYDROmorphone Hydrochloride	HYDROmorphone	d00255
42858030301	HYDROmorphone Hydrochloride	HYDROmorphone	d00255
42858033801	Dilaudid	HYDROmorphone	d00255
49999083601	HYDROmorphone Hydrochloride	HYDROmorphone	d00255
49999083690	HYDROmorphone Hydrochloride	HYDROmorphone	d00255
51224010250	HYDROmorphone Hydrochloride	HYDROmorphone	d00255
52959041302	HYDROmorphone Hydrochloride	HYDROmorphone	d00255
52959041360	HYDROmorphone Hydrochloride	HYDROmorphone	d00255
54868459800	HYDROmorphone Hydrochloride	HYDROmorphone	d00255

59011045810	Dilaudid	HYDROmorphone	d00255
68084079821	HYDROmorphone Hydrochloride	HYDROmorphone	d00255
68094085261	HYDROmorphone Hydrochloride	HYDROmorphone	d00255
00406330801	HYDROmorphone Hydrochloride	HYDROmorphone	d00255
00574029301	HYDROmorphone Hydrochloride	HYDROmorphone	d00255
00591362901	HYDROmorphone Hydrochloride	HYDROmorphone	d00255
23635040801	Exalgo	HYDROmorphone	d00255
00228402911	Ibuprofen-Oxycodone Hydrochloride	oxycodone	d05415
00555077802	Ibuprofen-Oxycodone Hydrochloride	oxycodone	d05415
00054043825	Levorphanol Tartrate	levorphanol	d00825
42358010210	Levorphanol Tartrate	levorphanol	d00825
00024033704	Demerol HCl	meperidine	d00017
00024033705	Demerol HCl	meperidine	d00017
00054459625	Meperidine Hydrochloride	meperidine	d00017
00555038202	Meperidine Hydrochloride	meperidine	d00017
00555039202	Meperidine Hydrochloride	meperidine	d00017
00603441621	Meperidine Hydrochloride	meperidine	d00017
30698033701	Demerol HCl	meperidine	d00017
42806005101	Meperidine Hydrochloride	meperidine	d00017
49999083801	Meperidine Hydrochloride	meperidine	d00017
49999083830	Meperidine Hydrochloride	meperidine	d00017
57664047108	Meperidine Hydrochloride	meperidine	d00017
60429053501	Meperidine Hydrochloride	meperidine	d00017
00024033504	Demerol HCl	meperidine	d00017
00024033505	Demerol HCl	meperidine	d00017
00054459525	Meperidine Hydrochloride	meperidine	d00017
00054859511	Meperidine Hydrochloride	meperidine	d00017
00555038102	Meperidine Hydrochloride	meperidine	d00017
00603441521	Meperidine Hydrochloride	meperidine	d00017
23490930003	Meperidine Hydrochloride	meperidine	d00017
23490930006	Meperidine Hydrochloride	meperidine	d00017
23490930009	Meperidine Hydrochloride	meperidine	d00017
42806005001	Meperidine Hydrochloride	meperidine	d00017
49999083730	Meperidine Hydrochloride	meperidine	d00017
54868123301	Meperidine Hydrochloride	meperidine	d00017
54868123302	Meperidine Hydrochloride	meperidine	d00017
54868123303	Meperidine Hydrochloride	meperidine	d00017
55887005130	Meperidine Hydrochloride	meperidine	d00017
57664046708	Meperidine Hydrochloride	meperidine	d00017
60429053401	Meperidine Hydrochloride	meperidine	d00017
68071076030	Meperidine Hydrochloride	meperidine	d00017
00054354563	Meperidine Hydrochloride	meperidine	d00017
51293062201	Meperidine HCl-Promethazine HCl	meperidine	d03433
00054421925	Dolophine	methadone	d00050
00054457125	Methadone Hydrochloride	methadone	d00050

00054855424	Methadone Hydrochloride	methadone	d00050
00406577101	Methadone Hydrochloride	methadone	d00050
00406577162	Methadone Hydrochloride	methadone	d00050
00904653060	Methadone Hydrochloride	methadone	d00050
00904653061	Methadone Hydrochloride	methadone	d00050
13107008901	Methadone Hydrochloride	methadone	d00050
23490587703	Methadone Hydrochloride	methadone	d00050
23490587706	Methadone Hydrochloride	methadone	d00050
23490587707	Methadone Hydrochloride	methadone	d00050
23490587709	Methadone Hydrochloride	methadone	d00050
54868494800	Methadone Hydrochloride	methadone	d00050
54868494801	Methadone Hydrochloride	methadone	d00050
54868494802	Methadone Hydrochloride	methadone	d00050
54868494803	Methadone Hydrochloride	methadone	d00050
54868494804	Methadone Hydrochloride	methadone	d00050
54868494805	Methadone Hydrochloride	methadone	d00050
54868494806	Methadone Hydrochloride	methadone	d00050
55289081430	Methadone Hydrochloride	methadone	d00050
55289081460	Methadone Hydrochloride	methadone	d00050
55289081490	Methadone Hydrochloride	methadone	d00050
55289081493	Methadone Hydrochloride	methadone	d00050
55289081498	Methadone Hydrochloride	methadone	d00050
55289081499	Methadone Hydrochloride	methadone	d00050
55887020001	Methadone Hydrochloride	methadone	d00050
55887020005	Methadone Hydrochloride	methadone	d00050
55887020010	Methadone Hydrochloride	methadone	d00050
55887020020	Methadone Hydrochloride	methadone	d00050
55887020030	Methadone Hydrochloride	methadone	d00050
55887020050	Methadone Hydrochloride	methadone	d00050
63739000610	Methadone Hydrochloride	methadone	d00050
63874126503	Methadone Hydrochloride	methadone	d00050
63874126506	Methadone Hydrochloride	methadone	d00050
66689081010	Methadone Hydrochloride	methadone	d00050
67877011601	Methadone Hydrochloride	methadone	d00050
68084073801	Methadone Hydrochloride	methadone	d00050
00054355663	Methadone Hydrochloride	methadone	d00050
66689071216	Methadone Hydrochloride	methadone	d00050
00054039168	Methadone Hydrochloride	methadone	d00050
00054039268	Methadone Hydrochloride	methadone	d00050
00054355344	Methadone Hydrochloride	methadone	d00050
00406052710	Methadose	methadone	d00050
00406872510	Methadose Sugar-Free	methadone	d00050
66689069430	Methadone Hydrochloride	methadone	d00050
66689069479	Methadone Hydrochloride	methadone	d00050
66689069579	Methadone Hydrochloride	methadone	d00050

00406054034	Methadose	methadone	d00050
00406254001	Methadone Hydrochloride	methadone	d00050
49999084130	Methadose	methadone	d00050
49999084160	Methadose	methadone	d00050
60687020932	Methadose	methadone	d00050
60687020933	Methadose	methadone	d00050
66689089840	Methadone Hydrochloride	methadone	d00050
68084097732	Methadone Hydrochloride	methadone	d00050
68084097733	Methadone Hydrochloride	methadone	d00050
00054421825	Dolophine	methadone	d00050
00054457025	Methadone Hydrochloride	methadone	d00050
00054855324	Methadone Hydrochloride	methadone	d00050
00406575501	Methadone Hydrochloride	methadone	d00050
00406575562	Methadone Hydrochloride	methadone	d00050
00406697434	Methadose	methadone	d00050
13107008801	Methadone Hydrochloride	methadone	d00050
23490587801	Methadone Hydrochloride	methadone	d00050
23490587802	Methadone Hydrochloride	methadone	d00050
23490587803	Methadone Hydrochloride	methadone	d00050
23490587809	Methadone Hydrochloride	methadone	d00050
43063022260	Dolophine	methadone	d00050
43063022290	Dolophine	methadone	d00050
43063022293	Dolophine	methadone	d00050
43063022298	Dolophine	methadone	d00050
49999096330	Methadone Hydrochloride	methadone	d00050
49999096360	Methadone Hydrochloride	methadone	d00050
49999096390	Methadone Hydrochloride	methadone	d00050
52959043502	Methadone Hydrochloride	methadone	d00050
52959043560	Methadone Hydrochloride	methadone	d00050
52959043590	Methadone Hydrochloride	methadone	d00050
54868570100	Methadone Hydrochloride	methadone	d00050
55887009030	Methadone Hydrochloride	methadone	d00050
55887009060	Methadone Hydrochloride	methadone	d00050
60687021401	Methadone Hydrochloride	methadone	d00050
00054355563	Methadone Hydrochloride	methadone	d00050
66689071116	Methadone Hydrochloride	methadone	d00050
70004013282	Morphine Sulfate	morphine	d00308
00574711212	Morphine Sulfate	morphine	d00308
00228350106	Morphine Sulfate ER	morphine	d00308
00228350111	Morphine Sulfate ER	morphine	d00308
00832022500	Morphine Sulfate ER	morphine	d00308
00832022550	Morphine Sulfate ER	morphine	d00308
35356004700	Kadian	morphine	d00308
35356004730	Kadian	morphine	d00308
52544001160	Kadian	morphine	d00308

46987041011	Kadian	morphine	d00308
00054023741	Morphine Sulfate	morphine	d00308
00054023749	Morphine Sulfate	morphine	d00308
00054023755	Morphine Sulfate	morphine	d00308
00054023763	Morphine Sulfate	morphine	d00308
57664035831	Morphine Sulfate	morphine	d00308
57664035835	Morphine Sulfate	morphine	d00308
66689003201	Morphine Sulfate	morphine	d00308
66689003204	Morphine Sulfate	morphine	d00308
66689003216	Morphine Sulfate	morphine	d00308
66689003250	Morphine Sulfate	morphine	d00308
68094000162	Morphine Sulfate	morphine	d00308
00115128201	Morphine Sulfate ER	morphine	d00308
00228350706	Morphine Sulfate ER	morphine	d00308
00228350711	Morphine Sulfate ER	morphine	d00308
00591345301	Morphine Sulfate ER	morphine	d00308
00832023300	Morphine Sulfate ER	morphine	d00308
00832023350	Morphine Sulfate ER	morphine	d00308
35356005200	Kadian	morphine	d00308
35356005230	Kadian	morphine	d00308
49884083801	Morphine Sulfate ER	morphine	d00308
52544016460	Kadian	morphine	d00308
54868457300	Kadian	morphine	d00308
59011026305	MS Contin	morphine	d00308
65597030410	MorphaBond ER	morphine	d00308
46987032411	Kadian	morphine	d00308
49884067001	Morphine Sulfate ER	morphine	d00308
00228432311	Morphine Sulfate ER	morphine	d00308
00378266101	Morphine Sulfate ER	morphine	d00308
00406839001	Morphine Sulfate ER	morphine	d00308
00406839023	Morphine Sulfate ER	morphine	d00308
00406839062	Morphine Sulfate ER	morphine	d00308
00904656061	Morphine Sulfate ER	morphine	d00308
23490791303	Morphine Sulfate ER	morphine	d00308
23490791306	Morphine Sulfate ER	morphine	d00308
23490791309	Morphine Sulfate ER	morphine	d00308
42858079901	MS Contin	morphine	d00308
42858080401	Morphine Sulfate ER	morphine	d00308
43386054301	Morphine Sulfate ER	morphine	d00308
51862018801	Morphine Sulfate ER	morphine	d00308
54868559000	Morphine Sulfate ER	morphine	d00308
55887009490	Morphine Sulfate ER	morphine	d00308
59011026310	MS Contin	morphine	d00308
60951065870	Morphine Sulfate ER	morphine	d00308
63304045201	Morphine Sulfate ER	morphine	d00308

68084016001	Morphine Sulfate ER	morphine	d00308
68084016011	Morphine Sulfate ER	morphine	d00308
68084040601	Morphine Sulfate ER	morphine	d00308
68084040611	Morphine Sulfate ER	morphine	d00308
68382090601	Morphine Sulfate ER	morphine	d00308
00228309311	Morphine Sulfate ER	morphine	d00308
60793060801	AVINza	morphine	d00308
46987032911	Kadian	morphine	d00308
00054023524	Morphine Sulfate	morphine	d00308
00054023525	Morphine Sulfate	morphine	d00308
00172218260	Morphine Sulfate ER	morphine	d00308
43063027230	Morphine Sulfate ER	morphine	d00308
43063027260	Morphine Sulfate ER	morphine	d00308
43063027290	Morphine Sulfate ER	morphine	d00308
63874123803	Morphine Sulfate ER	morphine	d00308
63874123806	Morphine Sulfate ER	morphine	d00308
65597030110	MorphaBond ER	morphine	d00308
68084015701	Morphine Sulfate ER	morphine	d00308
00228427011	Morphine Sulfate ER	morphine	d00308
00378265801	Morphine Sulfate ER	morphine	d00308
00406831501	Morphine Sulfate ER	morphine	d00308
00406831523	Morphine Sulfate ER	morphine	d00308
00406831562	Morphine Sulfate ER	morphine	d00308
00904655761	Morphine Sulfate ER	morphine	d00308
23490779201	Morphine Sulfate ER	morphine	d00308
23490779203	Morphine Sulfate ER	morphine	d00308
23490779206	Morphine Sulfate ER	morphine	d00308
23490779209	Morphine Sulfate ER	morphine	d00308
42858051501	MS Contin	morphine	d00308
42858080101	Morphine Sulfate ER	morphine	d00308
43063068960	Morphine Sulfate ER	morphine	d00308
43063068990	Morphine Sulfate ER	morphine	d00308
43386054001	Morphine Sulfate ER	morphine	d00308
49999084501	Morphine Sulfate ER	morphine	d00308
49999084530	Morphine Sulfate ER	morphine	d00308
49999084560	Morphine Sulfate ER	morphine	d00308
51862018501	Morphine Sulfate ER	morphine	d00308
52959018502	Morphine Sulfate ER	morphine	d00308
52959018530	Morphine Sulfate ER	morphine	d00308
52959018560	Morphine Sulfate ER	morphine	d00308
52959018590	Morphine Sulfate ER	morphine	d00308
54868513200	Morphine Sulfate ER	morphine	d00308
54868513204	Morphine Sulfate ER	morphine	d00308
59011026010	MS Contin	morphine	d00308
60951065270	Morphine Sulfate ER	morphine	d00308

63304045001	Morphine Sulfate ER	morphine	d00308
63739089910	Morphine Sulfate ER	morphine	d00308
68084040301	Morphine Sulfate ER	morphine	d00308
68382090301	Morphine Sulfate ER	morphine	d00308
69344011111	Arymo ER	morphine	d00308
46987033011	Kadian	morphine	d00308
00574711412	Morphine Sulfate	morphine	d00308
00115127701	Morphine Sulfate ER	morphine	d00308
00228350206	Morphine Sulfate ER	morphine	d00308
00228350211	Morphine Sulfate ER	morphine	d00308
00591344901	Morphine Sulfate ER	morphine	d00308
00832022600	Morphine Sulfate ER	morphine	d00308
00832022650	Morphine Sulfate ER	morphine	d00308
35356004800	Kadian	morphine	d00308
35356004830	Kadian	morphine	d00308
49884066501	Morphine Sulfate ER	morphine	d00308
49884083301	Morphine Sulfate ER	morphine	d00308
52544021160	Kadian	morphine	d00308
54868457100	Kadian	morphine	d00308
46987032211	Kadian	morphine	d00308
00054023849	Morphine Sulfate	morphine	d00308
00054023863	Morphine Sulfate	morphine	d00308
57664035931	Morphine Sulfate	morphine	d00308
57664035935	Morphine Sulfate	morphine	d00308
66689003301	Morphine Sulfate	morphine	d00308
66689003304	Morphine Sulfate	morphine	d00308
66689003316	Morphine Sulfate	morphine	d00308
66689003350	Morphine Sulfate	morphine	d00308
00054040441	Morphine Sulfate	morphine	d00308
00054040444	Morphine Sulfate	morphine	d00308
00054040450	Morphine Sulfate	morphine	d00308
00054051741	Morphine Sulfate	morphine	d00308
00054051744	Morphine Sulfate	morphine	d00308
00054051750	Morphine Sulfate	morphine	d00308
00406800312	Morphine Sulfate	morphine	d00308
00406800315	Morphine Sulfate	morphine	d00308
00406800324	Morphine Sulfate	morphine	d00308
00406800330	Morphine Sulfate	morphine	d00308
00527142535	Morphine Sulfate	morphine	d00308
00527142536	Morphine Sulfate	morphine	d00308
00527142562	Morphine Sulfate	morphine	d00308
00527142563	Morphine Sulfate	morphine	d00308
00527190635	Morphine Sulfate	morphine	d00308
00527190636	Morphine Sulfate	morphine	d00308
00527190662	Morphine Sulfate	morphine	d00308

00527190663	Morphine Sulfate	morphine	d00308
00574012730	Morphine Sulfate	morphine	d00308
00574015312	Morphine Sulfate	morphine	d00308
00574015330	Morphine Sulfate	morphine	d00308
27808008201	Morphine Sulfate	morphine	d00308
27808008202	Morphine Sulfate	morphine	d00308
68094075458	Morphine Sulfate	morphine	d00308
68094075558	Morphine Sulfate	morphine	d00308
52544022060	Kadian	morphine	d00308
46987037711	Kadian	morphine	d00308
00228434711	Morphine Sulfate ER	morphine	d00308
00378266201	Morphine Sulfate ER	morphine	d00308
00406832001	Morphine Sulfate ER	morphine	d00308
23490791403	Morphine Sulfate ER	morphine	d00308
23490791406	Morphine Sulfate ER	morphine	d00308
23490791409	Morphine Sulfate ER	morphine	d00308
42858080501	Morphine Sulfate ER	morphine	d00308
42858090001	MS Contin	morphine	d00308
43386054401	Morphine Sulfate ER	morphine	d00308
59011026410	MS Contin	morphine	d00308
60951065970	Morphine Sulfate ER	morphine	d00308
63304045301	Morphine Sulfate ER	morphine	d00308
68382090701	Morphine Sulfate ER	morphine	d00308
00054023624	Morphine Sulfate	morphine	d00308
00054023625	Morphine Sulfate	morphine	d00308
52959018202	Morphine Sulfate	morphine	d00308
52959018230	Morphine Sulfate	morphine	d00308
52959018260	Morphine Sulfate	morphine	d00308
52959018290	Morphine Sulfate	morphine	d00308
54868497302	Morphine Sulfate	morphine	d00308
55289086330	Morphine Sulfate	morphine	d00308
55289086360	Morphine Sulfate	morphine	d00308
55289086390	Morphine Sulfate	morphine	d00308
00574711612	Morphine Sulfate	morphine	d00308
00115127801	Morphine Sulfate ER	morphine	d00308
00228350306	Morphine Sulfate ER	morphine	d00308
00228350311	Morphine Sulfate ER	morphine	d00308
00591345001	Morphine Sulfate ER	morphine	d00308
00832022700	Morphine Sulfate ER	morphine	d00308
35356004900	Kadian	morphine	d00308
35356004930	Kadian	morphine	d00308
46987032511	Kadian	morphine	d00308
49884083401	Morphine Sulfate ER	morphine	d00308
52544003260	Kadian	morphine	d00308
54868498100	Kadian	morphine	d00308

43063027430	Morphine Sulfate ER	morphine	d00308
43063027460	Morphine Sulfate ER	morphine	d00308
43063027490	Morphine Sulfate ER	morphine	d00308
59011026105	MS Contin	morphine	d00308
65597030210	MorphaBond ER	morphine	d00308
68084015801	Morphine Sulfate ER	morphine	d00308
00228309011	Morphine Sulfate ER	morphine	d00308
00832022750	Morphine Sulfate ER	morphine	d00308
35356023830	AVINza	morphine	d00308
49884066601	Morphine Sulfate ER	morphine	d00308
60793060501	AVINza	morphine	d00308
00228427111	Morphine Sulfate ER	morphine	d00308
00378265901	Morphine Sulfate ER	morphine	d00308
00406833001	Morphine Sulfate ER	morphine	d00308
00406833062	Morphine Sulfate ER	morphine	d00308
00904655861	Morphine Sulfate ER	morphine	d00308
23490777701	Morphine Sulfate ER	morphine	d00308
23490777703	Morphine Sulfate ER	morphine	d00308
23490777706	Morphine Sulfate ER	morphine	d00308
23490777709	Morphine Sulfate ER	morphine	d00308
42858063101	MS Contin	morphine	d00308
42858080201	Morphine Sulfate ER	morphine	d00308
43063068830	Morphine Sulfate ER	morphine	d00308
43063068860	Morphine Sulfate ER	morphine	d00308
43063068890	Morphine Sulfate ER	morphine	d00308
43386054101	Morphine Sulfate ER	morphine	d00308
49999084601	Morphine Sulfate ER	morphine	d00308
49999084630	Morphine Sulfate ER	morphine	d00308
49999084660	Morphine Sulfate ER	morphine	d00308
51862018601	Morphine Sulfate ER	morphine	d00308
52959040602	Morphine Sulfate ER	morphine	d00308
52959040630	Morphine Sulfate ER	morphine	d00308
52959040660	Morphine Sulfate ER	morphine	d00308
52959040690	Morphine Sulfate ER	morphine	d00308
55887006760	Morphine Sulfate ER	morphine	d00308
55887009260	Morphine Sulfate ER	morphine	d00308
55887009290	Morphine Sulfate ER	morphine	d00308
59011026125	MS Contin	morphine	d00308
60951065370	Morphine Sulfate ER	morphine	d00308
63304045101	Morphine Sulfate ER	morphine	d00308
63739072610	Morphine Sulfate ER	morphine	d00308
63874123403	Morphine Sulfate ER	morphine	d00308
63874123406	Morphine Sulfate ER	morphine	d00308
68084040401	Morphine Sulfate ER	morphine	d00308
68382090401	Morphine Sulfate ER	morphine	d00308

69344021111	Arymo ER	morphine	d00308
46987032711	Kadian	morphine	d00308
52544003960	Kadian	morphine	d00308
00228311611	Morphine Sulfate ER	morphine	d00308
60793060301	AVINza	morphine	d00308
00574711012	Morphine Sulfate	morphine	d00308
00115127901	Morphine Sulfate ER	morphine	d00308
00228350406	Morphine Sulfate ER	morphine	d00308
00228350411	Morphine Sulfate ER	morphine	d00308
00591345101	Morphine Sulfate ER	morphine	d00308
00832022800	Morphine Sulfate ER	morphine	d00308
00832022850	Morphine Sulfate ER	morphine	d00308
35356005000	Kadian	morphine	d00308
35356005030	Kadian	morphine	d00308
49884083501	Morphine Sulfate ER	morphine	d00308
52544005260	Kadian	morphine	d00308
54868457200	Kadian	morphine	d00308
46987032311	Kadian	morphine	d00308
49884066701	Morphine Sulfate ER	morphine	d00308
00115128001	Morphine Sulfate ER	morphine	d00308
00228350506	Morphine Sulfate ER	morphine	d00308
00228350511	Morphine Sulfate ER	morphine	d00308
00591345201	Morphine Sulfate ER	morphine	d00308
00832022900	Morphine Sulfate ER	morphine	d00308
35356005100	Kadian	morphine	d00308
35356005130	Kadian	morphine	d00308
35356005160	Kadian	morphine	d00308
46987032611	Kadian	morphine	d00308
49884083601	Morphine Sulfate ER	morphine	d00308
52544006360	Kadian	morphine	d00308
54868585000	Kadian	morphine	d00308
43063027560	Morphine Sulfate ER	morphine	d00308
43063027590	Morphine Sulfate ER	morphine	d00308
43063027598	Morphine Sulfate ER	morphine	d00308
59011026205	MS Contin	morphine	d00308
65597030310	MorphaBond ER	morphine	d00308
00228309111	Morphine Sulfate ER	morphine	d00308
00832022950	Morphine Sulfate ER	morphine	d00308
35356011830	AVINza	morphine	d00308
49884066801	Morphine Sulfate ER	morphine	d00308
60793060601	AVINza	morphine	d00308
00228431111	Morphine Sulfate ER	morphine	d00308
00378266001	Morphine Sulfate ER	morphine	d00308
00406838001	Morphine Sulfate ER	morphine	d00308
00406838062	Morphine Sulfate ER	morphine	d00308

00904655961	Morphine Sulfate ER	morphine	d00308
23490791203	Morphine Sulfate ER	morphine	d00308
23490791206	Morphine Sulfate ER	morphine	d00308
23490791209	Morphine Sulfate ER	morphine	d00308
42858076001	MS Contin	morphine	d00308
42858080301	Morphine Sulfate ER	morphine	d00308
43063068430	Morphine Sulfate ER	morphine	d00308
43063068460	Morphine Sulfate ER	morphine	d00308
43063068490	Morphine Sulfate ER	morphine	d00308
43386054201	Morphine Sulfate ER	morphine	d00308
49999084701	Morphine Sulfate ER	morphine	d00308
49999084730	Morphine Sulfate ER	morphine	d00308
49999084760	Morphine Sulfate ER	morphine	d00308
49999084790	Morphine Sulfate ER	morphine	d00308
51862018701	Morphine Sulfate ER	morphine	d00308
59011026210	MS Contin	morphine	d00308
60951065570	Morphine Sulfate ER	morphine	d00308
63304075801	Morphine Sulfate ER	morphine	d00308
63874123503	Morphine Sulfate ER	morphine	d00308
63874123506	Morphine Sulfate ER	morphine	d00308
68084040501	Morphine Sulfate ER	morphine	d00308
68382090501	Morphine Sulfate ER	morphine	d00308
69344031111	Arymo ER	morphine	d00308
46987032811	Kadian	morphine	d00308
00228311711	Morphine Sulfate ER	morphine	d00308
60793060401	AVINza	morphine	d00308
00115128101	Morphine Sulfate ER	morphine	d00308
00228350606	Morphine Sulfate ER	morphine	d00308
00228350611	Morphine Sulfate ER	morphine	d00308
00591357601	Morphine Sulfate ER	morphine	d00308
00832023000	Morphine Sulfate ER	morphine	d00308
00832023050	Morphine Sulfate ER	morphine	d00308
35356007600	Kadian	morphine	d00308
35356007630	Kadian	morphine	d00308
46987041211	Kadian	morphine	d00308
49884066901	Morphine Sulfate ER	morphine	d00308
49884083701	Morphine Sulfate ER	morphine	d00308
52544089660	Kadian	morphine	d00308
00228309211	Morphine Sulfate ER	morphine	d00308
60793060701	AVINza	morphine	d00308
60793043701	Embeda	morphine	d07472
60793043720	Embeda	morphine	d07472
16590089730	Embeda	morphine	d07472
16590089760	Embeda	morphine	d07472
16590089790	Embeda	morphine	d07472

60793043001	Embeda	morphine	d07472
60793043020	Embeda	morphine	d07472
60793043101	Embeda	morphine	d07472
60793043120	Embeda	morphine	d07472
60793043301	Embeda	morphine	d07472
60793043320	Embeda	morphine	d07472
60793043401	Embeda	morphine	d07472
60793043420	Embeda	morphine	d07472
60793043501	Embeda	morphine	d07472
60793043520	Embeda	morphine	d07472
50383085516	Paregoric	morphine equivalent	d00824
00187420304	Opium Deodorized	morphine equivalent	d00824
00187420316	Opium Deodorized	morphine equivalent	d00824
42799021701	Opium	morphine equivalent	d00824
42799021702	Opium	morphine equivalent	d00824
42998020301	Opium Deodorized	morphine equivalent	d00824
42998020302	Opium Deodorized	morphine equivalent	d00824
62559015304	Opium	morphine equivalent	d00824
00603499321	OxyCODONE Hydrochloride	oxyCODONE	d00329
00904659061	OxyCODONE Hydrochloride	oxyCODONE	d00329
10702005601	OxyCODONE Hydrochloride	oxyCODONE	d00329
10702005650	OxyCODONE Hydrochloride	oxyCODONE	d00329
35356021160	OxyCODONE Hydrochloride	oxyCODONE	d00329
42806000601	OxyCODONE Hydrochloride	oxyCODONE	d00329
42858000201	OxyCODONE Hydrochloride	oxyCODONE	d00329
42858000210	OxyCODONE Hydrochloride	oxyCODONE	d00329
43063051230	OxyCODONE Hydrochloride	oxyCODONE	d00329
43063051260	OxyCODONE Hydrochloride	oxyCODONE	d00329
43063051290	OxyCODONE Hydrochloride	oxyCODONE	d00329
43063051293	OxyCODONE Hydrochloride	oxyCODONE	d00329
43063051298	OxyCODONE Hydrochloride	oxyCODONE	d00329
43063051299	OxyCODONE Hydrochloride	oxyCODONE	d00329
54868313700	OxyCODONE Hydrochloride	oxyCODONE	d00329
57664037088	OxyCODONE Hydrochloride	oxyCODONE	d00329
60429051701	OxyCODONE Hydrochloride	oxyCODONE	d00329
63739017710	OxyCODONE Hydrochloride	oxyCODONE	d00329
65162004810	OxyCODONE Hydrochloride	oxyCODONE	d00329
68084004801	OxyCODONE Hydrochloride	oxyCODONE	d00329
68382079401	OxyCODONE Hydrochloride	oxyCODONE	d00329
00093573101	OxyCODONE Hydrochloride ER	oxyCODONE	d00329
00115155601	OxyCODONE Hydrochloride ER	oxyCODONE	d00329
00781570301	OxyCODONE Hydrochloride ER	oxyCODONE	d00329
43063035402	OxyCONTIN	oxyCODONE	d00329
43063035410	OxyCONTIN	oxyCODONE	d00329
49884013601	OxyCODONE Hydrochloride ER	oxyCODONE	d00329

59011041010	OxyCONTIN	oxyCODONE	d00329
59011041020	OxyCONTIN	oxyCODONE	d00329
24510011510	Xtampza ER	oxyCODONE	d00329
00228287811	OxyCODONE Hydrochloride	oxyCODONE	d00329
00378611301	OxyCODONE Hydrochloride	oxyCODONE	d00329
00406851501	OxyCODONE Hydrochloride	oxyCODONE	d00329
00406851562	OxyCODONE Hydrochloride	oxyCODONE	d00329
00603499121	OxyCODONE Hydrochloride	oxyCODONE	d00329
00603499128	OxyCODONE Hydrochloride	oxyCODONE	d00329
00904644561	OxyCODONE Hydrochloride	oxyCODONE	d00329
10702000801	OxyCODONE Hydrochloride	oxyCODONE	d00329
10702000850	OxyCODONE Hydrochloride	oxyCODONE	d00329
13107005601	OxyCODONE Hydrochloride	oxyCODONE	d00329
23490781902	OxyCODONE Hydrochloride	oxyCODONE	d00329
23490781903	OxyCODONE Hydrochloride	oxyCODONE	d00329
23490781906	OxyCODONE Hydrochloride	oxyCODONE	d00329
23490781908	OxyCODONE Hydrochloride	oxyCODONE	d00329
23490781909	OxyCODONE Hydrochloride	oxyCODONE	d00329
23635058110	Roxicodone	oxyCODONE	d00329
31722091701	OxyCODONE Hydrochloride	oxycodone	d00329
31722091705	OxyCODONE Hydrochloride	oxycodone	d00329
42806000701	OxyCODONE Hydrochloride	oxyCODONE	d00329
42858000301	OxyCODONE Hydrochloride	oxyCODONE	d00329
43063021910	OxyCODONE Hydrochloride	oxyCODONE	d00329
43063021930	OxyCODONE Hydrochloride	oxyCODONE	d00329
43063021960	OxyCODONE Hydrochloride	oxyCODONE	d00329
43063021990	OxyCODONE Hydrochloride	oxyCODONE	d00329
43063021993	OxyCODONE Hydrochloride	oxyCODONE	d00329
43063021998	OxyCODONE Hydrochloride	oxyCODONE	d00329
47781026401	OxyCODONE Hydrochloride	oxyCODONE	d00329
47781026405	OxyCODONE Hydrochloride	oxyCODONE	d00329
49999085001	OxyCODONE Hydrochloride	oxyCODONE	d00329
49999085005	OxyCODONE Hydrochloride	oxyCODONE	d00329
49999085030	OxyCODONE Hydrochloride	oxyCODONE	d00329
49999085060	OxyCODONE Hydrochloride	oxyCODONE	d00329
49999085830	Roxicodone	oxyCODONE	d00329
49999085860	Roxicodone	oxyCODONE	d00329
49999085890	Roxicodone	oxyCODONE	d00329
52959012902	OxyCODONE Hydrochloride	oxyCODONE	d00329
52959012930	OxyCODONE Hydrochloride	oxyCODONE	d00329
52959012960	OxyCODONE Hydrochloride	oxyCODONE	d00329
52959012990	OxyCODONE Hydrochloride	oxyCODONE	d00329
54868472200	Roxicodone	oxyCODONE	d00329
54868498000	OxyCODONE Hydrochloride	oxyCODONE	d00329
55289096793	OxyCODONE Hydrochloride	oxyCODONE	d00329

55887013301	OxyCODONE Hydrochloride	oxyCODONE	d00329
55887013310	OxyCODONE Hydrochloride	oxyCODONE	d00329
55887013320	OxyCODONE Hydrochloride	oxyCODONE	d00329
55887013328	OxyCODONE Hydrochloride	oxyCODONE	d00329
55887013350	OxyCODONE Hydrochloride	oxyCODONE	d00329
57664018788	OxyCODONE Hydrochloride	oxyCODONE	d00329
60429051901	OxyCODONE Hydrochloride	oxyCODONE	d00329
63874126702	OxyCODONE Hydrochloride	oxyCODONE	d00329
63874126703	OxyCODONE Hydrochloride	oxyCODONE	d00329
63874126706	OxyCODONE Hydrochloride	oxyCODONE	d00329
64720022510	OxyCODONE Hydrochloride	oxyCODONE	d00329
65162004910	OxyCODONE Hydrochloride	oxyCODONE	d00329
65162004950	OxyCODONE Hydrochloride	oxyCODONE	d00329
66479058110	Roxicodone	oxyCODONE	d00329
68084018401	OxyCODONE Hydrochloride	oxyCODONE	d00329
68084018411	OxyCODONE Hydrochloride	oxyCODONE	d00329
68094000561	OxyCODONE Hydrochloride	oxyCODONE	d00329
68308011101	OxyCODONE Hydrochloride	oxyCODONE	d00329
68382079501	OxyCODONE Hydrochloride	oxyCODONE	d00329
00115155701	OxyCODONE Hydrochloride ER	oxyCODONE	d00329
59011041510	OxyCONTIN	oxyCODONE	d00329
59011041520	OxyCONTIN	oxyCODONE	d00329
24510012010	Xtampza ER	oxyCODONE	d00329
00603499421	OxyCODONE Hydrochloride	oxyCODONE	d00329
10702005701	OxyCODONE Hydrochloride	oxyCODONE	d00329
10702005750	OxyCODONE Hydrochloride	oxyCODONE	d00329
42806000901	OxyCODONE Hydrochloride	oxyCODONE	d00329
42858000401	OxyCODONE Hydrochloride	oxyCODONE	d00329
43063051130	OxyCODONE Hydrochloride	oxyCODONE	d00329
43063051160	OxyCODONE Hydrochloride	oxyCODONE	d00329
43063051190	OxyCODONE Hydrochloride	oxyCODONE	d00329
43063051193	OxyCODONE Hydrochloride	oxyCODONE	d00329
43063051198	OxyCODONE Hydrochloride	oxyCODONE	d00329
43063051199	OxyCODONE Hydrochloride	oxyCODONE	d00329
54868590200	OxyCODONE Hydrochloride	oxyCODONE	d00329
57664037188	OxyCODONE Hydrochloride	oxyCODONE	d00329
60429052001	OxyCODONE Hydrochloride	oxyCODONE	d00329
65162005010	OxyCODONE Hydrochloride	oxyCODONE	d00329
68084082821	OxyCODONE Hydrochloride	oxyCODONE	d00329
68382079601	OxyCODONE Hydrochloride	oxyCODONE	d00329
00093573201	OxyCODONE Hydrochloride ER	oxyCODONE	d00329
00115155801	OxyCODONE Hydrochloride ER	oxyCODONE	d00329
00781572601	OxyCODONE Hydrochloride ER	oxyCODONE	d00329
16590061690	OxyCONTIN	oxyCODONE	d00329
49884013701	OxyCODONE Hydrochloride ER	oxyCODONE	d00329

52959014860	OxyCONTIN	oxyCODONE	d00329
59011042010	OxyCONTIN	oxyCODONE	d00329
59011042020	OxyCONTIN	oxyCODONE	d00329
00054039344	OxyCODONE Hydrochloride	oxyCODONE	d00329
00054052244	OxyCODONE Hydrochloride	oxyCODONE	d00329
00527142635	OxyCODONE Hydrochloride	oxyCODONE	d00329
00527142636	OxyCODONE Hydrochloride	oxyCODONE	d00329
43386092060	OxyCODONE Hydrochloride	oxyCODONE	d00329
59011022520	Oxyfast	oxyCODONE	d00329
62559015230	OxyCODONE Hydrochloride	oxyCODONE	d00329
66689002530	OxyCODONE Hydrochloride	oxyCODONE	d00329
68094080101	OxyCODONE Hydrochloride	oxyCODONE	d00329
68094080158	OxyCODONE Hydrochloride	oxyCODONE	d00329
68308002003	OxyCODONE Hydrochloride	oxyCODONE	d00329
68462034737	OxyCODONE Hydrochloride	oxyCODONE	d00329
24510013010	Xtampza ER	oxyCODONE	d00329
00228287911	OxyCODONE Hydrochloride	oxyCODONE	d00329
00378611401	OxyCODONE Hydrochloride	oxyCODONE	d00329
00406853001	OxyCODONE Hydrochloride	oxyCODONE	d00329
00406853062	OxyCODONE Hydrochloride	oxyCODONE	d00329
00603499221	OxyCODONE Hydrochloride	oxyCODONE	d00329
00603499228	OxyCODONE Hydrochloride	oxyCODONE	d00329
00904659161	OxyCODONE Hydrochloride	oxyCODONE	d00329
10702000901	OxyCODONE Hydrochloride	oxyCODONE	d00329
10702000950	OxyCODONE Hydrochloride	oxyCODONE	d00329
13107005701	OxyCODONE Hydrochloride	oxyCODONE	d00329
23490782002	OxyCODONE Hydrochloride	oxyCODONE	d00329
23490782003	OxyCODONE Hydrochloride	oxyCODONE	d00329
23490782006	OxyCODONE Hydrochloride	oxyCODONE	d00329
23490782008	OxyCODONE Hydrochloride	oxyCODONE	d00329
23490782009	OxyCODONE Hydrochloride	oxyCODONE	d00329
23635058210	Roxicodone	oxyCODONE	d00329
31722091801	OxyCODONE Hydrochloride	oxycodone	d00329
31722091805	OxyCODONE Hydrochloride	oxycodone	d00329
42806000801	OxyCODONE Hydrochloride	oxyCODONE	d00329
42858000501	OxyCODONE Hydrochloride	oxyCODONE	d00329
43063022010	OxyCODONE Hydrochloride	oxyCODONE	d00329
43063022030	OxyCODONE Hydrochloride	oxyCODONE	d00329
43063022060	OxyCODONE Hydrochloride	oxyCODONE	d00329
43063022077	OxyCODONE Hydrochloride	oxyCODONE	d00329
43063022090	OxyCODONE Hydrochloride	oxyCODONE	d00329
43063022093	OxyCODONE Hydrochloride	oxyCODONE	d00329
43063022098	OxyCODONE Hydrochloride	oxyCODONE	d00329
47781026501	OxyCODONE Hydrochloride	oxyCODONE	d00329
47781026505	OxyCODONE Hydrochloride	oxyCODONE	d00329

49999085101	OxyCODONE Hydrochloride	oxyCODONE	d00329
49999085105	OxyCODONE Hydrochloride	oxyCODONE	d00329
49999085130	OxyCODONE Hydrochloride	oxyCODONE	d00329
49999085160	OxyCODONE Hydrochloride	oxyCODONE	d00329
49999085930	Roxicodone	oxyCODONE	d00329
49999085960	Roxicodone	oxyCODONE	d00329
52959013002	OxyCODONE Hydrochloride	oxyCODONE	d00329
52959013030	OxyCODONE Hydrochloride	oxyCODONE	d00329
52959013060	OxyCODONE Hydrochloride	oxyCODONE	d00329
52959013090	OxyCODONE Hydrochloride	oxyCODONE	d00329
54868478300	Roxicodone	oxyCODONE	d00329
54868539000	OxyCODONE Hydrochloride	oxyCODONE	d00329
55289051193	OxyCODONE Hydrochloride	oxyCODONE	d00329
55887013201	OxyCODONE Hydrochloride	oxyCODONE	d00329
55887013202	OxyCODONE Hydrochloride	oxyCODONE	d00329
55887013210	OxyCODONE Hydrochloride	oxyCODONE	d00329
55887013220	OxyCODONE Hydrochloride	oxyCODONE	d00329
55887013250	OxyCODONE Hydrochloride	oxyCODONE	d00329
57664022488	OxyCODONE Hydrochloride	oxyCODONE	d00329
60429052101	OxyCODONE Hydrochloride	oxyCODONE	d00329
63874126602	OxyCODONE Hydrochloride	oxyCODONE	d00329
63874126603	OxyCODONE Hydrochloride	oxyCODONE	d00329
63874126606	OxyCODONE Hydrochloride	oxyCODONE	d00329
64720022610	OxyCODONE Hydrochloride	oxyCODONE	d00329
65162005110	OxyCODONE Hydrochloride	oxyCODONE	d00329
65162005150	OxyCODONE Hydrochloride	oxyCODONE	d00329
66479058210	Roxicodone	oxyCODONE	d00329
68071045460	OxyCODONE Hydrochloride	oxyCODONE	d00329
68084018501	OxyCODONE Hydrochloride	oxyCODONE	d00329
68084018511	OxyCODONE Hydrochloride	oxyCODONE	d00329
68094000659	OxyCODONE Hydrochloride	oxyCODONE	d00329
68094000661	OxyCODONE Hydrochloride	oxyCODONE	d00329
68308011201	OxyCODONE Hydrochloride	oxyCODONE	d00329
68382079701	OxyCODONE Hydrochloride	oxyCODONE	d00329
00115155901	OxyCODONE Hydrochloride ER	oxyCODONE	d00329
59011043010	OxyCONTIN	oxyCODONE	d00329
59011043020	OxyCONTIN	oxyCODONE	d00329
24510014010	Xtampza ER	oxyCODONE	d00329
00093573301	OxyCODONE Hydrochloride ER	oxyCODONE	d00329
00115156001	OxyCODONE Hydrochloride ER	oxyCODONE	d00329
00591269301	OxyCODONE Hydrochloride ER	oxyCODONE	d00329
00781576701	OxyCODONE Hydrochloride ER	oxyCODONE	d00329
35356039930	OxyCONTIN	oxyCODONE	d00329
49884013801	OxyCODONE Hydrochloride ER	oxyCODONE	d00329
49999085630	OxyCONTIN	oxyCODONE	d00329

52959039660	OxyCONTIN	oxyCODONE	d00329
59011044010	OxyCONTIN	oxyCODONE	d00329
59011044020	OxyCONTIN	oxyCODONE	d00329
63304068301	OxyCODONE Hydrochloride ER	oxyCODONE	d00329
00527177401	OxyCODONE Hydrochloride	oxyCODONE	d00329
10702002301	OxyCODONE Hydrochloride	oxyCODONE	d00329
43386095001	OxyCODONE Hydrochloride	oxyCODONE	d00329
60687011611	OxyCODONE Hydrochloride	oxyCODONE	d00329
60687011621	OxyCODONE Hydrochloride	oxyCODONE	d00329
62559016701	OxyCODONE Hydrochloride	oxyCODONE	d00329
68308014501	OxyCODONE Hydrochloride	oxyCODONE	d00329
68462020401	OxyCODONE Hydrochloride	oxyCODONE	d00329
00228287611	OxyCODONE Hydrochloride	oxyCODONE	d00329
00378611201	OxyCODONE Hydrochloride	oxyCODONE	d00329
00406055201	OxyCODONE Hydrochloride	oxyCODONE	d00329
00406055262	OxyCODONE Hydrochloride	oxyCODONE	d00329
00603499021	OxyCODONE Hydrochloride	oxyCODONE	d00329
00603499028	OxyCODONE Hydrochloride	oxyCODONE	d00329
00904644461	OxyCODONE Hydrochloride	oxyCODONE	d00329
10702001801	OxyCODONE Hydrochloride	oxyCODONE	d00329
10702001850	OxyCODONE Hydrochloride	oxyCODONE	d00329
13107005501	OxyCODONE Hydrochloride	oxyCODONE	d00329
23490778101	OxyCODONE Hydrochloride	oxyCODONE	d00329
23490778102	OxyCODONE Hydrochloride	oxyCODONE	d00329
23490778103	OxyCODONE Hydrochloride	oxyCODONE	d00329
23490778106	OxyCODONE Hydrochloride	oxyCODONE	d00329
23490778108	OxyCODONE Hydrochloride	oxyCODONE	d00329
23490778109	OxyCODONE Hydrochloride	oxyCODONE	d00329
23635058010	Roxicodone	oxyCODONE	d00329
42806000501	OxyCODONE Hydrochloride	oxyCODONE	d00329
42858000101	OxyCODONE Hydrochloride	oxyCODONE	d00329
42858000110	OxyCODONE Hydrochloride	oxyCODONE	d00329
43063030406	OxyCODONE Hydrochloride	oxyCODONE	d00329
43063030415	OxyCODONE Hydrochloride	oxyCODONE	d00329
43063030430	OxyCODONE Hydrochloride	oxyCODONE	d00329
43063030460	OxyCODONE Hydrochloride	oxyCODONE	d00329
43063030490	OxyCODONE Hydrochloride	oxyCODONE	d00329
43063030493	OxyCODONE Hydrochloride	oxyCODONE	d00329
43063030498	OxyCODONE Hydrochloride	oxyCODONE	d00329
43063030499	OxyCODONE Hydrochloride	oxyCODONE	d00329
43063063110	OxyCODONE Hydrochloride	oxyCODONE	d00329
43063063120	OxyCODONE Hydrochloride	oxyCODONE	d00329
43063063130	OxyCODONE Hydrochloride	oxyCODONE	d00329
43063063140	OxyCODONE Hydrochloride	oxyCODONE	d00329
43063063156	OxyCODONE Hydrochloride	oxyCODONE	d00329

43063063160	OxyCODONE Hydrochloride	oxyCODONE	d00329
43063063188	OxyCODONE Hydrochloride	oxyCODONE	d00329
43063063190	OxyCODONE Hydrochloride	oxyCODONE	d00329
43063063193	OxyCODONE Hydrochloride	oxyCODONE	d00329
43063063198	OxyCODONE Hydrochloride	oxyCODONE	d00329
43063063199	OxyCODONE Hydrochloride	oxyCODONE	d00329
47781026301	OxyCODONE Hydrochloride	oxyCODONE	d00329
47781026305	OxyCODONE Hydrochloride	oxyCODONE	d00329
49999089930	OxyCODONE Hydrochloride	oxyCODONE	d00329
49999089960	OxyCODONE Hydrochloride	oxyCODONE	d00329
49999089990	OxyCODONE Hydrochloride	oxyCODONE	d00329
52959013102	OxyCODONE Hydrochloride	oxyCODONE	d00329
52959013130	OxyCODONE Hydrochloride	oxyCODONE	d00329
52959013150	OxyCODONE Hydrochloride	oxyCODONE	d00329
52959013160	OxyCODONE Hydrochloride	oxyCODONE	d00329
52959013190	OxyCODONE Hydrochloride	oxyCODONE	d00329
54868498300	OxyCODONE Hydrochloride	oxyCODONE	d00329
54868498301	OxyCODONE Hydrochloride	oxyCODONE	d00329
54868498303	OxyCODONE Hydrochloride	oxyCODONE	d00329
55289089130	OxyCODONE Hydrochloride	oxyCODONE	d00329
55289089160	OxyCODONE Hydrochloride	oxyCODONE	d00329
55289089190	OxyCODONE Hydrochloride	oxyCODONE	d00329
55289089193	OxyCODONE Hydrochloride	oxyCODONE	d00329
55289089198	OxyCODONE Hydrochloride	oxyCODONE	d00329
55289089199	OxyCODONE Hydrochloride	oxyCODONE	d00329
55887005001	OxyCODONE Hydrochloride	oxyCODONE	d00329
57664022388	OxyCODONE Hydrochloride	oxyCODONE	d00329
60429051601	OxyCODONE Hydrochloride	oxyCODONE	d00329
60793052501	Oxecta	oxyCODONE	d00329
63739036510	OxyCODONE Hydrochloride	oxyCODONE	d00329
64720022410	OxyCODONE Hydrochloride	oxyCODONE	d00329
65162004710	OxyCODONE Hydrochloride	oxyCODONE	d00329
65162004750	OxyCODONE Hydrochloride	oxyCODONE	d00329
66479058010	Roxicodone	oxyCODONE	d00329
66479058025	Roxicodone	oxyCODONE	d00329
68084035401	OxyCODONE Hydrochloride	oxyCODONE	d00329
68084035411	OxyCODONE Hydrochloride	oxyCODONE	d00329
68308010801	OxyCODONE Hydrochloride	oxyCODONE	d00329
68308050547	OxyCODONE Hydrochloride	oxyCODONE	d00329
68382079301	OxyCODONE Hydrochloride	oxyCODONE	d00329
69344011311	Oxaydo	oxyCODONE	d00329
00054039041	OxyCODONE Hydrochloride	oxyCODONE	d00329
00054039063	OxyCODONE Hydrochloride	oxyCODONE	d00329
00054052341	OxyCODONE Hydrochloride	oxyCODONE	d00329
00054052363	OxyCODONE Hydrochloride	oxyCODONE	d00329

00121483905	OxyCODONE Hydrochloride	oxyCODONE	d00329
00121483940	OxyCODONE Hydrochloride	oxyCODONE	d00329
60432070605	OxyCODONE Hydrochloride	oxyCODONE	d00329
62559015116	OxyCODONE Hydrochloride	oxyCODONE	d00329
64950035445	OxyCODONE Hydrochloride	oxyCODONE	d00329
64950035450	OxyCODONE Hydrochloride	oxyCODONE	d00329
64950035455	OxyCODONE Hydrochloride	oxyCODONE	d00329
66689002401	OxyCODONE Hydrochloride	oxyCODONE	d00329
66689002416	OxyCODONE Hydrochloride	oxyCODONE	d00329
66689002450	OxyCODONE Hydrochloride	oxyCODONE	d00329
66689040101	OxyCODONE Hydrochloride	oxyCODONE	d00329
66689040150	OxyCODONE Hydrochloride	oxyCODONE	d00329
66689040316	OxyCODONE Hydrochloride	oxyCODONE	d00329
00115156101	OxyCODONE Hydrochloride ER	oxyCODONE	d00329
59011046010	OxyCONTIN	oxyCODONE	d00329
59011046020	OxyCONTIN	oxyCODONE	d00329
60793052601	Oxecta	oxyCODONE	d00329
69344021311	Oxaydo	oxyCODONE	d00329
00093573401	OxyCODONE Hydrochloride ER	oxyCODONE	d00329
00115156201	OxyCODONE Hydrochloride ER	oxyCODONE	d00329
00591270801	OxyCODONE Hydrochloride ER	oxyCODONE	d00329
00781578501	OxyCODONE Hydrochloride ER	oxyCODONE	d00329
35356040090	OxyCONTIN	oxyCODONE	d00329
49884019701	OxyCODONE Hydrochloride ER	oxyCODONE	d00329
59011048010	OxyCONTIN	oxyCODONE	d00329
59011048020	OxyCONTIN	oxyCODONE	d00329
63304068401	OxyCODONE Hydrochloride ER	oxyCODONE	d00329
24510011010	Xtampza ER	oxyCODONE	d00329
00054028425	Oxymorphone Hydrochloride	oxyMORphone	d00833
00093586201	Oxymorphone Hydrochloride	oxyMORphone	d00833
00406101001	Oxymorphone Hydrochloride	oxyMORphone	d00833
10702007101	Oxymorphone Hydrochloride	oxyMORphone	d00833
13107010401	Oxymorphone Hydrochloride	oxyMORphone	d00833
35356038030	Opana	oxyMORphone	d00833
60687014811	Oxymorphone Hydrochloride	oxyMORphone	d00833
60687014821	Oxymorphone Hydrochloride	oxyMORphone	d00833
60951079570	Oxymorphone Hydrochloride	oxyMORphone	d00833
63481061370	Opana	oxyMORphone	d00833
64720025910	Oxymorphone Hydrochloride	oxyMORphone	d00833
00115123201	Oxymorphone Hydrochloride ER	oxyMORphone	d00833
00115123213	Oxymorphone Hydrochloride ER	oxyMORphone	d00833
00228322806	Oxymorphone Hydrochloride ER	oxyMORphone	d00833
00228322811	Oxymorphone Hydrochloride ER	oxyMORphone	d00833
35356038860	Opana ER	oxyMORphone	d00833
63481043670	Opana ER	oxyMORphone	d00833

63481067470	Opana ER	oxyMORphone	d00833
63481081420	Opana ER	oxyMORphone	d00833
63481081460	Opana ER	oxyMORphone	d00833
00115131601	Oxymorphone Hydrochloride ER	oxyMORphone	d00833
00115131613	Oxymorphone Hydrochloride ER	oxyMORphone	d00833
00228326206	Oxymorphone Hydrochloride ER	oxyMORphone	d00833
00228326211	Oxymorphone Hydrochloride ER	oxyMORphone	d00833
63481081520	Opana ER	oxyMORphone	d00833
63481081560	Opana ER	oxyMORphone	d00833
00115123301	Oxymorphone Hydrochloride ER	oxyMORphone	d00833
00115123313	Oxymorphone Hydrochloride ER	oxyMORphone	d00833
00228322906	Oxymorphone Hydrochloride ER	oxyMORphone	d00833
00228322911	Oxymorphone Hydrochloride ER	oxyMORphone	d00833
63481043870	Opana ER	oxyMORphone	d00833
63481061770	Opana ER	oxyMORphone	d00833
63481061775	Opana ER	oxyMORphone	d00833
63481081620	Opana ER	oxyMORphone	d00833
63481081660	Opana ER	oxyMORphone	d00833
00115131701	Oxymorphone Hydrochloride ER	oxyMORphone	d00833
00115131713	Oxymorphone Hydrochloride ER	oxyMORphone	d00833
00228326306	Oxymorphone Hydrochloride ER	oxyMORphone	d00833
00228326311	Oxymorphone Hydrochloride ER	oxyMORphone	d00833
63481043970	Opana ER	oxyMORphone	d00833
63481057170	Opana ER	oxyMORphone	d00833
63481081720	Opana ER	oxyMORphone	d00833
63481081760	Opana ER	oxyMORphone	d00833
00115123401	Oxymorphone Hydrochloride ER	oxyMORphone	d00833
00115123413	Oxymorphone Hydrochloride ER	oxyMORphone	d00833
00228323006	Oxymorphone Hydrochloride ER	oxyMORphone	d00833
00228323011	Oxymorphone Hydrochloride ER	oxyMORphone	d00833
63481044070	Opana ER	oxyMORphone	d00833
63481069370	Opana ER	oxyMORphone	d00833
63481081820	Opana ER	oxyMORphone	d00833
63481081860	Opana ER	oxyMORphone	d00833
00054028325	Oxymorphone Hydrochloride	oxyMORphone	d00833
00093586101	Oxymorphone Hydrochloride	oxyMORphone	d00833
00406100901	Oxymorphone Hydrochloride	oxyMORphone	d00833
10702007001	Oxymorphone Hydrochloride	oxyMORphone	d00833
13107010301	Oxymorphone Hydrochloride	oxyMORphone	d00833
60951079470	Oxymorphone Hydrochloride	oxyMORphone	d00833
63481061270	Opana	oxyMORphone	d00833
64720025810	Oxymorphone Hydrochloride	oxyMORphone	d00833
00115123101	Oxymorphone Hydrochloride ER	oxyMORphone	d00833
00115123113	Oxymorphone Hydrochloride ER	oxyMORphone	d00833
00228322706	Oxymorphone Hydrochloride ER	oxyMORphone	d00833

00228322711	Oxymorphone Hydrochloride ER	oxyMORphone	d00833
63481081220	Opana ER	oxyMORphone	d00833
63481081260	Opana ER	oxyMORphone	d00833
63481090770	Opana ER	oxyMORphone	d00833
63481090775	Opana ER	oxyMORphone	d00833
00115131501	Oxymorphone Hydrochloride ER	oxyMORphone	d00833
00115131513	Oxymorphone Hydrochloride ER	oxyMORphone	d00833
00228326106	Oxymorphone Hydrochloride ER	oxyMORphone	d00833
00228326111	Oxymorphone Hydrochloride ER	oxyMORphone	d00833
63481081320	Opana ER	oxyMORphone	d00833
63481081360	Opana ER	oxyMORphone	d00833
50458084002	Nucynta	tapentadol	d07453
50458084004	Nucynta	tapentadol	d07453
69865023002	Nucynta	tapentadol	d07453
50458086101	Nucynta ER	tapentadol	d07453
69865024502	Nucynta ER	tapentadol	d07453
50458086201	Nucynta ER	tapentadol	d07453
69865025002	Nucynta ER	tapentadol	d07453
50458086301	Nucynta ER	tapentadol	d07453
69865026002	Nucynta ER	tapentadol	d07453
50458086401	Nucynta ER	tapentadol	d07453
69865026501	Nucynta ER	tapentadol	d07453
50458082002	Nucynta	tapentadol	d07453
50458082004	Nucynta	tapentadol	d07453
69865021002	Nucynta	tapentadol	d07453
50458086001	Nucynta ER	tapentadol	d07453
69865024002	Nucynta ER	tapentadol	d07453
50458083002	Nucynta	tapentadol	d07453
50458083004	Nucynta	tapentadol	d07453
69865022002	Nucynta	tapentadol	d07453
13811068930	TraMADol Hydrochloride ER	traMADol	d03826
68025005330	ConZip	traMADol	d03826
68025007130	ConZip	traMADol	d03826
00378415293	TraMADol Hydrochloride ER	traMADol	d03826
10147090103	TraMADol Hydrochloride ER	traMADol	d03826
10370022111	TraMADol Hydrochloride ER	traMADol	d03826
21695029230	Ultram ER	traMADol	d03826
35356005530	Ultram ER	traMADol	d03826
42858090103	TraMADol Hydrochloride ER	traMADol	d03826
47335053183	TraMADol Hydrochloride ER	traMADol	d03826
47335053188	TraMADol Hydrochloride ER	traMADol	d03826
47335085983	TraMADol Hydrochloride ER	traMADol	d03826
49884082111	TraMADol Hydrochloride ER	traMADol	d03826
50458065330	Ultram ER	traMADol	d03826
59011033430	Ryzolt	traMADol	d03826

60429058330	TraMADol Hydrochloride ER	traMADol	d03826
68084096311	TraMADol Hydrochloride ER	traMADol	d03826
68084096321	TraMADol Hydrochloride ER	traMADol	d03826
68180038306	TraMADol Hydrochloride ER	traMADol	d03826
68180069706	TraMADol Hydrochloride ER	traMADol	d03826
69420300201	TraMADol Hydrochloride ER	traMADol	d03826
69467100101	TraMADol Hydrochloride ER	traMADol	d03826
13811069030	TraMADol Hydrochloride ER	traMADol	d03826
54569632800	ConZip	traMADol	d03826
68025005530	ConZip	traMADol	d03826
68025007230	ConZip	traMADol	d03826
00378415393	TraMADol Hydrochloride ER	traMADol	d03826
10147090203	TraMADol Hydrochloride ER	traMADol	d03826
10370022211	TraMADol Hydrochloride ER	traMADol	d03826
21695056330	Ultram ER	traMADol	d03826
42858090203	TraMADol Hydrochloride ER	traMADol	d03826
43063024130	Ryzolt	traMADol	d03826
47335053383	TraMADol Hydrochloride ER	traMADol	d03826
47335053388	TraMADol Hydrochloride ER	traMADol	d03826
47335086083	TraMADol Hydrochloride ER	traMADol	d03826
49884082211	TraMADol Hydrochloride ER	traMADol	d03826
49999089610	Ultram ER	traMADol	d03826
49999089630	Ultram ER	traMADol	d03826
50458065530	Ultram ER	traMADol	d03826
59011033530	Ryzolt	traMADol	d03826
60429058430	TraMADol Hydrochloride ER	traMADol	d03826
60760065530	Ultram ER	traMADol	d03826
68180038406	TraMADol Hydrochloride ER	traMADol	d03826
68180069806	TraMADol Hydrochloride ER	traMADol	d03826
13811069130	TraMADol Hydrochloride ER	traMADol	d03826
68025005630	ConZip	traMADol	d03826
68025007330	ConZip	traMADol	d03826
00378415493	TraMADol Hydrochloride ER	traMADol	d03826
10147090303	TraMADol Hydrochloride ER	traMADol	d03826
10370022311	TraMADol Hydrochloride ER	traMADol	d03826
21695091330	Ultram ER	traMADol	d03826
35356005630	Ultram ER	traMADol	d03826
42858090303	TraMADol Hydrochloride ER	traMADol	d03826
47335053783	TraMADol Hydrochloride ER	traMADol	d03826
47335053788	TraMADol Hydrochloride ER	traMADol	d03826
47335086183	TraMADol Hydrochloride ER	traMADol	d03826
49884082311	TraMADol Hydrochloride ER	traMADol	d03826
50458065730	Ultram ER	traMADol	d03826
59011033630	Ryzolt	traMADol	d03826
60429058530	TraMADol Hydrochloride ER	traMADol	d03826

68180038506	TraMADol Hydrochloride ER	traMADol	d03826
68180069906	TraMADol Hydrochloride ER	traMADol	d03826
00093005801	TraMADol Hydrochloride	traMADol	d03826
00093005805	TraMADol Hydrochloride	traMADol	d03826
00247123004	Ultram	traMADol	d03826
00247123006	Ultram	traMADol	d03826
00247123012	Ultram	traMADol	d03826
00247123015	Ultram	traMADol	d03826
00247123016	Ultram	traMADol	d03826
00247123020	Ultram	traMADol	d03826
00247123021	Ultram	traMADol	d03826
00247123025	Ultram	traMADol	d03826
00247123028	Ultram	traMADol	d03826
00247123030	Ultram	traMADol	d03826
00247123050	Ultram	traMADol	d03826
00247123060	Ultram	traMADol	d03826
00247123077	Ultram	traMADol	d03826
00247198004	TraMADol Hydrochloride	traMADol	d03826
00247198006	TraMADol Hydrochloride	traMADol	d03826
00247198010	TraMADol Hydrochloride	traMADol	d03826
00247198012	TraMADol Hydrochloride	traMADol	d03826
00247198015	TraMADol Hydrochloride	traMADol	d03826
00247198016	TraMADol Hydrochloride	traMADol	d03826
00247198020	TraMADol Hydrochloride	traMADol	d03826
00247198021	TraMADol Hydrochloride	traMADol	d03826
00247198025	TraMADol Hydrochloride	traMADol	d03826
00247198028	TraMADol Hydrochloride	traMADol	d03826
00247198030	TraMADol Hydrochloride	traMADol	d03826
00247198050	TraMADol Hydrochloride	traMADol	d03826
00247198060	TraMADol Hydrochloride	traMADol	d03826
00247198077	TraMADol Hydrochloride	traMADol	d03826
00378415101	TraMADol Hydrochloride	traMADol	d03826
00378415105	TraMADol Hydrochloride	traMADol	d03826
00378415193	TraMADol Hydrochloride	traMADol	d03826
00904611961	TraMADol Hydrochloride	traMADol	d03826
00904636561	TraMADol Hydrochloride	traMADol	d03826
10135051901	TraMADol Hydrochloride	traMADol	d03826
10135051905	TraMADol Hydrochloride	traMADol	d03826
10135051910	TraMADol Hydrochloride	traMADol	d03826
16590022920	TraMADol Hydrochloride	traMADol	d03826
16714011104	TraMADol Hydrochloride	traMADol	d03826
16714011105	TraMADol Hydrochloride	traMADol	d03826
16714011106	TraMADol Hydrochloride	traMADol	d03826
16714011110	TraMADol Hydrochloride	traMADol	d03826
16714011111	TraMADol Hydrochloride	traMADol	d03826

16714011112	TraMADol Hydrochloride	traMADol	d03826
16714048101	TraMADol Hydrochloride	traMADol	d03826
16714048102	TraMADol Hydrochloride	traMADol	d03826
16714048103	TraMADol Hydrochloride	traMADol	d03826
21695013208	TraMADol Hydrochloride	traMADol	d03826
21695013245	TraMADol Hydrochloride	traMADol	d03826
23490683200	TraMADol Hydrochloride	traMADol	d03826
23490683201	TraMADol Hydrochloride	traMADol	d03826
23490683202	TraMADol Hydrochloride	traMADol	d03826
23490683204	TraMADol Hydrochloride	traMADol	d03826
23490683205	TraMADol Hydrochloride	traMADol	d03826
23490683206	TraMADol Hydrochloride	traMADol	d03826
23490683207	TraMADol Hydrochloride	traMADol	d03826
23490683208	TraMADol Hydrochloride	traMADol	d03826
23490683209	TraMADol Hydrochloride	traMADol	d03826
23490784600	TraMADol Hydrochloride	traMADol	d03826
23490784601	TraMADol Hydrochloride	traMADol	d03826
23490784602	TraMADol Hydrochloride	traMADol	d03826
23490784603	TraMADol Hydrochloride	traMADol	d03826
33342020111	TraMADol Hydrochloride	traMADol	d03826
33342020115	TraMADol Hydrochloride	traMADol	d03826
33342020144	TraMADol Hydrochloride	traMADol	d03826
33358034201	TraMADol Hydrochloride	traMADol	d03826
43063005506	TraMADol Hydrochloride	traMADol	d03826
43353007230	TraMADol Hydrochloride	traMADol	d03826
43353007290	TraMADol Hydrochloride	traMADol	d03826
43353028330	TraMADol Hydrochloride	traMADol	d03826
49999002215	Ultram	traMADol	d03826
49999002220	Ultram	traMADol	d03826
49999002221	Ultram	traMADol	d03826
49999002225	Ultram	traMADol	d03826
49999002230	Ultram	traMADol	d03826
49999002256	Ultram	traMADol	d03826
49999012900	TraMADol Hydrochloride	traMADol	d03826
49999012901	TraMADol Hydrochloride	traMADol	d03826
49999012915	TraMADol Hydrochloride	traMADol	d03826
49999012918	TraMADol Hydrochloride	traMADol	d03826
49999012920	TraMADol Hydrochloride	traMADol	d03826
49999012924	TraMADol Hydrochloride	traMADol	d03826
49999012930	TraMADol Hydrochloride	traMADol	d03826
49999012960	TraMADol Hydrochloride	traMADol	d03826
49999012990	TraMADol Hydrochloride	traMADol	d03826
50458065960	Ultram	traMADol	d03826
51079099101	TraMADol Hydrochloride	traMADol	d03826
51079099120	TraMADol Hydrochloride	traMADol	d03826

51079099130	TraMADol Hydrochloride	traMADol	d03826
51079099156	TraMADol Hydrochloride	traMADol	d03826
52959041400	Ultram	traMADol	d03826
52959041402	Ultram	traMADol	d03826
52959041410	Ultram	traMADol	d03826
52959041415	Ultram	traMADol	d03826
52959041420	Ultram	traMADol	d03826
52959041421	Ultram	traMADol	d03826
52959041424	Ultram	traMADol	d03826
52959041428	Ultram	traMADol	d03826
52959041430	Ultram	traMADol	d03826
52959041440	Ultram	traMADol	d03826
52959041460	Ultram	traMADol	d03826
52959068800	TraMADol Hydrochloride	traMADol	d03826
52959068802	TraMADol Hydrochloride	traMADol	d03826
52959068803	TraMADol Hydrochloride	traMADol	d03826
52959068805	TraMADol Hydrochloride	traMADol	d03826
52959068810	TraMADol Hydrochloride	traMADol	d03826
52959068812	TraMADol Hydrochloride	traMADol	d03826
52959068815	TraMADol Hydrochloride	traMADol	d03826
52959068818	TraMADol Hydrochloride	traMADol	d03826
52959068820	TraMADol Hydrochloride	traMADol	d03826
52959068821	TraMADol Hydrochloride	traMADol	d03826
52959068824	TraMADol Hydrochloride	traMADol	d03826
52959068825	TraMADol Hydrochloride	traMADol	d03826
52959068828	TraMADol Hydrochloride	traMADol	d03826
52959068830	TraMADol Hydrochloride	traMADol	d03826
52959068835	TraMADol Hydrochloride	traMADol	d03826
52959068840	TraMADol Hydrochloride	traMADol	d03826
52959068850	TraMADol Hydrochloride	traMADol	d03826
52959068856	TraMADol Hydrochloride	traMADol	d03826
52959068860	TraMADol Hydrochloride	traMADol	d03826
52959068884	TraMADol Hydrochloride	traMADol	d03826
52959068890	TraMADol Hydrochloride	traMADol	d03826
54569408900	Ultram	traMADol	d03826
54569408901	Ultram	traMADol	d03826
54569408905	Ultram	traMADol	d03826
54569543600	TraMADol Hydrochloride	traMADol	d03826
54569543601	TraMADol Hydrochloride	traMADol	d03826
54569543602	TraMADol Hydrochloride	traMADol	d03826
54569543603	TraMADol Hydrochloride	traMADol	d03826
54569543604	TraMADol Hydrochloride	traMADol	d03826
54569543605	TraMADol Hydrochloride	traMADol	d03826
54569543607	TraMADol Hydrochloride	traMADol	d03826
54569543609	TraMADol Hydrochloride	traMADol	d03826

54569596700	TraMADol Hydrochloride	traMADol	d03826
54569596701	TraMADol Hydrochloride	traMADol	d03826
54569596702	TraMADol Hydrochloride	traMADol	d03826
54569596703	TraMADol Hydrochloride	traMADol	d03826
54569596704	TraMADol Hydrochloride	traMADol	d03826
54738021001	TraMADol Hydrochloride	traMADol	d03826
54868360500	Ultram	traMADol	d03826
54868360502	Ultram	traMADol	d03826
54868360503	Ultram	traMADol	d03826
54868360505	Ultram	traMADol	d03826
54868463800	TraMADol Hydrochloride	traMADol	d03826
55045292802	TraMADol Hydrochloride	traMADol	d03826
55045292803	TraMADol Hydrochloride	traMADol	d03826
55289065015	Ultram	traMADol	d03826
55289065020	Ultram	traMADol	d03826
55289065024	Ultram	traMADol	d03826
55289065030	Ultram	traMADol	d03826
55289071910	TraMADol Hydrochloride	traMADol	d03826
55289071912	TraMADol Hydrochloride	traMADol	d03826
55289071915	TraMADol Hydrochloride	traMADol	d03826
55289071920	TraMADol Hydrochloride	traMADol	d03826
55289071930	TraMADol Hydrochloride	traMADol	d03826
55289071940	TraMADol Hydrochloride	traMADol	d03826
55289071950	TraMADol Hydrochloride	traMADol	d03826
55289071960	TraMADol Hydrochloride	traMADol	d03826
55289071990	TraMADol Hydrochloride	traMADol	d03826
55289071994	TraMADol Hydrochloride	traMADol	d03826
55289071998	TraMADol Hydrochloride	traMADol	d03826
55887065810	TraMADol Hydrochloride	traMADol	d03826
55887065815	TraMADol Hydrochloride	traMADol	d03826
55887065850	TraMADol Hydrochloride	traMADol	d03826
55887065892	TraMADol Hydrochloride	traMADol	d03826
57664037708	TraMADol Hydrochloride	traMADol	d03826
57664037713	TraMADol Hydrochloride	traMADol	d03826
57664037718	TraMADol Hydrochloride	traMADol	d03826
58864067820	TraMADol Hydrochloride	traMADol	d03826
60429058801	TraMADol Hydrochloride	traMADol	d03826
60505017101	TraMADol Hydrochloride	traMADol	d03826
60505017102	TraMADol Hydrochloride	traMADol	d03826
60505017108	TraMADol Hydrochloride	traMADol	d03826
60760037709	TraMADol Hydrochloride	traMADol	d03826
60760037720	TraMADol Hydrochloride	traMADol	d03826
60760037730	TraMADol Hydrochloride	traMADol	d03826
60760037760	TraMADol Hydrochloride	traMADol	d03826
60760037798	TraMADol Hydrochloride	traMADol	d03826

62584055901	TraMADol Hydrochloride	traMADol	d03826
62584055911	TraMADol Hydrochloride	traMADol	d03826
63629286800	TraMADol Hydrochloride	traMADol	d03826
63739067110	TraMADol Hydrochloride	traMADol	d03826
63874532302	Ultram	traMADol	d03826
63874532303	Ultram	traMADol	d03826
63874532304	Ultram	traMADol	d03826
63874532305	Ultram	traMADol	d03826
63874532306	Ultram	traMADol	d03826
65162062710	TraMADol Hydrochloride	traMADol	d03826
65162062711	TraMADol Hydrochloride	traMADol	d03826
65162062750	TraMADol Hydrochloride	traMADol	d03826
65243030006	TraMADol Hydrochloride	traMADol	d03826
66267021025	TraMADol Hydrochloride	traMADol	d03826
66336091530	TraMADol Hydrochloride	traMADol	d03826
67544020753	TraMADol Hydrochloride	traMADol	d03826
67544027530	TraMADol Hydrochloride	traMADol	d03826
68084080801	TraMADol Hydrochloride	traMADol	d03826
68382031901	TraMADol Hydrochloride	traMADol	d03826
68382031905	TraMADol Hydrochloride	traMADol	d03826
68382031910	TraMADol Hydrochloride	traMADol	d03826
68387090012	TraMADol Hydrochloride	traMADol	d03826
69543013610	TraMADol Hydrochloride	traMADol	d03826
69543013611	TraMADol Hydrochloride	traMADol	d03826
69543013650	TraMADol Hydrochloride	traMADol	d03826
76439013610	TraMADol Hydrochloride	traMADol	d03826
76439013611	TraMADol Hydrochloride	traMADol	d03826
76439013650	TraMADol Hydrochloride	traMADol	d03826
59630082503	Rybix ODT	traMADol	d03826
68453082503	Rybix ODT	traMADol	d03826

[1] opioids prescribed up to one month prior to first pain severity score encounter and up to six months post, with designation of active or complete, with information used to capture MME and duration available: dose quantity, frequency of use, dose units, route, start/stop date, if multiple opioids prescribed in a day then MME calculated for each and summed, the median MME was taken across multiple opioids across the inclusion period

Supplemental Table 3: List of codes used to define Charlson Comorbidity Index (CCI)

CCI condition	Code type	Codes
Myocardial infarction	ICD-9	410.X, 411.0, 411.89, 412.X, 414.01, 414.8, 414.9, 429.2, 429.4, 429.6, 429.71, 429.79, 997.1
	SNOMED	10273003, 103011000119106, 1077002, 129574000, 15712841000119100, 15712881000119105, 15712921000119103, 15712961000119108, 15713041000119103, 15713081000119108, 15713121000119105, 15713161000119100, 15713201000119105, 15963181000119104, 15990001, 161502000, 161503005, 164865005, 164867002, 164868007, 164869004, 164870003, 164871004, 17531000119105, 1755008, 194802003, 194809007, 194856005, 194857001, 194858006, 194861007, 194862000, 194863005, 194865003, 194866002, 194867006, 194868001, 22298006, 23311000119105, 233825009, 233826005, 233827001, 233828006, 233829003, 233830008, 233831007, 233832000, 233833005, 233834004, 233835003, 233836002, 233837006, 233838001, 233839009, 233840006, 233841005, 233842003, 233843008, 233846000, 233847009, 233885007, 282006, 285981000119103, 285991000119100, 30277009, 304914007, 307140009, 308065005, 311792005, 311793000, 311796008, 314207007, 32574007, 371068009, 371824008, 371864007, 371865008, 371866009, 371867000, 380001000004106, 394710008, 399211009, 401303003, 401314000, 418044006, 42531007, 428196007, 428752002, 429391004, 429731003, 43630006, 461000119108, 52035003, 54329005, 57054005, 58612006, 59063002, 62695002, 64627002, 65547006, 66189004, 698593009, 70211005, 703164000, 703165004, 703209002, 703210007, 703211006, 703212004, 703213009, 703251009, 703252002, 703253007, 703326006, 703328007, 703330009, 703360004, 70422006, 70998009, 710031008, 71023004, 7326005, 73795002, 73999000, 76593002, 79009004
	ICD-10	I21.X, I22.X, I23.0, I23.1, I23.2, I23.3, I23.5, I23.6, I23.8, I24.1, I24.8, I25.10, I25.6, I25.9, I97.111, I97.191, I97.791, I25.2
Congestive heart failure	ICD-9	402.01, 402.11, 402.91, 404.01, 404.03, 404.11, 404.13, 404.91, 404.93,398.91
	SNOMED	15781000119107, 194779001, 194781004, 438367009, 5148006, 77737007, 3105008,194767001
	ICD-10	142.9X, 143.X, 150.X, 425.4X, 425.5X, 425.7X, 425.8X, 425.9X, 426.6X, 428.X, I11.0, I13.0, I13.2, I25.5, I42.0, I42.5X, I42.6X, I42.7X, I42.8X, P29.0, I09.9
Peripheral vascular disease	ICD-9	437.3, 440.X, 441.X, 443.X, 447.1, 447.9, 459.39, 459.89, 557.1, 557.9, V43.4,093.0
	SNOMED	153811000119105, 195313002, 233958001, 449821000124101, 449831000124103,399957001
	ICD-10	I70.X, I71.X, I73.8, I73.9, I77.1, I77.9, I79.0, I79.2, I87.399, I99.8, K55.1, K55.8, K55.9, Z95.9, I73.1
Cerebrovascular disease	ICD-9	377.72, 430.X-438.X, V12.54,362.34
	SNOMED	102831000119104, 103761000119107, 106241000119108, 108691000119102, 111297002, 111298007, 116288000, 118951000119103, 118971000119107, 125081000119106, 133981000119106, 133991000119109, 137991000119103, 140221000119109, 140281000119108, 140701000119108, 140711000119106, 140911000119109, 140921000119102, 141821000119104, 141831000119101, 145741000119101, 148871000119109, 149821000119103, 151161000119102, 15258001, 15967181000119108, 161511000, 18761000119108, 192813004, 195185009, 195186005, 195189003, 195190007, 195199008, 195200006, 195201005, 195206000, 195209007, 195211003, 195212005, 195213000, 195230003, 195231004, 195232006, 195233001, 195234007, 195236009, 195239002, 195243003, 20059004, 204501003, 21290001000004104, 230523009, 230690007, 230692004, 230694003, 230695002, 230696001, 230698000, 230699008, 230700009, 230701008, 230702001, 230703006, 230704000, 230706003, 230708002, 230713003, 230715005, 230716006, 230717002, 230738008, 23671000119107, 25133001, 26021000119107, 266257000, 275434003, 275526006, 276219001, 276220007, 276221006, 276222004, 281240008, 288723005, 293811000119100,

		293831000119105, 29941000119105, 302904002, 302909007, 307363008, 307766002, 307767006, 308067002, 33331000119103, 34181000119102, 34191000119104, 34781003, 361000119103, 371040005, 371041009, 373606000, 390936003, 40161000119102, 413102000, 413758000, 41713005, 422504002, 425642008, 425882004, 426033005, 426788002, 426814001, 426983002, 427065003, 427296003, 427432001, 428668000, 429235008, 429993008, 430781000124102, 430831000124106, 430841000124101, 430947007, 430959006, 432051000124108, 432181000124104, 432191000124101, 432504007, 433183000, 433891000124100, 433911000124103, 433931000124109, 433941000124104, 433951000124102, 433961000124100, 433971000124107, 434141000124103, 434151000124101, 434951000124104, 434961000124102, 434991000124105, 436041000124107, 440140008, 441526008, 441630004, 441735003, 441887006, 441894009, 441960006, 441991000, 442024001, 442097001, 442181008, 442212003, 442668000, 442676003, 442733008, 443929000, 444172003, 444657001, 46421000119102, 48601000119107, 5571000124103, 57981008, 62914000, 64009001, 672521000119108, 672561000119103, 674161000119102, 674361000119104, 674401000119108, 690051000119100, 690071000119109, 69533002, 699429007, 703163006, 703205008, 703207000, 703208005, 705128004, 705130002, 710575003, 78569004, 87551000119101, 87555007, 90921000119104, 91601000119109, 92341000119107, 95457000, 95460007, 97531000119106, 9901000119100, 99051000119101, 99451000119105, 441759008
	ICD-10	G45.X, G46.X, H47.649, I60.X, I61.X, I62.X, I63.X, I64.X, I65.X, I66.X, I67.X, I68.X, I69.X, Z86.73, H34.0
Dementia	ICD-9	290.X, 331.19, 331.2, 294.1
	SNOMED	10349009, 106021000119105, 14070001, 230285003, 230287006, 25772007, 420614009, 421023003, 421529006, 56267009, 703544004, 70936005, 713488003, 713844000, 281004
	ICD-10	F00.X, F01.X, F02.X, F03.X, G30.X, G31.09, G31.1, G71.2, F05.1
Chronic obstructive pulmonary disease	ICD-9	415.2, 416.9, 490.X-505.X, 506.4, 508.1, 508.8, 508.9, 514, 515,416.8
	SNOMED	106001000119101, 10692761000119107, 11211003, 11641008, 12428000, 125294002, 125295001, 13151001, 135836000, 13645005, 16003001, 16846004, 1751000119100, 185086009, 195949008, 195951007, 195957006, 195958001, 195959009, 195963002, 195967001, 195977004, 196001008, 196026004, 233672007, 233675009, 233677001, 233678006, 233679003, 233681001, 233683003, 233685005, 233686006, 233688007, 233935004, 23958009, 266355005, 266356006, 266361008, 266364000, 276637009, 281239006, 285381006, 293241000119100, 30352005, 304527002, 313296004, 313297008, 313299006, 31387002, 31886003, 31898008, 34015007, 370218001, 370219009, 370220003, 370221004, 389145006, 390921001, 40100001, 401000119107, 401193004, 404808000, 405944004, 407674008, 409663006, 423889005, 424199006, 424643009, 425969006, 426656000, 426979002, 427295004, 427354000, 427603009, 427679007, 442025000, 4981000, 5281000124103, 55570000, 56968009, 57546000, 57607007, 57686001, 59327009, 59786004, 63088003, 66110007, 68328006, 707412000, 707413005, 707445000, 707446004, 707447008, 70756004, 708030004, 85761009, 86680006, 87433001, 901000119100, 91340006, 92807009, 93432008,
	ICD-10	I27.9, J27.82, J40.X-J47.X, J60.X-J67.X, J68.4, J70.1, J70.3, J70.4, J70.9, J81.1, J84.10, P25.0, Q32.4,I27.8
Rheumatic disease	ICD-9	710.0X-710.4X, 714.0X-714.2X, 714.8, 725.X, 446.5
	SNOMED	1212005, 193248005, 193250002, 193252005, 196136009, 202768008, 234529004, 238935002, 238936001, 239898008, 239899000, 239901009, 240120003, 281357005, 281358000, 31384009, 396230008, 402358003, 402425006, 715401008, 86365006, 86365006, 95415006
	ICD-10	M05.X, M06.X, M32.X, M33.X, M34.X, M35.1, M35.3, M36.0, M31.5
Peptic ulcer disease	ICD-9	531.X-534.X

	SNOMED	111353003, 12274003, 12355008, 12847006, 15115006, 15902003, 16694003, 17067009, 17593008, 18367003, 2066005, 22157005, 23812009, 24001002, 26221006, 27281001, 28945005, 307233002, 308882008, 34021006, 35560008, 36975000, 41986000, 42698006, 43406003, 45640006, 46523000, 46708007, 47064007, 48658001, 48974009, 49232000, 50663005, 51847008, 53337006, 53877005, 54798007, 55617001, 55746001, 56461008, 57246001, 58711008, 59356009, 59515005, 61300005, 62341002, 62366003, 62838000, 63954007, 64094003, 64121000, 64398008, 66673003, 66767006, 70418001, 72408002, 74341002, 76078009, 76181002, 77661009, 81142005, 81387001, 81518000, 84124004, 85787009, 85859006, 86258000, 86895006, 87756006, 87796008, 89469000, 89748001, 90257004, 90489006
	ICD-10	,K25.X-K28.X
Mild liver disease	ICD-9	070.20, 070.23, 070.32, 070.33, 070.44, 070.54, 070.6, 070.9, 570.X, 571.X, 572.2, 573.3, 573.4, 573.8, 573.9, 582.8, V42.7,070.22
	SNOMED	103611000119102, 10807061000119103, 1082611000119101, 1085021000119106, 1092801000119102, 109819003, 111371005, 1116000, 123604002, 123605001, 123606000, 12368000, 123716002, 123717006, 15230009, 153091000119109, 15999000, 1761006, 17890003, 1861000, 186639003, 192811002, 197279005, 197284004, 197286002, 197291001, 197293003, 197294009, 197296006, 197299004, 197301006, 197303009, 197305002, 197310003, 197315008, 197321007, 197359004, 197362001, 197364000, 199117000, 199118005, 19943007, 2043009, 230800004, 235856003, 235859005, 235869004, 235871004, 235875008, 235878005, 235880004, 235881000, 235895002, 235896001, 235897005, 235899008, 235901004, 235902006, 243978007, 266468003, 266469006, 266470007, 266471006, 271440004, 27156006, 276552008, 28698006, 307757001, 31712002, 328383001, 33144001, 347891000119103, 36631002, 370889009, 371139006, 37688005, 38662009, 41309000, 413438002, 41889008, 419728003, 420054005, 424340000, 425413006, 427022004, 43904005, 447058001, 45256007, 50167007, 50325005, 536002, 57339008, 58008004, 59229005, 6183001, 61977001, 62484002, 703866000, 713181003, 713370005, 713529007, 713966008, 716203000, 722866000, 723829000, 725416005, 725938001, 725939009, 725940006, 735733008, 737202006, 76301009, 76783007, 78208005, 79720007, 831000119103, 831000119103, 86454000, 89580002, 95557003, 9953008
	ICD-10	B18.X, B19.11, K70.0X-K70.3X, K71.3X-K71.5X, K71.7, K72.91, K73.X, K74.X, K76.0, K76.2X-K76.4X, K76.8, K76.9, Z94.4, K70.9
Diabetes without chronic complication	ICD-9	250.0X-250.3X, 250.9, 250.8
	SNOMED	111231000119109, 111552007, 123763000, 137931000119102, 140401000119104, 140411000119101, 140521000119107, 1481000119100, 190330002, 190331003, 190368000, 190372001, 190388001, 190389009, 199229001, 199230006, 201250006, 23045005, 237599002, 237601000, 237604008, 237611007, 237613005, 237618001, 237619009, 237651005, 24203005, 28032008, 31321000119102, 313435000, 313436004, 314771006, 314893005, 314903002, 314904008, 359642000, 408539000, 420270002, 420868002, 421075007, 422228004, 426875007, 42954008, 44054006, 441628001, 443694000, 46635009, 530558861000132104, 59079001, 609561005, 609562003, 609565001, 609568004, 609569007, 609570008, 609571007, 609572000, 609573005, 609574004, 609575003, 609576002, 609577006, 609578001, 701000119103, 703136005, 703138006, 70694009, 709147009, 716362006, 719216001, 720519003, 722454003, 724136006, 724876003, 73211009, 75682002, 761000119102, 791000119109, 81531005, 8801005, 91352004, 9859006
	ICD-10	E10.1, E10.6, E10.8, E10.9, E11.0, E11.1, E11.6, E11.8, E11.9, E12.0, E12.1, E12.6, E12.8, E12.9, E13.0, E13.1, E13.6, E13.8, E13.9, E14.0, E14.1, E14.6, E14.8, E14.9, E10.0
Diabetes with chronic complication	ICD-9	250.4X-250.7X

	SNOMED	102781000119107, 103981000119101, 104941000119109, 104951000119106, 104961000119108, 109171000119104, 110996009, 126531000119109, 126591000119108, 127013003, 127014009, 127991000119101, 128001000119105, 138881000119106, 138891000119109, 138901000119108, 138911000119106, 138921000119104, 138941000119105, 140101000119109, 140111000119107, 140121000119100, 1491000119102, 1501000119109, 1551000119108, 18521000119106, 193349004, 193350004, 197605007, 232020009, 232021008, 232022001, 232023006, 236499007, 236500003, 243421000119104, 25412000, 28331000119107, 309426007, 310387003, 311366001, 311782002, 31211000119101, 312903003, 312904009, 312905005, 312906006, 312907002, 312908007, 312909004, 312910009, 312912001, 314010006, 314011005, 314014002, 314015001, 314902007, 368521000119107, 368711000119106, 368721000119104, 368741000119105, 38046004, 39058009, 390834004, 399862001, 399863006, 399864000, 399865004, 399866003, 399868002, 399869005, 399870006, 399871005, 399872003, 399873008, 399874002, 399875001, 399876000, 399877009, 401110002, 408409007, 408410002, 408411003, 408412005, 408413000, 408414006, 414894003, 414910007, 417677008, 420279001, 420486006, 420514000, 420715001, 420756003, 420789003, 420918009, 421305000, 421365002, 421779007, 421893009, 421895002, 421920002, 421986006, 422034002, 422166005, 425455002, 426907004, 427027005, 427571000, 43959009, 445170001, 4855003, 59276001, 60961000119107, 60971000119101, 60991000119100, 63510008, 711000119100, 712882000, 71701000119105, 71721000119101, 71791000119104, 721000119107, 731000119105, 82541000119100, 82571000119107, 82581000119105, 90721000119101, 90731000119103, 90751000119109, 90761000119106, 90771000119100, 90791000119104, 96441000119101, 97331000119101, 97341000119105,90741000119107
	ICD-10	E10.2X-E10.5X, E11.2X-E11.5X, E11.7, E12.2X-E12.5X, E12.7, E13.2X-E13.5X, E13.7, E14.2X-E14.5X, E14.7, J44.X-J47.X, J60.X-J67.X, E10.7
Hemiplegia or paraplegia	ICD-9	342.X, 343.X, 344.0X-344.6X, 344.9, 438.20,334.1
	SNOMED	103761000119107, 140281000119108, 140701000119108, 140711000119106, 230702001, 24654003, 361000119103, 425491009, 432181000124104, 432191000124101, 441991000, 442024001, 442668000, 442676003, 442733008, 48601000119107, 54364001
	ICD-10	G11.4, G80.1, G80.2, G81.X, G82.X, G83.0X-G83.4X, G83.9, I69.259, I69.359, G04.1
Renal disease	ICD-9	403.11, 403.91, 404.02, 404.03, 404.12, 404.13, 404.92, 404.93, 582.X, 583.0X-583.7X, 585.X, 586.X, 588.0, V42.0, V45.1, V56.X,403.01
	SNOMED	104931000119100, 111411000119103, 117681000119102, 120261000119101, 127991000119101, 128001000119105, 129151000119102, 129161000119100, 129171000119106, 129181000119109, 140101000119109, 140111000119107, 140121000119100, 14973001, 153851000119106, 153891000119101, 16218721000119107, 194780003, 236434000, 236435004, 236436003, 28119000, 284991000119104, 285011000119108, 285001000119105, 285081000119102, 285101000119109, 285841000119104, 285851000119102, 285861000119100, 285871000119106, 285881000119109, 285911000119109, 285921000119102, 286371000119107, 38481006, 428937001, 428982002, 429075005, 431857002, 433144002, 433146000, 434431000124103, 443143006, 443596009, 46177005, 473392002, 49220004, 57684003, 66052004, 66610008, 691401000119104, 691411000119101, 691421000119108, 698591006, 698810000, 700378005, 700379002, 704667004, 707324008, 711000119100, 712487000, 713696000, 714152005, 714153000, 71701000119105, 721000119107, 731000119105, 78544004, 8501000119104, 86234004, 90721000119101, 90731000119103, 90741000119107, 90751000119109, 90761000119106, 90771000119100, 90791000119104, 96441000119101, 96701000119107, 96711000119105, 96721000119103, 96731000119100, 96741000119109, 96751000119106
	ICD-10	I13.1, N03.4X-N03.7X, N05.2X-N05.7X, N18.X, N19.X, N25.0, Z49.0X-Z49.2X, Z94.0, Z99.2, I12.0
Any malignancy, including lymphoma and	ICD-9	140.X-159.X, 161.X-172.X, 174.X-195.X, 200.X-208.X,238.6

leukemia, except malignant neoplasm of skin		
	SNOMED	109838007, 109839004, 109840002, 109841003, 109842005, 109843000, 109844006, 109962001, 109964000, 109965004, 109966003, 109967007, 109968002, 109969005, 109970006, 109971005, 109972003, 109975001, 109976000, 109977009, 109978004, 109979007, 109980005, 109988003, 109991003, 110000005, 110002002, 110004001, 110005000, 110006004, 110007008, 116711000119103, 116741000119104, 116811000119106, 116821000119104, 117061000119101, 117091000119108, 117111000119100, 118599009, 118600007, 118601006, 118602004, 118605002, 118606001, 118607005, 118608000, 118609008, 118610003, 118611004, 118612006, 118613001, 118614007, 118615008, 118617000, 118618005, 122881000119107, 122901000119109, 122951000119108, 122961000119105, 122981000119101, 12301000132103, 12311000132101, 123781000119107, 127070008, 127220001, 127225006, 127961000119108, 13048006, 133751000119102, 1701000119104, 184881000119106, 187757001, 187767006, 187769009, 187773007, 187776004, 187777008, 187786003, 187822008, 188269007, 188487008, 188489006, 188492005, 188493000, 188498009, 188500005, 188501009, 188502002, 188503007, 188504001, 188505000, 188506004, 188507008, 188510001, 188511002, 188512009, 188513004, 188514005, 188515006, 188516007, 188517003, 188524002, 188526000, 188529007, 188531003, 188534006, 188536008, 188537004, 188538009, 188541000, 188544008, 188547001, 188548006, 188551004, 188554007, 188558005, 188559002, 188562004, 188565002, 188566001, 188567005, 188568000, 188569008, 188570009, 188572001, 188575004, 188576003, 188577007, 188578002, 188579005, 188580008, 188582000, 188585003, 188586002, 188587006, 188589009, 188590000, 188591001, 188592008, 188593003, 188609000, 188612002, 188613007, 188627002, 188630009, 188631008, 188632001, 188633006, 188634000, 188635004, 188637007, 188640007, 188641006, 188642004, 188645002, 188648000, 188649008, 188662007, 188660004, 188663002, 188664008, 188665009, 188666005, 188667001, 188668006, 188669003, 188672005, 188674006, 188675007, 188676008, 188679001, 188718006, 188725004, 188726003, 188728002, 188729005, 188732008, 188736006, 188737002, 188738007, 188741003, 188744006, 188745007, 188746008, 188748009, 188754005, 188768003, 188770007, 190030009, 203436008, 232075002, 236512004, 236513009, 240531002, 253018005, 254582000, 254586002, 254601002, 254792006, 254994000, 255081007, 255101006, 255102004, 269475001, 269476000, 269533000, 269544008, 274902006, 274905008, 276811008, 276815004, 276822007, 276836002, 277473004, 277474005, 277545003, 277549009, 277550009, 277551008, 277567002, 277568007, 277569004, 277570003, 277571004, 277572006, 277573001, 277574007, 277575008, 277587001, 277589003, 277601005, 277602003, 277604002, 277609007, 277610002, 277611003, 277612005, 277613000, 277614006, 277615007, 277616008, 277617004, 277618009, 277619001, 277622004, 277623009, 277624003, 277625002, 277626001, 277627005, 277628000, 277629008, 277632006, 277637000, 277641001, 277642008, 277643003, 277651000, 277653002, 277664004, 278024000, 278051002, 278052009, 278189009, 278453007, 285312008, 285769009, 285776004, 285839005, 301756000, 302841002, 302842009, 302845006, 302848008, 302855005, 302856006, 303017006, 30305500, 303056000, 303057009, 307341004, 307592006, 307617006, 307622006, 307623001, 307624007, 307625008, 307633009, 307634003, 307635002, 307636001, 307637005, 307646004, 307647008, 307649006, 307650006, 308121000, 312111009, 312112002, 312113007, 312114001, 312115000, 314963000, 314965007, 314966008, 314997007, 314998002, 315058005, 359631009, 359640008, 359648001, 363350007, 363351006, 363406005, 363407001, 363408006, 363409003, 363410008, 363412000, 363413005, 363414004, 363491008, 363495004, 363510005, 369448007, 369449004, 369450004, 369451000, 369452007, 369453002, 369454008, 369455009, 369456005, 369457001, 369458006, 369459003, 369460008, 369461007, 371012000, 371134001, 371977004, 373168002, 3854002, 397008008, 397009000, 397011009, 400122007, 402881008, 402882001, 404106004, 404107008, 404108003, 404109006, 404110001, 404111002, 404112009, 404113004, 404114005, 404115006,

404116007, 404117003, 404118008, 404119000, 404120006, 404121005, 404122003, 404123008,
404124002, 404128004, 404129007, 404130002, 404131003, 404132005, 404134006, 404136008,
404137004, 404138009, 404139001, 404140004, 404141000, 404142007, 404143002, 404144008,
404145009, 404147001, 404148006, 404149003, 404150003, 404151004, 404152006, 404153001,
404154007, 404155008, 404157000, 404169008, 404172001, 408645001, 408646000, 413389003,
413441006, 413442004, 413446001, 413656006, 413842007, 413843002, 413847001, 414780005,
414785000, 415112005, 415287001, 420302007, 420519005, 420524008, 420788006, 421246008,
421283008, 422052002, 422853008, 425178004, 425688002, 425749006, 425869007, 425941003,
426071002, 426124006, 426217000, 426248008, 426336007, 426370008, 426642002, 426885008,
427056005, 427141003, 427642009, 427658007, 430338009, 433511000124108, 433541000124107,
433551000124109, 433561000124106, 433571000124104, 433581000124101, 436321000124102,
436331000124104, 436341000124109, 436351000124106, 441559006, 441962003, 442537007,
443487006, 444597005, 444910004, 444911000, 445227008, 445269007, 445448008, 447100004,
447109003, 447656001, 447658000, 447766003, 447805007, 447806008, 447989004, 448212009,
448372003, 448213004, 448217003, 448220006, 448231003, 448254007, 448269008, 448317000,
448319002, 448354009, 448371005, 448376000, 448384001, 448386004, 448387008, 448447004,
448465000, 448468003, 448553002, 448555009, 448560008, 448561007, 448607004, 448609001,
448663003, 448666006, 448672006, 448709005, 448738008, 448774004, 448865007, 448867004,
448994001, 448995000, 449058008, 449059000, 449063007, 449065000, 449074003, 449075002,
449108003, 449173006, 449176003, 449177007, 449216004, 449217008, 449218003, 449219006,
449220000, 449221001, 449222008, 449292003, 449307001, 449318001, 449386007, 449418000,
449419008, 450521000124109, 61291000119103, 61301000119102, 61311000119104, 61321000119106,
681601000119101, 698646006, 699818003, 702446006, 702786004, 702977001, 703387000, 709471005,
713325002, 713483007, 713516007, 713718006, 713897006, 714251006, 714463003, 715664005,
715950008, 716648006, 716654007, 716788007, 718200007, 721305008, 721306009, 721308005,
721310007, 721313009, 721314003, 721555001, 721695008, 721696009, 721699002, 721716004,
721762007, 722795004, 722953004, 722954005, 723889003, 724644005, 724645006, 724647003,
724648008, 724649000, 724650000, 725390002, 725437002, 726721002, 733598001, 733627006,
733834006, 734066005, 737058005, 739301006, 84811000119107, 84831000119102, 91854005,
91855006, 91856007, 91857003, 91858008, 91860005, 91861009, 92508006, 92509003, 92510008,
92511007, 92512000, 92513005, 92514004, 92515003, 92516002, 92811003, 92812005, 92813000,
92814006, 92817004, 92818009, 93133006, 93134000, 93135004, 93136003, 93137007, 93138002,
93139005, 93140007, 93141006, 93142004, 93143009, 93144003, 93145002, 93146001, 93147005,
93148000, 93149008, 93150008, 93151007, 93152000, 93169003, 93182006, 93183001, 93184007,
93185008, 93186009, 93187000, 93188005, 93189002, 93190006, 93191005, 93192003, 93193008,
93194002, 93195001, 93196000, 93197009, 93198004, 93199007, 93200005, 93201009, 93202002,
93203007, 93204001, 93205000, 93206004, 93207008, 93208003, 93450001, 93451002, 93487009,
93488004, 93489007, 93492006, 93493001, 93494007, 93495008, 93496009, 93497000, 93498005,
93500006, 93501005, 93505001, 93506000, 93507009, 93509007, 93510002, 93514006, 93515007,
93516008, 93518009, 93519001, 93520007, 93521006, 93522004, 93523009, 93524003, 93525002,
93526001, 93527005, 93528000, 93530003, 93531004, 93532006, 93533001, 93534007, 93536009,
93537000, 93541001, 93542008, 93543003, 93545005, 93546006, 93547002, 93548007, 93549004,
93550004, 93551000, 93552007, 93554008, 93555009, 93683002, 93761005, 93771007, 93772000,
93826009, 93870000, 93899005, 93900000, 93901001, 93902008, 93903003, 93904009, 93905005,
93906006, 93907002, 93908007, 93909004, 93910009, 93911008, 93912001, 93913006, 93980002,
93984006, 94006002, 94071006, 94072004, 94105000, 94148006, 94686001, 94687005, 94688000,
94690004, 94707004, 94708009, 94709001, 94710006, 94711005, 94712003, 94714002, 94715001,
94716000, 94718004, 94719007, 95186006, 95187002, 95188007, 95192000, 95193005, 95194004,
95209008, 95210003, 95214007, 95224004, 95225003, 95226002, 95230004, 95231000, 95260009,
95261008, 95263006, 95264000, 96281000119107, 96981000119102, 98981000119103,449053004

	ICD-10	C30.X-C34.X, C37.X-C41.X, C43.X, C45.X-C58.X, C60.X-C76.X, C81.X-C85.X, C88.X, C90.X-X97.X, C00.X-C26.X
Moderate or severe liver disease	ICD-9	456.0X-456.2X, 572.2X-572.8X,570
	SNOMED	1082621000119108, 11350001, 13923006, 153091000119109, 197268000, 197269008, 197270009, 197356006, 213230009, 235881000, 235884008, 235886005, 24807004, 26485002, 302126000, 37292007, 405542005, 413438002, 435091000124105, 435101000124104, 51292008, 53104002, 55294001, 59927004, 61250001, 62216007, 65617004, 707167006, 708248004, 720461006, 722864002, 724361001, 81675001, 87248009, 89835009,17709002
	ICD-10	I85.9, I86.4, I98.2, K70.4, K71.1, K72.00, K72.1, K72.9, K76.5, K76.6, K76.7, I85.0
Metastatic solid tumor	ICD-9	196.X-199.X
	SNOMED	116821000119104, 1691000119104, 236512004, 239898008, 277664004, 285610008, 285611007, 285612000, 285613005, 314997007, 314998002, 369448007, 369449004, 369450004, 369451000, 369452007, 369453002, 369454008, 369455009, 369456005, 369457001, 369458006, 369459003, 369460008, 369461007, 404122003, 422282000, 433511000124108, 433521000124100, 433531000124102, 433541000124107, 433551000124109, 433561000124106, 433571000124104, 436321000124102, 436331000124104, 436341000124109, 448922007, 608874000, 713572001, 94179005, 94235004, 94260004, 94271003, 94272005, 94328005, 94349006, 94365007, 94381002, 94418000, 94419008, 94420002, 94421003, 94422005, 94423000, 94424006, 94425007, 94426008, 94427004, 94428009, 94429001, 94430006, 94431005, 94432003, 94509004, 94513006, 94538001, 94604000, 94643001, 96981000119102,116811000119106
	ICD-10	C77.X-C80.X
AIDS/HIV	ICD-9	042.X-044.X
	SNOMED	10746341000119109, 111880001, 186706006, 186707002, 186708007, 186709004, 186717007, 186718002, 186719005, 186721000, 186723002, 186725009, 186726005, 230180003, 230201009, 230598008, 235009000, 235726002, 240103002, 276666007, 315019000, 359791000, 397763006, 398329009, 402915006, 402916007, 405631006, 40780007, 421508002, 421510000, 421529006, 421571007, 421597001, 421660003, 421666009, 421671002, 421695000, 421706001, 421708000, 421710003, 421766003, 421827003, 421851008, 421874007, 421883002, 421929001, 421983003, 421998001, 422003001, 422012004, 422074008, 422089004, 422127002, 422136003, 422177004, 422189002, 422194002, 422282000, 422337001, 442134007, 442537007, 445945000, 48794007, 52079000, 5810003, 62246005, 62479008, 697904001, 697965002, 700053002, 713260006, 713275003, 713278001, 713297001, 713298006, 713299003, 713300006, 713316008, 713318009, 713320007, 713325002, 713339002, 713340000, 713341001, 713342008, 713349004, 713444005, 713445006, 713446007, 713483007, 713484001, 713487008, 713488003, 713489006, 713490002, 713491003, 713497004, 713503007, 713504001, 713505000, 713506004, 713507008, 713508003, 713510001, 713511002, 713523008, 713526000, 713527009, 713530002, 713531003, 713532005, 713533000, 713543002, 713544008, 713545009, 713546005, 713570009, 713571008, 713572001, 713695001, 713696000, 713718006, 713722001, 713729005, 713730000, 713731001, 713732008, 713733003, 713734009, 713844000, 713845004, 713880000, 713881001, 713887002, 713897006, 713964006, 713967004, 714083007, 714464009, 719522009, 721166000, 722557007, 72621000119104, 733834006, 733835007, 735521001, 735522008, 735523003, 735524009, 735525005, 735526006, 735527002, 735528007, 76981000119106, 76991000119109, 77070006, 80191000119101, 81000119104, 86406008, 87117006,442134007
	ICD-10	B24.X, B20.X-B22.X

Supplemental Table 4: List of codes used to define chronic pain

Code Type	Codes
ICD 9	053.12, 053.13, 53.12, 53.13, 260.6X, 274.X, 307.8X, 337.0, 337.1, 337.2X, 338.0, 338.2, 338.2X, 338.4, 339.X, 346.X, 349, 350.X, 352.1, 353.X, 354.X, 355.X, 356.X, 357.X, 377.X, 710-739.X, 780.96, 784.0,
ICD 10	B02.22, B02.23, B02.29, D48.1X, E08.42, E09.42, E10.42, E11.42, E13.42, F45.4X, G43.X, G44.X, G50.X, G51.X, G52.X, G53.X, G54.0X, G54.1X, G54.2X, G54.3X, G54.4X, G54.5X, G54.6X, G54.8X, G54.9X, G56.0X, G56.4X, G57.X, G58.7, G58.9, G60.X, G62.X, G63.X, G64.X, G65.X, G89.0, G89.2, G89.2X, G89.4, G90.0X, G90.5X, G97.1, G99.0, H46.X, H47.X, M00.X, M01.X, M02.X, M05.X, M06.X, M07.X, M08.X, M1A.X, M10.X, M11.X, M12.X, M13.X, M14.X, M15.X, M16.X, M17.X, M18.X, M19.X, M20.X, M21.X, M22.X, M23.X, M24.X, M25.X, M30-M99.X, R26.2X, R26.4X, R29.898, R51.X, R52, S12.X, S13.X, S14.X, S22.X, S23.X, S24.X, S32.X, S33.X, S34.X, S39.012X, S39.023X, S39.092X, S83.0X, S83.1X, S83.2X, S83.3X, S83.4X, S83.5X, S83.6X, S83.8X, S83.9X, M43.06
SNOMED	100491000119103, 10181000119102, 102481003, 10713006, 1073681000119109, 1073691000119107, 1073701000119107, 1073711000119105, 1073721000119103, 1073731000119100, 1073741000119109, 1073751000119106, 1073761000119108, 1073771000119102, 1073781000119104, 1073791000119101, 1073801000119100, 1073811000119102, 1073821000119109, 1073831000119107, 1073861000119104, 1073871000119105, 1073881000119108, 1073891000119106, 108523005, 109771000119j103, 11055151000119108, 111218008, 111220006, 111221005, 111237004, 111985007, 1121000119107, 115491000119105, 117216004, 11892641000119101, 121881000119102, 122061000119109, 122141000119109, 122161003, 123798002, 12396006, 124001000119104, 12400141000119103, 12400181000119108, 12400221000119100, 12400261000119105, 12400301000119102, 12400341000119100, 124041000119102, 124071000119109, 124161000119104, 124171000119105, 128079007, 129501000119107, 129511000119105, 1304004, 133731000119108, 134407002, 136791000119103, 137741002, 137808009, 138752005, 138835002, 13888000, 139311007, 141481000119102, 14150005, 143245009, 143441000119108, 143557001, 145611000119107, 148071003, 148454008, 154931007, 155046006, 155047002, 155048007, 155072002, 155090001, 15633361000119103, 156471009, 156472002, 156473007, 156474001, 156475000, 156476004, 156477008, 156478003, 156479006, 156480009, 156481008, 156483006, 156625009, 156626005, 156649007, 156727006, 15685921000119102, 15685961000119107, 15686001000119104, 15686281000119101, 15686321000119106, 15687201000119107, 15687321000119109, 15687841000119108, 15691161000119108, 15691721000119102, 15691761000119107, 15691801000119104, 15743521000119108, 15743561000119103, 15744441000119101, 15744481000119106, 15744961000119106, 15749801000119104, 15968741000119100, 16002671000119106, 16002871000119105, 16002911000119108, 160342001, 160413008, 16041671000119109, 161481007, 161567008, 162040008, 16206661000119108, 16208561000119106, 16208721000119109, 16580691000119107, 165841003, 16839401000119104, 170845006, 171239004, 1715006, 17205007, 18347007, 192028000, 192499007, 193027003, 193029000, 193030005, 193031009, 193032002, 193033007, 193034001, 193035000, 193036004, 193037008, 193039006, 193040008, 193041007, 193105005, 193119005, 193125009, 193157005, 193180002, 193184006, 193250002, 194493009, 194494003, 194502007, 194503002, 194508006, 1961000, 197834003, 198407008, 201763001, 201764007, 201765008, 201766009, 201767000, 201768005, 201769002, 201770001, 201771002, 201772009, 201773004, 201774005, 201775006, 201776007, 201777003, 201778008, 201779000, 201780002, 201781003, 201782005, 201783000,

201784006, 201785007, 201787004, 201790005, 201791009, 201796004, 201798003, 201799006, 201800005, 201808003, 201810001, 201811002, 201813004, 201819000, 201826000, 201829007, 201831003, 201832005, 201833000, 201834006, 201836008, 201837004, 201847001, 201849003, 201850003, 201851004, 201852006, 201854007, 201855008, 202674000, 202675004, 202676003, 202677007, 202678002, 202679005, 202680008, 202693003, 202694009, 202695005, 202673006, 202696006, 202737009, 20279300, 202794004, 203082005, 203138004, 203490000, 203492008, 203493003, 203729008, 203730003, 203732006, 203746006, 206796000, 207219003, 207621000, 2103002, 21430002, 22193007, 227588009, 22902007, 230462002, 230463007, 230464001, 230465000, 230468003, 230469006, 230470007, 230471006, 230481005, 23056005, 230575000, 23186000, 232284007, 235841007, 237067000, 23894005, 239791005, 239792003, 239793008, 239794002, 239795001, 239801005, 239862000, 24271000087103, 24281000087101, 24291000087104, 24301000087100, 24311000087103, 24331000087108, 24351000087104, 24361000087101, 247365004, 25343008, 254779008, 26002007, 26150009, 267699004, 267887009, 267888004, 267972003, 267984001, 268052008, 2700007, 273206005, 274665008, 274669002, 275902004, 278860009, 279032003, 279039007, 28, 287006005, 287007001, 287008006, 287009003, 287010008, 28736004, 287984007, 290531000119102, 290541000119106, 3, 3061000119102, 307177001, 308143008, 311804006, 313501000119105, 314097005, 316692001, 318871000119107, 318881000119105, 318941000119109, 318951000119106, 319031000119108, 319081000119109, 319111000119104, 319841000119107, 320281000119104, 320291000119101, 322837006, 322838001, 33262002, 33952002, 35074008, 363558001, 373621006, 37796009, 37895003, 389320000, 390055001, 392680008, 393605009, 393638000, 394707001, 396275006, 398057008, 40089004, 402431009, 40709005, 408276001, 408381007, 408662006, 408954001, 408955000, 408956004, 408957008, 40913006, 410795001, 410796000, 410797009, 416666007, 417291007, 41907006, 419568001, 420454001, 421107001, 4222000, 423279000, 423683008, 424699007, 425007008, 425365009, 425936006, 426135001, 426566004, 426628005, 427419006, 427770001, 429181000124108, 429192004, 430696004, 431061000124100, 431481001, 431601000124105, 431707004, 432615008, 433228003, 434011000124101, 436771000124102, 437931000124100, 441711008, 442194005, 442277000, 442521001, 443349002, 443539004, 443728000, 444009006, 444060007, 444746004, 445322004, 445479007, 445985006, 446043004, 4473006, 448701000124105, 45064008, 458071000124104, 458081000124101, 459911000124100, 459921000124108, 46960006, 48210000, 49605003, 50642008, 51049005, 51777006, 51881000119109, 54314008, 56097005, 57160007, 58156007, 59185006, 59292006, 608837004, 631000119102, 64309007, 674051000119103, 69896004, 699314009, 701602006, 702840008, 703182002, 711545001, 712537009, 7126001, 713911007, 714252004, 722981005, 722982003, 724429004, 725058003, 726678001, 734947007, 734989004, 7355002, 735599007, 735600005, 735644008, 735935009, 735936005, 736464002, 737305006, 737306007, 74391003, 745233009, 75822003, 75879005, 7607008, 762451005, 762452003, 762454002, 762589002, 762590006, 762591005, 762593008, 762594002, 762595001, 762596000, 762597009, 762598004, 762599007, 762600005, 762601009, 762602002, 762603007, 764528008, 764529000, 76462000, 764894008, 764895009, 764896005, 764897001, 764898006, 764899003, 764900008, 764901007, 764902000, 764903005, 764904004, 764905003, 764906002, 773908008, 77994009, 781206002, 782661001, 788891004, 79267007, 80843008, 81455003, 82300000, 82304009, 82423001, 83351003, 83793004, 86119004, 86219005, 86345004, 90378003, 90641006, 90860001, 95417003, 95653008, 95655001, 95656000, 95657009, 95658004, 96531000119109, 98611000119104, 98611000119104

Supplemental Table 5: List of codes used to define procedures

Procedure Department	Procedure Type	Code Type	Codes
Orthopedics	Carpal tunnel	CPT	20526, 25000, 25001, 29848, 64721
		ICD-9	78.44
		SNOMED	171837008, 171839006, 171840008, 171841007
		ICD-10	OPNNXXX, 0PNMXXX
	Hip replacement	CPT	01215, 27125, 27130, 27132, 27134, 27137, 27138, 27236, 27258, 01214
		ICD-9	81.51
		SNOMED	15163009, 179304004, 179326003, 265106004, 265157000, 265160007, 314491003, 32581000, 340922009, 443435007, 450813004, 52734007, 711202009, 713686009, 770606008, 386649003
		ICD-10	0SR901A, 0SR901Z, 0SR9029, 0SR902A, 0SR902Z, 0SR9039, 0SR903A, 0SR903Z, 0SR9049, 0SR904A, 0SR904Z, 0SR9069, 0SR906A, 0SR906Z, 0SR907Z, 0SR90EZ, 0SR90J9, 0SR90JA, 0SR90JZ, 0SR90KZ, 0SRB019, 0SRB01A, 0SRB01Z, 0SRB029, 0SRB02A, 0SRB02Z, 0SRB039, 0SRB03A, 0SRB03Z, 0SRB049, 0SRB04A, 0SRB04Z, 0SRB069, 0SRB06A, 0SRB06Z, 0SRB07Z, 0SRB0EZ, 0SRB0J9, 0SRB0JA, 0SRB0JZ, 0SRB0KZ
	Knee osteotomy	CPT	27448, 27450, 27454, 27455, 27457, 27705, 27709, 27712, 01484
		ICD-9	77.27
		SNOMED	171837008, 171839006, 171840008, 39585008, 56582000, 171841007
		ICD-10	0G8HXXX, 0G8JXXX, 0G8KXXX, 0Q8GXXX
	ACL reconstruction	CPT	29888
		ICD-9	81.45
		SNOMED	239431009, 313315002, 314284003, 37422000, 391101007, 444889008, 54419004, 55244002, 239426007
		ICD-10	0MQPXXX, 0MQNXXX
	Lumbar interbody arthrodesis	CPT	22630, 22633, 22558
		SNOMED	10420000, 178603007, 178646005, 178647001, 265717009, 265718004, 276850007, 277764006, 278659001, 34238000, 428925003, 429541002, 448025007, 448240004, 448514006, 449448002, 705043000, 719217005, 85926009, 50172003
	Fracture or dislocation of hip and femur	CPT	27230, 27232, 27235, 27236, 27246, 27248, 27130

		SNOMED	179051005, 179064006, 179104006, 179105007, 179136004, 179137008, 179138003, 179139006, 179159005, 180315008, 429891009, 432304006, 439995008, 440302000, 440304004, 440376001, 440417002, 442957002, 708898008, 708900005, 708903007, 448243002
	Knee arthroplasty	CPT	27446, 27447, 27486, 27487, 27488, 27438
		ICD-9	0.8
		SNOMED	265170009, 265172001, 443681002, 443682009, 713687000, 609588000
		ICD-10	0SPC09Z, 0SPC0JZ, 0SPC48Z, 0SPC4JZ, 0SPD08Z, 0SPD09Z, 0SPD0JZ, 0SPD48Z, 0SPD4JZ, 0SRC069, 0SRC06A, 0SRC06Z, 0SRC0J9, 0SRC0JA, 0SRC0JZ, 0SRD069, 0SRD06A, 0SRD06Z, 0SRD0J9, 0SRD0JA, 0SRD0JZ, 0SPC08Z
Neurology	Spinal fusion	CPT	22533, 22534, 22548, 22551, 22552, 22554, 22556, 22558, 22585, 22586, 22590, 22595, 22600, 22610, 22612, 22614, 22630, 22632, 22633, 22634, 22800, 22802, 22804, 22808, 22810, 22812, 22830, 22840, 22841, 22842, 22843, 22844, 22845, 22846, 22847, 22848, 22532
		SNOMED	10420000, 1253002, 178603007, 178647001, 239546004, 239547008, 25789004, 265717009, 277764006, 278659001, 279526001, 359586003, 428547005, 428925003, 429769008, 441715004, 448514006, 448807001, 448808006, 448918002, 449048009, 449243009, 59323008, 705043000, 709292007, 709504001, 81099000, 50172003
		ICD-10	0RG00J, 0RG00K, 0RG037, 0RG03J, 0RG047, 0RG04J, 0RG04K, 0RG007, 0RG03K
	Laminectomy	CPT	22101, 22102, 22103, 62351, 63001, 63003, 63005, 63011, 63012, 63015, 63016, 63017, 63020, 63035, 63045, 63046, 63047, 63048, 63170, 63172, 63173, 63185, 63190, 63191, 63194, 63195, 63196, 63197, 63198, 63199, 63200, 63250, 63251, 63252, 63265, 63266, 63267, 63268, 63270, 63271, 63272, 63273, 63275, 63276, 63277, 63278, 63280, 63281, 63282, 63283, 63285, 63286, 63287, 63290, 63295, 63300, 63301, 63302, 63303, 63304, 63305, 63306, 63307, 63308, 63655, 22100
		SNOMED	197889000, 21089001, 2564002, 260647003, 260648008, 261540001, 283165009, 284088004, 359586003, 387731002, 429377005, 438362003, 440211003, 445429009, 448234006, 47478002, 709217002, 83471008, 83948001
		ICD-10	0R533ZZ, 0R534ZZ, 0R550ZZ, 0R553ZZ, 0R554ZZ, 0R590ZZ, 0R593ZZ, 0R594ZZ, 0R5B0ZZ, 0R5B3ZZ, 0R5B4ZZ, 0RB30ZZ, 0RB33ZZ, 0RB34ZZ, 0RB50ZZ, 0RB53ZZ, 0RB54ZZ, 0RB90ZZ, 0RB93ZZ, 0RB94ZZ, 0RBB0ZZ, 0RBB3ZZ, 0RBB4ZZ, 0RT30ZZ, 0RT40ZZ, 0RT50ZZ, 0RT90ZZ, 0RTB0ZZ, 0S520ZZ, 0S523ZZ, 0S524ZZ, 0S540ZZ, 0S543ZZ, 0S544ZZ, 0SB20ZZ, 0SB23ZZ, 0SB24ZZ, 0SB40ZZ, 0SB43ZZ, 0SB44ZZ, 0ST20ZZ, 0ST40ZZ, 0R530ZZ
	Diskectomy	CPT	22224, 22857, 22858, 62287, 63064, 63075, 22856
		ICD-9	80.5
		SNOMED	178618008, 178619000, 178620006, 178623008, 178624002, 178625001, 178626000, 265714002, 307720007, 309702009, 450837004, 448234006
	Carotid endarterectomy	CPT	35390, 35301

		ICD-9	38.12
		SNOMED	175367001, 233296007, 233297003, 233298008, 276949008, 276950008, 276951007, 405407008, 405408003, 405409006, 405411002, 405412009, 66951008
		ICD-10	03CH4ZZ, 03CJ0ZZ, 03CJ4ZZ, 03CK0ZZ, 03CK4ZZ, 03CL0ZZ, 03CL4ZZ, 03CM0ZZ, 03CM4ZZ, 03CN0ZZ, 03CN4ZZ, 03CH0ZZ
	Vertebroplasty	CPT	22511, 22512, 22510
		ICD-9	81.65
		SNOMED	401226007, 431206007, 431328005, 431496002, 431555002, 431911003, 432037000, 433034001, 433220005, 432637001
		ICD-10	0PU33JZ, 0PU34JZ, 0PU43JZ, 0PU44JZ, 0QU03JZ, 0QU13JZ, 0QU14JZ, 0QU04JZ
Obstetrics / Gynecology	Cesarean section	CPT	01968, 58611, 59100, 59510, 59514, 59515, 59525, 59610, 59612, 59614, 59618, 59620, 59622, 01961
		ICD-9	649.81, 649.82, 669.70, 669.71, 74.1, 74.2, 74.4, 74.9, 74.99, O75.82, O82.XX, 74.0
		SNOMED	156258008, 177141003, 177142005, 177143000, 177144006, 177145007, 200148001, 236985002, 236986001, 236987005, 236988000, 236989008, 236990004, 267357005, 274130007, 288042004, 41059002, 450483001, 450484007, 57271003, 709004006, 736018001, 736026009, 788180009, 84195007, 89053004, 11466000
	Hysterecomy	CPT	00944, 01962, 01963, 01969, 45126, 58150, 58152, 58180, 58200, 58210, 58240, 58260, 58262, 58263, 58267, 58270, 58275, 58280, 58285, 58290, 58291, 58292, 58293, 58294, 58541, 58542, 58543, 58544, 58545, 58546, 58548, 58550, 58552, 58553, 58554, 58570, 58571, 58572, 58573, 58575, 58661, 58951, 58952, 58953, 58954, 58956, 59525, 00846
		SNOMED	431316002, 11050006, 112918004, 116140006, 116141005, 116142003, 116143008, 120038005, 12398007, 176795006, 176895001, 17744000, 236884004, 236887006, 236888001, 24068006, 265056007, 265065000, 288042004, 288043009, 309880009, 359971002, 359974005, 359977003, 359983000, 36384005, 387643005, 387644004, 41059002, 413145007, 427107006, 447237002, 448539002, 449727003, 450692005, 54130005, 54261007, 54490004, 63516002, 699789005, 708985003, 739671004, 739672006, 740514001, 740515000, 75835007, 762625001, 767610009, 88144003, 88218008, 236886002
	Myomectomy	CPT	45108, 58145, 58146, 58545, 58546, 58140
		SNOMED	195459001, 236901008, 236902001, 265059000, 306966000, 42010004, 428652006, 446804002, 450559006
	Dilation and curettage	CPT	57522, 57558, 58120, 57520

		SNOMED	176832001, 176833006, 265062002, 274972007, 274973002, 28379004, 287927002, 391998006, 74608009, 76810006, 11401008
Otolaryngology	Thyroidectomy	CPT	60210, 60212, 60220, 60225, 60240, 60252, 60254, 60260, 60270, 60271, 60512, 60200
		ICD-9	06.4, 06.39
		SNOMED	171977001, 237486002, 24443003, 27210007, 274005006, 302338000, 30956003, 359884005, 50339005, 52814001, 52826006, 53533006, 708915006, 712978001, 719753008, 744854002, 744855001, 91276009, 13619001
		ICD-10	0GBG0ZX, 0GBG0ZZ, 0GBG3ZX, 0GBG3ZZ, 0GBG4ZX, 0GBG4ZZ, 0GBH0ZX, 0GBH0ZZ, 0GBH3ZX, 0GBH3ZZ, 0GBH4ZX, 0GBH4ZZ, 0GTK0ZZ
	Reflux surgery	CPT	43325, 43327, 43328, 43280
		SNOMED	265358004, 265359007, 265360002, 359890009, 359893006, 7161000179101, 78656005, 265357009
	Otoplasty	CPT	69300
		SNOMED	120135005, 120136006, 16443000, 172624005, 19934003, 210805003, 212525003, 232161006, 232162004, 23235002, 33901004, 350515003, 417224006, 50691002, 84705009, 52577005
General Surgery	Laparoscopic appendectomy	CPT	44979, 44970
		ICD-9	47.11, 47.01
		SNOMED	174041007, 307581005, 708876004, 6025007
		ICD-10	0DTJ4ZZ
	Laparoscopic cholecystectomy	CPT	47563, 47564, 47562
		ICD-9	51.24, 51.23
		SNOMED	20630000, 450499007, 67557008, 713872007, 45595009
		ICD-10	0FB44ZZ, 0FB48ZZ, 0FT44ZZ
	Mastectomy	CPT	19125, 19126, 19294, 19297, 19300, 19301, 19302, 19303, 19304, 19305, 19306, 19307, 19120
		ICD-9	85.42, 85.43, 85.44, 85.41

		SNOMED	12708000, 14693006, 14714006, 172044000, 172049005, 172111009, 20486005, 22418005, 237367009, 237368004, 237370008, 237393002, 237394008, 237400005, 265255003, 274957008, 27865001, 287653007, 307796007, 310638008, 318190001, 35212009, 35482003, 359728003, 359731002, 359734005, 359740003, 384723003, 392021009, 395702000, 406505007, 428554004, 428564008, 428571003, 429400009, 446109005, 446420001, 447135002, 447168009, 447421006, 451201000124106, 451211000124109, 456903003, 52314009, 59620004, 60633004, 6189002, 62347003, 64368001, 66398006, 70183006, 72432009, 72577009, 726429001, 726430006, 726434002, 726435001, 726436000, 726437009, 735085002, 735086001, 736751004, 736752006, 736753001, 736754007, 741009001, 741010006, 741018004, 741019007, 76468001, 770108008, 172043006
		ICD-10	0HTU0ZZ, 0HTV0ZZ, 0HTT0ZZ
	Open inguinal hernia repair	CPT	49568
		ICD-9	53.02, 53.03, 53.04, 53.01
		SNOMED	771714004, 771716002, 771715003
		ICD-10	0YQ50ZZ, 0YQA0ZZ, 0YU507Z, 0YU50JZ, 0YU50KZ, 0YU607Z, 0YU60JZ, 0YU60KZ, 0YUA07Z, 0YUA0JZ, 0YUA0KZ, 0YQ60ZZ
	Open appendectomy	CPT	44955, 44960, 44950
		ICD-9	47.09
		SNOMED	443935000
		ICD-10	0DTJ0ZZ
Bariatrics	Sleeve gastrectomy	CPT	43775
		ICD-9	43.89, 43.82
		SNOMED	427074001, 427980007, 87604009
		ICD-10	0DB63Z3, 0DB64Z3, 0DB67Z3, 0DB68Z3, 0DB60Z3
	Open small bowel resection	CPT	44005, 44125, 44158, 44180, 44625, 44626
	Colectomy	CPT	44140, 44141, 44143, 44144, 44145, 44146, 44147, 44150, 44151, 44155, 44156, 44157, 44158, 44160, 44204, 44205, 44206, 44207, 44208, 44210, 44211, 44212, 44213, 44626, 44701, 45121, 45126, 44139
		ICD-9	45.79, 45.71

		SNOMED	13327002, 174059005, 174081000, 26390003, 274025005, 275017002, 276190007, 287815004, 301699000, 304587000, 307507006, 307654002, 307659007, 307662005, 307666008, 31130001, 32044008, 359571009, 36192008, 386197003, 425851003, 426699005, 43075005, 443480008, 44378008, 443909001, 444165004, 445884009, 446747005, 448050005, 450462007, 54164009, 75312003, 75404005, 771568007, 787109009, 787874000, 80294005, 84604002, 84952009, 87279008, 23968004
		ICD-10	0DBE3ZZ, 0DBE7ZZ, 0DBE8ZZ, 0DBGFZZ, 0DBLFZZ, 0DBMFZZ, 0DBNFZZ, 0DTMFZZ, 0DBE0ZZ
	Bariatric surgery	CPT	43644, 43645, 43659, 43771, 43772, 43773, 43774, 43775, 43842, 43843, 43844, 43845, 43846, 43847, 43770
		SNOMED	2391000175104, 430715008
	Proctectomy	CPT	44156, 44157, 44158, 44210, 44211, 44212, 45110, 45111, 45112, 45113, 45114, 45116, 45119, 45120, 45121, 45123, 45126, 45395, 45397, 44155
		SNOMED	11626008, 174240003, 235364003, 303584001, 44751009, 55966001, 77878003, 787108001, 787109009, 31130001
Colo-Rectal	Colostomy	CPT	44143, 44144, 44146, 44160, 44188, 44205, 44206, 44208, 44320, 44322, 44340, 44345, 44346, 44388, 44390, 44391, 44392, 44141
		ICD-9	46.1X
		SNOMED	11626008, 16564004, 17671008, 24221008, 25196007, 261030007, 261072008, 261765002, 261766001, 297223007, 299681007, 299682000, 307645000, 307657009, 35535008, 37828009, 386651004, 386828002, 386829005, 387606008, 387607004, 398740003, 4044002, 44642007, 447982008, 448653006, 46295004, 49440008, 49924002, 51977003, 54629001, 62069000, 64215005, 73509004, 75312003, 89339008, 89642006, 9905009, 46070005
		ICD-10	0D1K0J4, 0D1K0K4, 0D1K0Z4, 0D1K3J4, 0D1K474, 0D1K4J4, 0D1K4K4, 0D1K4Z4, 0D1K874, 0D1K8J4, 0D1K8K4, 0D1K8Z4, 0D1L0J4, 0D1L0L4, 0D1L0Z4, 0D1L3J4, 0D1L474, 0D1L4J4, 0D1L4L4, 0D1L4Z4, 0D1L874, 0D1L8J4, 0D1L8L4, 0D1L8Z4, 0D1M074, 0D1M0J4, 0D1M0M4, 0D1M0Z4, 0D1M3J4, 0D1M474, 0D1M4J4, 0D1M4M4, 0D1M4Z4, 0D1M874, 0D1M8J4, 0D1M8M4, 0D1M8Z4, 0D1N074, 0D1N0J4, 0D1N0N4, 0D1N0Z4, 0D1N3J4, 0D1N474, 0D1N4J4, 0D1N4N4, 0D1N4Z4, 0D1N874, 0D1N8J4, 0D1N8N4, 0D1N8Z4, OD1L074, 0D1K074
	Hemorrhoidectomy	CPT	46255, 46257, 46258, 46260, 46261, 46262, 46250
		ICD-9	49.46
		SNOMED	12896001, 22432007, 235389009, 235390000, 235391001, 287802008, 442792000, 49645007, 5796001, 61498008, 24496007
		ICD-10	06BY3ZC, 06BY4ZC, 06BY0ZC
	Rectopexy	CPT	45402, 45540, 45541, 45550, 57280, 57425, 45400
		SNOMED	112877004, 2234009, 235376003, 235377007, 235378002, 235379005, 265412004, 265417005, 265418000, 265420002, 275011001, 29205000, 30402002, 440032004, 440033009, 444688002, 708642005, 73996007, 75894009, 782836001, 10259007

Transplant & Hepatobiliary Surgery	Kidney transplant	CPT	0088U, 50220, 50225, 50230, 50234, 50236, 50240, 50300, 50320, 50323, 50325, 50327, 50328, 50329, 50340, 50360, 50365, 50370, 50380, 00868
		ICD-9	55.69
		SNOMED	236436003, 428575007, 714153000
		ICD-10	0TY00Z0, 0TY00Z1, 0TY00Z2, 0TY10Z1, 0TY10Z2, 0TY10Z0
	Whipple	CPT	48150, 48152, 48153, 48154, 00794
		SNOMED	116031009, 116242006, 265458003, 287846000, 265459006
	Distal pancreatectomy	CPT	48145, 48146, 48160, 48140
		SNOMED	235468001, 235469009, 235470005, 265461002, 60194009, 91516004, 401004
	Liver transplant	CPT	47133, 47135, 47140, 47141, 47142, 47143, 47144, 47145, 47146, 47147, 00796
		ICD-9	50.59, 50.51
		SNOMED	174425003, 174426002, 174427006, 27280000, 28009009, 426356008, 18027006
		ICD-10	0FY00Z1, 0FY00Z2, 0FY00Z0
	Heart transplant	CPT	33927, 33928, 33929, 33930, 33933, 33935, 33944, 33945, 00580
		ICD-9	37.51
		SNOMED	174802006, 174808005, 174809002, 232973007, 232974001, 32477003, 405768001, 47058000, 32413006
		ICD-10	02YA0Z1, 02YA0Z2, 02YA0Z0
Cardiac	Aortic valve repair	CPT	33361, 33362, 33363, 33364, 33365, 33366, 33390, 33391, 33400, 33414, 33415, 33416, 33417, 33470, 33471, 33474, 33602, 33782, 33783, 33852, 33853, 33858, 33859, 33864, 93591, 93592, 92986
		SNOMED	174927000, 232823005, 232827006, 232843004, 265473002, 357575008, 444812008, 52247003, 232848008
	Coronary artery bypass graft	CPT	33361, 33362, 33363, 33364, 33365, 33366, 33511, 33512, 33513, 33514, 33516, 33510
		SNOMED	10326007, 119565001, 17073005, 232717009, 232720001, 232721002, 232722009, 232723004, 232724005, 309814006, 3546002, 359601003, 39202005, 405598005, 405599002, 67166004, 736966005, 736967001, 736968006, 736969003, 74371005, 82247006, 8876004, 232719007
	Implantable cardioverter-defibrillator	CPT	0575T, 0576T, 0577T, 0578T, 0579T, 33223, 33224, 33230, 33241, 33249, 33262, 33263, 33264, 4470F, 0571T
		ICD-9	37.96, 37.95
		SNOMED	360056009, 450651000124104, 462655000, 463257006, 465460004, 465652008, 465790005, 468542000, 704707009, 72506001
		ICD-10	02H60KZ, 02H63KZ, 02H64KZ, 02H70KZ, 02H73KZ, 02H74KZ, 02HK0KZ, 02HK3KZ, 02HK4KZ, 02HL0KZ, 02HL3KZ, 02HL4KZ, 02PA0MZ, 02PA3MZ, 02PA4MZ, 02PAXMZ, 0JH609Z, 0JH60FZ, 0JH638Z, 0JH639Z, 0JH63FZ, 0JH808Z, 0JH809Z, 0JH838Z, 0JH839Z, 0JPT0FZ, 0JPT0PZ, 0JPT3FZ, 0JPT3PZ, 0JH608Z

	Balloon angioplasty	CPT	37246, 37248, 61630, 92921, 92924, 92925, 92928, 92929, 92933, 92934, 92937, 92938, 92941, 92943, 92944, 92997, 92998, 92920
		SNOMED	175066001, 175354004, 230923002, 233260008, 233269009, 233284000, 233286003, 233291002, 425979008, 426674009, 428901006, 429639007, 429933006, 433711000, 433734009, 434433007, 707828002, 713155002, 713169002, 86274005
	Catheter ablation	CPT	93462, 93650, 93653, 93654, 93655, 93656, 93657
		SNOMED	702181002, 702209009, 702210004, 704706000, 717300007, 705732002
Urology	Prostatectomy	CPT	00908, 00914, 52601, 52630, 52640, 55801, 55810, 55812, 55815, 55821, 55831, 55840, 55842, 55845, 55866, 00865
		ICD-9	60.4, 60.5, 60.6, 60.3
		SNOMED	176106009, 176258007, 176260009, 176261008, 176262001, 176263006, 176267007, 176288003, 19149007, 21190008, 236209003, 236211007, 26294005, 28579000, 30426000, 314202001, 36253005, 37851009, 41371003, 41416003, 427985002, 446445003, 57525009, 65551008, 67598001, 68986004, 699077003, 708622001, 708919000, 72388004, 81232004, 83154001, 85768003, 87795007, 8782006, 90199006, 91531008, 90470006
		ICD-10	0VT04ZZ, 0VT07ZZ, 0VT08ZZ, 0VT00ZZ
	Orchiopexy	CPT	54640, 54650, 54692, 00930
		ICD-9	62.5
		SNOMED	12410002, 15791006, 176434006, 176437004, 176438009, 236340008, 25041004, 301774009, 301776006, 387668007, 387669004, 387671004, 447082006, 719441005, 719444002, 735023007, 735024001, 762902004, 762903009, 767331003, 767332005, 767378002, 767379005, 89844005, 85419002
		ICD-10	0VS93ZZ, 0VS94ZZ, 0VS98ZZ, 0VSB0ZZ, 0VSB3ZZ, 0VSB4ZZ, 0VSB8ZZ, 0VSC0ZZ, 0VSC3ZZ, 0VSC4ZZ, 0VSC8ZZ, 0VS90ZZ
	Vasectomy	CPT	0421T, 52402, 52601, 52630, 52647, 52648, 52649, 55250, 55400, 55801, 55821, 55831, 00921
		ICD-9	63.73
		SNOMED	17293009, 276487005, 287662009, 47655001, 22523008
		ICD-10	0VBQ3ZZ, 0VBQ4ZZ, 0VBQ8ZZ, 0VBQ0ZZ
	Cystoplasty	CPT	5180050825
		ICD-9	57.89, 57.87
		SNOMED	176117005, 176118000, 176165006, 176166007, 277837003, 361256009, 361257000, 386652006, 48007004
		ICD-10	0TQBXXX, 0TRBXXX, 0TUBXXX
	Lithotripsy	CPT	00873, 43265, 47544, 50080, 50081, 50590, 51065, 52325, 52353, 52356, 00872
		ICD-9	98.52, 98.51

		SNOMED	175951002, 175952009, 175953004, 176050007, 176058000, 176064007, 20042009, 235525000, 235547000, 236172004, 236173009, 236179008, 236180006, 236190003, 24376003, 274446006, 274447002, 274448007, 287586004, 37597002, 386189005, 386198008, 404812006, 42041003, 425577000, 427001005, 427278007, 431401004, 431494004, 431847003, 432032006, 432036009, 432649000, 432650000, 432880008, 433137006, 44345001, 446125000, 446297008, 446587003, 446685001, 44817001, 4501000087102, 450497009, 450498004, 4511000087100, 53514001, 61417004, 713163001, 713164007, 82920008, 90705003, 133864008
		ICD-10	0FF4XZZ, 0TF3XZZ, 0TF4XZZ, 0TF7XZZ, 0TFBXZZ, 0TFCXZZ, 0TF6XZZ
	Angioplasty	CPT	35879, 36903, 36905, 36906, 36907, 36908, 37186, 37215, 37216, 37217, 37218, 37220, 37221, 37222, 37223, 37224, 37225, 37226, 37227, 37228, 37229, 37230, 37231, 37232, 37233, 37234, 37235, 37236, 37237, 37238, 37239, 37246, 37247, 37248, 37249, 61630, 61635, 61640, 61641, 61642, 36902
		SNOMED	233259003, 405326004, 405407008, 405408003, 405409006, 405411002, 405415006, 417884003, 419014003, 420026003, 420046008, 429287007
Vascular Surgery	Atherectomy	CPT	0234T, 0235T, 0236T, 0237T, 0238T, 37227, 37229, 37231, 37233, 37235, 92924, 92933, 92937, 92941, 92943, 37225
		SNOMED	126062009, 15101009, 175764006, 175882004, 20500004, 230922007, 232727003, 232728008, 232729000, 232980009, 232988002, 232996007, 233002007, 233214006, 233258006, 233261007, 233262000, 233263005, 233266002, 233268001, 233270005, 233272002, 233275000, 233277008, 233290001, 233476002, 24088005, 257765008, 257766009, 261562001, 27659003, 27672002, 276861004, 276890005, 276897008, 29843007, 301429003, 301430008, 312610006, 346007006, 359551000, 373360004, 373363002, 397194000, 405395009, 405396005, 405410001, 405454007, 405593001, 410023005, 41339005, 415069007, 418076001, 418269003, 419224002, 419801000, 419806005, 419827000, 425712000, 428811000, 429287007, 429813006, 429814000, 429815004, 429816003, 431253005, 431522007, 431523002, 431581003, 431752001, 431759005, 431932006, 432640001, 432866001, 432867005, 441540009, 442881005, 443038001, 443513002, 446051001, 446052008, 446878003, 448754006, 448798006, 51158001, 5431005, 58622000, 609167004, 65659003, 6832004, 698740005, 70131004, 708872002, 708873007, 708874001, 709019007, 709021002, 709862009, 709863004, 709981002, 710161003, 711222008, 723683000, 726555002, 7414004, 76611008

		ICD-10	02C13Z7, 02C23Z7, 02C33Z7, 02C03Z7
	Endarterectomy	CPT	03CL0ZZ, 33501, 33502, 33503, 33504, 33505, 33506, 33507, 33572, 33916, 35301, 35302, 35303, 35304, 35305, 35306, 35311, 35321, 35331, 35341, 35351, 35355, 35361, 35363, 35371, 35372, 35390, 38.1X, 33500
		SNOMED	14259004, 15023006, 15853003, 16589005, 16903003, 17437005, 175346001, 175367001, 175418002, 175419005, 175423002, 175424008, 175425009, 175477003, 175514002, 175689005, 175690001, 19606007, 19953008, 20738002, 21260009, 232726007, 233296007, 233297003, 233298008, 233299000, 233300008, 233301007, 233302000, 233303005, 233310004, 240923000, 240925007, 26076006, 265517007, 275064002, 276921001, 276922008, 276923003, 276925005, 276926006, 276927002, 276928007, 276929004, 276930009, 276931008, 276932001, 276933006, 276937007, 276939005, 276942004, 276946001, 276949008, 276950008, 307800001, 35653006, 35888008, 37199006, 38260008, 38863001, 40455006, 405337003, 405338008, 405343001, 405389008, 405392007, 405395009, 405396005, 405397001, 405398006, 405399003, 405400005, 405401009, 405402002, 405403007, 405406004, 405407008, 405410001, 405411002, 405412009, 405454007, 405455008, 405456009, 415071007, 415072000, 43418007, 44620006, 447746007, 448341002, 448534007, 448939009, 450513009, 450696008, 49187005, 52005005, 66951008, 68324008, 71198003, 72396009, 72672007, 74556005, 7642007, 77163008, 77445003, 82467004, 83662005, 84594003, 85448008, 87742009, 90499001, 392031002
	Endovascular stent graft	CPT	34813, 37221, 37223, 37226, 37227, 37230, 37231, 33891
		SNOMED	434159001, 434378006, 433591001
	Bypass surgery	CPT	33315, 33916, 35523, 35535, 35537, 35538, 35539, 35540, 35570, 35632, 35633, 35634, 35637, 35638, 35686, 35883, 35884, 33310
		SNOMED	10190003, 10326007, 119565001, 14323007, 17073005, 175045009, 232717009, 232719007, 232720001, 232721002, 232722009, 232723004, 232724005, 29819009, 309814006, 3546002, 359601003, 39202005, 405598005, 405599002, 67166004, 736962007, 736963002, 736964008, 736965009, 736966005, 736967001, 736968006, 736969003, 736970002, 736971003, 736972005, 736973000, 74371005, 82247006, 8876004, 90487008, 175021005
Plastic Surgery	Liposuction	CPT	15771, 15772, 15773, 15774, 15819, 15876, 15877, 15878, 15879, 0566T
		ICD-9	86.9

		SNOMED	15039004, 177252003, 177710009, 444162001, 75674002, 8085000, 302441008
		ICD-10	0JDXXXX
	Rhinoplasty	CPT	30410, 30420, 30430, 30435, 30450, 30460, 30462, 30400
		SNOMED	11759002, 172761005, 172773005, 172786001, 172796005, 172842002, 172848003, 172849006, 172850006, 20805001, 232463004, 232464005, 232466007, 232467003, 232468008, 232482008, 265021008, 265023006, 315312002, 32010001, 360754002, 360762005, 415302008, 45474001, 52103001, 53356001, 704040006, 704041005, 704053009, 72009003, 72767005, 736513001, 8142006, 82862002, 172765001
	Breast augmentation/implant	CPT	19318, 19325, 19328, 19330, 19350, 19355, 19357, 19361, 19364, 19367, 19368, 19369
		SNOMED	172079003, 172080000, 172081001, 287649005, 287650005, 287651009, 443180000, 45467006, 22890008
	Abdominoplasty	CPT	15847, 17999, 15830
		ICD-9	86.89
		SNOMED	177252003, 240972007, 240973002, 240974008, 240975009, 442209001, 59716009, 177250006
		ICD-10	0W0F0ZZ
Burn Surgery	Laryngoplasty	CPT	31552, 31553, 31554, 31580, 31582, 31584, 31587, 31588, 31591, 31599, 31551
		SNOMED	38724002
	Escharotomy	CPT	15003, 15004, 15005, 16035, 16036, 15002
		SNOMED	177652008, 177653003, 177654009, 177656006, 177659004, 240992000, 449665001, 449666000, 449667009, 449668004, 70177008

Supplemental Table 6: Mental health codes by mental health disorder classification

Mental Health Disorder	Code Type	Code(s)
Anxiety Disorders	ICD-9	293.84, 300.10, 300.20, 300.21, 300.22, 300.3, 300.0X, 308.X
	SNOMED	48694002, 197480006, 21897009, 192404005, 21897009, 52910006, 160332003, 154882009, 268752000, 191706008, 192401002, 192459009, 191720001, 111487009, 16265701000119107, 17496003, 192398000, 192400001, 192405006, 197480006, 154884005, 191703000, 192192006, 65673007, 126943008, 192393009, 192397005, 192399008, 192403004, 268714001, 436001000124105, 69479009, 371631005, 386810004, 25501002, 52039009, 191736004, 71478004, 1376001, 191739006, 192411009, 192406007, 192410005, 192394003, 414371008, 111490003, 111491004, 11941006, 1380006, 1816003, 191722009, 191723004, 19766004, 22230001, 24781009, 30059008, 3158007, 31781004, 32388005, 34116005, 35607004, 38328002, 43150009, 4932002, 49564006, 50983008, 53956006, 5509004, 56576003, 59923000, 61212007, 61569007, 63701002, 63909006, 64060000, 65064003, 70691001, 72861004, 74010007, 76812003, 76868007, 8185002, 82415003, 82494000, 82738004, 83631006, 87798009, 89948007, 154885006
	ICD-10	F06.4, F40.0X, F41.X, F42.X
Depression	ICD-9	296.89, 300.4, 309.1, 311, 296.2X, 296.3X
	SNOMED	25922000, 35489007, 320751009, 36923009, 370143000, 42925002, 69392006, 63778009, 87512008, 79298009, 48589009, 832007, 15639000, 73867007, 75084000, 430852001, 77911002, 20250007, 76441001, 19527009, 14183003, 300706003, 231499006, 321717001, 63412003, 30605009, 42810003, 70747007, 726772006, 320751009, 36923009, 370143000, 10811121000119102, 10811161000119107, 42925002, 69392006, 63778009, 87512008, 79298009, 16265951000119109, 720455008, 720454007, 832007, 15639000, 16266831000119100, 719592004, 720453001, 450714000, 73867007, 33736005, 60099002, 75084000, 251000119105, 430852001, 77911002, 20250007, 76441001, 16266991000119108, 19527009, 191606003, 191601008, 191602001, 191604000, 2506003, 19694002, 83176005, 38451003, 67711008, 85080004, 3109008, 36170009, 78667006, 2506003, 19694002, 83176005, 38451003, 67711008, 85080004, 3109008, 36170009, 191659001, 192080009, 35489007, 40379007, 191610000, 40379007, 191610000, 18818009, 191611001, 18818009, 191611001, 36474008, 281000119103, 36474008, 39809009, 191613003, 28475009, 33078009, 15193003, 39809009, 191613003, 28475009, 33078009, 15193003, 68019004, 33135002, 46244001, 191615005, 40568001, 38694004, 274948002, 38694004, 2618002, 191616006, 66344007, 319768000, 71336009, 268621008, 2618002, 720451004, 720452006, 66344007, 319768000, 71336009, 268621008
	ICD-10	F31.4, F31.5, F31.75, F31.76, F31.77, F31.78, F31.81, F32.0, F32.1, F32.3, F32.4, F32.5, F32.9, F33, F34.1, F34.21, F34.23, F31.3X, F31.6X, F33.X
ADD/ADHD	ICD-9	314.X
	SNOMED	406506008, 35253001, 35253001, 192127007, 31177006
	ICD-10	F90.X
Bipolar Disorder	ICD-9	296.X
	SNOMED	191627008, 191618007, 192362008, 191634005, 191629006, 191630001, 191632009, 191625000, 191620005, 191621009, 191623007, 13746004,

		41836007, 5703000, 85248005, 371596008, 29929003, 21900002, 75752004, 87203005, 31446002, 17782008, 55516002, 73471000, 65042007, 83225003, 48937005, 16295005, 43568002, 22407005, 34315001, 30687003, 51637008, 1196001, 49468007, 22121000, 49512000, 53607008, 68569003, 30935000, 63249007, 45479006, 13313007, 41552001, 71294008, 74686005, 71984005, 43769008, 191636007, 191643001, 191638008, 191639000, 191641004, 16506000, 111485001, 36583000, 35481005, 79584002, 28884001, 35846004, 66631006, 82998009, 40926005, 371600003, 4441000, 70546001, 26530004, 53049002, 371599001, 14495005, 371604007, 30520009, 19300006, 20960007, 81319007, 12969000, 67002003, 35722002, 59617007, 54761006, 26203008, 61403008, 28663008, 78640000, 33380008, 162004, 10981006, 64731001, 10875004, 46229002, 191627008, 191618007, 192362008, 191634005, 191629006, 191630001, 191632009, 191625000, 191620005, 191621009, 191623007, 13746004, 41836007, 5703000, 85248005, 371596008, 87203005, 31446002, 17782008, 55516002, 73471000, 65042007, 83225003, 48937005, 16295005, 43568002, 22407005, 34315001, 30687003, 51637008, 1196001, 49468007, 22121000, 49512000, 53607008, 68569003, 30935000, 63249007, 45479006, 13313007, 41552001, 71294008, 74686005, 71984005, 43769008, 191636007, 191643001, 191638008, 191639000, 191641004, 16506000, 111485001, 36583000, 35481005, 79584002, 28884001, 35846004, 66631006, 82998009, 40926005, 133091000119105, 371600003, 4441000, 70546001, 26530004, 53049002, 371599001, 14495005, 371604007, 19300006, 20960007, 81319007, 261000119107, 61403008, 23741000119105, 28663008, 78640000, 33380008, 162004, 271000119101, 10981006, 64731001, 10875004, 46229002, 81319007
	ICD-10	F31.X
Schizophrenic/Psychotic Disorders	ICD-9	295.X, 297.X, 298.X
	SNOMED	58214004, 191527001, 191567000, 69322001
	ICD-10	F20.X, F22.X, F23.X, F24.X, F28.X, F29.X
PTSD	ICD-9	309.81
	SNOMED	47505003, 313182004, 443919007, 318784009, 192042008
	ICD-10	F43.1X
Other	ICD-9	301.X, 307.X, 308.X, 309.0X, 309.1X, 309.2X, 309.3X, 309.4X, 312.X, 313.X
	SNOMED	72366004, 439960005, 33449004, 55341008, 20010003, 80711002, 698700004, 20010003, 191765005, 4306003
	ICD-10	F30.X, F39.X, F43, F43.0, F43.2, F43.8, F43.9, F44.X, F45.X, F48.X, F50.X, F53.X, F54.X, F60.X, F63.X, F65.X, F68.X, F69.X, F91.X, F93.X, F94.X, F95.X, F98.X, F99.X

Supplemental Table 7a: List of codes used to define medications for opioid use disorder (MOUD) treatment

Medication	Code Type	Codes
buprenorphine (Brixadi, Bunavail, Cassipa, Probuphine, Sublocade, Suboxone, Subutex, Zubsolv)	NDC	54017613, 54017713, 54018813, 54018913, 93537856, 93537956, 93572056, 93572156, 228315303, 228315403, 228315473, 228315503, 228315567, 228315573, 228315603, 378092393, 378092493, 378876716, 378876793, 378876816, 378876893, 406192303, 406192403, 406800503, 406802003, 490005100, 490005130, 490005160, 490005190, 781721606, 781721664, 781722706, 781722764, 781723806, 781723864, 781724906, 781724964, 12496010001, 12496010002, 12496010005, 12496030001, 12496030002, 12496030005, 12496120201, 12496120203, 12496120401, 2496120403, 12496120801, 12496120803, 12496121201, 12496121203, 12496127802, 12496128302, 2496130602, 12496131002, 16590066605, 16590066630, 16590066705, 16590066730, 16590066790, 3490927003, 23490927006, 23490927009, 35356000407, 35356000430, 35356055530, 35356055630, 2291017430, 42291017530, 42858050103, 42858050203, 43063018407, 43063018430, 43063066706, 3063075306, 43598057901, 43598057930, 43598058001, 43598058030, 43598058101, 43598058130, 3598058201, 43598058230, 47781035503, 47781035511, 47781035603, 47781035611, 47781035703, 7781035711, 47781035803, 47781035811, 49999039507, 49999039515, 49999039530, 49999063830, 9999063930, 50090292400, 50268014411, 50268014415, 50268014511, 50268014515, 50383028793, 0383029493, 50383092493, 50383093093, 52427069203, 52427069211, 52427069403, 52427069411, 2427069803, 52427069811, 52427071203, 52427071211, 52440010014, 52959030430, 52959074930, 3217013830, 53217024630, 54123011430, 54123090730, 54123091430, 54123092930, 54123095730, 4123098630, 54569549600, 54569573900, 54569573901, 54569573902, 54569639900, 54569640800, 4569657800, 54868570700, 54868570701, 54868570702, 54868570703, 54868570704, 54868575000, 5045378403, 55700014730, 55700018430, 55700030230, 55700030330, 58284010014, 59385001201, 9385001230, 59385001401, 59385001430, 59385001601, 59385001630, 60429058611, 60429058630, 0429058633, 60429058711, 60429058730, 60429058733, 62175045232, 62175045832, 62756045983, 2756046083, 62756096983, 62756097083, 63629402801, 63629403401, 63629403402, 63629403403, 3629409201, 63874108403, 63874108503, 63874117303, 65162041503, 65162041603, 66336001630, 8071138003, 68071151003, 68258299103, 68258299903, 68308020230, 68308020830
	MMSL Synonym	1819, 12512, 24676, 45125, 45126, 45128, 45204, 45205, 45231, 45232, 45311, 45312, 45342, 45343, 72461, 72462, 72463, 176533, 176535, 176536, 176558, 186680, 196662, 196663, 196664, 196665, 203841, 204727, 204728, 204743, 204744, 204745, 206241, 227088, 227090, 227093, 227095, 227101, 227102, 227103, 233047, 233048, 236126, 236127, 236128, 236129, 237313, 237314, 237315, 237316, 237317, 237318, 237319, 241413, 241420, 241421, 254927, 254928, 267706, 267707, 267708, 267709, 267710, 351264, 351265, 351266, 351267, 352364, 352990, 904870, 904876, 904880, 1010600, 1010603, 1010604, 1010606, 1010608, 1307056, 1307058, 1307061, 1307063, 1431077, 1431083, 1431104, 1432969, 1542391, 1542997, 1544853, 1597570, 1597575, 1666385, 1996189, 199619
	MMSL Drug ID	d00840, d04819
	HCPCS	G2068, G2069, G2070, G2071, G2072, G2079, J0571, J0572, J0573, J0574, J0575, J0592
naltrexone (Vivitrol, Revia, Depade)	NDC	001850039, 004061170, 005550902, 009047036, 167290081, 422910632, 430630469, 430630591, 473350326, 500902866, 500903076, 500903929, 500904925, 504360105, 512240206, 521250727, 532170261, 548685574, 621350242, 636291046, 636291047, 636295304, 680712156, 680712721, 680840291, 680940853, 687887084, 691890499, 693643143, 705181146, 705181312, 705182718, 713350014, 713351480, 713352062, 721621566, 765191160, 657570300, 657570301, 512850275, 56001122, 56001130, 56001170, 56007950, 56008050, 185003901, 185003930, 406009201, 406009203, 406117001, 406117003, 555090201, 555090202, 16729008101, 16729008110, 42291063230, 43063059115, 47335032683, 47335032688, 50090286600, 50436010501, 51224020630, 51224020650, 51285027501, 51285027502, 52152010502, 52152010504, 52152010530, 54868557400, 63459030042, 63629104601, 63629104701, 65694010003, 65694010010, 65757030001, 65757030202, 68084029111, 68084029121, 68094085362, 68115068030
	MMSL Synonym	167360, 12838, 72424, 228674, 18420, 167361, 167363, 167359, 167323, 81542, 11621, 136490, 136489, 228676, 187249, 167362, 137294, 244063, 167364, 81544, 81543, 1157, 15708
	MMSL Drug ID	d01406, d07135, d07472
	HCPCS	G0723, J2315
	ICD-10 Procedure	HZ84ZZZ, HZ94ZZZ

Methadone	HCPCS	G2067, G2078, H0020, S0109, J1230
	ICD-10 Procedure	HZ81ZZZ, HZ91ZZZ
Other types	ICD-10 Procedure	HZ82ZZZ, HZ85ZZZ, HZ86ZZZ, HZ92ZZZ, HZ95ZZZ, HZ96ZZZ

Supplemental Table 7b: List of codes used to define medications for alcohol use disorder (MAUD) treatment

Medication	Code Type	Codes
acamprosate (Campral)	NDC	000935352, 002584000, 003786333, 009047213, 101350636, 422910104, 510790241, 606870121, 683820569, 684620435, 691890437, 707711057, 004563330, 681514760
	MMSL Synonym	48384, 48401, 59284, 59283
	MMSL Drug ID	d04986
disulfiram (Antabuse)	NDC	000540356, 000540357, 000935035, 000935036, 003784140, 003784141, 006033432, 006033433, 427940028, 477810607, 604290196, 621350431, 621350432, 636296854, 649800171, 649800172, 681512694, 512850523, 512850524, 548685034
	MMSL Synonym	12827, 18860, 18861, 17279, 17278
	MMSL Drug ID	d01389
	ICD-10 Procedure	HZ83ZZZ, HZ93ZZZ
topiramate (Topamax, Topiragen, Eprontia, Qudexy, Trokendi)	NDC	000930155, 000937219, 000937220, 000937335, 000937336, 000937540, 001439755, 001439756, 001439757, 001439758, 002450707, 002450708, 002450709, 002450710, 003786101, 003786102, 003786103, 003786105, 004802356, 004802357, 004802358, 004802359, 006157562, 006157563, 006157564, 006157565, 006158138, 006158139, 006158140, 008320707, 008320708, 008320709, 008320710, 008321071, 008321072, 008321073, 008321074, 008321075, 009046016, 009046017, 009046018, 009046928, 009046929, 103700365, 103700366, 103700367, 103700368, 105440489, 105440628, 105440847, 126340083, 126340452, 126340453, 136680031, 136680032, 136680033, 136680034, 162520568, 162520569, 165710705, 165710706, 165710707, 165710708, 165900817, 165900824, 165900825, 216950162, 216950205, 216950348, 216950349, 272410227, 272410228, 272410229, 272410230, 293000115, 293000116, 293000117, 293000118, 317220181, 317220182, 317220183, 317220184, 317220278, 317220279, 317220280, 317220281, 332610106, 332610400, 332610480, 353560469, 353560470, 353560471, 353560472, 425490619, 425490620, 425490621, 430630094, 430630114, 430630189, 430630417, 430630436, 430630538, 430630573, 430630605, 430630612, 430630729, 430630734, 430630735, 430630997, 430630998, 433530695, 433530696, 433530697, 436020457, 436020458, 436020459, 436020460, 458650459, 458650551, 458650963, 473350707, 473350710, 473350711, 473350712, 493490116, 493490118, 493490142, 493490365, 493490395, 493490402, 493490705, 493490778, 493490802, 493490820, 493490907, 493490942, 493490993, 500901137, 500901138, 500901139, 500901676, 500901721, 500901741, 500901755, 500901962, 500902102, 500902203, 500902248, 500903389, 500903461, 500903752, 500903926, 500903931, 500903932, 500904614, 500905309, 500905853, 502680750, 502680751, 502680752, 502680753, 504360139, 504361220, 504369949, 504369950, 504369951, 504369952, 510790726, 510790727, 510790728, 516550032, 516550429, 516550606, 516550608, 516550609, 516550746, 516550848, 521250047, 521250061, 521250089, 521250463, 521250852, 521250876, 521250914, 529590441, 529590643, 529590994, 530021547, 532170274, 532170291, 532170315, 532170329, 538080921, 538080932, 538080967, 538081131, 538081132, 545696137, 545696138, 545696139, 548686014, 548686015, 548686016, 548686017, 551545371, 551545372, 551547142, 551547146, 557000210, 557000217, 557000226, 557000227, 557000228, 557000596, 557000691, 557000894, 557000995, 572370134, 572370135, 572370136, 572370137, 581180707, 581180710, 581180711, 581180712, 591150124, 591150125, 591150126, 591150127, 597621030, 597621031, 597621032, 597621033, 604290769, 604290770, 604290771, 604290772, 605052760, 605052761, 605052762, 605052763, 606870108, 607230031, 607230032, 607230033, 607600075, 607600094, 607600278, 607600279, 607600280, 607600287, 607600514,

	607600577, 617860293, 617860298, 617860580, 617860611, 617860623, 617860668, 617860732, 617860736, 617860863, 617860996, 619190172, 619190185, 619190190, 619190212, 619190318, 619190369, 619190439, 619190679, 619190691, 619190817, 619190823, 619190824, 619190825, 619190883, 627560707, 627560710, 627560711, 627560712, 631870059, 631870060, 631870077, 631870118, 631870228, 631870230, 631870283, 631870479, 631870696, 631870758, 631870773, 631870801, 631870963, 633040778, 633040779, 633040780, 636293294, 636293994, 636293995, 636294947, 636296431, 636296871, 636298189, 647250707, 647250710, 658410647, 658410648, 658410649, 658410650, 658410651, 658410652, 658620171, 658620172, 658620173, 658620174, 680711612, 680711900, 680711960, 680711971, 680713012, 680713088, 680713196, 680714756, 680714760, 680714793, 680714877, 680715125, 680715165, 680840342, 680840343, 680840344, 680840345, 681800170, 681800171, 681800172, 681800173, 682583000, 682583001, 682583002, 682587056, 682587156, 682587159, 683820004, 683820005, 683820138, 683820139, 683820140, 683820141, 683820769, 683820863, 683820864, 683870558, 683870559, 683870560, 684620108, 684620109, 684620110, 684620153, 684620370, 684620371, 684620372, 684620373, 684620374, 687886366, 687886377, 687886417, 687886435, 687886770, 687886824, 687886916, 687887010, 687887016, 687887351, 687887460, 687887468, 687887568, 687888965, 687888967, 687889499, 687889532, 687889533, 687889730, 690970122, 690970123, 690970124, 690970125, 690970816, 690970817, 690970818, 690970819, 705180233, 705180333, 705180344, 705180370, 705180420, 705180517, 705180625, 705180887, 705181180, 705181196, 705181503, 705181651, 705181674, 705181718, 705181853, 705181937, 705181953, 705182001, 705182104, 705182322, 705182391, 705182419, 705182498, 705182517, 705182899, 705182955, 705183067, 705183460, 705183758, 707101039, 707101040, 707101041, 707101042, 707101043, 707711315, 707711316, 707711317, 707711656, 707711657, 707711658, 707711659, 707711660, 709340022, 709340133, 709340149, 709340420, 709340450, 709340606, 709340733, 709340748, 709340768, 709340772, 712050187, 712050195, 712050202, 712050214, 712050233, 712050819, 713350047, 713350325, 713350337, 713350359, 713350487, 713350494, 713350499, 713350538, 713350548, 713350925, 713351064, 713351116, 713351143, 713351147, 713351684, 713359727, 716100193, 716100485, 716100486, 716100492, 716100493, 726030120, 726030121, 726030122, 726030123, 726030124, 727890004, 762820278, 762820279, 762820280, 762820281, 764200276, 764200278, 764200279, 764200280, 804250092, 804250195, 804250202, 804250208, 804250214, 804250288, 820090135, 820090136, 820090137, 830080011, 830080012, 216950128, 216950129, 216950130, 504580639, 504580640, 504580641, 504580642, 504580645, 504580647, 548684672, 548684674, 548685190, 548685343, 552890433, 619190189, 636293321, 705181393, 705182165, 705182753, 526529001, 002451071, 002451072, 002451073, 002451074, 002451075, 177720101, 177720102, 177720103, 177720104
MMSL Synonym	205293, 18525, 19089, 353803, 343382, 205295, 19088, 162273, 19087, 205296, 213505, 343383, 13669, 49828, 192126, 343379, 205294, 192128, 19090, 343576, 205302, 343380, 192129, 192130, 192131, 65036, 65035, 49829, 6438, 16508, 17041, 17040, 13957, 170342, 170344, 170343, 170345, 170341, 353814, 353813, 343382, 213511, 343383, 213510, 213507, 343379, 213506, 213509, 213508, 343576, 343380, 205299, 205297, 205298, 205300, 205301
MMSL Drug ID	d04115, d07881

naltrexone (Vivitrol, Revia, Depade)	NDC	001850039, 004061170, 005550902, 009047036, 167290081, 422910632, 430630469, 430630591, 473350326, 500902866, 500903076, 500903929, 500904925, 504360105, 512240206, 521250727, 532170261, 548685574, 621350242, 636291046, 636291047, 636295304, 680712156, 680712721, 680840291, 680940853, 687887084, 691890499, 693643143, 705181146, 705181312, 705182718, 713350014, 713351480, 713352062, 721621566, 765191160, 657570300, 657570301, 512850275, 56001122, 56001130, 56001170, 56007950, 56008050, 185003901, 185003930, 406009201, 406009203, 406117001, 406117003, 555090201, 555090202, 16729008101, 16729008110, 42291063230, 43063059115, 47335032683, 47335032688, 50090286600, 50436010501, 51224020630, 51224020650, 51285027501, 51285027502, 52152010502, 52152010504, 52152010530, 54868557400, 63459030042, 63629104601, 63629104701, 65694010003, 65694010010, 65757030001, 65757030202, 68084029111, 68084029121, 68094085362, 68115068030
	MMSL Synonym	167360, 12838, 72424, 228674, 18420, 167361, 167363, 167359, 167323, 81542, 11621, 136490, 136489, 228676, 187249, 167362, 137294, 244063, 167364, 81544, 81543, 1157, 15708
	MMSL Drug ID	d01406, d07135, d07472
	HCPCS	G0723, J2315
	ICD-10 Procedure	HZ84ZZZ, HZ94ZZZ

Supplemental Table 7c: List of codes used to define medications for tobacco use disorder (MTUD), psychotropic and general SUD treatment

Type	Medication	Code Type	Codes
MTUD	Medications	NDC	00024581030, 00024581130, 00069046856, 00069046903, 00069046912, 00069046956, 00069046997, 00069047102, 00069047103, 00069047197, 00093081001, 00093081005, 00093081101, 00093081105, 00093081201, 00093081205, 00093081301, 00093570301, 00115544513, 00115681102, 00115681108, 00115681110, 00121067816, 00173013555, 00173017755, 00173017855, 00173055601, 00173055602, 00173072200, 00173073001, 00173094755, 00185041001, 00185041005, 00185041060, 00185041501, 00185041505, 00185041560, 00185111160, 00187073030, 00187073090, 00187073130, 00187581030, 00187581130, 00187581230, 00378043301, 00378043305, 00378043501, 00378043505, 00378200905, 00378552101, 00406991003, 00406991103, 00406991203, 00406991303, 00527241532, 00527243032, 00591333105, 00591333119, 00591333130, 00591333205, 00591333230, 00591354005, 00591354060, 00591354105, 00591354160, 00591354260, 00591354360, 00781105301, 00781106401, 00781516960, 00781552810, 00781552910, 00904657304, 00904658561, 00904663561, 00904663661, 00904708404, 00904708461, 10370010103, 10370010150, 10370010203, 10370010250, 16571086250, 16590024630, 16729044310, 16729044315, 16729044316, 16729044410, 16729044416, 21695063356, 23155019101, 23155019201, 23490923006, 23490923103, 23490923203, 24689011901, 24979010207, 35356008730, 35356008760, 35356036960, 42806034809, 42806034905, 42806034909, 42806041501, 43547028810, 43547028910, 43547028950, 43547029010, 43598065530, 43598075105, 43598075160, 43598075201, 43598075360, 43598086360, 45963014205, 45963014290, 47781063730, 49884015576, 49884015676, 49884094499, 49909001030, 50268014013, 50268060315, 50268060415, 51079004720, 51079094320, 51079094420, 51407020605, 51672400101, 51672400201, 51672400205, 51672400305, 51672400401, 51862001505, 51862001601, 51862001605, 51862001610, 51862001705, 51862001801, 51862094505, 51862094601, 51862094605, 51862094705, 51862094801, 52427057530, 52959028530, 52959086930, 54868476300, 54868566400, 54868567400, 55289090015, 55289090030, 57866308305, 58016059990, 60505015701, 60505015801, 60505476505, 60505476606, 60687028101, 60687029301, 60687034001, 60687035101, 64679083001, 64980020101, 67767013305, 67767014190, 68001030800, 68001032203,

			68001052004, 68084025221, 68084069701, 68084070825, 68180031902, 68180031906, 68180031909, 68180032002, 68180032006, 68180032009, 68382035305, 69097087512, 69097087712, 69097087812, 69097087907, 69097091707, 69097091807, 70436001002, 70436001004, 70436001006, 70436001102, 70436001104, 70436005901, 70436005902, 70436005922, 76282048130
		MMSL Drug ID	d00144, d00181, d05807, d08288
		MMSL Synonym	2738, 4319, 6125, 7531, 7866, 10183, 10184, 12079, 12112, 16938, 16939, 16940, 16941, 17734, 18232, 18272, 18770, 20202, 20461, 20462, 20463, 20464, 23091, 25304, 25642, 25643, 26869, 26870, 27437, 31548, 42347, 42568, 43167, 44154, 44267, 47392, 47393, 47394, 47395, 47396, 47397, 47868, 82164, 82169, 82170, 82172, 82173, 82174, 82175, 82176, 110992, 147258, 147259, 162120, 162121, 162122, 162123, 162131, 166175, 166176, 186279, 186280, 188113, 188114, 193448, 193454, 193455, 198045, 198046, 198047, 203130, 203204, 209329, 209339, 209350, 209391, 227948, 228674, 228676, 312036, 317136, 328261, 329425, 329426, 329427, 351831, 351951, 351952, 361181, 361182, 581650, 591622, 636230, 636671, 636674, 636676, 637188, 637190, 749289, 749788, 794222, 795735, 795737, 835486, 993503, 993511, 993518, 993524, 993528, 993536, 993537, 993541, 993542, 993545, 993552, 993557, 993559, 993564, 993569, 993683, 993687, 993688, 993691, 993693, 993954, 1232585, 1232591, 1551467, 1551474, 1801289, 2572371, 2611266
	Procedures	ICD-10	HZ80ZZZ, HZ87ZZZ, HZ90ZZZ, HZ97ZZZ
Psychotropic SUD	Procedures	ICD-10	HZ88ZZZ, HZ98ZZZ
General SUD	Procedures	ICD-10	HZ89ZZZ, HZ99ZZZ
		HCPCS	G2074, G2075, G2080

Supplemental Table 8: List of codes used to define complementary medicine (CM)

CM Type[1]	Code Type	Code(s)
Acupuncture	ICD-9	99.92, 99.91
	SNOMED	182614009, 231088001, 231111004, 44868003, 182615005, 231081007, 231082000, 231100007, 231101006, 231102004, 231103009, 231104003, 231105002, 1251553005
	ICD-10	8E0H30Z, 8E0H300
	CPT	97810, 97811, 97813, 97814
	HCPCS	S8930
Chiropractic Care	SNOMED	913000, 127812007, 16187000, 18362009, 23457006, 2530001, 28443006, 29367007, 29419003, 33503006, 46893003, 46947000, 49332003, 58400001, 64133005, 722170006, 746002, 77866002, 84790009, 88419003, 182548004
	ICD-10	9WB0XBZ,9WB0XCZ, 9WB0XDZ, 9WB0XFZ, 9WB0XKZ, 9WB0XLZ, 9WB4XBZ, 9WB4XCZ, 9WB4XDZ, 9WB4XFZ, 9WB4XKZ, 9WB4XLZ, 9WB5XBZ, 9WB5XCZ, 9WB5XDZ, 9WB5XFZ, 9WB5XKZ, 9WB5XLZ, 9WB9XBZ, 9WB9XCZ, 9WB9XDZ, 9WB9XFZ, 9WB9XKZ, 9WB9XLZ, 9WB0XGZ, 9WB0XHZ, 9WB1XBZ, 9WB1XCZ, 9WB1XDZ, 9WB1XFZ, 9WB1XKZ, 9WB1XLZ, 9WB2XBZ, 9WB2XCZ, 9WB2XDZ, 9WB2XFZ, 9WB2XKZ, 9WB2XLZ, 9WB3XBZ, 9WB3XCZ, 9WB3XDZ, 9WB3XFZ, 9WB3XKZ, 9WB3XLZ, 9WB4XGZ, 9WB4XHZ, 9WB5XGZ, PWB5XHZ, 9WB6XBZ, 9WB6XCZ, 9WB6XDZ, 9WB6XFZ, 9WB6XKZ, 9WB6XLZ, 9WB7XBZ, 9WB7XCZ, 9WB7XDZ, 9WB7XFZ, 9WB7XKZ, 9WB7XLZ, 9WB8XBZ, 9WB8XCZ, 9WB8XDZ, 9WB8XFZ, 9WB8XKZ, 9WB8XLZ, 9WB9XGZ, 9WB9XHZ, 9WB0XJZ, 9WB1XGZ, 9WB1XHZ, 9WB1XJZ, 9WB2XGZ, 9WB2XHZ, 9WB2XJZ, 9WB3XGZ, 9WB3XHZ, 9WB3XJZ, 9WB4XJZ, 9WB5XJZ, 9WB6XGZ, 9WB6XHZ, 9WB6XJZ, 9WB7XGZ, 9WB7XHZ, 9WB7XJZ, 9WB8XGZ, 9WB8XHZ, 9WB8XJZ, 9WB9XJZ
	CPT	98940, 98941, 98942, 98943
	HCPCS	G0065
Physical Therapy	ICD-9	93.1, 93.11, 93.12, 93.13, 93.14, 93.15, 93.16, 93.17, 93.18, 93.19, 93.2, 93.21, 93.22, 93.25, 93.26, 93.27, 93.28, 93.29, 93.3, 93.31, 93.32, 93.33, 93.34, 93.35, 93.38, 93.39, V57.1
	SNOMED	424203006, 424291000, 410160006, 183326003, 91251008, 410158009, 410159001, 722138006, 722140001
	ICD-10	F0700EZ, F0700FZ, F0700UZ, F0700YZ, F0700ZZ, F0701EZ, F0701FZ, F0701UZ, F0701YZ, F0701ZZ, F0702EZ, F0702FZ, F0702UZ, F0702YZ, F0702ZZ, F0703EZ, F0703FZ, F0703UZ, F0703YZ, F0703ZZ, F0706BZ, F0706CZ, F0706DZ, F0706EZ, F0706FZ, F0706GZ, F0706HZ, F0706UZ, F0706YZ, F0706ZZ, F0707ZZ, F0710EZ, F0710FZ, F0710UZ, F0710YZ, F0710ZZ, F0711EZ, F0711FZ, F0711UZ, F0711YZ, F0711ZZ, F0712EZ, F0712FZ, F0712UZ, F0712YZ, F0712ZZ, F0713EZ, F0713FZ, F0713UZ, F0713YZ, F0713ZZ,

	F0716BZ, F0716CZ, F0716DZ, F0716EZ, F0716FZ, F0716GZ, F0716HZ,<br>F0716UZ, F0716YZ, F0716ZZ, F0717ZZ,<br>F0720EZ, F0720FZ, F0720UZ, F0720YZ, F0720ZZ, F0721EZ, F0721FZ,<br>F0721UZ, F0721YZ, F0721ZZ, F0722EZ, F0722FZ, F0722UZ, F0722YZ,<br>F0722ZZ, F0723EZ, F0723FZ, F0723UZ, F0723YZ, F0723ZZ,<br>F0726BZ, F0726CZ, F0726DZ, F0726EZ, F0726FZ, F0726GZ, F0726HZ,<br>F0726UZ, F0726YZ, F0726ZZ,<br>F0727ZZ, F0730EZ, F0730FZ, F0730UZ, F0730YZ, F0730ZZ, F0731EZ,<br>F0731FZ, F0731UZ, F0731YZ, F0731ZZ, F0732EZ, F0732FZ, F0732UZ,<br>F0732YZ, F0732ZZ, F0733EZ, F0733FZ, F0733UZ, F0733YZ, F0733ZZ,<br>F0736BZ, F0736CZ, F0736DZ, F0736EZ, F0736FZ, F0736GZ, F0736HZ,<br>F0736UZ, F0736YZ, F0736ZZ, F0737ZZ,<br>F0746BZ, F0746CZ, F0746DZ, F0746EZ, F0746FZ, F0746GZ, F0746HZ,<br>F0746UZ, F0746YZ, F0746ZZ, F0756BZ, F0756CZ, F0756DZ, F0756EZ,<br>F0756FZ, F0756GZ, F0756HZ, F0756UZ, F0756YZ, F0756ZZ,<br>F0766BZ, F0766CZ, F0766DZ, F0766EZ, F0766FZ, F0766GZ, F0766HZ,<br>F0766UZ, F0766YZ, F0766ZZ,<br>F0776BZ, F0776CZ, F0776DZ, F0776EZ, F0776FZ, F0776GZ, F0776HZ,<br>F0776UZ, F0776YZ, F0776ZZ,<br>F07D0EZ, F07D0FZ, F07D0UZ, F07D0YZ, F07D0ZZ, F07D1EZ, F07D1FZ,<br>F07D1UZ, F07D1YZ, F07D1ZZ, F07D2EZ, F07D2FZ, F07D2UZ,<br>F07D2YZ, F07D2ZZ, F07D3EZ, F07D3FZ, F07D3UZ, F07D3YZ, F07D3ZZ,<br>F07D6BZ, F07D6CZ, F07D6DZ, F07D6EZ, F07D6FZ, F07D6GZ,<br>F07D6HZ, F07D6UZ, F07D6YZ, F07D6ZZ, F07D7ZZ, F07F0EZ, F07F0FZ,<br>F07F0UZ, F07F0YZ, F07F0ZZ, F07F1EZ, F07F1FZ, F07F1UZ, F07F1YZ,<br>F07F1ZZ, F07F2EZ, F07F2FZ, F07F2UZ, F07F2YZ, F07F2ZZ, F07F3EZ,<br>F07F3FZ, F07F3UZ, F07F3YZ, F07F3ZZ, F07F6BZ, F07F6CZ, F07F6DZ,<br>F07F6EZ, F07F6FZ, F07F6GZ, F07F6HZ, F07F6UZ, F07F6YZ, F07F6ZZ,<br>F07F7ZZ,<br>F07G0EZ, F07G0FZ, F07G0UZ, F07G0YZ, F07G0ZZ, F07G1EZ, F07G1FZ,<br>F07G1UZ, F07G1YZ, F07G1ZZ, F07G2EZ, F07G2FZ, F07G2UZ,<br>F07G2YZ, F07G2ZZ, F07G3EZ, F07G3FZ, F07G3UZ, F07G3YZ, F07G3ZZ,<br>F07G6BZ, F07G6CZ, F07G6DZ, F07G6EZ, F07G6FZ, F07G6GZ,<br>F07G6HZ, F07G6UZ, F07G6YZ, F07G6ZZ, F07G7ZZ, F07J0EZ, F07J0FZ,<br>F07J0UZ, F07J0YZ, F07J0ZZ, F07J1EZ, F07J1FZ, F07J1UZ, F07J1YZ,<br>F07J1ZZ, F07J2EZ, F07J2FZ, F07J2UZ, F07J2YZ, F07J2ZZ, F07J3EZ,<br>F07J3FZ, F07J3UZ, F07J3YZ, F07J3ZZ, F07J6BZ, F07J6CZ, F07J6DZ,<br>F07J6EZ, F07J6FZ, F07J6GZ, F07J6HZ, F07J6UZ, F07J6YZ, F07J6ZZ,<br>F07J7ZZ, F07K0EZ, F07K0FZ, F07K0UZ, F07K0YZ, F07K0ZZ, F07K1EZ,<br>F07K1FZ, F07K1UZ, F07K1YZ, F07K1ZZ, F07K2EZ, F07K2FZ, F07K2UZ,<br>F07K2YZ, F07K2ZZ, F07K3EZ, F07K3FZ, F07K3UZ, F07K3YZ, F07K3ZZ,<br>F07K6BZ, F07K6CZ, F07K6DZ, F07K6EZ, F07K6FZ, F07K6GZ,<br>F07K6HZ, F07K6UZ, F07K6YZ, F07K6ZZ, F07K7ZZ, F07L0EZ, F07L0FZ,<br>F07L0UZ, F07L0YZ, F07L0ZZ, F07L1EZ, F07L1FZ, F07L1UZ, F07L1YZ,<br>F07L1ZZ, F07L2EZ, F07L2FZ, F07L2UZ, F07L2YZ, F07L2ZZ, F07L3EZ,

		F07L3FZ, F07L3UZ, F07L3YZ, F07L3ZZ, F07L6BZ, F07L6CZ, F07L6DZ, F07L6EZ, F07L6FZ, F07L6GZ, F07L6HZ, F07L6UZ, F07L6YZ, F07L6ZZ, F07L7ZZ, F07M0EZ, F07M0FZ, F07M0UZ, F07M0YZ, F07M0ZZ, F07M1EZ, F07M1FZ, F07M1UZ, F07M1YZ, F07M1ZZ, F07M2EZ, F07M2FZ, F07M2UZ, F07M2YZ, F07M2ZZ, F07M3EZ, F07M3FZ, F07M3UZ, F07M3YZ, F07M3ZZ, F07M6BZ, F07M6CZ, F07M6DZ, F07M6EZ, F07M6FZ, F07M6GZ, F07M6HZ, F07M6UZ, F07M6YZ, F07M6ZZ, F07M7ZZ, F07Z4DZ, F07Z4EZ, , F07Z4FZ, F07Z4UZ, F07Z4YZ, F07Z4ZZ, F07Z5CZ, F07Z5EZ, F07Z5FZ, F07Z5UZ, F07Z5YZ, F07Z5ZZ, F07Z8CZ, F07Z8DZ, F07Z8EZ, F07Z8FZ, F07Z8UZ, F07Z8ZZ, F07Z9CZ, F07Z9DZ, F07Z9EZ, F07Z9FZ, F07Z9GZ, F07Z9UZ, F07Z9YZ, F07Z9ZZ
	CPT	94667, 94668, 97014, 97032, 97035, 97039, 97110, 97140, 97161, 97162, 97163, 97164, 97750, 97799
	HCPCS	G2168, G0040, G0151, G0157, G0159, G4026, S8990, S9131
Massage therapy	SNOMED	387854002, 229488002, 229489005, 432085004, 440644000, 182612008, 229486003, 229499000, 229500009, 229501008, 229502001, 229503006, 229506003, 229507007, 309688004, 386438000, 387856000, 440240000, 440406003, 444934009, 444983008, 439822002
	ICD-10	8E0KX1Z
	CPT	97124
Osteopathic Manipulation	ICD-9	93.6, 93.61, 93.62, 93.63, 93.64, 93.65, 93.66, 93.67
	SNOMED	16992002, 65361000, 416278003, 417602003, 723231001, 416815004, 591261000124105, 19859006, 416244009, 416304004, 416327007, 416355006, 416424001, 416615002, 416689004, 416723008, 416760007, 416797002, 416899006, 417656005, 417665003, 461901000124108, 732219004, 73997003, 773517000, 591281000124100, 456391000124104
	ICD-10	7W00X0Z, 7W00X1Z, 7W00X2Z, 7W00X3Z, 7W00X4Z, 7W00X5Z, 7W00X6Z, 7W00X7Z, 7W00X8Z, 7W00X9Z, 7W01X0Z, 7W01X1Z, 7W01X2Z, 7W01X3Z, 7W01X4Z, 7W01X5Z, 7W01X6Z, 7W01X7Z, 7W01X9Z, 7W02X0Z, 7W02X1Z, 7W02X2Z, 7W02X3Z, 7W02X4Z, 7W02X5Z, 7W02X6Z, 7W02X7Z, 7W02X8Z, 7W02X9Z, 7W03X0Z, 7W03X1Z, 7W03X2Z, 7W03X3Z, 7W03X4Z, 7W03X5Z, 7W03X6Z, 7W03X7Z, 7W03X8Z, 7W03X9Z, 7W04X0Z, 7W04X1Z, 7W04X2Z, 7W04X3Z, 7W04X4Z, 7W04X5Z, 7W04X6Z, 7W04X7Z, 7W04X8Z, 7W04X9Z, 7W05X0Z, 7W05X1Z, 7W05X2Z, 7W05X3Z, 7W05X4Z, 7W05X5Z, 7W05X6Z, 7W05X7Z, 7W05X8Z, 7W05X9Z, 7W06X0Z, 7W06X1Z, 7W06X2Z, 7W06X3Z, 7W06X4Z, 7W06X5Z, 7W06X6Z, 7W06X7Z, 7W06X8Z, 7W06X9Z, 7W07X0Z, 7W07X1Z, 7W07X2Z, 7W07X3Z, 7W07X4Z, 7W07X5Z, 7W07X6Z, 7W07X7Z, 7W07X8Z, 7W07X9Z, 7W08X0Z, 7W08X1Z, 7W08X2Z, 7W08X3Z, 7W08X4Z, 7W08X5Z, 7W08X6Z, 7W08X7Z, 7W08X8Z,

		7W08X9Z, 7W09X0Z, 7W09X1Z, 7W09X2Z, 7W09X3Z, 7W09X4Z, 7W09X5Z, 7W09X6Z, 7W09X7Z, 7W09X8Z, 7W09X9Z
Yoga	CPT	98925, 98926, 98927, 98928, 98929
	ICD-9	93.12, E005.1
	SNOMED	1259940003, 229224000
	ICD-10	8E0ZXY4, Y93.42
Psychotherapy/cognitive behavioral therapy	ICD-9	94.3, 94.31, 94.32, 94.33, 94.37, 94.38, 94.39
	SNOMED	166001, 15558000, 183382003, 314034001, 38678006, 15550055, 183381005, 183383008, 183385001, 183398005, 18512000, 266744007, 27591006, 302236003, 304821008, 304822001, 304825004, 304826003, 305878005, 310124002, 361230002, 394913002, 401157001, 440646003, 443730003, 76168009, 85925008, 302235004
	ICD-10	GZ50ZZZ, GZ51ZZZ, GZ52ZZZ, GZ53ZZZ, GZ54ZZZ, GZ55ZZZ, GZ56ZZZ, G58ZZZ, GZ59ZZZ, GHZZZZ, HZ50ZZZ, HZ51ZZZ, HZ52ZZZ, HZ53ZZZ, HZ54ZZZ, HZ55ZZZ, HZ56ZZZ, HZ57ZZZ, HZ58ZZZ, HZ59ZZZ, HZ5BZZZ, HZ5CZZZ, HZ5CZZZ, HZ5DZZZ, HZ32ZZZ, HZ42ZZZ, HZ30ZZZ, HZ40ZZZ, HZ31ZZZ, HZ41ZZZ,
	CPT	G0323, G0410, G0411, 90832, 90833, 90834, 90836, 90837, 90838, 90845, 90853, 90875, 90876, 90880, 99510
Transcutaneous electrical nerve stimulation (TENS)	SNOMED	229559001, 231110003, 770743001, 700669005, 710917005, 725868009
	ICD-10	0KWXXMZ, 0KWYXMZ
	HCPCS	E0720, E0730

1 Specific types chosen based on a comprehensive search of numerous complementary medicine subtypes (additional types inclusive of such examples as Tai Chi/Qigong, meditation, low-level laser therapy, mindfulness-based stress reduction, etc.) and final list was chosen based on those that had any capture in the database

Supplemental Table 9: List of codes used to define outpatient SUD-related services

Code Type	Code(s)
CPT	90791, 90792, 90832, 90833, 90834, 90835, 90836, 90837, 90838, 90839, 90840, 90845, 90846, 90847, 90848, 90849, 90853, 90857, 90865, 90867, 90868, 90869, 90870,90871,90875,90876, 90880, 90900, 90901, 90902, 90904, 90906, 90908, 90910, 97003, 97004, 98960, 98961, 98962, 99058, 99078, 99201, 99202, 99203, 99204, 99205, 99211, 99212, 99213, 99214, 99215, 99241, 99242, 99243, 99244, 99245, 99341, 99342, 99343, 99344, 99345, 99347, 99348, 99349, 99350, 99382, 99383, 99384, 99385, 99386, 99387, 99392, 99393, 99394, 99395, 99396, 99397, 99401, 99402, 99403, 99404, 99408, 99409, 99411, 99412, 99420, 99441, 99442, 99443, 99843, 99490, 99495, 99496, 99510, 0359T, 0360T, 0361T, 0362T, 0363T, 0364T, 0365T, 0366T, 0367T, 0368T, 0369T, 0370T, 0371T, 0372T, 0373T,0374T
HCPCS	G0155, G0175, G0351, G0396, G0397, G0438, G0439, G0442, G0443, G0463, G0466, G0467, G0468, G0469, G0470, G0505, G0507, G0513, G0514, G0515, G2025, G2067, G2068, G2069, G2080, G2086, G2087, G2088, H0001, H0002, H0003, H0004, H0005, H0006, H0007, H0014, H0016, H0022, H0023, H0028, H0029, H0031, H0034, H0036, H0037, H0038, H0039, H0040, H0041, H0042, H0043, H0044, H0045, H0046, H0047, H0048, H0049, H0050, H1011, H2000, H2001, H2010, H2011, H2012, H2013, H2014, H2015, H2016, H2017, H2018, H2019, H2020, H2021, H2022, H2023, H2024, H2025, H2026, H2027, H2028, H2029, H2030, H2031, H2032, H2033, H2037, H5010, H5020, H5025, H5030, H5220, H5230, H5240, H5299, M0064, S9454, S9482, S9484, S9485, T1006, T1007, T1011, T1012, T1015, T1016, T1017, T1018, T1023, T1024, T1025, T1026, T1027, T1040, T1041, T2010, T2011, T2012, T2013, T2014, T2015, T2018, T2019, T2020, T2021, T2022, T2023, T2024, T2036, T2037, Z0001, Z0002

Supplemental Table 10a: Predicted[1] mean pain severity scores (95% confidence intervals) by opioid Rx dosing/duration[2] and time (in months) since first pain severity assessment among patients with OUD, on and up to two years after first pain severity score

	**Opioid Rx**						
	No opioid Rx	MME < 50, Duration <30 days	MME >=50, Duration < 30 days	MME < 50, Duration >=30 days & Duration <=90 days	MME >=50, Duration >=30 days & Duration <=90 days	MME <50, Duration >90 days	MME >=50, Duration >90 days
**n (%[3])**	40887 (22.8)	43931 (24.5)	8274 (4.6)	16318 (9.1)	2492 (1.4)	55472 (30.9)	12257 (6.8)
**Time (in months)**	**Mean (95% CI)**						
1	3.51 (3.50, 3.52)	4.42 (4.41, 4.43)	4.63 (4.62, 4.64)	4.62 (4.61, 4.63)	4.74 (4.72, 4.75)	4.71 (4.70, 4.72)	4.78 (4.77, 4.79)
2	3.36 (3.35, 3.38)	4.08 (4.07, 4.09)	4.36 (4.33, 4.38)	4.59 (4.58, 4.60)	4.68 (4.66, 4.71)	4.74 (4.73, 4.75)	4.69 (4.68, 4.70)
3	3.45 (3.43, 3.46)	4.06 (4.05, 4.07)	4.22 (4.19, 4.25)	4.48 (4.47, 4.49)	4.60 (4.57, 4.62)	4.75 (4.74, 4.76)	4.75 (4.74, 4.77)
4	3.42 (3.40, 3.43)	4.07 (4.06, 4.08)	4.07 (4.04, 4.10)	4.29 (4.27, 4.30)	4.22 (4.18, 4.25)	4.74 (4.73, 4.75)	4.76 (4.75, 4.77)
5	3.45 (3.43, 3.46)	4.09 (4.08, 4.10)	4.22 (4.19, 4.24)	4.29 (4.27, 4.30)	4.20 (4.17, 4.24)	4.72 (4.71, 4.73)	4.71 (4.69, 4.72)
6	3.43 (3.41, 3.45)	4.06 (4.05, 4.07)	4.24 (4.22, 4.27)	4.17 (4.16, 4.18)	4.14 (4.11, 4.18)	4.69 (4.68, 4.70)	4.71 (4.70, 4.73)
7	3.67 (3.65, 3.68)	4.09 (4.08, 4.11)	4.33 (4.29, 4.36)	4.29 (4.27, 4.30)	4.26 (4.22, 4.30)	4.70 (4.69, 4.71)	4.61 (4.59, 4.62)
8	3.86 (3.85, 3.88)	4.20 (4.19, 4.22)	4.48 (4.45, 4.52)	4.37 (4.36, 4.39)	4.63 (4.59, 4.67)	4.74 (4.73, 4.75)	4.69 (4.67, 4.70)
9	3.88 (3.87, 3.90)	4.27 (4.26, 4.28)	4.48 (4.45, 4.51)	4.46 (4.45, 4.48)	4.66 (4.61, 4.71)	4.76 (4.75, 4.77)	4.81 (4.79, 4.82)
10	3.91 (3.89, 3.92)	4.27 (4.25, 4.28)	4.47 (4.43, 4.50)	4.46 (4.44, 4.48)	4.63 (4.58, 4.68)	4.82 (4.81, 4.83)	4.77 (4.75, 4.79)
11	3.95 (3.93, 3.96)	4.32 (4.31, 4.33)	4.59 (4.56, 4.63)	4.47 (4.45, 4.49)	4.59 (4.54, 4.65)	4.82 (4.81, 4.83)	4.80 (4.78, 4.82)
12	3.97 (3.95, 3.99)	4.36 (4.35, 4.37)	4.57 (4.53, 4.61)	4.45 (4.44, 4.47)	4.66 (4.60, 4.72)	4.84 (4.83, 4.85)	4.77 (4.75, 4.79)
13	3.99 (3.97, 4.00)	4.26 (4.25, 4.28)	4.43 (4.39, 4.46)	4.51 (4.49, 4.52)	4.61 (4.56, 4.67)	4.85 (4.84, 4.86)	4.75 (4.73, 4.77)
14	4.01 (3.99, 4.03)	4.33 (4.31, 4.34)	4.41 (4.37, 4.45)	4.51 (4.48, 4.53)	4.50 (4.43, 4.57)	4.83 (4.82, 4.84)	4.79 (4.77, 4.82)
15	3.98 (3.96, 4.00)	4.29 (4.27, 4.30)	4.42 (4.39, 4.46)	4.54 (4.52, 4.56)	4.55 (4.50, 4.61)	4.84 (4.83, 4.85)	4.71 (4.69, 4.73)
16	4.00 (3.99, 4.02)	4.26 (4.24, 4.27)	4.54 (4.51, 4.57)	4.49 (4.47, 4.51)	4.70 (4.64, 4.76)	4.86 (4.85, 4.87)	4.77 (4.75, 4.79)
17	4.06 (4.04, 4.08)	4.31 (4.30, 4.33)	4.53 (4.50, 4.57)	4.45 (4.43, 4.47)	4.44 (4.38, 4.50)	4.84 (4.83, 4.85)	4.82 (4.80, 4.84)
18	3.99 (3.97, 4.01)	4.32 (4.31, 4.34)	4.52 (4.48, 4.56)	4.51 (4.49, 4.53)	4.58 (4.53, 4.64)	4.83 (4.82, 4.84)	4.74 (4.72, 4.76)
19	4.04 (4.02, 4.06)	4.33 (4.31, 4.34)	4.48 (4.44, 4.52)	4.47 (4.45, 4.49)	4.67 (4.61, 4.72)	4.85 (4.84, 4.86)	4.81 (4.79, 4.83)
20	4.07 (4.05, 4.09)	4.26 (4.25, 4.28)	4.55 (4.52, 4.59)	4.44 (4.42, 4.47)	4.61 (4.55, 4.67)	4.81 (4.80, 4.82)	4.82 (4.80, 4.84)
21	4.15 (4.12, 4.17)	4.31 (4.29, 4.33)	4.45 (4.41, 4.50)	4.38 (4.35, 4.41)	4.51 (4.43, 4.60)	4.79 (4.78, 4.80)	4.66 (4.64, 4.69)
22	4.12 (4.10, 4.14)	4.30 (4.29, 4.32)	4.43 (4.39, 4.47)	4.43 (4.41, 4.45)	4.58 (4.52, 4.65)	4.81 (4.80, 4.82)	4.81 (4.79, 4.83)
23	4.14 (4.12, 4.16)	4.26 (4.24, 4.27)	4.46 (4.42, 4.50)	4.50 (4.47, 4.53)	4.46 (4.40, 4.52)	4.78 (4.77, 4.79)	4.83 (4.81, 4.85)
24	4.13 (4.11, 4.15)	4.29 (4.27, 4.31)	4.52 (4.48, 4.56)	4.40 (4.38, 4.42)	4.59 (4.52, 4.66)	4.80 (4.79, 4.81)	4.79 (4.77, 4.82)

1 linear mixed-effects regression adjusting for age, gender, race, census region, insurance, year of first pain severity score, comorbidity, chronic pain, one-year procedure history, mental health condition history, other SUD conditions (for OUD and SUD models), opioid prescribing*MOUD, MSUD (for SUD model), opioid prescribing*CM/OS services (just CM for control model), with hospital ID and patient ID as crossed random effects **2** from up to one month before first pain severity score and up to six months later **3** row % out of overall n

Supplemental Table 10b: Predicted[1] mean pain severity scores (95% confidence intervals) by opioid Rx dosing/duration[2] and time (in months) since first pain severity assessment among patients with other SUD, on and up to two years after first pain severity score

	Opioid Rx						
	No opioid Rx	MME < 50, Duration <30 days	MME >=50, Duration < 30 days	MME < 50, Duration >=30 days & Duration <=90 days	MME >=50, Duration >=30 days & Duration <=90 days	MME <50, Duration >90 days	MME >=50, Duration >90 days
**n (%[3])**	274217 (49.1)	144314 (25.9)	18306 (3.3)	33142 (5.9)	3303 (0.6)	74853 (13.4)	9856 (1.8)
**Time (in months)**	**Mean (95% CI)**						
1	2.73 (2.72, 2.74)	4.06 (4.05, 4.07)	4.04 (4.03, 4.05)	4.32 (4.31, 4.33)	4.18 (4.16, 4.19)	4.46 (4.45, 4.47)	4.44 (4.43, 4.45)
2	2.65 (2.64, 2.66)	3.68 (3.67, 3.69)	3.65 (3.63, 3.66)	4.28 (4.27, 4.29)	4.09 (4.06, 4.12)	4.48 (4.47, 4.49)	4.38 (4.36, 4.40)
3	2.70 (2.69, 2.71)	3.66 (3.65, 3.67)	3.58 (3.56, 3.60)	4.21 (4.20, 4.22)	3.95 (3.92, 3.98)	4.49 (4.48, 4.50)	4.49 (4.46, 4.51)
4	2.75 (2.74, 2.75)	3.68 (3.67, 3.69)	3.54 (3.52, 3.57)	4.07 (4.06, 4.08)	3.98 (3.93, 4.02)	4.50 (4.49, 4.51)	4.45 (4.43, 4.48)
5	2.76 (2.75, 2.77)	3.71 (3.70, 3.72)	3.62 (3.59, 3.64)	4.08 (4.07, 4.10)	3.82 (3.77, 3.87)	4.47 (4.46, 4.48)	4.33 (4.31, 4.35)
6	2.80 (2.79, 2.81)	3.73 (3.72, 3.74)	3.65 (3.63, 3.67)	4.07 (4.06, 4.08)	3.82 (3.76, 3.87)	4.47 (4.46, 4.48)	4.34 (4.32, 4.36)
7	3.05 (3.04, 3.06)	3.84 (3.83, 3.85)	3.74 (3.71, 3.77)	4.13 (4.11, 4.14)	3.79 (3.73, 3.85)	4.49 (4.48, 4.50)	4.61 (4.58, 4.64)
8	3.18 (3.17, 3.19)	3.89 (3.88, 3.90)	3.77 (3.74, 3.80)	4.15 (4.13, 4.16)	3.81 (3.74, 3.87)	4.51 (4.50, 4.52)	4.50 (4.47, 4.53)
9	3.20 (3.20, 3.21)	3.92 (3.91, 3.93)	3.87 (3.83, 3.90)	4.19 (4.18, 4.21)	4.07 (4.00, 4.13)	4.49 (4.48, 4.50)	4.38 (4.34, 4.41)
10	3.25 (3.24, 3.25)	3.91 (3.90, 3.92)	3.90 (3.87, 3.94)	4.18 (4.16, 4.20)	3.98 (3.91, 4.05)	4.51 (4.50, 4.52)	4.46 (4.42, 4.49)
11	3.23 (3.22, 3.24)	3.95 (3.94, 3.96)	3.85 (3.82, 3.88)	4.16 (4.14, 4.18)	4.20 (4.13, 4.27)	4.53 (4.52, 4.54)	4.40 (4.36, 4.43)
12	3.25 (3.24, 3.26)	3.95 (3.94, 3.96)	3.83 (3.79, 3.86)	4.16 (4.14, 4.18)	4.12 (4.04, 4.20)	4.56 (4.54, 4.57)	4.51 (4.47, 4.54)
13	3.24 (3.23, 3.25)	3.95 (3.94, 3.96)	3.81 (3.77, 3.84)	4.21 (4.19, 4.23)	4.03 (3.97, 4.09)	4.51 (4.50, 4.52)	4.44 (4.41, 4.48)
14	3.33 (3.32, 3.34)	3.87 (3.85, 3.88)	3.82 (3.78, 3.86)	4.22 (4.19, 4.24)	3.98 (3.89, 4.08)	4.53 (4.52, 4.55)	4.38 (4.34, 4.42)
15	3.30 (3.29, 3.30)	3.91 (3.90, 3.92)	3.85 (3.82, 3.89)	4.23 (4.21, 4.25)	4.18 (4.11, 4.25)	4.47 (4.46, 4.48)	4.45 (4.41, 4.48)
16	3.29 (3.28, 3.30)	3.92 (3.91, 3.93)	3.82 (3.79, 3.86)	4.20 (4.18, 4.22)	4.06 (3.99, 4.13)	4.51 (4.50, 4.52)	4.60 (4.56, 4.63)
17	3.35 (3.34, 3.36)	3.92 (3.91, 3.93)	3.88 (3.84, 3.92)	4.18 (4.15, 4.20)	4.17 (4.09, 4.24)	4.51 (4.50, 4.52)	4.55 (4.50, 4.59)
18	3.37 (3.36, 3.38)	3.95 (3.94, 3.96)	3.90 (3.86, 3.94)	4.10 (4.08, 4.12)	4.12 (4.05, 4.19)	4.51 (4.49, 4.52)	4.43 (4.39, 4.47)
19	3.37 (3.36, 3.38)	3.94 (3.93, 3.95)	3.95 (3.90, 3.99)	4.11 (4.09, 4.14)	4.10 (4.01, 4.19)	4.49 (4.48, 4.50)	4.46 (4.41, 4.51)
20	3.43 (3.42, 3.44)	3.92 (3.91, 3.93)	3.77 (3.73, 3.81)	4.10 (4.08, 4.13)	4.44 (4.34, 4.53)	4.51 (4.50, 4.52)	4.49 (4.44, 4.54)
21	3.41 (3.40, 3.42)	3.92 (3.90, 3.94)	3.77 (3.72, 3.82)	4.12 (4.09, 4.15)	4.43 (4.34, 4.53)	4.45 (4.43, 4.47)	4.47 (4.42, 4.52)
22	3.42 (3.41, 3.43)	3.90 (3.89, 3.92)	3.78 (3.74, 3.82)	4.19 (4.16, 4.21)	4.43 (4.36, 4.51)	4.46 (4.44, 4.47)	4.54 (4.49, 4.58)
23	3.45 (3.44, 3.46)	3.89 (3.87, 3.90)	3.75 (3.71, 3.79)	4.09 (4.07, 4.12)	4.40 (4.31, 4.50)	4.44 (4.43, 4.46)	4.43 (4.38, 4.48)
24	3.48 (3.47, 3.49)	3.91 (3.90, 3.93)	3.70 (3.66, 3.75)	4.14 (4.11, 4.16)	3.99 (3.91, 4.08)	4.44 (4.43, 4.46)	4.47 (4.42, 4.52)

1 linear mixed-effects regression adjusting for age, gender, race, census region, insurance, year of first pain severity score, comorbidity, chronic pain, one-year procedure history, mental health condition history, other SUD conditions (for OUD and SUD models), opioid prescribing*MOUD, MSUD (for SUD model), opioid prescribing*CM/OS services (just CM for control model), with hospital ID and patient ID as crossed random effects **2** from up to one month before first pain severity score and up to six months later **3** row % out of overall n

Supplemental Table 10c: Predicted[1] mean pain severity scores (95% confidence intervals) by opioid Rx dosing/duration[2] and time (in months) since first pain severity assessment among control patients (without OUD and other SUD), on and up to two years after first pain severity score

	**Opioid Rx**						
	No opioid Rx	MME < 50, Duration <30 days	MME >=50, Duration < 30 days	MME < 50, Duration >=30 days & Duration <=90 days	MME >=50, Duration >=30 days & Duration <=90 days	MME <50, Duration >90 days	MME >=50, Duration >90 days
**n (%[3])**	399659 (54.2)	188395 (25.6)	33374 (4.5)	31449 (4.3)	4350 (0.6)	67953 (9.2)	12033 (1.6)
**Time (in months)**	**Mean (95% CI)**						
1	2.56 (2.55, 2.57)	3.66 (3.65, 3.67)	3.63 (3.62, 3.64)	3.88 (3.87, 3.89)	3.86 (3.85, 3.88)	3.63 (3.51, 3.74)	4.02 (4.01, 4.03)
2	2.39 (2.38, 2.40)	3.23 (3.22, 3.24)	3.26 (3.25, 3.28)	3.81 (3.80, 3.82)	3.77 (3.75, 3.79)	3.98 (3.97, 3.99)	3.96 (3.93, 3.98)
3	2.42 (2.41, 2.43)	3.22 (3.21, 3.23)	3.26 (3.25, 3.28)	3.76 (3.75, 3.77)	3.64 (3.61, 3.67)	3.92 (3.91, 3.93)	3.91 (3.88, 3.93)
4	2.41 (2.40, 2.42)	3.26 (3.25, 3.27)	3.29 (3.27, 3.31)	3.49 (3.48, 3.51)	3.65 (3.60, 3.70)	3.92 (3.91, 3.93)	3.98 (3.95, 4.00)
5	2.46 (2.45, 2.47)	3.26 (3.25, 3.27)	3.21 (3.19, 3.24)	3.42 (3.40, 3.44)	3.44 (3.38, 3.51)	3.93 (3.92, 3.94)	3.84 (3.82, 3.87)
6	2.50 (2.49, 2.51)	3.29 (3.28, 3.30)	3.25 (3.23, 3.28)	3.46 (3.44, 3.48)	3.40 (3.34, 3.46)	3.89 (3.88, 3.90)	3.89 (3.87, 3.92)
7	2.80 (2.79, 2.81)	3.41 (3.40, 3.42)	3.29 (3.26, 3.31)	3.54 (3.51, 3.56)	3.42 (3.36, 3.48)	3.86 (3.85, 3.87)	3.99 (3.96, 4.03)
8	2.90 (2.89, 2.91)	3.42 (3.41, 3.43)	3.34 (3.31, 3.37)	3.51 (3.49, 3.54)	3.43 (3.36, 3.50)	3.92 (3.91, 3.93)	3.95 (3.91, 3.98)
9	2.85 (2.84, 2.86)	3.44 (3.43, 3.45)	3.45 (3.42, 3.48)	3.53 (3.51, 3.56)	3.59 (3.52, 3.65)	3.86 (3.84, 3.87)	4.01 (3.97, 4.05)
10	2.84 (2.83, 2.85)	3.47 (3.46, 3.49)	3.45 (3.42, 3.48)	3.59 (3.57, 3.62)	3.31 (3.23, 3.39)	3.88 (3.87, 3.90)	4.00 (3.95, 4.04)
11	2.89 (2.88, 2.90)	3.45 (3.44, 3.47)	3.40 (3.37, 3.43)	3.60 (3.57, 3.63)	3.76 (3.69, 3.84)	3.88 (3.87, 3.90)	4.05 (4.01, 4.09)
12	2.88 (2.87, 2.89)	3.47 (3.46, 3.48)	3.45 (3.43, 3.48)	3.52 (3.49, 3.55)	3.65 (3.57, 3.74)	3.85 (3.83, 3.87)	3.94 (3.90, 3.98)
13	2.87 (2.86, 2.88)	3.45 (3.44, 3.46)	3.43 (3.41, 3.46)	3.57 (3.54, 3.59)	3.45 (3.38, 3.53)	3.89 (3.88, 3.91)	3.83 (3.79, 3.86)
14	2.88 (2.87, 2.89)	3.47 (3.45, 3.48)	3.47 (3.43, 3.50)	3.45 (3.41, 3.48)	3.32 (3.22, 3.43)	3.84 (3.83, 3.86)	3.94 (3.88, 3.99)
15	2.94 (2.93, 2.95)	3.45 (3.44, 3.46)	3.46 (3.43, 3.49)	3.47 (3.44, 3.49)	3.44 (3.37, 3.51)	3.88 (3.86, 3.90)	3.88 (3.84, 3.93)
16	2.93 (2.93, 2.94)	3.41 (3.40, 3.42)	3.34 (3.32, 3.37)	3.45 (3.42, 3.48)	3.46 (3.37, 3.54)	3.77 (3.75, 3.79)	3.92 (3.88, 3.96)
17	2.93 (2.92, 2.94)	3.43 (3.41, 3.44)	3.36 (3.33, 3.39)	3.39 (3.36, 3.42)	3.56 (3.47, 3.66)	3.79 (3.77, 3.80)	3.76 (3.71, 3.80)
18	2.95 (2.94, 2.96)	3.43 (3.42, 3.44)	3.37 (3.34, 3.40)	3.44 (3.40, 3.47)	3.45 (3.36, 3.53)	3.79 (3.77, 3.81)	3.70 (3.66, 3.75)
19	2.93 (2.92, 2.94)	3.42 (3.41, 3.44)	3.34 (3.31, 3.37)	3.43 (3.40, 3.46)	3.32 (3.24, 3.41)	3.74 (3.72, 3.76)	3.84 (3.79, 3.88)
20	2.98 (2.97, 2.99)	3.45 (3.43, 3.46)	3.47 (3.43, 3.50)	3.38 (3.35, 3.41)	3.54 (3.46, 3.63)	3.79 (3.77, 3.81)	3.81 (3.77, 3.85)
21	3.00 (2.99, 3.02)	3.37 (3.35, 3.39)	3.42 (3.38, 3.46)	3.42 (3.37, 3.46)	3.63 (3.50, 3.75)	3.73 (3.71, 3.74)	3.75 (3.69, 3.80)
22	3.01 (3.00, 3.02)	3.40 (3.39, 3.42)	3.38 (3.35, 3.41)	3.47 (3.44, 3.51)	3.50 (3.40, 3.60)	3.69 (3.66, 3.71)	3.75 (3.71, 3.79)
23	3.01 (3.00, 3.02)	3.39 (3.37, 3.40)	3.35 (3.32, 3.38)	3.44 (3.41, 3.47)	3.48 (3.38, 3.57)	3.71 (3.69, 3.73)	3.74 (3.69, 3.78)
24	3.04 (3.03, 3.05)	3.36 (3.34, 3.37)	3.21 (3.18, 3.24)	3.45 (3.41, 3.48)	3.60 (3.51, 3.69)	3.70 (3.68, 3.72)	3.84 (3.79, 3.89)

1 linear mixed-effects regression adjusting for age, gender, race, census region, insurance, year of first pain severity score, comorbidity, chronic pain, one-year procedure history, mental health condition history, other SUD conditions (for OUD and SUD models), opioid prescribing*MOUD, MSUD (for SUD model), opioid prescribing*CM/OS services (just CM for control model), with hospital ID and patient ID as crossed random effects **2** from up to one month before first pain severity score and up to six months later **3** row % out of overall n

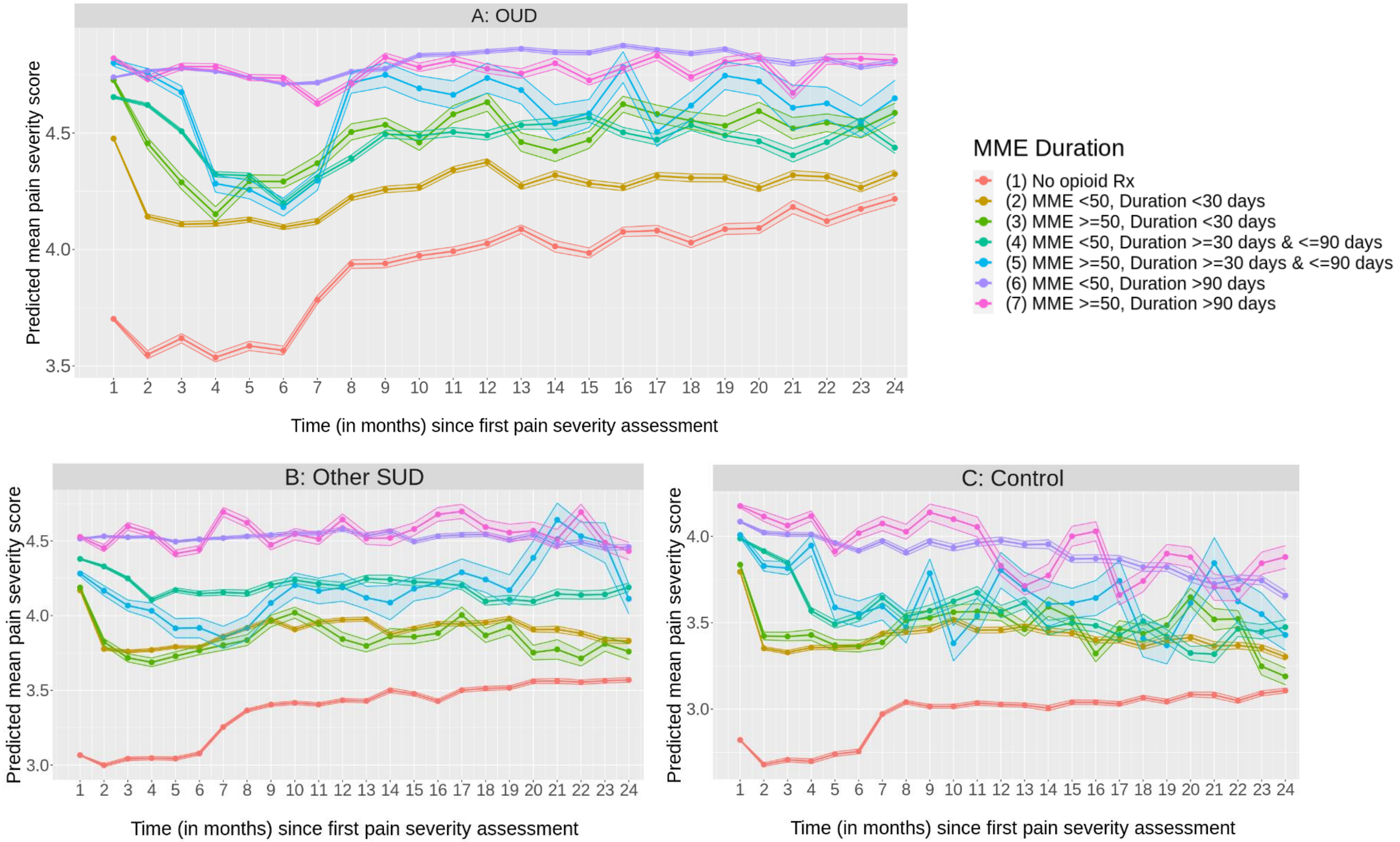

Supplemental Figure 1A-C: Predicted[1] mean pain severity scores (95% confidence intervals) vs. time (in months) since first pain severity assessment (by opioid Rx dosing/duration[2]) among **A**: patients with OUD; **B**: patients with other SUD; **C**: control patients (without OUD or SUD), on and up to two years after first pain severity score, among patients with chronic pain

1 linear mixed-effects regression adjusting for age, gender, race, census region, insurance, year of first pain severity score, comorbidity, one-year procedure history, mental health condition history, other SUD conditions (for OUD and SUD models), opioid Rx*MOUD (for OUD model), MSUD (for SUD model), opioid Rx*CM, opioid Rx*OS (just for OUD and SUD model), with hospital ID and patient ID as crossed random effects **2** prescriptions from up to one month before first pain severity score and up to six months later

Supplemental Table 11: Predicted[1] mean pain severity scores (95% confidence intervals) by opioid Rx dosing/duration[2] and treatment, among patients with OUD, other SUD, controls (without OUD or SUD), on and up to two years after first pain severity score, among patients with chronic pain

**OUD**	**Opioid Rx**						
	No opioid Rx	MME < 50, Duration <30 days	MME >=50, Duration < 30 days	MME < 50, Duration >=30 days & Duration <=90 days	MME >=50, Duration >=30 days & Duration <=90 days	MME <50, Duration >90 days	MME >=50, Duration >90 days
**n (%[3])**	25422 (17.6)	34483 (23.9)	6421 (4.4)	14320 (9.9)	2181 (1.5)	50598 (35.0)	11068 (7.7)
**Mean (95% CI)**	**Overall = 4.58 (4.57, 4.59)**						
**MOUD[4]**							
No	3.83 (3.82, 3.84)	4.30 (4.29, 4.31)	4.57 (4.56, 4.58)	4.49 (4.48, 4.50)	4.81 (4.80, 4.82)	4.77 (4.76, 4.78)	4.85 (4.84, 4.86)
Yes	4.08 (4.07, 4.09)	4.49 (4.48, 4.50)	4.54 (4.51, 4.56)	4.81 (4.80, 4.83)	4.76 (4.70, 4.81)	5.00 (4.99, 5.01)	4.82 (4.80, 4.84)
**CM[5]**							
No	3.85 (3.84, 3.86)	4.33 (4.32, 4.34)	4.58 (4.57, 4.59)	4.53 (4.52, 4.54)	4.66 (4.65, 4.67)	4.79 (4.78, 4.80)	4.78 (4.77, 4.79)
Yes	4.01 (3.99, 4.03)	3.97 (3.96, 3.98)	4.23 (4.20, 4.27)	4.06 (4.05, 4.07)	4.14 (4.10, 4.18)	4.47 (4.46, 4.48)	4.53 (4.51, 4.55)
**OS[6]**							
No	3.83 (3.82, 3.84)	4.32 (4.31, 4.33)	4.57 (4.56, 4.58)	4.51 (4.50, 4.52)	4.66 (4.65, 4.67)	4.78 (4.77, 4.79)	4.79 (4.78, 4.80)
Yes	4.14 (4.13, 4.15)	4.24 (4.23, 4.25)	4.43 (4.41, 4.46)	4.46 (4.45, 4.47)	4.37 (4.33, 4.41)	4.71 (4.70, 4.72)	4.52 (4.50, 4.54)
R^2	0.29						
**Other SUD**	**Opioid Rx**						
	No opioid Rx	MME < 50, Duration <30 days	MME >=50, Duration < 30 days	MME < 50, Duration >=30 days & Duration <=90 days	MME >=50, Duration >=30 days & Duration <=90 days	MME <50, Duration >90 days	MME >=50, Duration >90 days
**n (%[3])**	131496 (42.5)	82628 (26.7)	10501 (3.4)	22726 (7.3)	2241 (0.7)	52777 (17.1)	6888 (2.2)
**Mean (95% CI)**	**Overall = 3.98 (3.97, 3.99)**						
**CM[5]**							
No	3.25 (3.24, 3.26)	4.00 (3.99, 4.01)	4.00 (3.99, 4.01)	4.27 (4.26, 4.28)	4.21 (4.19, 4.22)	4.52 (4.51, 4.53)	4.56 (4.55, 4.57)
Yes	3.41 (3.39, 3.42)	3.76 (3.75, 3.77)	3.57 (3.54, 3.60)	4.23 (4.22, 4.24)	3.72 (3.67, 3.77)	4.50 (4.49, 4.51)	4.54 (4.51, 4.57)
**OS[6]**							
No	3.24 (3.23, 3.25)	4.02 (4.01, 4.03)	4.03 (4.02, 4.04)	4.28 (4.27, 4.29)	4.22 (4.21, 4.23)	4.54 (4.53, 4.55)	4.57 (4.56, 4.58)
Yes	3.38 (3.37, 3.39)	3.78 (3.77, 3.79)	3.64 (3.62, 3.66)	4.13 (4.12, 4.14)	3.89 (3.85, 3.93)	4.39 (4.38, 4.40)	4.26 (4.24, 4.28)
R^2	0.29						
**Control**	**Opioid Rx**						
	No opioid Rx	MME < 50, Duration <30 days	MME >=50, Duration < 30 days	MME < 50, Duration >=30 days & Duration <=90 days	MME >=50, Duration >=30 days & Duration <=90 days	MME <50, Duration >90 days	MME >=50, Duration >90 days
**n (%[3])**	162402 (49.1)	87766 (26.5)	14034 (4.2)	18228 (5.5)	2353 (0.7)	39654 (12.0)	6492 (2.0)
**Mean (95% CI)**	**Overall = 3.47 (3.46, 3.48)**						
**CM[5]**							
No	2.92 (2.91, 2.93)	3.61 (3.60, 3.62)	3.66 (3.65, 3.67)	3.81 (3.80, 3.82)	3.99 (3.95, 4.03)	4.00 (3.99, 4.01)	4.07 (4.06, 4.08)
Yes	2.84 (2.83, 2.85)	3.29 (3.27, 3.30)	3.17 (3.14, 3.20)	3.69 (3.67, 3.71)	3.84 (3.83, 3.85)	3.86 (3.84, 3.87)	4.04 (4.01, 4.07)
R^2	0.31						

1 linear mixed-effects regression adjusting for age, gender, race, census region, insurance, year of first pain severity score, opioid Rx*time (in months) since first pain severity score, comorbidity, one-year procedure history, mental health condition history, other SUD conditions (for OUD and SUD models), MSUD (for SUD model), with hospital ID and patient ID as crossed random effects
2 prescriptions from up to one month before first pain severity score and up to six months later **3** row % out of overall n **4** medications for opioid use disorder **5** complementary medicine **6** outpatient SUD-related services
